# Trametinib versus standard of care in patients with recurrent low-grade serous ovarian cancer (GOG 281/LOGS): an international, randomised, open-label, multicentre, phase 2/3 trial

**DOI:** 10.1016/S0140-6736(21)02175-9

**Published:** 2022-02-05

**Authors:** David M Gershenson, Austin Miller, William E Brady, James Paul, Karen Carty, William Rodgers, David Millan, Robert L Coleman, Kathleen N Moore, Susana Banerjee, Kate Connolly, Angeles Alvarez Secord, David M O'Malley, Oliver Dorigo, Stephanie Gaillard, Hani Gabra, Brian Slomovitz, Parviz Hanjani, John Farley, Michael Churchman, Ailith Ewing, Robert L Hollis, C Simon Herrington, Helen Q Huang, Lari Wenzel, Charlie Gourley

**Affiliations:** a Department of Gynecologic Oncology and Reproductive Medicine, The University of Texas MD Anderson Cancer Center, Houston, TX, USA; b NRG Oncology, Clinical Trial Development Division, Biostatistics & Bioinformatics, Roswell Park Comprehensive Cancer Center, Buffalo, NY, USA; c Cancer Research UK Clinical Trials Unit, Institute of Cancer Sciences, University of Glasgow, Glasgow, UK; d New York Presbyterian/Queens, Department of Pathology, Weill Medical College of Cornell University, Flushing, NY, USA; e Queen Elizabeth University Hospital, Glasgow, UK; f Stephenson Cancer Center at the University of Oklahoma, Oklahoma City, OK, USA; g The Royal Marsden Hospital NHS Foundation Trust, The Institute of Cancer Research, London, UK; h Edinburgh Cancer Centre, Edinburgh, UK; i Duke Cancer Institute, Durham, NC, USA; j The Ohio State University and the James Cancer Center, Columbus, OH, USA; k Stanford Cancer Institute, Stanford University, Stanford, CA, USA; l Johns Hopkins Sidney Kimmel Cancer Center, Baltimore, MD, USA; m Surgery and Cancer, Imperial College London, London, UK; n Division ofGynecologic Oncology, Mount Sinai Medical Center, Miami Beach, FL, USA; o Abington Memorial Hospital, Abington, PA, USA; p St Joseph's Hospital and Medical Center, Phoenix, AZ, USA; q Nicola Murray Centre for Ovarian Cancer Research, Institute of Genetics and Cancer, Western General Hospital, Edinburgh, UK; r MRC Human Genetics Unit and CRUK Edinburgh Centre, MRC Institute of Genetics and Cancer, University of Edinburgh, Western General Hospital, Edinburgh, UK; s Medicine and Public Health, University of California at Irvine, Irvine, CA, USA

## Abstract

**Background:**

Low-grade serous carcinoma of the ovary or peritoneum is characterised by MAPK pathway aberrations and its reduced sensitivity to chemotherapy relative to high-grade serous carcinoma. We compared the MEK inhibitor trametinib to physician's choice standard of care in patients with recurrent low-grade serous carcinoma.

**Methods:**

This international, randomised, open-label, multicentre, phase 2/3 trial was done at 84 hospitals in the USA and UK. Eligible patients were aged 18 years or older with recurrent low-grade serous carcinoma and measurable disease, as defined by Response Evaluation Criteria In Solid Tumors version 1.1, had received at least one platinum-based regimen, but not all five standard-of-care drugs, and had received an unlimited number of previous regimens. Patients with serous borderline tumours or tumours containing low-grade serous and high-grade serous carcinoma were excluded. Eligible patients were randomly assigned (1:1) to receive either oral trametinib 2 mg once daily (trametinib group) or one of five standard-of-care treatment options (standard-of-care group): intravenous paclitaxel 80 mg/m^2^ by body surface area on days 1, 8, and 15 of every 28-day cycle; intravenous pegylated liposomal doxorubicin 40–50 mg/m^2^ by body surface area once every 4 weeks; intravenous topotecan 4 mg/m^2^ by body surface area on days 1, 8, and 15 of every 28-day cycle; oral letrozole 2·5 mg once daily; or oral tamoxifen 20 mg twice daily. Randomisation was stratified by geographical region (USA or UK), number of previous regimens (1, 2, or ≥3), performance status (0 or 1), and planned standard-of-care regimen. The primary endpoint was investigator-assessed progression-free survival while receiving randomised therapy, as assessed by imaging at baseline, once every 8 weeks for 15 months, and then once every 3 months thereafter, in the intention-to-treat population. Safety was assessed in patients who received at least one dose of study therapy. This trial is registered with ClinicalTrials.gov, NCT02101788, and is active but not recruiting.

**Findings:**

Between Feb 27, 2014, and April 10, 2018, 260 patients were enrolled and randomly assigned to the trametinib group (n=130) or the standard-of-care group (n=130). At the primary analysis, there were 217 progression-free survival events (101 [78%] in the trametinib group and 116 [89%] in the standard-of-care group). Median progression-free survival in the trametinib group was 13·0 months (95% CI 9·9–15·0) compared with 7·2 months (5·6–9·9) in the standard-of-care group (hazard ratio 0·48 [95% CI 0·36–0·64]; p<0·0001). The most frequent grade 3 or 4 adverse events in the trametinib group were skin rash (17 [13%] of 128), anaemia (16 [13%]), hypertension (15 [12%]), diarrhoea (13 [10%]), nausea (12 [9%]), and fatigue (ten [8%]). The most frequent grade 3 or 4 adverse events in the standard-of-care group were abdominal pain (22 [17%]), nausea (14 [11%]), anaemia (12 [10%]), and vomiting (ten [8%]). There were no treatment-related deaths.

**Interpretation:**

Trametinib represents a new standard-of-care option for patients with recurrent low-grade serous carcinoma.

**Funding:**

NRG Oncology, Cancer Research UK, Target Ovarian Cancer, and Novartis.

## Introduction

Epithelial ovarian cancers comprise five different histological subtypes (high-grade serous, endometrioid, clear-cell, mucinous, and low-grade serous).^1^, ^2^ These subtypes vary in terms of the tissue of origin, molecular biology, stage of presentation, chemosensitivity, and patient outcome.^3^, ^4^ Low-grade serous carcinoma, which accounts for approximately 5% of all cases of epithelial ovarian cancer, is distinguished from high-grade serous carcinoma primarily based on its degree of nuclear atypia (ie, mild to moderate *vs* marked), with mitotic index (ie, ≥12 mitoses *vs* >12 mitoses per ten high-power fields) as a secondary feature.^2^, ^5^, ^6^ From a molecular perspective, low-grade and high-grade serous carcinomas have distinct developmental pathways. Low-grade serous carcinoma (and the serous borderline tumours from which they can arise) have a high frequency of activating mutations in the MAPK pathway and generally express wild-type *TP53*.^7^, ^8^, ^9^, ^10^ By contrast, high-grade serous carcinomas have almost ubiquitous *TP53* mutations, defects in DNA repair capability, and gross gene copy number abnormalities.^11^, ^12^

**Research in context**

**Evidence before this study**

Low-grade serous ovarian cancer is a rare and under-studied form of ovarian cancer that has only become prominent as a discrete entity since 2004. We searched PubMed using the terms “low-grade serous” and “ovarian cancer”. We searched for clinical trials published in English between Jan 1, 2004, and Dec 31, 2020. Only two randomised controlled trials of this disease have been published, both in 2020. One trial compared the MEK inhibitor binimetinib to standard-of-care chemotherapy without showing a significant benefit, and the other trial compared the MEK inhibitor pimasertib to pimasertib plus the PI3K inhibitor SAR245409 without showing additional benefit for the combination group. Nonetheless, these trials and other previous single-arm studies suggested that MEK inhibition might be a useful strategy with some degree of efficacy.

**Added value of this study**

This is the first positive randomised clinical trial of a MEK inhibitor reported in low-grade serous ovarian cancer. Our study shows that the use of the oral MEK inhibitor trametinib significantly increased progression-free survival and objective response rate compared with standard-of-care therapies in patients with relapsed or persistent low-grade serous ovarian cancer. These outcomes were achieved without a clinically significant effect of the therapy on quality of life.

**Implications of all the available evidence**

Trametinib represents a new standard-of-care therapy option in the treatment of relapsed or persistent low-grade serous ovarian cancer.

In the largest unselected cohort of low-grade serous carcinomas sequenced to date, the frequency of *KRAS* mutations was 27%*, BRAF* mutations was 13%, and *NRAS* mutations was 9%.^10^ In the same cohort, the frequency of oestrogen receptor expression was 97% and progesterone receptor expression was 68%.

Low-grade serous carcinoma is characterised by a younger age at diagnosis, relative resistance to platinum-based chemotherapy, and extended overall survival compared with high-grade serous carcinoma.^13^, ^14^ However, similar to high-grade serous carcinoma, most patients with low-grade disease are diagnosed in the advanced stages, and over 70% of patients relapse.^15^ As in the primary treatment setting, response rates to salvage chemotherapy are low.^16^ Thus, a continued search for novel, more effective therapies is a priority for this rare type of ovarian cancer, which is in-keeping with the potential importance of this trial.

Retrospective series have suggested activity of aromatase inhibition and antiangiogenic therapy in low-grade serous carcinoma.^17^, ^18^, ^19^, ^20^, ^21^ However, no positive randomised study of any therapy in low-grade serous carcinoma has been reported to date. A single-arm, phase 2 study of the MEK inhibitor selumetinib showed an objective response rate (ORR) of 15% (95% CI 7·9–26·1).^22^ The MEK Inhibitor in Low-Grade Serous Ovarian Cancer (MILO)/ENGOT-ov11 study compared the MEK inhibitor binimetinib to investigator's choice chemotherapy.^23^ This study was stopped early due to crossing of the prespecified futility boundary. Although the study did not meet its progression-free survival endpoint in terms of superiority to chemotherapy, binimetinib efficacy was shown across several endpoints. In addition, a post-hoc analysis suggested a possible association between the presence of mutant *KRAS* and binimetinib efficacy.

Trametinib is a selective, reversible, allosteric inhibitor of MEK1 and MEK2. This drug is currently approved for use in combination with dabrafenib for unresectable or metastatic *BRAF*^V600E^ (ie, Val600Glu) or *BRAF*^V600K^ (ie, Val600Lys) mutation-positive melanoma, metastatic *BRAF*^V600E^ mutation-positive non-small-cell lung cancers, and metastatic *BRAF*^V600E^ mutation-positive anaplastic thyroid cancer. The aim of our study was to evaluate the efficacy and safety of the MEK inhibitor, trametinib, at its licensed dose in other malignancies, compared with physician's choice standard of care in women with recurrent low-grade serous carcinoma.

## Methods

### Study design

This international, randomised, open-label, multicentre, phase 2/3 trial was done at 72 hospitals in the USA and 12 hospitals in the UK. The study was approved by the institutional review boards or the Central Institutional Review Board of the National Cancer Institute (USA) and by the East of Scotland Research Ethics Service Research Ethics Committee 2 (UK). The trial was sponsored by NRG Oncology (Buffalo, NY, USA) and the University of Glasgow and National Health Service Greater Glasgow and Clyde Health Board (Glasgow, UK). This trial was done in accordance with the Declaration of Helsinki and Good Clinical Practice guidelines.

### Patients

Eligible patients were aged 18 years or older with recurrent low-grade serous carcinoma following initial diagnosis of ovarian or peritoneal low-grade serous carcinoma or serous borderline tumour. Histology was confirmed by prospective expert pathology review. The pathology review included digital tissue review by a panel of three pathologists. In addition, separate panels of three pathologists in the USA and UK reviewed the pathology. Confirmation of eligibility required agreement by at least two pathologists on the diagnosis of recurrent low-grade serous carcinoma. Specifically, patients with serous borderline tumours or tumours containing low-grade and high-grade serous carcinomas were excluded. Patients were eligible if they had previously received at least one platinum-based chemotherapy regimen, but not all five standard-of-care options. Receipt of an unlimited number of previous therapy regimens, including chemotherapy or hormonal therapy, was allowed. Measurable disease, as defined by Response Evaluation Criteria In Solid Tumors (RECIST) version 1.1, was required.^24^ Further details and a complete list of eligibility criteria are provided in the study protocol (appendix pp 37–41). All patients provided written informed consent.

### Randomisation and masking

Patients were randomly assigned (1:1) to receive either trametinib or one of five standard-of-care options. Randomisation was stratified using minimisation to balance treatment assignment by geographical region (USA or UK), number of previous regimens (1, 2, or ≥3), performance status (0 or 1), and planned standard-of-care regimen (if in the standard-of-care group). All patients were randomly assigned and registered centrally via the National Cancer Institute Oncology Patient Enrollment Network. The study was open-label, with both patients and investigators aware of treatment assignment.

### Procedures

Patients in the trametinib group received oral trametinib 2 mg once daily. Patients in the standard-of-care group received one of five physician's choice (selected before randomisation) standard-of-care options: paclitaxel 80 mg/m^2^ by body surface area, administered via intravenous infusion over 1 h, on days 1, 8, and 15 of every 28-day cycle; pegylated liposomal doxorubicin 40–50 mg/m^2^ by body surface area, administered via intravenous infusion over 1 h, on day 1 every 28 days; topotecan 4 mg/m^2^ by body surface area, administered via intravenous infusion over 30 min, on days 1, 8, and 15 of every 28-day cycle; oral letrozole 2·5 mg once daily; or oral tamoxifen 20 mg twice daily. Treatment continued until either unacceptable toxicity or disease progression (defined as a ≥20% increase in the sum of the diameters of target lesions, as per RECIST version 1.1 criteria). Patients in the standard-of-care group were allowed to discontinue therapy after six cycles at the investigator's discretion. For the standard-of-care regimens, dose adjustments were made according to standard of care at the investigator's discretion (appendix pp 60–77). After disease progression, patients in the standard-of-care group could cross over to receive trametinib. The trametinib regimen allowed two dose reductions (to 1·5 mg or 1 mg) for haematological or other adverse events. Details of the criteria for dose modifications are noted in the appendix (pp 60–77).

Efficacy, measured by contrast CT or MRI lesion assessment, was planned to be assessed at baseline, once every 8 weeks for the first 15 months, and then once every 3 months thereafter. Details of additional assessments are included in the appendix (p 2). Safety assessments were done at baseline and then before each treatment cycle while on treatment. Following treatment, safety assessments were completed once every 3 months for 2 years, followed by once every 6 months for 3 years, and then annually.

Quality of life was assessed by use of the Functional Assessment of Cancer Therapy-Ovarian Cancer Trial Outcome Index (FACT-O TOI) and the adapted self-administered Functional Assessment of Cancer Therapy Gynecologic Oncology Group-Neurotoxicity questionnaire (FACT-GOG-Ntx) subscale.^25^, ^26^ Assessments were done before cycle 1, before cycle 4 (week 12), 4 weeks after cycle 6 (week 24), and at weeks 36 and 52 after starting the study therapy. A five-point difference between the trametinib group and standard-of-care group was considered the minimal clinically important difference. Additional details on quality-of-life assessments are provided in the appendix (p 2).

For the biomarker analysis, archival formalin-fixed paraffin-embedded (FFPE) specimens were retrieved and underwent formal pathology review to confirm the presence of low-grade serous carcinoma with greater than 40% tumour cellularity within an area of interest marked up by an expert gynaecological pathologist. This area was macrodissected and DNA isolation was performed. Whole exome sequencing of FFPE DNA was performed by Genuity Science (Dublin, Ireland) with the Agilent SureSelect Human All Exon V6 Exome Capture Kit (Agilent, Santa Clara, CA, USA) and Illumina NovaSeq 6000 (San Diego, CA, USA). Sequence reads were aligned to the reference human genome (GrCh38/hg38 in the University of California Santa Cruz Genome Browser) with Burrows-Wheeler Aligner software, version 0.7.17 (full details of this analysis are provided in the appendix p 3). Variant calling was performed by use of a majority vote system with three variant caller algorithms: VarDict, Mutect2, and Freebayes. Filtering for FFPE and oxidation artifacts was applied using the German Cancer Research Center (known as DKFZ) Bias Filter. Variants within *KRAS, NRAS,* and *BRAF* were extracted and filtered to remove variants at low allele frequency (<0·1), low sequencing depth at the locus (<20 times), or low number of variant-containing reads (<8 reads). Pathogenic mutations were flagged using ClinVar; any remaining benign changes and variants of unknown significance were flagged as probably non-functional (appendix p 3).

### Outcomes

The primary endpoint was investigator-assessed progression-free survival (defined as the time from randomisation to disease progression or death). Secondary endpoints were adverse events, objective tumour response rate (ORR; defined as the proportion of patients in each group with a clinical response), quality of life, the predictive and prognostic effect of MAPK pathway activation on response or progression-free survival, the prognostic effect of phosphorylated ERK expression, and overall survival. All secondary endpoints are included in this report except for the prognostic effect of phosphorylated ERK expression, which will be examined in a subsequent publication of additional translational aspects of the trial. Exploratory endpoints included progression-free survival and ORR after crossover. Disease progression and tumour response were assessed by radiological and clinical review according to RECIST version 1.1 criteria. A complete response was defined as the disappearance of all target lesions, and a partial response was defined as a 30% or greater decrease in the sum of the diameters of the target lesions. Stable disease was defined as neither sufficient tumour shrinkage to qualify for partial response nor sufficient increase to qualify for progressive disease. Adverse events, including adverse events of special interest, were described using the maximum grade (1–5) for affected patients, according to Common Terminology Criteria for Adverse Events (version 4) preferred terms and system organ class classifications. The main quality-of-life objective focused on the assessment timepoints from baseline up to the post-cycle 6 visit (at 24 weeks), though responses were collected up to week 52.

### Statistical analysis

As designed, the phase 3 trial had 80% power to detect a 50% or greater improvement in progression-free survival in the trametinib group compared with the standard-of-care group (from 8 to 12 months; hazard ratio [HR] 0·67), based on a one-sided 0·025 level log-rank test. The design targeted 213 progression-free survival events among 250 patients at the final analysis. An interim futility analysis was planned when 106 progression-free survival events were observed. A Rho-family spending function determined the early stopping rule. More details of the interim futility analysis are available in the appendix (p 2). The progression-free survival and ORR endpoints were assessed in the intention-to-treat population, and safety was assessed in all patients who had received at least one dose of study treatment. The mutation analysis was done in all patients with evaluable tissue samples. All patients who completed the baseline assessment and at least one follow-up assessment were evaluable for quality-of-life analysis.

All analyses in the study report were planned in the protocol, except for the following post-hoc analyses: proportional hazards assumption, per-protocol analysis, number-needed-to-treat analysis, treatment-free interval until disease progression in the patients who received chemotherapy in the standard-of-care group, and Kaplan-Meier plots for progression-free survival of trametinib versus letrozole, paclitaxel, pegylated liposomal doxorubicin, topotecan, and tamoxifen. For the quality-of-life assessment, the planned analysis was restricted to data up to 24 weeks; analysis of timepoints beyond 24 weeks was exploratory. Further details on the statistical analysis related to efficacy and quality of life are provided in the appendix (pp 2–3).

Sequence analysis and correlation of *KRAS*, *BRAF* and *NRAS* mutation status with outcome were done according to the final specification of statistical analyses to be undertaken relating clinical study data to translational biomarker data for the primary clinical publication (appendix pp 167–68). For the dichotomous biomarker analysis, patients were categorised on the basis of the presence or absence of activating mutations in any of *KRAS*, *BRAF,* or *NRAS*. The predictive and prognostic effects of the biomarker were assessed with respect to both progression-free survival and ORR clinical outcomes. Multiplicity adjustments using Hommel's method were made separately within the prognostic and predictive analysis to allow for two endpoints to be examined. The multivariable prognostic and predictive models included covariate adjustment for the stratification factors. Formal assessment of prognostic effect was restricted to patients in the standard-of-care group; corresponding results for the trametinib group were considered exploratory. Further details on the biomarker analysis are provided in the appendix (p 3).

All statistical analyses were done using SAS, version 9.4. p values are two-sided unless otherwise stated. The results of the interim futility analysis were evaluated by the NRG Oncology data monitoring committee, who recommended that the trial could proceed to phase 3. No new safety signals were observed during this review. The data monitoring committee reviewed accumulating summaries of toxicities and all serious adverse event reports on an ongoing basis. The frequency and severity of all toxicities were tabulated from submitted case-report forms and summarised for review by the study chair and the data monitoring committee semi-annually. Standardised toxicity reports were also submitted to the drug and disease monitors at the Investigational Drug Branch and Clinical Investigation Branch of the National Cancer Institute.

This trial is registered with ClinicalTrials.gov, NCT02101788, and is active but not recruiting.

### Role of the funding source

The funders of the study had a role in study design, data collection, data analysis, and data interpretation, but did not have a role in the writing of the report.

## Results

**Figure 1:**
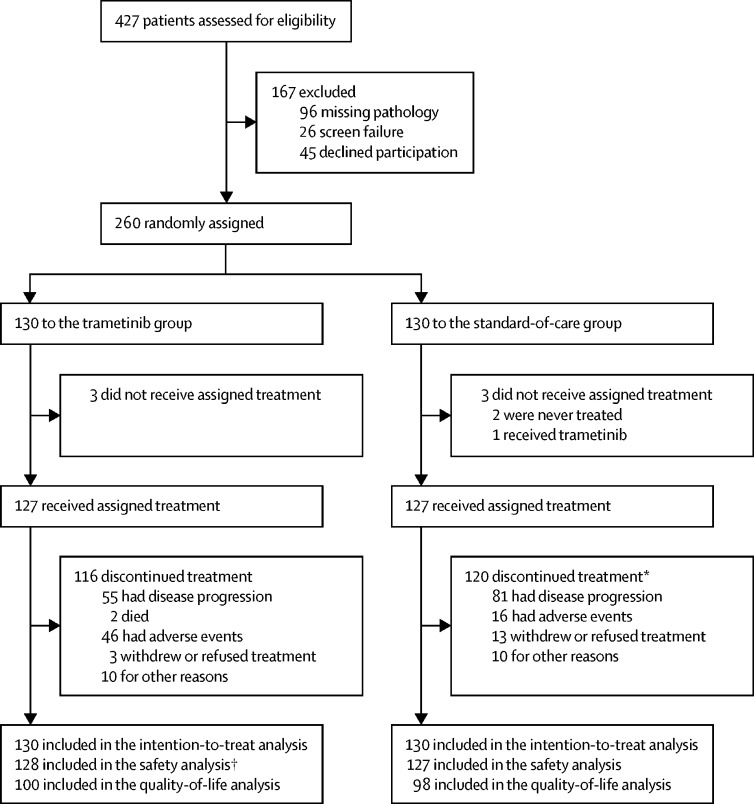
Trial profile *88 patients received trametinib following disease progression. †One patient randomly assigned to the standard-of-care group received trametinib instead and was included in the safety analysis for the trametinib group.

Between Feb 27, 2014, and April 10, 2018, 427 patients were assessed for eligibility and 260 eligible patients were enrolled and randomly assigned to the trametinib group (n=130) or the standard-of-care group (n=130; figure 1). The characteristics of patients at baseline and details of previous treatments are included in table 1. The median duration of follow-up was 31·3 months (IQR 15·7–41·9) in the standard-of-care group and 31·5 months (18·1–43·3) in the trametinib group. At data cutoff (July 16, 2019), 229 (88%) of 260 patients had discontinued their assigned treatment due to disease progression, toxicity, patient choice, other disease, or death.

**Table 1:** Characteristics of patients at baseline

		**Standard-of-care group (n=130)**	**Trametinib group (n=130)**
Age, years		55·3 (42·4–65·6)	56·6 (44·6–63·3)
Country			
	USA	102 (79%)	103 (79%)
	UK	28 (21%)	27 (21%)
Race			
	White	114 (88%)	115 (89%)
	Black or African American	5 (4%)	4 (3%)
	Asian	4 (3%)	3 (2%)
	Native Hawaiian or Pacific Islander	0 (0%)	1 (1%)
	Patient refused to specify or unknown	7 (5%)	7 (5%)
Ethnicity			
	Hispanic	7 (5%)	8 (6%)
	Non-Hispanic	118 (91%)	118 (91%)
	Refused to specify	5 (4%)	4 (3%)
Disease site			
	Ovary	117 (90%)	119 (92%)
	Peritoneum	13 (10%)	11 (8%)
Stage			
	I	8 (6%)	11 (8%)
	II	11 (8%)	11 (8%)
	III	93 (72%)	96 (74%)
	IV	18 (14%)	12 (9%)
Mutational status*			
	*KRAS, BRAF*, or *NRAS* mutation	22 (17%)	22 (17%)
	*KRAS* mutation	14 (11%)	16 (12%)
	*BRAF* mutation	1 (1%)	2 (2%)
	*NRAS* mutation	7 (5%)	4 (3%)
	No mutation	42 (32%)	48 (37%)
	Missing (no tissue)	66 (51%)	60 (46%)
Performance status			
	0	93 (72%)	93 (72%)
	1	37 (28%)	37 (28%)
Previous lines of systemic therapy†			
	1	30 (23%)	29 (22%)
	2	37 (28%)	39 (30%)
	≥3	63 (49%)	62 (48%)
	Mean number	2·9 (1·7)	2·9 (1·9)
	Range	1–10	1–10
Previous lines of chemotherapy‡			
	1	55 (42%)	62 (48%)
	2	39 (30%)	32 (25%)
	≥3	36 (28%)	36 (28%)
Previous lines of hormonal therapy‡			
	0	56 (43%)	54 (42%)
	1	68 (52%)	76 (58%)
	2	6 (5%)	0 (0%)
Planned treatment§			
	Letrozole	44 (34%)	43 (33%)
	Tamoxifen	27 (21%)	27 (21%)
	Paclitaxel	11 (8%)	14 (11%)
	Pegylated liposomal doxorubicin	40 (31%)	37 (28%)
	Topotecan	8 (6%)	9 (7%)
Current status¶			
	Alive	69 (53%)	78 (60%)
	Dead	61 (47%)	52 (40%)

Data are median (IQR), n (%), or mean (SD), unless otherwise indicated.

* No patient had more than one type of mutation.

† As reported by study site for stratification before randomisation; includes both chemotherapy and hormonal therapy.

‡ As reported on study case report form.

§ Of the 44 patients who received letrozole and 27 patients who received tamoxifen in the standard-of-care group, only one patient received previous hormonal therapy; they received letrozole and tamoxifen sequentially and then received tamoxifen on the standard-of-care group.

¶ As of July 16, 2019.

**Figure 2:**
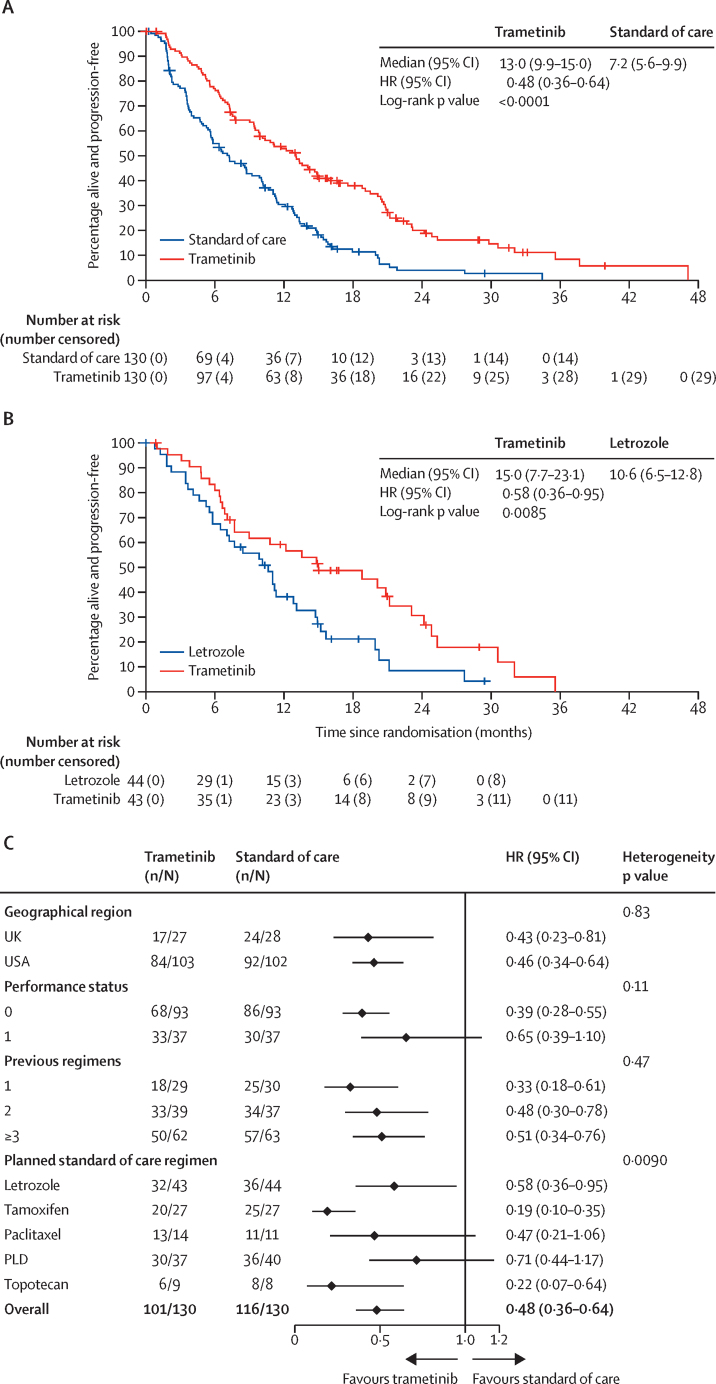
Assessment of investigator-assessed progression-free survival (A) Kaplan-Meier estimates of progression-free survival in the intention-to-treat population. (B) Kaplan-Meier estimates of progression-free survival for trametinib versus letrozole among 87 patients prespecified to receive letrozole if randomly assigned to the standard-of-care group. (C) Forest plot of multivariable-adjusted treatment hazard ratio (95% CI) estimates across the levels of each stratification factor. Smaller heterogeneity p values indicate a stronger departure from the null hypothesis of equal treatment effects across the different stratification factor levels. p values are not adjusted for multiple testing, and p values in (A) and (B) are one-sided. HR=hazard ratio. n=number of events. N=total number of patients. PLD=pegylated liposomal doxorubicin.

The primary analysis was done after 217 progression-free survival events (in 101 [78%] of 130 patients in the trametinib group and in 116 [89%] of 130 in the standard-of-care group), and favoured the trametinib group. Median progression-free survival was 13·0 months (95% CI 9·9–15·0) in the trametinib group compared with 7·2 months (5·6–9·9) in the standard-of-care group (HR 0·48 [95% CI 0·36–0·64]; one-sided p<0·0001; figure 2A). No violation of the proportional hazards assumption was found (p=0·68). An exploratory analysis of the number needed to treat is shown in the appendix (p 5).

In a post-hoc analysis of 87 patients preplanned to receive letrozole if randomly assigned to the standard-of-care group, those randomly assigned to the trametinib group had longer progression-free survival (median 15·0 months [95% CI 7·7–23·1]) than those given letrozole (median 10·6 months [6·5–12·8]; HR 0·58 [95% CI 0·36–0·95]; one-sided p=0·0085; figure 2B). The results of post-hoc analyses comparing progression-free survival in patients who received trametinib with those who received the other four standard-of-care therapies (pegylated liposomal doxorubicin, paclitaxel, topotecan, and tamoxifen) are shown in the appendix (pp 8–11).

The forest plot of treatment effects in terms of progression-free survival according to each stratification factor shows that all HRs favour trametinib, with none less than 0·71 (figure 2C). Significant heterogeneity (p=0·009; p=0·036 after Bonferroni adjustment) was found in the preplanned standard-of-care treatment factor. The heterogeneity relates to the marked treatment effect of trametinib in patients preplanned to receive tamoxifen. Excluding this subgroup, no significant heterogeneity was observed (p=0·21), and the significant estimated effect of trametinib remained in the overall analysis (HR 0·56 [95% CI 0·41–0·77]; one-sided p=0·0004).

The ORR of trametinib was 26% (34 of 130 patients), with a further 59% (77 of 130) having stable disease for a minimum of 8 weeks. This ORR compared favourably with both the standard-of-care group as a whole (ORR 6% [eight of 130]; odds ratio 5·4 [95% CI 2·4–12·2], p<0·0001; stable disease 71% [92 of 130]), and with the individual therapies (ORR 14% [six of 44] for letrozole, 9% [one of 11] for paclitaxel, 3% [one of 40] for pegylated liposomal doxorubicin, 0% [none of 27] for tamoxifen, and 0% [none of eight] for topotecan). Details regarding clinical response (complete, partial, stable disease, or disease progression) are provided in the appendix (p 6). The median duration of response was 13·6 months (IQR 7·2–19·9; 95% CI 8·1–18·8) for trametinib versus 5·9 months (4·0–12·2; 2·8–12·2) for standard of care.

At data cutoff, the overall survival analysis included 260 patients, of whom 111 (43%) had died (51 [39%] of 130 in the trametinib group and 60 [46%] of 130 in the standard-of-care group). Median overall survival was 37·6 months (95% CI 32·0–non-evaluable) in the trametinib group and 29·2 months (23·5–51·6) in the standard-of-care group. The HR for death was 0·76 (95% CI 0·51–1·12; one-sided p value 0·056) in favour of the trametinib group (appendix p 12). This overall survival analysis includes the effect of 88 (68%) of 130 patients in the standard-of-care group who crossed over to trametinib following disease progression.

Ten (18%) of 57 patients who received paclitaxel, pegylated liposomal doxorubicin, or topotecan in the standard-of-care group (two patients randomly assigned to this group did not receive treatment) stopped treatment after six cycles, as allowed per protocol. In these patients, the median treatment-free interval until disease progression was 4·0 months (IQR 0·9–6·8). The median treatment-free interval in all 57 patients who received chemotherapy in the standard-of-care group was 1·0 month (0·8–3·4). Details of the relative dose intensity of the six drugs included in the study is shown in the appendix (p 7).

Median progression-free survival in patients in the standard-of-care group who crossed over to trametinib following disease progression was 10·8 months (95% CI 7·3–12·0), and the ORR was 15% (95% CI 7·0–22·0; 13 of 88 patients). Of the 66 standard-of-care patients who progressed or died after crossing over to trametinib, 43 (65%) had a longer time to disease progression on trametinib than they had on their preceding standard-of-care therapy.

The occurrence of treatment-emergent adverse events reported in 20% or more of treated patients in both groups is shown in table 2. The patient randomly assigned to the standard-of-care group who received trametinib is included in the trametinib safety profile. The most frequent grade 3 or 4 adverse events in the trametinib group were acneiform or maculo-papular skin rash (17 [13%] of 128 patients), anaemia (16 [13%]), hypertension (15 [12%]), diarrhoea (13 [10%]), nausea (12 [9%]), and fatigue (ten [8%]). In the 127 standard-of-care group patients, the most frequent grade 3 or 4 adverse events were abdominal pain (22 [17%]), nausea (14 [11%]), anaemia (12 [10%]), and vomiting (ten [8%]). Ten (8%) patients in the trametinb group had a decrease in ejection fraction (eight grade 2 events and two grade 3 events). The frequency of other adverse events of special interest in the trametinib group was pneumonitis (in three [2%]; one each grade 1, 2, and 3), QTc prolongation (two [2%]; one grade 1 and one grade 4), left ventricular systolic dysfunction (two [2%]; both grade 3), retinal vascular disorder (two [2%]; one grade 2 and one grade 3), and retinal tear (one [1%]; grade 3). Of the 20 patients with these rare but clinically significant adverse events, three (15%) patients who had a decrease in ejection fraction and one (5%) patient who had QTc prolongation were able to resume trametinib treatment. No grade 5 adverse events reported were definitely attributable to trametinib. In the standard-of-care group, the following adverse events of special interest were observed: one (1%) patient had grade 3 left ventricular systolic dysfunction, and one (1%) patient had grade 3 decreased ejection fraction.

**Table 2:** Treatment-emergent adverse events in the safety analysis population

	**Trametinib group (n=128)**				**Standard-of-care group (n=127)**			
	Any grade	Grade 1	Grade 2	Grade >3	Any grade	Grade 1	Grade 2	Grade >3
**General disorders**								
Fatigue	93 (73%)	47 (37%)	36 (28%)	10 (8%)	74 (58%)	44 (35%)	25 (20%)	5 (4%)
Peripheral oedema	62 (49%)	44 (34%)	18 (14%)	0	15 (12%)	9 (7%)	5 (4%)	1 (1%)
**Gastrointestinal disorders**								
Abdominal pain	57 (45%)	37 (29%)	13 (10%)	7 (6%)	60 (47%)	27 (21%)	11 (9%)	22 (17%)
Constipation	54 (42%)	43 (34%)	8 (6%)	3 (2%)	49 (39%)	38 (30%)	8 (6%)	3 (2%)
Diarrhoea	93 (73%)	57 (45%)	23 (18%)	13 (10%)	43 (34%)	29 (23%)	10 (8%)	4 (3%)
Oral mucositis	45 (35%)	34 (27%)	8 (6%)	3 (2%)	23 (18%)	13 (10%)	8 (6%)	2 (2%)
Nausea	78 (61%)	43 (34%)	23 (18%)	12 (9%)	65 (51%)	39 (31%)	12 (9%)	14 (11%)
Vomiting	59 (46%)	40 (31%)	10 (8%)	10 (7%)	44 (35%)	24 (19%)	10 (8%)	10 (8%)
**Skin and subcutaneous tissue disorders**								
Dry skin	56 (44%)	46 (36%)	9 (7%)	1 (1%)	17 (13%)	16 (13%)	1 (1%)	0
Acneiform rash	81 (63%)	52 (41%)	21 (16%)	8 (6%)	13 (10%)	10 (8%)	2 (2%)	1 (1%)
Maculopapular rash	54 (42%)	30 (23%)	15 (12%)	9 (7%)	28 (22%)	21 (17%)	7 (6%)	0
**Blood and lymphatic system disorders**								
Anaemia	67 (52%)	30 (23%)	21 (16%)	16 (13%)	54 (43%)	23 (18%)	19 (15%)	12 (10%)
White blood cell count decreased	28 (22%)	19 (15%)	8 (6%)	1 (1%)	21 (17%)	13 (10%)	5 (4%)	3 (2%)
**Injury, poisoning, and procedural complications**								
Alkaline phosphatase increased	32 (25%)	29 (23%)	1 (1%)	2 (2%)	11 (9%)	11 (9%)	0	0
Aspartate aminotransferase increased	47 (37%)	43 (34%)	3 (2%)	1 (1%)	15 (12%)	13 (10%)	1 (1%)	1 (1%)
Alanine aminotransferase increased	28 (22%)	24 (19%)	2 (2%)	2 (2%)	13 (10%)	11 (9%)	2 (2%)	0
Creatinine increased	26 (20%)	21 (16%)	4 (3%)	1 (1%)	10 (8%)	7 (6%)	3 (2%)	0
**Metabolism and nutrition disorders**								
Anorexia	34 (27%)	22 (17%)	10 (8%)	2 (2%)	24 (19%)	15 (12%)	8 (6%)	1 (1%)
Hyperglycaemia	32 (25%)	26 (20%)	6 (5%)	0	25 (20%)	20 (16%)	3 (2%)	2 (2%)
Hypokalaemia	26 (20%)	21 (16%)	0	5 (4%)	16 (13%)	11 (9%)	2 (2%)	3 (2%)
Hypomagnesemia	41 (32%)	34 (27%)	6 (5%)	1 (1%)	29 (23%)	27 (21%)	2 (2%)	0
Hypoalbuminemia	43 (34%)	19 (15%)	20 (16%)	4 (3%)	16 (13%)	8 (6%)	7 (6%)	1 (1%)
**Nervous system disorders**								
Headache	27 (21%)	22 (17%)	5 (4%)	0	24 (19%)	19 (15%)	4 (3%)	1 (1%)
Peripheral sensory neuropathy	36 (28%)	31 (24%)	4 (3%)	1 (1%)	28 (22%)	23 (18%)	4 (3%)	1 (1%)
**Vascular disorders**								
Hypertension	50 (39%)	7 (6%)	28 (22%)	15 (12%)	27 (21%)	8 (6%)	13 (10%)	6 (5%)
**Respiratory, thoracic, and mediastinal disorders**								
Dyspnoea	45 (35%)	31 (24%)	10 (8%)	4 (3%)	28 (22%)	20 (16%)	5 (4%)	3 (2%)
**Infections and infestations**								
Urinary tract infection	29 (23%)	0	20 (16%)	9 (7%)	18 (14%)	0	12 (9%)	6 (5%)

Data are n (%). Adverse events occurring in more than 20% of patients according to system organ class are shown.

The 128 patients given trametinib completed a total of 1365 cycles. The median number of cycles received was eight (IQR 3–16). Dose reductions occurred in 156 (11%) of all trametinib cycles. 90 (70%) patients required at least one dose reduction during the study period. 38 (30%) patients required two dose reductions; of these patients, 14 withdrew due to disease progression, 17 due to adverse events, and two for other reasons. Five (4%) patients were still on treatment at the data cutoff date. The median number of trametinib cycles after the second dose reduction was six (IQR 1–12). During cycle 1, nine (7%) patients required a dose modification due to adverse events, but by cycle 2, 46 (39%) of 117 patients required dose modification due to adverse events, after which point the proportion of patients requiring further dose modifications gradually decreased (data not shown). A total of 46 (36%) of 128 patients discontinued trametinib due to toxicity compared with 38 (30%) of 127 patients who discontinued standard-of-care therapy due to toxicity.

Overall, grade 3 or higher gastrointestinal disorders occurred in 72 (28%) of 255 patients; in 37 (29%) of 128 patients in the trametinib group and in 35 (28%) of 127 patients in the standard-of-care group. Small intestine obstruction occurred in nine (7%) patients in the standard-of-care group and in 16 (13%) patients in the trametinib group, and colon obstruction occurred in six (5%) patients in the standard-of-care group and in one (1%) patient in the trametinib group.

The compliance rates of quality-of-life assessments in patients were 88% (227 of 259 patients) at baseline and 77% (194 of 253) at 12 weeks, 63% (153 of 244) at 24 weeks, 60% (139 of 233) at 36 weeks, and 56% (125 of 222) at 52 weeks after cycle 1. No significant difference in quality-of-life assessment compliance rates between the two groups was observed (p=0·57). A total of 198 evaluable patients (98 in the standard-of-care group and 100 in the trametinib group) who completed the baseline assessment and at least one follow-up assessment were evaluable for quality-of-life analysis. The patient-reported FACT-O TOI scores are presented in the appendix (p 13). After adjusting for baseline quality-of-life score, age, and stratification factors, the interaction between assessment times and treatment group on the FACT-O TOI was significant (p=0·0013). Although less than the minimal clinically important difference, patients in the trametinib group reported a worse quality of life by 3·6 points (95% CI –6·8 to –0·5; adjusted p=0·048) at 12 weeks compared with the standard-of-care group. We found no significant differences in quality of life between the two groups at other timepoints, including in an exploratory examination of differences at weeks 36 and 52. We also found no patient-reported neurotoxicity differences between the two groups using the FACT-GOG-Ntx subscale. Exploratory subset quality-of-life analyses of comparisons within the chemotherapy and hormonal therapy standard-of-care treatment strata suggest that the difference at week 12 was mainly due to the hormonal strata (appendix pp 3–4).

Tumour samples were available for 189 patients; 150 samples were confirmed to contain low-grade serous carcinoma with a minimum cellularity of 40% on pathology review and underwent DNA extraction. Five samples failed DNA quality control (ie, they contained <200 ng DNA). 11 samples failed quality control for excessive FFPE damage (n=10) or low sequencing coverage (ie, <50 times the mean target coverage; n=1). The median per-sample on-target coverage for successfully sequenced samples was 102 times (range 59–172).

Of the 134 patients for whom sequence data was obtained, 44 (33%) had tumours harbouring activating mutations in *KRAS*, *BRAF,* or *NRAS*. Mutations were detected in 22 (31%) of 70 patients in the trametinib group and in 22 (34%) of 64 patients in the standard-of-care group. The observed treatment effect in terms of progression-free survival was in favour of trametinib in mutation-positive patients (HR 0·55 [95% CI 0·28–1·07]) and mutation-negative patients (0·64 [0·39–1·03]; table 3). There was no evidence that mutation status was predictive for progression-free survival (multiple comparison adjusted p=0·72, test for interaction). Overall, ORRs were markedly more favourable for trametinib than standard-of-care therapy in mutation-positive patients (11 [50%] of 22 *vs* two [9%] of 22) than in mutation-negative patients (four [8%] of 48 *vs* three [7%] of 42), although this did not reach statistical significance (multiple comparison adjusted p=0·11, test for interaction).

**Table 3:** Predictive value of mutation status

		***KRAS, BRAF*, or *NRAS* mutation present**			***KRAS, BRAF*, and *NRAS* mutation absent**			**Nominal interaction p value**	Adjusted interaction p value*
		Standard-of- care group (n=22)	Trametinib group (n=22)	HR or OR (95% CI)	Standard-of-care group (n=42)	Trametinib group (n=48)	HR or OR (95% CI)		
Progression-free survival		..	..	..	..	..	..	0·72	0·72
	Number of events	20	20	..	38	37	..	..	..
	Median progression-free survival, months (95% CI)	11·4 (3·7–13·3)	13·2 (9·4–20·8)	HR 0·55 (0·28–1·07)	6·3 (3·7–9·9)	7·3 (5·6–12·7)	HR 0·64 (0·39–1·03)	..	..
Clinical response		..	..	..	..	..	..	0·054	0·11
	Number of participants with a complete or partial response	2	11	..	3	4	..	..	..
	Overall response rate (95% CI)	9·1% (1·9–26·1)	50·0% (30·2–69·8)	OR 10·17 (1·89–54·65)	7·1% (2·1–17·9)	8·3% (2·9–18·6)	OR 1·13 (0·26–4·79)	..	..

HR=hazard ratio. OR=odds ratio.

* Adjusted for multiple comparisons.

In the standard-of-care group, mutation status was not a significant prognostic factor for progression-free survival (HR 0·58 [95% CI 0·30–1·10]; multiple comparison adjusted p=0·19) or ORR (odds ratio 1·67 [95% CI 0·30–9·28]; multiple comparison adjusted p=0·39; table 4).

**Table 4:** Prognostic value of mutation status

		**Standard-of-care group**					**Trametinib group**		
		*KRAS, BRAF*, or *NRAS* mutation present (n=22)	*KRAS, BRAF,* and *NRAS* mutation absent (n=42)	HR or OR (95% CI)	Nominal prognostic p value	Adjusted prognostic p value*	*KRAS, BRAF*, or *NRAS* mutation present (n=22)	*KRAS, BRAF,* and *NRAS* mutation absent (n=48)	HR or OR (95% CI)
Progression-free survival		..	..	..	0·093	0·19	..	..	..
	Number of events	20	38	..	..	..	20	37	..
	Median progression-free survival, months (95% CI)	11·4 (3·7–13·3)	6·3 (3·7–9·9)	HR 0·58 (0·30–1·10)	..	..	13·2 (9·4–20·8)	7·3 (5·6–12·7)	HR 0·41 (0·21–0·80)
Clinical response		..	..	..	0·39	0·39	..	..	..
	Number of participants with a complete or partial response	2	3	..	..	..	11	4	..
	Overall response rate (95% CI)	9·1% (1·9–26·1)	7·1% (2·1–17·9)	OR 1·67 (0·30–9·28)	..	..	50·0% (30·2–69·8)	8·3% (2·9–18·6)	OR 15·07 (3·33–68·22)

HR=hazard ratio. OR=odds ratio.

* Adjusted for multiple comparisons. p values are provided for the standard-of-care group only because the prognostic value of mutational status was formally assessed in this group alone. Trametinib group statistics are provided for information only.

## Discussion

Until now, treatment recommendations for low-grade serous carcinoma were based on extrapolated results from historical randomised studies mainly involving patients with high-grade serous carcinoma, despite clearly discrete biology and clinical behaviour. Our trial is the first positive randomised trial of any therapy in low-grade serous carcinoma showing that the MEK inhibitor trametinib reduced the risk of disease progression or death by 52% compared with investigator's choice of endocrine therapy or chemotherapy. Trametinib was also associated with a four-fold increase in the probability of response to therapy, and showed a trend toward an overall survival benefit, despite the 68% of patients in the standard-of care group who crossed over to trametinib.

Therapeutic options for low-grade serous carcinoma have been scarce, in part because recruitment challenges have made randomised controlled trials in rare ovarian cancer subtypes infeasible.^27^ For this trial, accrual was optimised by expanding eligibility; almost half of patients had received three or more previous regimens, and crossover to receive trametinib was allowed. The investigator's choice standard-of-care group accommo-dated the scarcity of data (and genuine uncertainty) regarding optimal therapy, and minimised ineligibility due to patients having received multiple standard-of-care options. Potential weaknesses introduced by the standard-of-care group were mitigated by requiring the enrolling physician to specify the standard-of-care choice before randomisation. In the patient subgroup preplanned to receive letrozole if randomly assigned to the standard-of-care group, those randomly assigned to the trametinib group had superior progression-free survival compared with those assigned to receive standard-of-care letrozole, suggesting that this benefit was not attributable to inclusion of possibly ineffective standard-of-care therapies.

One potential weakness of this study was the bias of individual investigators to prematurely ascertain disease progression to allow their patient to crossover to trametinib. To control this, the protocol required objective evidence of RECIST criteria-defined progression before crossover was permitted. Despite patients crossing over, the intention-to-treat analysis of overall survival showed a trend, although not statistically significant, towards a marked benefit associated with trametinib, supporting the evidence for improvement in progression-free survival.

Possible explanations for why the positive outcome from this MEK inhibitor study contrasted to the negative result of the MILO/ENGOT-ov11 study^23^ (the other large randomised MEK inhibitor study in low-grade serous carcinoma) include differences in the number of previous treatment lines or allowed standard-of-care therapies, and in the effectiveness of MEK inhibition. More specifically, the control group in the MILO/ENGOT-ov11 study had a better-than-expected outcome (median progression-free survival 10·6 months [95% CI 9·2–14·5]). Similar to our trial, median progression-free survival in the control group of the MILO/ENGOT-ov11 study was estimated to be 7 months based on two previous retrospective studies.^16^, ^20^ The trial design aimed to detect a HR of 0·60 for progression-free survival in the binimetinib group versus the control group, corresponding to a median progression-free survival of 11·7 months in the binimetinib group. In the MILO/ENGOT-ov11 trial, eligibility was limited to three or fewer lines of previous chemotherapy regimens, with no limit on the number of lines of previous hormonal therapy. However, only 55 (27%) of 201 patients in the MILO/ENGOT-ov11 study received three or more previous systemic regimens of any type. By contrast, in the two studies used to estimate median progression-free survival in the control group of the MILO/ENGOT-ov11 study (the same two studies used for a similar estimate in our trial), the proportions of patients who received three or more previous systemic regimens were 62% (67 of 108; range 1–11)^16^ and 56% (50 of 89; 1–14).^20^ In our trial, 125 (48%) of 260 patients had received at least three previous systemic regimens and thus represented a more heavily pretreated cohort with a potentially poorer prognosis than the control group of the MILO/ENGOT-ov11 study. Compared with the MILO/ENGOT-ov11 trial, this cohort replicates more closely the patient populations on which the statistical analysis plan was based. Another difference between the MILO/ENGOT-ov11 trial and ours is that, although both trials included the same three chemotherapy drugs in the control group, our control group also included two endocrine drugs. In addition, preclinical studies suggest that trametinib is a more potent MEK inhibitor than selumetinib, binimetinib, and refametinib, but whether this increased potency translates into greater clinical efficacy is unknown.^28^

The toxic effects observed in patients in the trametinib group of our study were similar to previous MEK inhibitor studies in patients with other cancer types, such as melanoma. However, clinical management of adverse events can be challenging, with fatigue, skin rash, and gastrointestinal side-effects occurring most commonly, and dose reductions being necessary for many patients. For adverse events of special interest, the incidence of retinal events was 2%, and, although 8% of patients had decreased ejection fraction, they often recovered, and some patients were able to be rechallenged.

Given the increased toxicity associated with trametinib, an important secondary outcome of this study was quality of life. Although patients in the trametinib group reported significantly worse quality of life at 12 weeks than those in the standard-of-care group, this did not reach the five-point difference threshold for a clinically meaningful difference. The reduction in quality of life at week 12 is consistent with the higher frequency of adverse events reported in the trametinib group than in the standard-of-care group. Although, it is possible that the difference in quality of life apparent at 6 months could be due to the disease rather than toxicity, this notion is difficult to reliably discern.

The apparent progression-free survival benefit of trametinib regardless of *KRAS, BRAF,* or *NRAS* mutation status suggests that MAPK pathway activity is important, even in the absence of a canonical mutation. This benefit could be due to less common gene mutation events or to activation of the pathway at the epigenetic, transcriptional, or post-transcriptional levels. By comparison, the MILO/ENGOT-ov11 study reported improvements in progression-free survival and ORR in the *KRAS-*mutant group compared with the wild-type *KRAS* group of patients given binimetinib; however, the study did not directly address whether this mutation was predictive. Similarly, in patients given trametinib in our study, progression-free survival and ORR were both markedly higher in the patients with *KRAS, BRAF,* or *NRAS* mutations than in those with wild-type *KRAS, BRAF,* or *NRAS* (median progression-free survival 13·2 months [95% CI 9·4–20·8] *vs* 7·3 months [5·6–12·7]; ORR 50·0% [95% CI 30·2–69·8] *vs* 8·3% [2·9–18·6]). Additionally, we found that this mutation profile might be predictive of ORR (p=0·11). Nevertheless, although this emerging preliminary data is of interest, until more definitive information regarding the association between mutation status and treatment outcomes, as well as the optimal predictive biomarker panel becomes available, MEK inhibitors should be considered as an option for all women with recurrent low-grade serous carcinoma, regardless of mutation status. The rate of *KRAS*, *BRAF,* and *NRAS* mutations in our study population of relapsed disease patients (33%) is less than that found in series of unselected patients (around 50%),^10^ raising the possibility that patients with MAPK wild-type tumours could be selected for in this study. Evidence already exists showing that women whose tumours contain a *KRAS* or *BRAF* mutation have a better outcome than those whose tumours do not carry these MAPK pathway-activating mutations.^29^

The findings of our study raise the question of how to sequence chemotherapy, hormonal therapy, and trametinib in the treatment programme of a patient with low-grade serous carcinoma. In many institutions, first-line systemic therapy will consist of chemotherapy followed by aromatase inhibitor therapy. In such patients, the results of our study would strongly suggest that trametinib would be the most favourable option at first relapse. In patients who have not received a previous aromatase inhibitor, a discussion between the clinician and patient regarding the superior efficacy but less favourable toxicity profile of a MEK inhibitor at first relapse should guide decision-making. Preclinical data suggest that MEK inhibition could promote hormone sensitivity in ovarian cancer cells; therefore, trials combining trametinib and aromatase inhibitors could be warranted in the future.^30^, ^31^

In conclusion, based on the findings of our study, trametinib should be considered a new standard-of-care option for women with progressive or relapsed low-grade serous carcinoma.

## Data sharing

Data collected for the study, including individual participant data and a data dictionary defining each field in the set, will be made available to others on www.clinicaltrials.gov within 1 year of publication. Requests for deidentified patient-level data from studies funded through the National Cancer Institute Cancer Therapy Evaluation Program must comply with the US Department of Health and Human Services and Office for Human Research Protections policies and requirements. Requests for sharing of deidentified patient-level data should be sent to the corresponding author and will be considered on a case-by-case basis with the National Cancer Institute Cancer Therapy Evaluation Program.

For the **study protocol** see https://clinicaltrials.gov/ProvidedDocs/88/NCT02101788/Prot_SAP_000.pdf

## Declaration of interests

DMG reports payments made to his institution from the National Cancer Institute (NRG Oncology), Novartis, and the GOG Foundation for this study; royalties or licenses from Elsevier and UpToDate outside the submitted work; consulting fees from Genentech outside the submitted work; personal fees for service as a member of the Committee of the National Cancer Institute outside the submitted work; and stock or stock options from Johnson & Johnson, Bristol Myers Squibb, and Biogen outside the submitted work. AM reports service as a masked study statistician for VBL Therapeutics; provision of statistical institutional support for Advaxis; service as a data safety monitoring board reporting statistician for Regeneron; and service on a study steering committee for Genentech and AstraZeneca, all outside the submitted work. RLC reports consulting fees from AstraZeneca, Merck–GlaxoSmithKline, and Clovis; grants from Genmab, Genentech–Roche, Janssen, Agenus, Regeneron, and OncoQuest; payments or honoraria for lectures, presentations, speaker's bureaus, manuscript writing, or educational events from AstraZeneca, Merck–GlaxoSmithKline, and Clovis; and service on a data safety monitoring board or an advisory board for Janssen, VBL Therapeutics, and AstraZeneca, all outside the submitted work. KNM reports service on advisory boards for Alkemeres, AstraZeneca, Aravive, Blueprint Medicines, Eisai, Elevar, GlaxoSmithKline–Tesaro, Genentech–Roche, Hengrui, Immunogen, IMab, Merck, Mereo, Mersana, Myriad, OncXerna, Onconova, Novartis, Sorrento, Tarveda, and VBL Therapeutics, all outside the submitted work; grant funding from PTC Therapeutics, Lilly, Merck, and Genentech–Roche outside the submitted work; funding paid to her institution from Cyteir, Immunocore, Bolt Bio, Amgen, Artios, GlaxoSmithKline–Tesaro, Mereo, Regeneron, Aravive, Verastem, and AstraZeneca, for all of which she also serves as a local principal investigator; serving as national principal investigator for the IMaGYN050 trial of ovarian cancer (Genentech–Roche), the MIRASOL trial of ovarian cancer (Immunogen), the Relevare study of ovarian cancer (OncXerna), the FIRST study of ovarian cancer (GlaxoSmithKline–Tesaro), and the ETCTN 10422 study of ovarian cancer (National Cancer Institute); being the associate director for GOG Partners, on the GOG Foundation board of directors, and the NRG Oncology chair of the ovarian cancer committee; and receiving speaking reimbursement from PER, Research to Practice, Medscape, Great Debates and Updates, and Gyn Mal, all of which are outside the scope of this work but were active during the conduct of this study. SB reports grants paid to their institution from AstraZeneca, Tesaro, and GlaxoSmithKline; personal fees for participation in advisory boards from Amgen, AstraZeneca, Genmab, GlaxoSmithKline, Immunogen, Merck Sharp & Dohme, Merck Serono, Mersana, Oncxerna, Seagen, Shattuck Lab, and Epsilogen; and personal fees for lectures from Amgen, AstraZeneca, Clovis, GlaxoSmithKline, Immunogen, Merck Sharp & Dohme, Mersana, Pfizer, Roche, and Takeda, all outside the submitted work. AAS reports a National Cancer Trial Network grant; grants paid to her institution from AbbVie, Amgen, AstraZeneca, Boehringer Ingelheim, Clovis, Eisai, Immutep, Merck, OncoQuest, PharmaMar, Genentech–Roche, Seagen, Tesaro–GlaxoSmithKline, and VBL Therapeutics; consulting fees from Aravive, AstraZeneca, Clovis, Cordgenics, Eisai, Merck, Mersana, Myriad, Oncoquest, Genentech–Roche, and Tesaro–GlaxoSmithKline; presentations for educational events on cervical cancer sponsored by Research to Practice; service on advisory boards (uncompensated) for AbbVie and Regeneron; service on clinical trial steering committees (all uncompensated) for the AtTEnd trial (Hoffman-LaRoche), the Oval Trial (VBL Therapeutics), and the FLORA-5 trial (Oncoquest); and service on the GOG Foundation Board of Directors, the Society of Gynecologic Oncology Board of Directors, and the American Association of Obstetricians and Gynecologists Foundation Board of Trustees, all outside the submitted work. DMO reports National Cancer Institute support for this clinical trial; research funding paid to their institution from AstraZeneca, Tesaro–GlaxoSmithKline, Immunogen, Janssen–Johnson & Johnson, AbbVie Regeneron, Amgen, Novocure, Genentech–Roche, VentiRx Array Biopharma EMD Serono, Ergomed, Ajinomoto, Ludwig Cancer Research Stemcentrx, Cerulean Pharma, the GOG Foundation, the National Cancer Institute, Bristol Myers Squibb, C Serono, TRACON Pharmaceuticals, Yale University, New Mexico Cancer Care Alliance, inVentiv Health Clinical, Iovance Biotherapeutics, PRA Intl, Eisai, Agenus, Merck, GenMab, SeaGen, Mersana, and Clovis; personal fees for consulting or advisory board membership, or both, from AstraZeneca, Tesaro–GlaxoSmithKline, Immunogen, Ambry, Janssen–Johnson & Johnson, BBI, Agenus, AbbVie, Regeneron, Amgen, Novocure, Genentech–Roche, GOG Foundation, Iovance Biotherapeutics, Myriad Genetics, Eisai, Agenus, Tarveda, Merck, SeaGen, Novartis, Mersana, Clovis, Rubius, and Elevar, all outside the submitted work. OD reports grants or contracts from AstraZeneca, Clovis, Genentech, AbbVie, IMV, Millenium, and Pharmamar; payment or honoraria for lectures, presentations, speakers’ bureaus, manuscript writing, or educational events from Tesaro–GlaxoSmithKline and AstraZeneca; payment for expert testimony in one legal case unrelated to the current study; and participation on a data safety monitoring board or advisory board for Merck, Clovis, PACT, and Genentech, all outside the submitted work. SG reports financial support paid to her institution for the present study from the National Clinical Trials Network; grants or contracts from AstraZeneca, AbbVie, Pfizer, Rigel, Iovance, Tesaro, Genentech–Roche, PharmaMar, and GlaxoSmithKline; patents licensed to Sermonix (US patent numbers 10,905,659 and 10,258,604); participation on a data safety monitoring board or advisory board for Sermonix, Elevar Therapeutics, and GlaxoSmithKline; and service as an NRG Oncology phase 1 subcommittee co-chair, all outside the submitted work. BS reports personal fees for service on advisory boards for Abbvie, AstraZeneca, Bostongene, Clovis, Eisai, Genentech, GlaxoSmithKline, Jazz Pharmaceticals, Lilly, Merck, Myriad, Novartis, Nuvation, Onconova, and Seagen; and consulting for the GOG Foundation, all outside the submitted work. AE reports grants or contracts from UKRI for a post-doctoral fellowship outside the submitted work. CG reports grants from GlaxoSmithKline and Novartis for this study (trametinib, the test drug, was sold by GlaxoSmithKline to Novartis during the course of the study). WEB, JP, KCa, WR, DM, KCo, HG, PH, JF, MC, RLH, CSH, HQH, and LW declare no competing interests.
