# Supplementary material for: Trametinib versus standard of care in patients with recurrent low-grade serous ovarian cancer (GOG 281/LOGS): an international, randomised, open-label, multicentre, phase 2/3 trial
Source: Lancet. 2022 Feb 5;399(10324):541–53. doi: 10.1016/S0140-6736(21)02175-9 (PMC8819271; doi:10.1016/S0140-6736(21)02175-9)
Supplement: Supplementary appendix [file mmc1.pdf]

THE LANCET

Supplementary appendix

This appendix formed part of the original submission and has been peer reviewed. We post it as supplied by the authors.

Supplement to: Gershenson DM, Miller A, Brady WE, et al. Trametinib versus standard of care in patients with recurrent low-grade serous ovarian cancer (GOG 281/LOGS): an international, randomised, open-label, multicentre, phase 2/3 trial. *Lancet* 2022; **399:** 541–53.

Supplementary Material Appendix

Table of Contents

Supplementary Methods	2
Assessments related to procedures	2
Assessments related to quality of life	2
Statistical analysis	2-3
Supplementary Results	3
Comparison of study treatment with endocrine therapy or chemotherapy subgroups for QOL	3
References	4
Table S1: Number needed to treat	5
Table S2: Best overall response	6
Table S3: Relative dose intensity	7
Fig. S1: PFS trametinib vs pegylated liposomal doxorubicin	8
Fig. S2: PFS trametinib vs weekly paclitaxel	9
Fig. S3: PFS trametinib vs topotecan	10
Fig. S4: PFS trametinib vs tamoxifen	11
Fig. S5: OS trametinib vs SOC	12
Fig. S6: QOL trametinib vs SOC	13
Protocol GOG 0281	14
Final specification of statistical analyses relating clinical to biomarker data	167

Supplementary Methods

Assessments related to procedures
All patients underwent an electrocardiogram and either echocardiogram or multiple-gated acquisition (MUGA) scan at baseline. For patients receiving trametinib, electrocardiograms and either echocardiograms or MUGA scans were required before cycles 2, 5, 8, and 11, and then every 4 cycles. All patients underwent an ophthalmologic exam before receiving trametinib. If visual disturbances were reported, a follow-up ophthalmologic exam was required.

Assessments related to quality of life
The FACT-O TOI measures the general QOL of ovarian cancer patients. It consists of three subscales: Physical Well Being (7 items), Functional Well Being (7 items), and an Ovarian Cancer subscale (11 items). The FACT-GOG-NTX subscale consists of 4 items to measure chemotherapy-related peripheral neuropathy symptoms. Each item in the FACT-O TOI and the FACT-GOG-NTX subscale is scored using a 5-point scale (0=not at all; 1=a little bit; 2=somewhat; 3=quite a bit; 4=very much). For the negative statements (or questions), reversal was performed before score calculation. A FACT-O TOI total score ranges from 0-100, and an NTX subscale score ranges from 0-16, respectively. A higher score indicates better QOL for the FACT-O TOI and less neurotoxicity for the FACT/GOG-NTX subscales. The minimal clinically important difference (MCID) is 5 points for the FACT-O TOI and 1 point for the FACT-GOG-NTX subscale.[1]

Statistical analysis
The interim futility analysis was planned when 58 PFS events were observed in either arm (50% information time). The futility threshold was determined using a Rho family spending function with a parameter of 0·95.[2] A sample size of 250 patients was planned to accrue over 46 months, with 10 months of follow-up. The design targeted 80% power to detect a treatment hazard ratio (HR) of 0·67 in favor of trametinib using a one-sided alpha of 0·025 level log-rank test. This HR corresponds to a median PFS improvement from 8 months on the SOC arm to 12 months on the trametinib arm, assuming exponentially distributed PFS times. The final analysis was indicated when 112 PFS events occurred on the SOC arm. The primary analysis is used a log-rank test stratified by geographic region, performance status (0 or 1), and planned treatment regimen.
At the time of primary PFS analysis, 111 deaths were expected, giving 80% power for an OS hazard ratio of 0·67 using a 1-sided alpha = 0·25 (similar to a randomized phase 2 criterion). The log-rank test included all available follow-up for SOC patients, regardless of crossover. The OS estimates include all follow-up for patients on the SOC arm, including time after crossover to trametinib.
For the two QOL endpoints (FACT-O TOI and NTX scores), the overall type I error was controlled at alpha=0.10 (two-sided). The type I error or significance level for each QOL hypothesis was set to 5% by using a Bonferroni adjustment. All tests were two-sided.
Efficacy and QOL data were summarized in the intention-to-treat population and safety in patients receiving $\geq$ 1 dose of study therapy. PFS and OS distributions were summarized using Kaplan-Meier methods. Treatment effect hazard- and odds-ratios estimates were obtained from multivariable proportional hazards and logistic regression models with covariate effects for treatment and stratification factors used in the randomization. Homogeneity of the treatment effect across stratification levels was assessed using the same approach but including the interaction between the treatment and stratification factor of interest. The proportional hazards assumption was corroborated by testing a time-dependent treatment effect, and inspection of log (-log) survival plots. The date of the enrollment was taken as time zero for the Cox proportional hazards models. The PFS time at risk ended on the first observed date of disease progression, or death from any cause, with censoring on the date of the last patient visit. Three SOC patients who crossed over to trametinib before disease progression were censored on their crossover date. A sensitivity per-protocol analysis of PFS was also conducted.
QOL was analyzed using linear mixed models with an unstructured covariance matrix and adjusted for the pretreatment score, assessment time, age at enrollment, and stratification factors. The interaction between assessment time points and treatment assignments was tested first for the constant differential effects of treatment over time. If the interaction effect was not statistically significant, an overall treatment effect was estimated by a weighted average of estimates from each time point. If the treatment-time interaction test was rejected, the treatment comparison was performed for each assessment time. In this case, Hochberg's step-up multiplicity adjustment would be applied to timepoint-specific comparisons.[3]
For the initial biomarker analysis, archival formalin fixed paraffin embedded (FFPE) specimens were retrieved and underwent formal pathology review to confirm the presence of low-grade serous carcinoma with >40% tumor cellularity within an area of interest marked up by an expert gynecologic oncology pathologist. This area was

macrodissected and DNA isolation was performed. A minimum of 200ng of tumor DNA was required per patient. Whole exome sequencing of FFPE DNA was performed by Genuity Science using the Agilent SureSelect Human All Exon V6 Exome Capture Kit and Illumina NovaSeq 6000.
Sequence reads were aligned to the GRCh38 human reference genome using Burrow-Wheeler Aligner (bwa), duplicates were marked, and base quality scores recalibrated with the Genome Analysis Toolkit (GATK) v4·1·9 within the bcbio pipeline (v1·2·0) at the University of Edinburgh, UK. Adapters were trimmed from the reads. The cut-off for excessive FFPE DNA damage was set at >=3000 total base-to-base damage.
Variant calling was performed using a majority vote system with three variant caller algorithms: VarDict, Mutect2, and Freebayes. Filtering for FFPE and oxidation artifacts was applied using DKFZBiasFilter. Variants within *KRAS*, *NRAS*, and *BRAF* were extracted and filtered to remove variants at low allele frequency (<0·1), low sequencing depth at the locus (<20X), or a low number of variant-containing reads (<8 reads). Pathogenic mutations were flagged using ClinVar; remaining benign changes and variants of unknown significance were flagged as likely non-function.

Supplementary Results

Comparison of study treatment with endocrine therapy or chemotherapy for QOL
Exploratory QOL analyses compared patients receiving trametinib to SOC subsets defined by either endocrine therapy (letrozole or tamoxifen), or standard chemotherapy (paclitaxel, PLD or topotecan)..
A total of 54 patients (letrozole, n = 36; tamoxifen, n = 18) evaluable for QOL received standard endocrine therapy in the SOC group. For the comparison of trametinib with endocrine therapy, after adjustment for patient's age, baseline score, and stratification factors, the fitted mixed model estimates up to 24 weeks (4 weeks post-cycle 6) suggested that the treatment differences in the FACT-O TOI score varied significantly over the assessment times (p = 0·003 for interaction between assessment times and treatment groups). The estimated least squared mean treatment differences were -4·4 points (95% CI, -8·2 to -0·6; adjusted p-value = 0·044) at 12 weeks (pre-cycle 4) and 3·5 points (95% CI, -0·8 to 7·9; adjusted p-value = 0·22) at 24 weeks (4 weeks post-cycle 6), respectively, when compared to those receiving letrozole or tamoxifen in the control group. Thus, this analysis did not reach the minimal clinically important difference (MCID) for the FACT-O TOI scale.[1]
For the patient-reported FACT-GOG-NTX subscale scores, after adjustment for patient's age, baseline score, and stratification factors, the fitted mixed model estimates up to 24 weeks (4 weeks post-cycle 6) suggested that the treatment differences in the FACT-GOG-NTX subscale score did not vary significantly over the assessment times (p = 0·8 for the interaction between assessment times and treatment groups). The patients on trametinib reported 0·14 points higher (95% CI, -0·6 to 0·9; p-value = 0·7) on average across assessment times up to 24 weeks (4 weeks post-cycle 6) when compared to those receiving letrozole or tamoxifen in the control group.
A total of 42 patients evaluable for QOL received standard chemotherapy (paclitaxel, n = 7; PLD, n = 29; topotecan, n = 6) in the control group. After adjustment for patient's age, baseline score, and stratification factors, the fitted mixed model estimates up to 24 weeks (4 weeks post-cycle 6) suggested that the treatment differences in the FACT-O TOI score did not vary significantly over the assessment times (p = 0·17 for the interaction between assessment times and treatment groups). The patients on trametinib reported 1·26 points lower (95% CI, -5·2 to 2·7; p-value = 0·5) on average across assessment times up to 24 weeks (4 weeks post-cycle 6) when compared to those who received standard chemotherapy in the control group.
For the patient-reported FACT-GOG-NTX subscale scores, after adjustment for patient's age, baseline score, and stratification factors, the fitted mixed model estimates up to 24 weeks (4 weeks post-cycle 6) suggested that the treatment differences in the FACT-GOG-NTX subscale score did not vary significantly over the assessment times (p = 0·9 for the interaction between assessment times and treatment groups). The patients on trametinib reported 0·5 points higher (95% CI, -0·4 to 1·3; p-value = 0·26) on average across assessment times up to 24 weeks (4 weeks post-cycle 6) when compared to those who received standard chemotherapy in the control group.
In summary, compared to the standard endocrine therapy subgroup, the FACT-O TOI scores reported at 12 weeks (pre-cycle 4) in the trametinib group were lower by a statistically significant margin, but not clinically significant (the FACT-O TOI least squared mean difference = -4·4 (95% CI: -8·2 to -0·6)). No difference was noted in comparison to the standard chemotherapy group (the FACT-O TOI least squared mean difference – 1·6 (95% CI: -5.2 to 2.7)). For the FACT-GOG-NTX subscale scores, the primary QOL analyses found no difference in trametinib patients relative to the control group. Similarly, no treatment differences were found in the exploratory control subgroup comparisons.

References

1. Yost KJ, Eton DT. Combining distribution- and anchor-based approaches to determine minimally important differences: the FACIT experience. Eval Health Prof 2005;28:172-91.
2. Kim K, Demets DL. Design and analysis of group sequential tests based on the type I error spending rate function. Biometrika 1987;74:149-54.
3. Hochberg YA. sharper Bonferroni procedure for multiple tests of significance. Biometrika 1988;75:800-2.

Table S1. Number needed to treat at various times after starting treatment to prevent one progression/death with trametinib versus SOC.

Time for treatment (mos)	Number needed to treat (95% CI)	Number of patients still at risk
6	4·97 (3·89, 7·55)	166
12	3·83 (2·88, 6·15)	99
18	4·21 (2·92, 7·46)	46
24	5·93 (3·73, 11·85)	19
30	6·91 (4·18, 14·50)	10

Abbreviations: SOC = standard of care; mos = months.

Altman DG, Andersen PK. Calculating the number needed to treat for trials where the outcome is time to an event. BMJ 1999;319:1492-5.

Table S2. Best overall response (n = 260)

Best overall response	SOC	Trametinib	All
Complete	1 (1%)	1 (1%)	2 (1%)
Partial	7 (5%)	33 (25%)	40 (15%)
Stable disease	92 (71%)	77 (59%)	169 (65%)
Progressive disease	22 (17%)	9 (7%)	31 (12%)
Undetermined	8 (6%)	10 (8%)	18 (7%)
All	130 (100%)	130 (100%)	260 (100%)

Abbreviations: SOC = standard of care arm

Table S3. Relative dose intensity for trametinib and five standard of care drugs

	No. Patients	Median RDI (IQR)	Median No. Cycles (IQR)
Trametinib	127	75 (59-91)	8 (3-16)
Letrozole	44	100 (96-100)	10 (5-16)
Tamoxifen	27	82 (64-98)	4 (2-6)
PLD	40	100 (88-100)	6 (4-11)
Paclitaxel	11	100 (67-100)	4 (2-6)
Topotecan	8	98 (83-100)	2 (2-3)

Abbreviations: No. = number; RDI = relative dose intensity; PLD = pegylated liposomal doxorubicin.

Figure S1. PFS trametinib vs pegylated liposomal doxorubicin

Kaplan–Meier estimates of PFS comparing trametinib versus PLD among the 77 patients pre-specified to receive PLD if randomized to the SOC therapy.

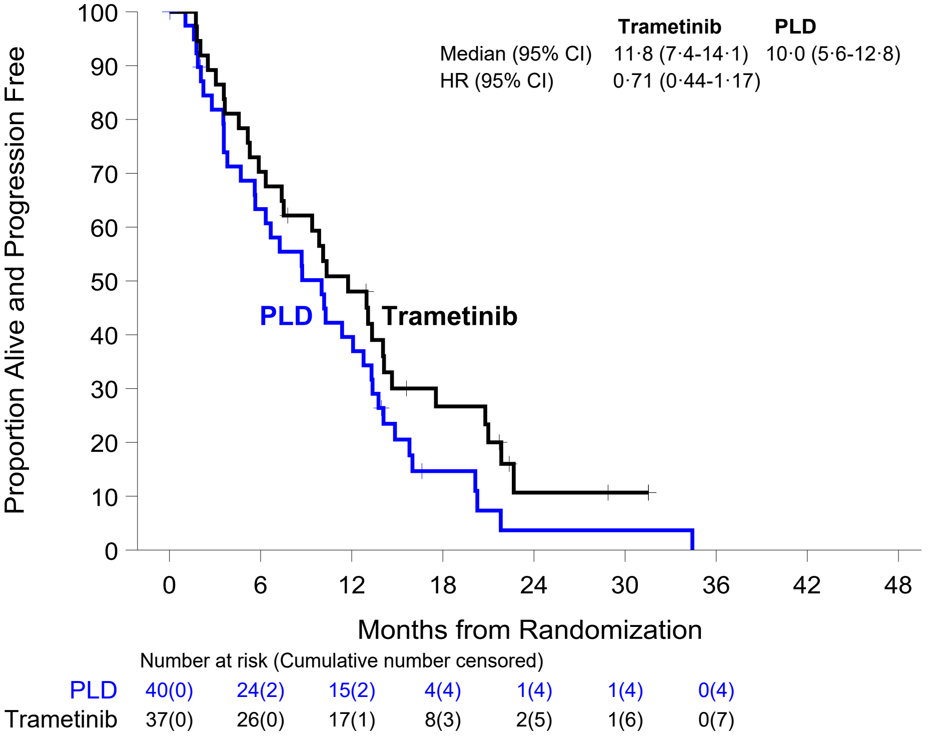

Figure S2. PFS trametinib vs weekly paclitaxel

Kaplan–Meier estimates of PFS comparing trametinib versus paclitaxel among the 54 patients pre-specified to receive paclitaxel if randomized to the SOC therapy.

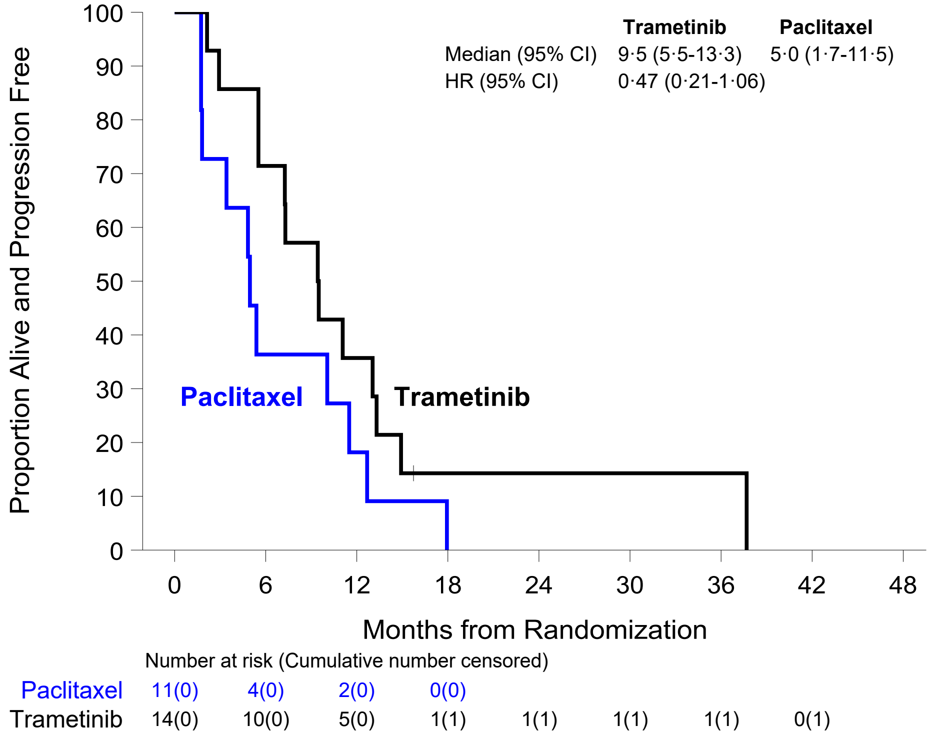

Figure S3. PFS trametinib vs topotecan

Kaplan–Meier estimates of PFS comparing trametinib versus topotecan among the 17 patients pre-specified to receive topotecan if randomized to the SOC therapy.

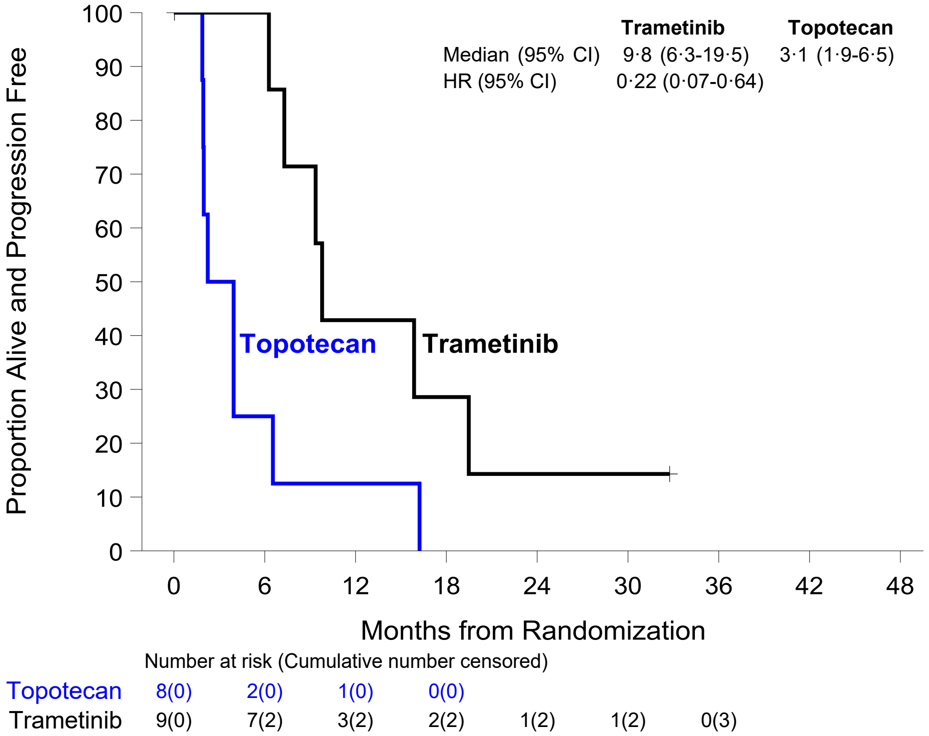

Figure S4. PFS trametinib vs tamoxifen

Kaplan–Meier estimates of PFS comparing trametinib versus tamoxifen among the 54 patients pre-specified to receive tamoxifen if randomized to the SOC therapy.

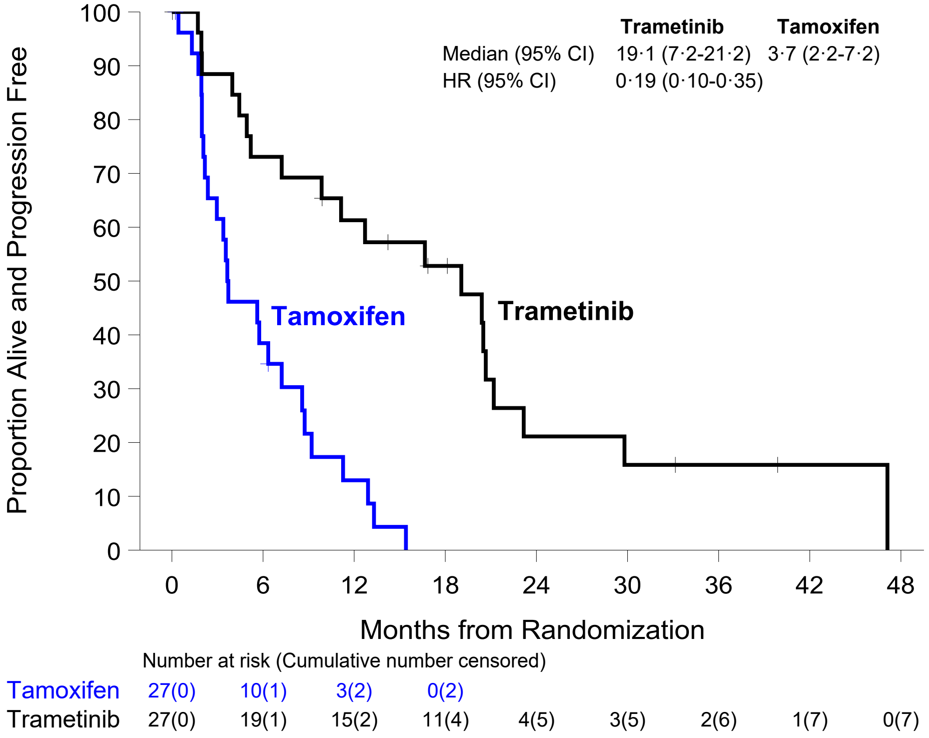

Figure S5. OS trametinib vs SOC

Intent to treat Kaplan–Meier estimates of overall survival comparing trametinib versus SOC.

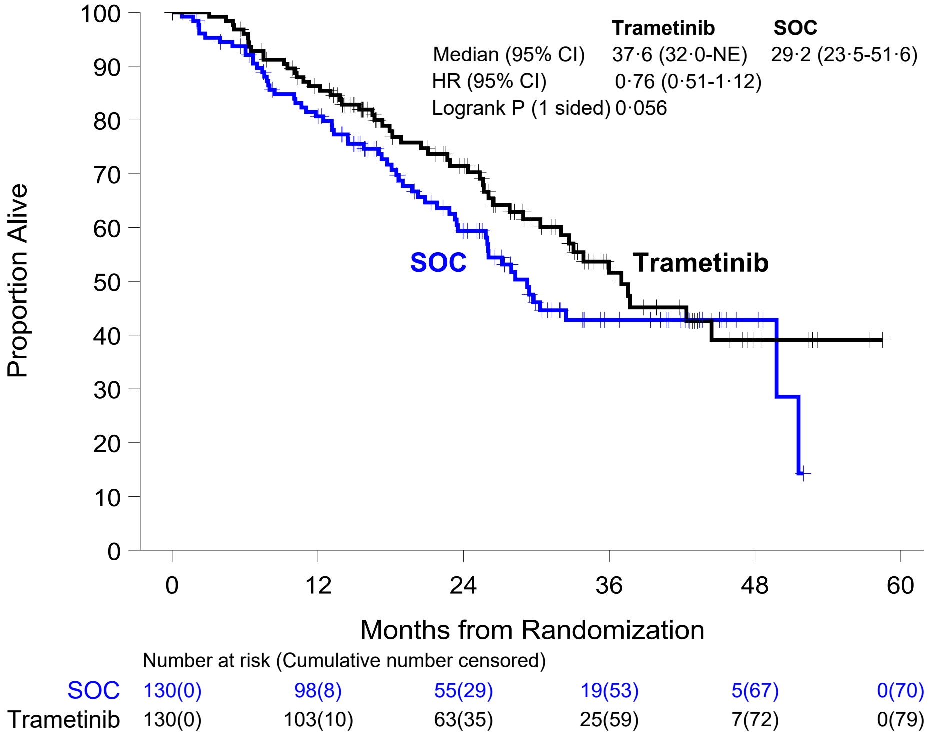

Figure S6. FACT-TOI trametinib vs SOC

Patient reported FACT-O-TOI score by randomized treatment groups. A larger score suggests better quality of life. The lines represent the mean FACT-O-TOI scores, which are displayed and accompanied by the standard deviation (SD) in brackets. The last two rows are the number of patients evaluable at each assessment time point.

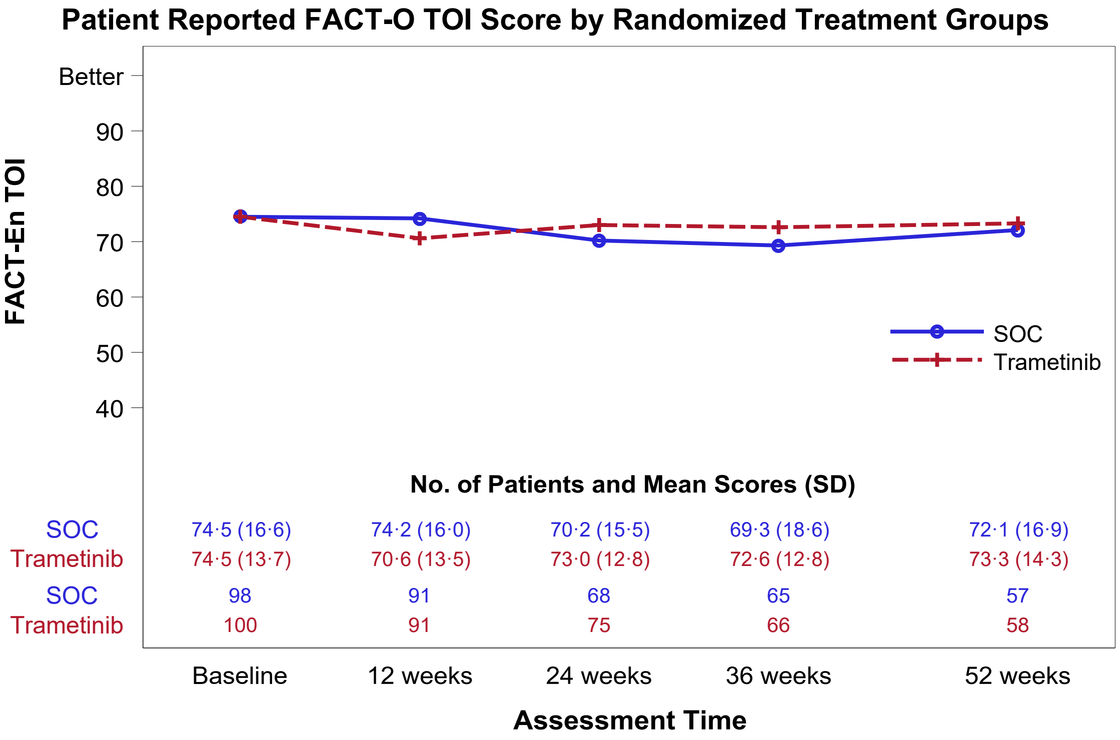

#	Section	Comments
1	Title Page	• NCI Version date has been updated<br>• Amendment 20 added
2	4.6	• Trametinib language updated with current DSMO language received in RA received from Dr. Johnson on June 5, 2020
3	ICF	• Version date has been updated.<br>• No other changes were made to ICF

PROTOCOL GOG-0281

A RANDOMIZED PHASE II/III STUDY TO ASSESS THE EFFICACY OF TRAMETINIB (GSK 1120212) IN PATIENTS WITH RECURRENT OR PROGRESSIVE LOW-GRADE SEROUS OVARIAN CANCER OR PERITONEAL CANCER. NCT # NCT02101788 (07/28/14)

NCI Version Date: July 24, 2020

Includes Amendments 1-20

POINTS:

PER CAPITA -

MEMBERSHIP –

TR PER CAPITA – Award based on specimen submission with 1.0 point for the frozen biopsy and 1.0 point for each FFPE and plasma (MAX = 7.0). **Note: PK plasma will be collected from approximately 12 GOG and 12 UK patients.**

Lead Organization: NRG / NRG Oncology (07/28/14)

Participating Organizations (07/28/14)
ALLIANCE / Alliance for Clinical Trials in Oncology
ECOG-ACRIN / ECOG-ACRIN Cancer Research Group
SWOG / SWOG

STUDY CHAIR
DAVID M. GERSHENSON, MD
THE UNIV. OF TEXAS, M.D. ANDERSON CANCER CENTER.
DEPARTMENT OF GYN/ONC, UNIT 1362
PO BOX 301439
HOUSTON TX, 77230 1439
(713)-745-2565
FAX: (713) 745-3510
EMAIL: dgershen@mdanderson.org

STUDY CO-CHAIR(S)
JOHN FARLEY, M.D.
See GOG Website Directory

QOL CO-CHAIR
LARI B. WENZEL, PHD
See GOG Website Directory

TR CO-CHAIR
ANIL SOOD, MD

See GOG Website Directory

SCIENTIFIC COLLABORATORS
KWONG WONG, PHD

JAMES BRENTON, MB, PHD

NURSE CONTACT
HEESUN KIM-SUH, BSN, RN
See GOG Website Directory

PATHOLOGIST
WILLIAM H RODGERS, MD, PHD
See GOG Website Directory

STATISTICIAN
AUSTIN MILLER, PH.D.
See GOG Website Directory

TR SCIENTIST
HEATHER A LANKES, PHD, MPH
See GOG Website Directory

This protocol was designed and developed by the Gynecologic Oncology Group (GOG). It is intended to be used only in conjunction with institution-specific IRB approval for study entry. No other use or reproduction is authorized by GOG nor does GOG assume any responsibility for unauthorized use of this protocol.

OPEN TO PATIENT ENTRY FEBRUARY 27, 2014: REVISED JULY 28, 2014; REVISED DECEMBER 22, 2014; REVISED JUNE 8, 2015; REVISED AUGUST 17, 2015; REVISED NOVEMBER 30, 2015; REVISED DECEMBER 14, 2015, TEMPORARILY CLOSED TO ACCRUAL FEBRUARY 2, 2016; REVISED MARCH 21, 2016; REOPENED TO PATIENT ENTRY APRIL 11, 2016; REVISED MAY 31, 2016; CLOSED TO ACCRUAL

CONTACT INFORMATION (07/28/14)(02-JUN-2020)

**For regulatory requirements:**	**For patient enrollments:**	**For data submission:**
Regulatory documentation must be submitted to the CTSU via the Regulatory Submission Portal.<br>(Sign in at www.ctsu.org, and select the Regulatory > Regulatory Submission.)<br><br>Institutions with patients waiting that are unable to use the Portal should alert the CTSU Regulatory Office immediately at 1-866-651-2878 to receive further instruction and support.<br><br>Contact the CTSU Regulatory Help Desk at 1-866-651-2878 for regulatory assistance.	Please refer to the patient enrollment section of the protocol for instructions on using the Oncology Patient Enrollment Network (OPEN) is accessed at https://www.ctsu.org/OPEN_SYSTEM/ or https://OPEN.ctsu.org.<br><br>Contact the CTSU Help Desk with any OPEN-related questions by phone or email : 1-888-823-5923, or ctsucontact@westat.com.	Data collection for this study will be done exclusively through Medidata Rave. Please see the data submission section of the protocol for further instructions.

The most current version of the **study protocol and all supporting documents** must be downloaded from the protocol-specific Web page of the CTSU Member Web site located at https://www.ctsu.org. Access to the CTSU members' website is managed through the Cancer Therapy and Evaluation Program - Identity and Access Management (CTEP-IAM) registration system and requires user log in with a CTEP-IAM username and password. Permission to view and download this protocol and its supporting documents is restricted and is based on person and site roster assignment housed in the CTSU RSS.

For clinical questions (i.e. patient eligibility or treatment-related) contact the Study PI of the Lead Protocol Organization.

For non-clinical questions (i.e. unrelated to patient eligibility, treatment, or clinical data submission) Contact the CTSU Help Desk by phone or e-mail:
CTSU General Information Line – 1-888-823-5923, or ctsucontact@westat.com. All calls and correspondence will be triaged to the appropriate CTSU representative.

For detailed information on the regulatory and monitoring procedures for CTSU sites please review the CTSU Regulatory and Monitoring Procedures policy located on the CTSU members' website https://www.ctsu.org > education and resources tab > CTSU Operations Information >CTSU Regulatory and Monitoring Policy

CRCTU / Cancer Research UK Clinical Trials Unit: (08/17/15) (11/30/15) (12/14/15) (03/21/16) (05/31/16) (09/19/16) (06/26/17)

**Institution Name**	**Institution CTEP ID**	**Investigator Name**	**Investigator CTEP ID**
Beatson West of Scotland Cancer Centre	64017	Dr Rosalind Glasspool	55234
Western General Hospital	64006	Professor Charles Gourley	55329
Christie Hospital	25113	Dr Andrew Clamp	55963
Musgrove Park Hospital	25102	Dr Clare Barlow	55807
St Bartholomew's Hospital	25050	Dr Melanie Powell	56284
University College London Hospital	98013	Dr Rebecca Kristeleit	55929
Hammersmith Hospital	25067	Professor Hani Gabra	56215
Addenbrookes Hospital-Medical School	25018	Dr Helena Earl	14680
Royal Marsden Institute of Cancer Research	25094	Dr Susana Banerjee	56316
Bristol Haematology and Oncology Centre	98015	Dr Axel Walther	56827
Nottingham City Hospital	25155	Dr Anjana Anad	57767
Belfast City Hospital	39013	Dr Joanne Millar	57325
Saint James's University Hospital	25011	Dr Geoff Hall	57317
Velindre Hospital	25171	Dr Emma Hudson	58710

SCHEMA

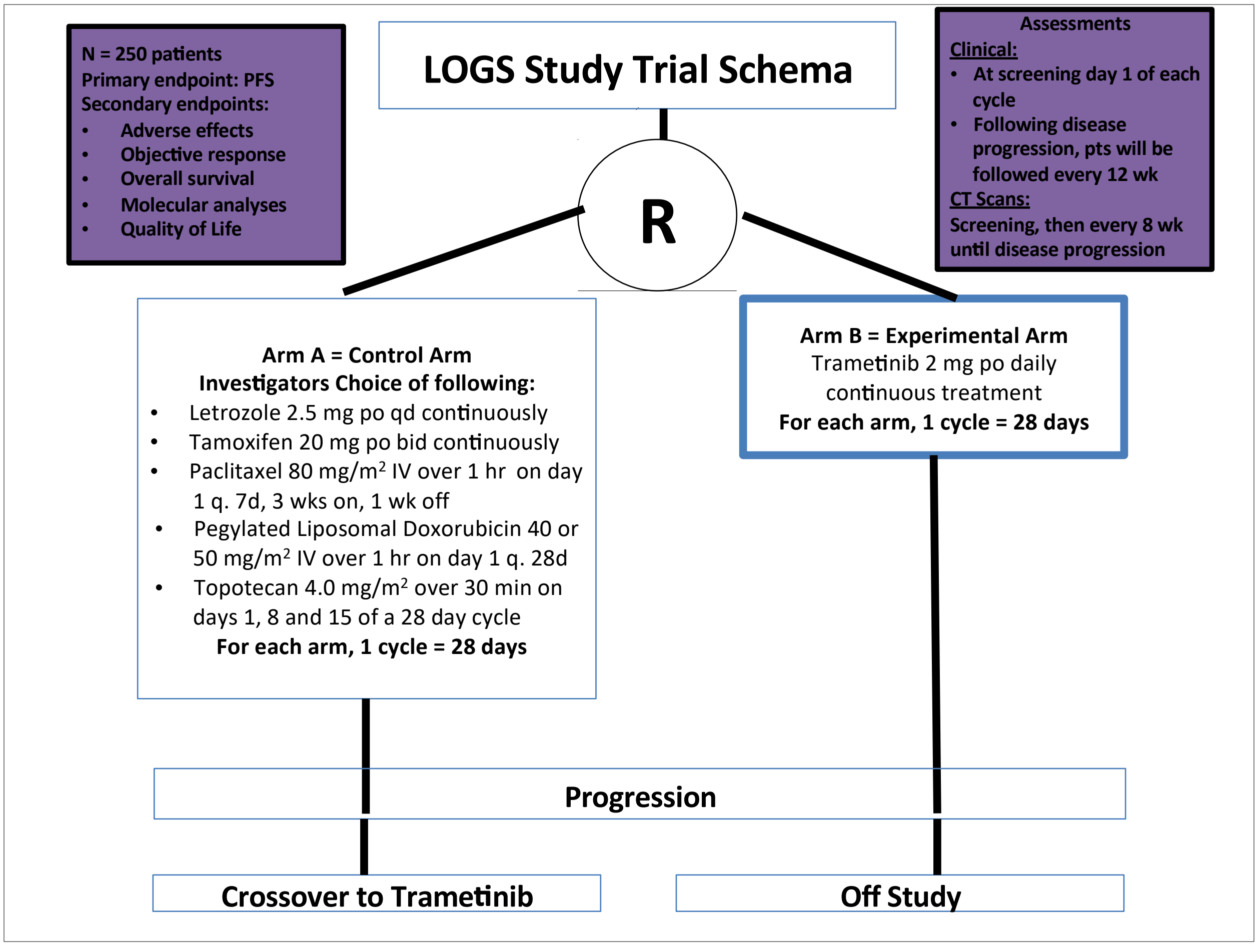
N = 250 patients
Primary endpoint: PFS
Secondary endpoints:
• Adverse effects
• Objective response
• Overall survival
• Molecular analyses
• Quality of Life
LOGS Study Trial Schema
R
Assessments
Clinical:
• At screening day 1 of each cycle
• Following disease progression, pts will be followed every 12 wk
CT Scans:
Screening, then every 8 wk until disease progression
Arm A = Control Arm
Investigators Choice of following:
• Letrozole 2.5 mg po qd continuously
• Tamoxifen 20 mg po bid continuously
• Paclitaxel 80 mg/m2 IV over 1 hr on day 1 q. 7d, 3 wks on, 1 wk off
• Pegylated Liposomal Doxorubicin 40 or 50 mg/m2 IV over 1 hr on day 1 q. 28d
• Topotecan 4.0 mg/m2 over 30 min on days 1, 8 and 15 of a 28 day cycle
For each arm, 1 cycle = 28 days
Arm B = Experimental Arm
Trametinib 2 mg po daily continuous treatment
For each arm, 1 cycle = 28 days
Progression
Crossover to Trametinib
Off Study

<u>TABLE OF CONTENTS</u>

PAGE

1.0	OBJECTIVES	9
1.1	Primary objective:	9
1.2	Secondary objectives:	9
2.0	BACKGROUND AND RATIONALE	9
2.1	Clinical Research Background	9
2.2	Translational Research Background	21
2.3	Integrated Biomarker Studies	21
2.4	Inclusion of Women and Minorities	22
3.0	PATIENT ELIGIBILITY AND EXCLUSIONS	23
3.1	Eligible Patients (06/08/15)	23
3.2	Ineligible Patients	25
4.0	STUDY MODALITIES	28
4.1	Letrozole (Femara) (NSC #719435)	28
4.2	Tamoxifen Citrate (Nolvadex®) (NSC #180973)	28
4.3	Pegylated Liposomal Doxorubicin (DOXIL®, NSC #712227, Lipodox™)	28
4.4	Topotecan (NSC #609699)	30
4.5	Paclitaxel (NSC#673089)	31
4.6	Trametinib dimethyl sulfoxide (GSK1120212B) (NSC 763093) (06/08/15)	31
5.0	TREATMENT PLAN AND ENTRY/RANDOMIZATION PROCEDURE (02-JUN-2020)	38
5.1	Registration Procedures (07/28/14)	38
5.2	Patient Screening (07/28/14) (06/08/15)	41
5.3	Patient Entry and Registration (07/28/14)	42
5.4	Treatment Plan	43

6.0 TREATMENT MODIFICATIONS 46

6.1 Control Treatments 46

6.2 Trametinib 46

7.0 STUDY PARAMETERS 64

7.1 Tests and Observations (07/28/14)(06/08/15) 64

7.2 Pathology Requirements 65

7.3 Translational Research 66

7.4 Quality of Life (12/22/14) 68

8.0 EVALUATION CRITERIA 71

8.1 Antitumor Effect – Solid Tumors 71

9.0 DURATION OF STUDY 79

9.1 Patients will receive therapy until disease progression or intolerable toxicity 79

9.2 All patients will be treated (with completion of all required case report forms) 79

9.3 All patients will be followed for 10 years after removal from study or until death, 79

9.4 In the absence of treatment delays due to adverse event(s), treatment may 79

10.0 STUDY MONITORING AND REPORTING PROCEDURES 80

10.1 ADVERSE EVENT REPORTING FOR AN INVESTIGATIONAL AGENT (CTEP IND) 80

10.2 ADVERSE EVENT REPORTING FOR A COMMERCIAL AGENT (06/08/15) 83

10.3 Medidata Rave Data Submission and Reporting (07/28/14)(03/21/16) 86

10.4 GOG DATA MANAGEMENT FORMS (06/08/15) 87

11.0 STATISTICAL CONSIDERATIONS 91

11.1 Design Summary 91

11.2 Principal Parameters 91

11.3 Accrual Goal, Accrual Rate, and Study Duration 92

11.4 Design and Analyses for the Primary Objective 92

11.5 Interim Analysis (11/27/17) 93

11.6 Monitoring Adverse Events and the Role of the GOG DSMB 93

11.7 Analyses for Secondary Objectives 94

11.8 Analyses for Integrated Biomarker Objectives 97

11.9 Anticipated Gender and Minority Inclusion 101

12.0 BIBLIOGRAPHY 102

APPENDIX I - CRADA/CTA LANGUAGE 107

APPENDIX II - NRG Oncology General Therapy Guidelines (06/08/15) 109

APPENDIX III - New York Heart Association Classifications 110

APPENDIX IV – Translational Research Specimen Procedures (07/28/14)(06/08/15) (11/27/17) 111

APPENDIX V - Patient Medication Calendar (12/22/14) 116

APPENDIX VI – FROZEN BIOPSY PATHOLOGY VERIFICATION (12/22/14) (11/27/17) 117

APPENDIX VII – Translational Research Laboratory Testing Procedures 118

APPENDIX VIII - UK Group Specific Appendix to GOG protocol (GOG-0281) (07/28/14) (12/14/15) (05/31/16) (09/19/16) (01/16/17) 126

1.0 OBJECTIVES

1.1 Primary objective:

1.11 To estimate the progression-free survival (PFS) hazard ratio of trametinib compared to that of "commercially available therapies" consisting of one of five commercially available agents in women with recurrent low-grade serous carcinoma of the ovary or peritoneum previously treated with platinum-based chemotherapy.

1.2 Secondary objectives:

1.21 To determine the nature, frequency and maximum degree of toxicity as assessed by CTCAE v4 for each treatment arm

1.22 To determine the quality of life, as assessed by the FACT-O

1.221 To compare trametinib to the control arm with regard to patients' self-reported acute (up to post-cycle 6) quality of life as measured by the FACT-O-TOI.

1.222 To compare trametinib to the control arm with regard to patients' self-reported acute (up to post-cycle 6) neurotoxicity as measured by the FACT-GOG-NTX.

1.23 To estimate the objective response rate (RR) of patients in each treatment arm.

1.24 To test whether high expression of pERK, as quantified by immunohistochemistry (IHC), is associated with better prognosis (RR or PFS) among patients receiving the randomized treatment.

1.25 To test whether genetic changes associated with MAPK pathway activation (KRAS, NRAS, HRAS, BRAF, MEK,ERBB2 or NF1) are associated with improved prognosis (RR or PFS) among patients receiving the randomized treatment.

2.0 BACKGROUND AND RATIONALE

2.1 Clinical Research Background

Serous carcinoma represents the most common histologic subtype of epithelial ovarian cancer. Although histologic grade has been found to be a major prognostic factor in women with epithelial ovarian cancer, no universal grading system exists.[1-5] After almost 20 years of analysis and refinement of the system, investigators at MD Anderson Cancer Center proposed a two-tier grading system for serous carcinoma of the ovary and subsequently found it to be highly reproducible, with excellent interobserver and intraobserver agreement.[6, 7] Importantly, a recent GOG study indicated that the two-tier grading system for serous carcinoma is superior to that of the International Federation of Gynecology and Obstetrics (FIGO).[8] Concomitantly, several other studies provided substantial

evidence to suggest that serous ovarian tumors of low malignant potential and low-grade serous carcinomas exist on a continuum and have molecular signatures and clinical behaviors distinct from those of the high-grade variety.[9-23]

Compared with other subtypes of ovarian cancer, advanced stage low-grade serous carcinoma is associated with a younger age at diagnosis and significantly longer overall survival but a higher rate of persistent disease at completion of primary platinum-based chemotherapy.[20] Confirmatory evidence of the relative chemoresistance of low-grade serous carcinoma is found in the MD Anderson experience with neoadjuvant chemotherapy for this disease.[22] Recurrent low-grade serous carcinoma may occur after an original diagnosis of serous tumor of low malignant potential or after a diagnosis of primary low-grade serous carcinoma.

Recurrences that are observed after a primary diagnosis of serous tumor of low malignant potential are low-grade serous carcinomas in 75-80% of cases.[24, 25] Whether recurrent low-grade serous carcinoma is diagnosed after an original diagnosis of serous tumor of low malignant potential or primary low-grade serous carcinoma, there is no standard therapy approach, both in terms of choice of agent or sequencing of agents. Because the overall survival of this patient cohort is prolonged, women typically receive multiple different treatments. Therapeutic options currently include conventional chemotherapy or hormonal therapy. Preliminary data suggest that recurrent low-grade serous carcinomas are relatively resistant to conventional chemotherapy agents, with an objective response rate of <5% but a stable disease rate of 60%.[22] In another report from the MD Anderson group, a variety of hormonal agents were found to result in a response rate of 9% among 64 patients who received a total of 89 hormonal regimens, but with a stable disease rate of 62%. [26] Median progression-free survival rates in these two reports was 7.2 and 7.4 months, respectively. Whether the high rate of stable disease relates more to tumor biology or the effects of therapy remains unclear. Nevertheless, findings to date appear to warrant separate clinical trials for women with low-grade serous carcinoma of the ovary using novel targeted agents.

Current therapies licensed for use in relapsed serous ovarian cancer following platinum-based chemotherapy are pegylated liposomal doxorubicin (Caelyx), weekly paclitaxel (Taxol) and topotecan (Hycamtin). Letrozole (Femara) and tamoxifen (Nolvadex) are often treatments of choice in this disease as well due to its indolent nature and poor response to cytotoxic therapeutics. However, there is little to no prospective evidence to assess their efficacy. High expression of ER and PR is seen providing a rationale for aromatase inhibitor therapy where a response rate of 9% is observed.[19, 26] These studies are based on retrospective data, and therefore, prospective study of targeted biological agents compared with available chemotherapeutic or hormonal agents is warranted.

Through the Gynecologic Oncology Group Rare Tumor Committee, the first national trial exclusively for patients with recurrent low-grade serous carcinoma—GOG-0239—was activated in December 2007 and closed to accrual in November 2009. This phase II trial studied the response rate of the MEK

inhibitor, Selumetinib, in the treatment of 52 women with recurrent low-grade serous carcinoma of the ovary or peritoneum.[27] Fifty-eight percent of the women had received three or more prior chemotherapy regimens. This study demonstrated a response rate to Selumetinib of 15.4% and a stable disease rate of 65%. Furthermore, the median progression-free survival time was 11 months—almost four months better than observed with chemotherapy or hormonal therapies in the MD Anderson reports. There were no treatment-related deaths. Grade 3 toxicities that occurred in more than one patient were gastrointestinal (13), dermatological (9), metaboli (7), fatigue (6), anemia (4), pain (4), constitutional (3), and cardiac events (2). Grade 4 toxicities were cardiac (1), pain (1), and pulmonary events (1). Mutational analysis of formalin-fixed, paraffin-embedded tissue from 34 patients with sufficient DNA was conducted; 38% had no mutation, 6% had *BRAF mutation,* 41% had *KRAS mutation,* and 15% had *NRAS* mutations. This analysis failed to demonstrate a correlation between mutation status and response. However, only primary tumor was available in 28/34 patients. In the present study, recurrent tissue will be studied in all patients using next generation sequencing, which will exclude issues with spacial or temporal biomarker instability. In addition, in as yet unpublished data, 33 of the 52 patients had pERK data available. Using a cut point of 109, there were statisically significant differences in the proportion of patients with complete or partial responses by any mutation (refer Table 1 in Appendix VII). Nevertheless, based on the promising response findings of this study, continuing to pursue the study of novel agents that target the MAP kinase pathway using a superior translational research design appears to be warranted. It is quite likely that the MAP kinase pathway is not linear and that we do not yet fully understand the complex circuitry under which it functions.

Singer et al. initially reported that the MAP kinase pathway may play a role in the pathogenesis of low-grade serous carcinoma.[10] In that study, 68% of low-grade serous carcinomas were found to have mutations in *BRAF* or *KRAS* (33% and 35%, respectively).[10] By comparison, the incidence of activation of this pathway in high-grade serous ovarian cancer is low, with p53 mutations being the predominant molecular lesion along with mutations or epigenetic inactivation of *BRCA1* or *BRCA2*. While subsequent studies have confirmed the relatively high frequency of KRAS mutations in low-grade serous carcinoma—19% in an MD Anderson report[28] and 41% in the GOG-0239 trial—BRAF mutations appear to be much less common than originally thought—2% in the MD Anderson series[28] and 6% in GOG-0239. Clearly, elucidating the mechanisms of action of agents that target this pathway in serous ovarian cancer is warranted.

Marked *in vitro* and *in vivo* sensitivity to a MEK inhibitor has been demonstrated in *KRAS* and *BRAF* mutated ovarian cancers,[29] suggesting that patients with this tumor type may benefit. In patients with *BRAF* V600E/K mutated melanoma, 40% had a complete or partial response to GSK1120212, a reversible highly selective allosteric inhibitor of MEK1 and MEK2, which is now in Phase III trials.

Further support for this type of clinical trial is provided by a Gynecologic Cancer Inter Group (GCIG) Ovarian Cancer Consensus Conference held in Vancouver, BC, in 2010, in which the consensus was to recommend separate clinical trials for women with low-grade serous carcinoma.[30] Subsequently, a National Cancer Institute Clinical Trials Planning Meeting held in Philadelphia in October 2011 agreed with this strategy [manuscript in preparation].

Upon progression of disease, patients on the standard treatment arm will be allowed to crossover to the experimental arm. While this complicates the interpretation of the comparison of overall survival (OS) between the two arms of the study and may even preclude meaningful interpretation of that endpoint, OS is not the primary endpoint of the study. Moreover, this patient population's long overall survival prognosis could make an OS comparison largely impractical anyway. And lastly, there are potentially other MEK inhibitors entering trials in this population, which could make crossover to a similar treatment regimen a likely possibility even outside this trial.

If a positive result is observed, with the MEK inhibitor leading to superior outcomes compared to "standard" therapy, at the very least, another therapeutic option would become available to women with this tumor subtype. At the most, a true standard therapy for women with recurrent low-grade serous carcinoma could become a reality, and the integration of the MEK inhibitor into primary therapy would also become a future focus.

The proposed study is an international collaboration between the GOG in the United States and National Cancer Research Network (NCRN) institutions in the UK. This study aims to assess the efficacy of a MEK inhibitor in the context of a randomized phase II/III study in a prospective manner for the first time in low-grade serous ovarian cancer. The investigation of mutational status of members of the RAS/RAF/MEK/ERK and PI3K/PTEN/Akt/mTOR pathways will be important to allow clinical correlation of treatment response in this rare disease.

2.11 Trametinib Dimethyl Sulfoxide (GSK1120212B, MEKINIST) (07/28/14)

The RAF-MEK-ERK pathway plays a critical role in multiple cellular functions. Activation of the pathway can result from activation/mutations of the upstream receptor tyrosine kinases (RTKs) and RAS, or upregulation/mutations in RAF and MEK. Upon activation, RAF acts as the MAPK kinase and activates MAPKK (MEK1/2), which in turn catalyze activation of the effectors ERK1/ERK2. Once activated, ERK1/2 translocate into the nucleus and phosphorylate a number of effector proteins and transcriptional factors that regulate cell proliferation, motility, differentiation, and survival.

Trametinib is one of the several MEK inhibitors in clinical development. On May 29, 2013, the U.S. Food and Drug Administration (FDA) approved trametinib for the treatment of patients with unresectable or metastatic melanoma with $BRAF^{V600E}$ or $BRAF^{V600K}$ mutations as detected by an FDA-approved test (U.S. Food and Drug Administration, 2013). On January 10, 2014, the Food and Drug

Administration granted accelerated approval to trametinib and dabrafenib for use in combination to treat patients with unresectable or metastatic melanoma with a BRAF V600E or V600K mutation as detected by an FDA-approved test (U.S. Food and Drug Administration, 2014).

Experience to date indicates that MEK is a valid target. In a phase 3 trial comparing trametinib with dacarbazine or paclitaxel in patients with BRAF V600E or V600K mutant metastatic melanoma, trametinib demonstrated a significantly better response rate, progression-free survival, and overall survival (Flaherty *et al.*, 2012). However, single agent activities are limited. Extensive research is underway to identify the patient selection markers and develop rational combination strategies. Preclinical studies have provided strong rationale and proof of principle for combination of MEK inhibitors with RTK inhibitors (EGFR or IGF-1R) (Gopal *et al.*, 2010; Ebi *et al.*, 2011), PI3K/AKT inhibitors (Engelman *et al.*, 2008; Hoeflich *et al.*, 2009), and mTOR inhibitors. On the other hand, the optimal dose/schedule and patient selection criteria for combination regimens have not been defined. Phase 1 results for a number of combinations have been reported, including AZD6244 + MK2206 (Tolcher *et al.*, 2011) and GDC-0973 + GDC-094 (MEK + PI3K inhibitor) (Bendell *et al.*, 2011).

The most up-to-date preclinical and clinical study information for trametinib can be found in the GSK1120212 (trametinib) Investigator's Brochure (2013).

2.111 Mechanisms of Action and Preclinical Data with Trametinib

Trametinib is a dimethyl sulfoxide (DMSO) solvate compound (ratio 1:1) with potent, allosteric and ATP non-competitive inhibition of MEK1/2 (IC_{50} of 0.7 and 0.9 nM against MEK1 and MEK2, respectively) (Gilmartin *et al.*, 2011). Trametinib inhibited MEK1/2 kinase activity and prevented RAF-dependent MEK phosphorylation (S217 for MEK1), producing prolonged pERK1/2 inhibition. Trametinib showed better potency against unphosphorylated MEK1/2 (u-MEK1/2) when compared with preactivated diphosphorylated MEK (pp-MEK), suggesting that u-MEK affords a higher affinity binding site for trametinib than does pp-MEK.

The specificity of trametinib was confirmed against a panel of 183 kinases, including MEK5 (the closet kinase homolog to MEK1/2), CRAF, BRAF, ERK1, and ERK2 (Yamaguchi *et al.*, 2011). Trametinib demonstrated equal potency against activated MEK1- and MEK2-mediated phosphorylation of ERK (sequence identity of 85% across the whole protein and 100% in the active site for humans). Trametinib demonstrated preferential inhibition of RAF-mediated MEK1 activation (IC_{50} = 0.60 nM) over pMEK1 kinase activity (IC_{50} = 13 nM) (Investigator’s Brochure, 2012a).

BRAF-mutant Colo205, A375P F11s, and HT-29 human tumor xenograft mouse models showed the most significant mean tumor growth inhibition (TGI) (80% to 87%) at 3.0 mg/kg trametinib, with multiple complete and partial tumor regressions. In the Colo205 model, tumor regression was

observed even at a dose of 0.3 mg/kg (Yamaguchi *et al.*, 2011). Two KRAS-mutant xenograft models, HCT-116 and A549, also showed significant TGI (83% and 75%) but without significant tumor regressions (Gilmartin *et al.*, 2011). As predicted by cell proliferation assays, tumor xenograft lines with wild-type (wt) RAF/RAS (PC3, BxPC3, and BT474) were much less sensitive, showing only modest TGI (44-46%) with no tumor regressions.

Pharmacodynamic studies were performed in mice treated with trametinib for 14 days (Gilmartin *et al.*, 2011). In the A375P F11s xenograft model, the first dose of trametinib (3 mg/kg) significantly reduced pERK for more than 8 hours on Day 1. pERK inhibition was more sustained (over 24 hours) after the Day 7 dose, probably due to an increase in the steady-state levels of trametinib after repeated doses. The average C_{max} in blood was 1,410 nM on Day 7, with an estimated half-life ($t_{1/2}$) of 33 hours. In addition, immunohistochemistry (IHC) also confirmed inhibition of cell proliferation (reduced Ki67) and G1 cell cycle arrest (elevated p27Kip1/CDKN1B) following 4 days of treatment.

2.112 Clinical Pharmacokinetics (PK) and Activity of Trametinib

FTIH Phase 1 Trial of Trametinib Monotherapy (MEK111054)
There are 3 parts in this study. Part 1: The dose-escalation portion involves administration of trametinib (repeat doses of 0.125 mg to 4.0 mg) to patients with solid tumors or lymphoma in one of three schedules - (1) QD for 21 days followed by 7 days without drug, (2) loading dose on Day 1 or Day 1-2, followed by QD with the designated dose, or (3) QD dosing without a drug holiday. Part 2: cohort expansion at the recommended phase 2 dose (RP2D) for pancreatic cancer, melanoma, non-small cell lung cancer (NSCLC), colorectal cancer (CRC), or any BRAF mutation-positive cancer. Part 3: expansion to characterize the biologically active range of trametinib via analysis of pharmacodynamic biomarkers (biopsies or FDG-PET).

The MTD of trametinib was established as 3 mg QD, but the recommended phase 2 dose (RP2D) was chosen at 2 mg QD based on tolerability of repeated cycles (Infante *et al.*, 2010).

PK and metabolism of trametinib:
PK measurements were conducted under fasting conditions. After a single dose (Day 1), AUC_{0-24} and C_{max} values were dose-proportional up to 6 mg, lower than dose proportional following 8 mg, and greater than dose proportional following the 10 mg dose. Median T_{max} was 1.5 hours.

After repeat doses (Day 15), trametinib accumulated with a mean accumulation ratio of 6.6 at the RP2D of 2 mg QD. Between-subject variability in exposure ranged from 27-50% for C_{max} and 20-41% for AUC_{0-24} across all dosing regimens. The effective $t_{1/2}$ was approximately 4.5 days, and steady state was reached by approximately Day 15. Trametinib had a small peak:trough ratio of ~2 (Infante *et al.*, 2010). At 2 mg QD on Day 15, mean

AUC_{0-24} was 376 ng•h/mL and C_{max} 23 ng/mL, and the mean trough concentrations ranged from 10.0 to18.9 ng/mL. The long half-life and small peak:trough ratio of trametinib allowed constant target inhibition within a narrow range of exposure.

Drug-drug interactions:
Trametinib is metabolized predominantly via deacetylation (non-cytochrome P450 [CYP450]-mediated) with secondary oxidation or in combination with glucuronidation biotransformation pathways (Investigator's Brochure, 2012a). The deacetylation is likely mediated by hydrolytic esterases, such as carboxylesterases, or amidases. Based on *in vitro* studies, trametinib is not an inhibitor of CYP1A2, CYP2A6, CYP2B6, CYP2D6, and CYP3A4. Trametinib has an overall low potential for drug-drug interactions.

Pharmacodynamic effect and biomarkers:
The relationship between dose and tumor biomarkers such as pERK, Ki67, and p27, were evaluated in patients with BRAF or NRAS mutation-positive metastatic melanoma (Investigator's Brochure, 2012a). In general, increasing exposures and/or doses provided greater pharmacodynamic effects. The median change observed at a dose of 2 mg QD was 62% inhibition of pERK, 83% inhibition of Ki67, and a 175% increase in p27.

Antitumor Activity in the FTIH phase 1 trial:
In the FTIH phase 1 trial, 14 patients with BRAF-mutant melanoma received trametinib at 2 mg QD. The overall objective response rate (ORR) was 43% (6/14), including 2 complete responses (CRs) (Investigator's Brochure, 2012a). In 9 patients with BRAF wt melanoma, 2 patients achieved a partial response (PR), and 3 stable disease (SD) (Infante *et al.*, 2010). In 26 evaluable pancreatic cancer patients, there were 2 PRs (1 PR was KRAS mutation-positive) and 11 SD (2 achieved ≥20% tumor reduction) (Messersmith *et al.*, 2011). Among the 27 CRC patients (without selection of RAS or RAF mutations), 8 SD were observed.

Antitumor Activity in Melanoma

Phase 3 trial of trametinib vs. chemotherapy in advanced V600 mutant melanoma:
In a phase 3 trial, patients with unresectable stage IIIC or IV cutaneous melanoma with a BRAF V600E or V600K mutation were randomized (2:1) to trametinib (2 mg, PO, QD) or chemotherapy (dacarbazine or paclitaxel) (Flaherty *et al.*, 2012; MEKINIST, 2013). There were 322 patients in the intention-to-treat (ITT) population, of whom 273 (85%) were in the primary efficacy population (patients with $BRAF^{V600E}$-positive cancer who did not have brain metastases at baseline). Of the patients, 214 were randomized to receive trametinib, and 108 were randomized to receive chemotherapy. Investigator-assessed efficacy data are summarized as follows:

	Trametinib (n=214)	Chemotherapy (DTIC) (n=108)
**PFS**<br>Median, months (95% CI)	4.8 (4.3, 4.9)	1.5 (1.4, 2.7)
HR (95% CI)<br>*P* value (log-rank test)	0.47 (0.34, 0.65)<br>*P*<0.0001	
**Confirmed Tumor Responses**<br>Objective Response Rate (95% CI)<br>CR, n (%)<br>PR, n (%)	22% (17, 28)<br>4 (2%)<br>43 (20%)	8% (4, 15)<br>0<br>9 (8%)
Duration of response<br>Median, months (95% CI)	5.5 (4.1, 5.9)	NR (3.5, NR)
CI = confidence interval; CR = complete response; HR = hazard ratio; NR = not reached; PFS = progression-free survival; PR = partial response		

The 6-month OS rate was 81% in the trametinib group and 67% in the chemotherapy group. Mature data on OS are pending.

Experience with Trametinib in Metastatic Melanoma Following BRAF Inhibitor Therapy

The clinical activity of single-agent trametinib was evaluated in a single-arm, multicenter, international trial in 40 patients with BRAF V600E or V600K mutation-positive, unresectable, or metastatic melanoma who had received prior treatment with a BRAF inhibitor. All patients received trametinib at a dose of 2 mg PO QD until disease progression or unacceptable toxicity. None of the patients achieved a confirmed PR or CR.

Antitumor Activity of Trametinib in Cancer Other Than Melanoma

In a phase 1/2 monotherapy study, acute myeloid leukemia (AML) or myelodysplastic syndrome (MDS) patients were given trametinib at dose levels from 1-2 mg QD. Drug-related AEs in 45 patients were similar to that observed in patients with solid tumors, and 2 mg PO QD was selected for further investigation in this patient population. Twelve patients (23%) withdrew due to an AE, including cardiac failure (2) and infection (2). Efficacy was reported in 39 patients (Borthakur *et al.*, 2010). The best response in 13 patients with KRAS or NRAS mutations included 3 CRs (23%), 7 SD (54%), and 1 PD (progressive disease) (5%). In 26 patients with wild-type RAS or an unknown mutation, there were 2 PRs (8%).

In a multicenter phase 2 study, NSCLC patients with KRAS mutant tumors were randomized 2:1 to receive trametinib (2 mg QD) or docetaxel (75 mg/m^2 IV every 3 weeks) (Blumenschein *et al.*, 2013). A total of 134 pts were randomized to trametinib (89) or docetaxel (45); 129 patients had KRAS-mutant NSCLC. The hazard ratio for PFS was 1.14 (95% CI, 0.75-1.75; *P*=0.5197) with a median PFS of 11.7 versus 11.4 weeks for

trametinib versus docetaxel. The overall response rate (ORR) was 12% for trametinib and 12% for docetaxel.

In a double-blind, phase 2 study evaluating the combination of gemcitabine with trametinib, untreated pancreatic cancer patients were randomized to receive gemcitabine (1000 mg/m2 weekly ×7 for 8 weeks, then weekly ×3 every 4 weeks) plus either trametinib 2mg or placebo QD (Infante *et al.*, 2013). Median OS was 8.4 months with trametinib compared to 6.7 months with placebo. Median PFS was 16 weeks versus 15 weeks, and ORRs and median duration of responses were 22% and 23.9 weeks and 18% and 16.1 weeks on trametinib and placebo; the median OS and ORR in the subgroup of patients with KRAS mutations (143/160) was similar to OS and ORR for all randomized patients.

2.113 Trametinib Safety Profile

A **Comprehensive Adverse Events and Potential Risks (CAEPR)** list using NCI Common Terminology Criteria for Adverse Events (CTCAE) terms is included in Section 4.6 of the protocol.

Based on available AE data from clinical studies involving trametinib to date, the most common toxicities are rash and diarrhea. Rash and diarrhea are common, class-effect toxicities for MEK inhibitors. In addition, visual impairment and left ventricular ejection fraction (LVEF) reduction, although observed at lower frequencies, are also considered class-effect toxicities as they have been observed with trametinib as well as other MEK inhibitors.

AEs of special interest:
Rash, diarrhea, visual disorders, hepatic disorders, cardiac-related AEs, and pneumonitis are considered AEs of special interest because they are either known class effects (*i.e.*, have been observed with other MEK inhibitors) or are potentially life-threatening (Investigator’s Brochure, 2013). The following sections provide integrated summaries for these AEs across different clinical trials, with emphasis on trials using trametinib as monotherapy, especially at the RP2D of 2 mg.

Refer to dose modification guidelines for the toxicities for which they are addressed in Section 6.

<u>Rash</u>: Rash was a common AE observed across different dose levels and in different combinations (Investigator’s Brochure, 2013). At the 2 mg dose, rash was seen in 27% to 78% of patients in different trials. Of the ~370 subjects with rash AEs at the 2 mg monotherapy dose (including crossover subjects) in five studies, the majority of rash AEs were grades 1 or 2 (24% to 73%); 0% to 9% of patients experienced grade 3 rash AEs, and four patients had a grade 4 rash AE.

In a randomized phase 3 trial of trametinib vs. chemotherapy, the overall incidence of skin toxicity (including rash, dermatitis, acneiform rash, palmar-plantar erythrodysesthesia syndrome, and erythema) was 87% in patients treated with trametinib and 13% in chemotherapy-treated patients. Severe skin toxicity occurred in 12% of patients on the trametinib arm, most commonly for secondary infections of the skin. The median time to onset of skin toxicity was 15 days (range: 1 to 221 days), and median time to resolution was 48 days (range: 1 to 282 days). Dose reduction was required in 12% for skin toxicities, and permanent discontinuation of trametinib was required in 1% of patients.

Diarrhea: At the 2 mg monotherapy dose, 33% to 58% of patients in five trials had diarrhea (Investigator's Brochure, 2013). Of ~320 subjects (including crossover subjects) with diarrhea at this dose, the majority of diarrhea AEs were grade 1 or 2 in severity (33% to 56% of all study patients); 17 patients had grade 3 diarrhea, and none had grade 4 diarrhea.

Visual disorders: At the 2 mg monotherapy dose, 4% to 21% of the patients in five trials experienced visual disorders (Investigator's Brochure, 2013). Of the 85 total subjects (including crossover subjects) experiencing visual disorders at this dose level, the majority of visual disorders were grades 1 or 2 (4% to 20% of all study patients); six patients experienced grade 3 visual disorders, and one patient experienced a grade 4 visual disorder.

- *Retinal Pigment Epithelial Detachment (RPED):* Also known as chorioretinopathy, RPED is a visual impairment due to fluid accumulation under the retina and causes blurry vision. There were five cases of RPED, previously termed central serous retinopathy, reported from the integrated trametinib safety population consisting of subjects treated with trametinib 2 mg once daily from five studies (Investigator's Brochure, 2013). As of 23 June 2013, 14 cases of RPED were reported across the entire trametinib program amongst subjects treated with trametinib either as monotherapy or in combination with other anti-cancer agents (including cases from a MEK/BRAF combination study).

- *Retinal vein occlusion (RVO):* As of 23 June 2013, a total of four cases of RVO were reported across the entire trametinib program (including one case from a MEK/BRAF combination study) (Investigator's Brochure, 2013). All cases of RVO occurred in one eye only. Study drug was stopped at time of diagnosis in all cases. There was a decrease of visual acuity in two subjects with central RVO (CRVO) while the other two subjects had no meaningful decrease of visual acuity. In the two subjects with CRVO, local treatment with intravitreal injections of anti-VEGF antibodies was initiated within 2 weeks after RVO diagnosis, and visual acuity improved in one subject and restored to baseline conditions in another

subject, at the time of the data cutoff. Three of these four cases were considered related to study treatment by the investigators.

Hepatic disorders: Abnormalities of liver enzymes and bilirubin have been observed with administration of trametinib (Investigator's Brochure, 2013). However, assessment of these cases was often confounded by co-morbid conditions (such as biliary obstruction), concomitant use of other potentially hepatotoxic drugs, and liver metastases. At the 2 mg monotherapy dose, 8% to 34% of patients in five trials had LFT abnormalities. Of the 96 total patients (including crossovers) with LFT changes, the majority were grade 1 or 2 in severity (4% to 20% of all study patients); 26 had grade 3 events, and 6 patients had grade 4 events.

Cardiac-related AEs: At the 2 mg monotherapy dose, 3% to 21% of the subjects in six studies had cardiac-related AEs (Investigator's Brochure, 2013). Of the 65 total subjects (including crossover subjects) experiencing cardiac-related AEs at the 2.0 mg monotherapy dose in five of the studies, the majority of cardiac-related AEs were grades 1 or 2 in severity (0% to 16% of all study subjects); 18 subjects had grade 3 cardiac-related AEs, and no subjects had Grade 4 cardiac-related AEs in any study. No subject in one study, which evaluated the effect of repeat oral dosing of trametinib 2 mg QD on cardiac repolarization in subjects with solid tumors, had cardiac-related AEs. One study subject receiving trametinib 2 mg QD had grade 5 (fatal) acute cardiac failure, with evidence of massive tumor invasion of the heart; this AE was considered not drug-related by the investigator.

In the phase 3 trial of trametinib vs. chemotherapy in patients with melanoma (MEK114267), cardiomyopathy (defined as cardiac failure, left ventricular dysfunction, or decreased LVEF) occurred in 7% (14/211) of patients treated with trametinib, and in no patients in the chemotherapy arm. Cardiomyopathy was identified within the first month of treatment in five of these 14 patients; median onset of cardiomyopathy was 63 days (range: 16 to 156 days). Cardiomyopathy resolved in 10 of these 14 (71%) patients. Cardiac monitoring should be included in trametinib protocols, to include LVEF assessment by echocardiogram or MUGA scan at baseline, one month after initiation of trametinib and then at 2- to 3-month intervals while on treatment. Refer to dose modification guidelines for cardiac AEs in the event of LVEF decline or symptomatic cardiac AEs.

Pneumonitis: At the 2 mg monotherapy dose, 0% to 4% of the subjects in five studies had pneumonitis (Investigator's Brochure, 2013). Of the nine total subjects (including crossovers) experiencing pneumonitis AEs at this dose, three subjects had grade 1 or 2 pneumonitis and six subjects had grade 3 pneumonitis.

Embryofetal toxicity: Based on its mechanism of action, trametinib can cause fetal harm when administered to a pregnant woman. Trametinib

was embryotoxic and abortifacient in rabbits at doses greater than or equal to those resulting in exposures approximately 0.3 times the human exposure at the recommended clinical dose. If this drug is used during pregnancy, or if the patient becomes pregnant while taking this drug, the patient should be apprised of the potential hazard to a fetus.

Incidence of common AEs reported from a phase III trial of trametinib vs. chemotherapy in patients with advanced melanoma:

Patients with abnormal LVEF, history of acute coronary syndrome within 6 months, or current evidence of Class II or greater congestive heart failure (New York Heart Association) were excluded from this trial. Selected adverse reactions (AR) occurring in patients receiving trametinib as compared to patients in the chemotherapy arm are listed as below:

Table: Selected adverse reactions (ARs) occurring in ≥10% of patients receiving trametinib AND at a higher incidence than in the chemotherapy arm (high in the trametinib arm compared with chemotherapy by ≥5% in overall incidence or by ≥2% grade 3 or 4 AEs)

	**Trametinib (n=211)**		**Chemotherapy (n=99)**	
**Adverse Reactions**	**All Grades**	**Grades 3 and 4**	**All Grades**	**Grades 3 and 4**
**Skin and subcutaneous tissue disorders**				
Rash	57	8	10	0
Dermatitis acneiform	19	<1	1	0
Dry skin	11	0	0	0
Pruritis	10	2	1	0
Paronychia	10	0	1	0
**Gastrointestinal disorders**				
Diarrhea	43	0	16	2
Stomatitis	15	2	2	0
Abdominal pain	13	1	5	1
**Vascular disorders**				
Lymphedema	32	1	4	0
Hypertension	15	12	7	3
Hemorrhage	13	<1	0	0

Table: Percent-patient incidence of laboratory abnormalities occurring at a higher incidence in patients treated with trametinib versus chemotherapy (between-arm difference of ≥5% [all grades] or ≥2% [grades 3 or 4])

	**Trametinib (n=211)**		**Chemotherapy (n=99)**	
**Preferred term**	**All Grades**	**Grades 3 and 4**	**All Grades**	**Grades 3 and 4**
Increased aspartate aminotransferase (AST)	60	2	16	1
Increased alanine aminotransferase (ALT)	39	3	20	3
Hypoalbuminemia	42	2	23	1
Anemia	38	2	26	3
Increased alkaline phosphatase	24	2	18	3

Other clinically important adverse reactions observed in ≤10% of patients (n=329) treated with trametinib were: nervous system disorders (dizziness, dysgeusia), ocular disorders (blurred vision, dry eye), infections and infestations (folliculitis, rash pustular, cellulitis), cardiac disorders (bradycardia), gastrointestinal disorders (xerostomia), and musculoskeletal and connective tissue disorders (rhabdomyolysis).

2.2 Translational Research Background

Mutation Analysis

RAF/RAS mutations have been shown to be a strong predictor of sensitivity to MEK inhibitors.[31] Formalin-fixed, paraffin-embedded (FFPE) primary, metastatic, and/or recurrent tumor will be collected from consenting patients and used for mutational analysis. Whole exome sequencing analysis will be performed on all fixed tumor submitted prior to treatment. Results from mutational analyses of KRAS, NRAS, HRAS, BRAF, MEK, ERBB2 and NF1 mutations will be correlated with tumor response and PFS.

Immunohistochemistry

FFPE tumor will be used for immunohistochemical analysis of pERK. Tumor response will be correlated with expression levels of pERK.

Population Pharmacokinetics

A population PK analysis will be completed utilizing a sparse PK sampling design on a subset of patients (approximately 12 GOG and 12 UK) to assess the pharmacokinetics of trametinib in this patient population. Population pharmacokinetic parameters including but not limited to clearance (CL) and volume of distribution (Vd), depending on the final compartmental model describing trametinib concentrations will be derived. If the data permit, a population PK/PD analysis may be completed to explore the trametinib exposure-response relationship including but not limited to safety, efficacy and/or toxicity.

2.3 Integrated Biomarker Studies

Low-grade ovarian serous carcinomas (LGSC) account for a significant proportion of epithelial ovarian cancers in young women. The exact biological pathways that underlie this disease are incompletely understood. Because the Ras/Raf/MEK/ERK pathway is frequently activated in LGSC, targeting this pathway has been examined as a potential treatment.[27] In this study, integrated biomarker studies will test whether (1) high expression of pERK is associated with a low hazard of progression among patients treated with trametinib by immunohistochemistry (IHC), and (2)

KRAS, NRAS, HRAS, BRAF, MEK, ERBB2 and NF1 mutations are associated with a lower hazard of progression among patients treated with trametinib by whole exome sequencing. Please refer to Appendix VII for additional background information and assay details.

2.4 Inclusion of Women and Minorities

The Gynecologic Oncology Group and GOG participating institutions will not exclude potential subjects from participating in this or any study solely on the basis of ethnic origin or socioeconomic status. Every attempt will be made to enter all eligible patients into this protocol and therefore address the study objectives in a patient population representative of the entire serous ovarian and peritoneal population treated by participating institutions.

3.0 PATIENT ELIGIBILITY AND EXCLUSIONS

3.1 Eligible Patients (06/08/15)

3.11 Patients age greater than 18 with the following tumors are included in the study:

3.111 Patients initially diagnosed with low-grade serous ovarian or peritoneal carcinoma that recur as low-grade serous carcinoma (invasive micropapillary serous carcinoma or invasive grade I serous carcinomas as defined by GOG, FIGO WHO or Silverberg).

3.112 Patients initially diagnosed with serous borderline ovarian or peritoneal carcinoma that recur as low-grade serous carcinoma (invasive micropapillary serous carcinoma or invasive grade I serous carcinomas as defined by GOG, FIGO WHO or Silverberg).

3.12 At least 4 weeks must have elapsed since the patient underwent any major

Surgery (eg. MAJOR: Laparotomy, laparoscopy, thoracotomy, VATS (video assisted thorascopic surgery). There is no restriction on MINOR procedures: (eg. central venous catheter placement, ureteral stent placement or exchange, tumor core or FNA biopsy). (12/22/14)

3.13 Patients must have documented low-grade serous carcinoma. Confirmation must occur by prospective pathology review prior to study entry (as specified in Section 7.1). The prospective pathology review can be done on tissue from the recurrent carcinoma or from original diagnostic specimen. (12/22/14)

3.14 All patients must have measurable disease as defined by RECIST 1.1. Measurable disease is defined as at least one target lesion that can be accurately measured in at least one dimension (longest diameter to be recorded). Each lesion must be $\geq$ 10 mm when measured by CT, MRI, or caliper measurement by clinical exam; or $\geq$ 20 mm when measured by chest x-ray. Lymph nodes must be $\geq$ 15 mm in short axis when measured by CT or MRI. (06/08/15)

All imaging studies must be performed within 28 days prior to registration.

3.15 Prior therapy (12/22/14)

3.151 Patients must have recurred or progressed following at least one platinum-based chemotherapy regimen.

3.152 Patients may have received an unlimited number of prior therapy regimens.

3.153 Patients may not have received all of the five choices in the "standard therapy" arm.

3.154 Any hormonal therapy directed at the malignant tumor must be discontinued at least one week prior to registration

3.155 Any other prior therapy directed at the malignant tumor, including chemotherapy and radiation therapy, must be discontinued at least 4 weeks prior to registration. Any investigational agent must be discontinued at least 28 days prior to registration.

3.16 Trametinib, can cause fetal harm when administered to a pregnant woman. Women of child-bearing potential (i.e. patients whose reproductive organs remain in place and who have not passed menopause) and men must agree to use a highly effective method of contraception (e.g. hormonal, intrauterine device or; abstinence[1]) prior to study entry, during the study participation, and for six months after the last dose of the drug. Women of child-bearing potential must have a negative serum pregnancy test within 14 days prior to randomization, cannot be breast-feeding, and must agree to use a highly effective form of contraception throughout the treatment period and for 6 months after the last dose of study treatment. Should a woman become pregnant or suspect she is pregnant while she or her partner is participating in this study, she should inform her treating physician immediately.
([1]Abstinence is only acceptable when this is in line with the preferred and usual lifestyle of the patient.)
(07/28/14) (12/22/14)

3.17 Patients must have ability to understand and sign an approved informed consent and authorization permitting release of personal health information.

3.18 Patients must have a GOG Performance Status of 0 or 1.

3.19 Able to swallow and retain orally-administered medication and does not have any clinically significant gastrointestinal abnormalities that may alter absorption, such as malabsorption syndrome, bowel obstruction, or major resection of the stomach or bowel. (12/22/14)

3.110 All prior treatment-related toxicities must be CTCAE v4 grade ≤1 (**except alopecia**) at the time of randomization. (07/28/14)

3.111 Patients must have a left ventricular ejection fraction ≥ lower limit of normal by ECHO or MUGA.

3.112 Patients must have adequate renal, endocrine, and hepatic function and bone marrow reserve. (07/28/14) (12/22/14)

- Serum creatinine ≤1.5 mg/dL *OR* calculated creatinine clearance (Cockroft-Gault formula) ≥50 mL/min *OR* 24-hour urine creatinine clearance ≥50 mL/min
- Bilirubin ≤ 1.5 times upper limit of normal
- ALT ≤ 2.5 times upper limit of normal
- AST ≤ 2.5 times upper limit of normal
- Albumin ≥ 2.5 g/dL
- PT and APTT ≤ 1.5 times upper limit of normal
- Neutrophil count ≥ 1.5 x 10^9/L Platelet count ≥ 100 x 10^9/L
- Hemoglobin ≥ 9.0 g/dL

Samples must be taken within 14 days prior to treatment.

3.113 If letrozole is selected as the control therapy, patients must be postmenopausal, either following bilateral oophorectomy or at least 5 years after spontaneous menopause. Patients within 5 years of spontaneous menopause or who have had a hysterectomy without bilateral oophorectomy must have postmenopausal LH and FSH levels. Patients on HRT must agree to withdrawal of hormone therapy before letrozole is started.

3.2 Ineligible Patients

3.21 Patients who have had chemotherapy or radiotherapy within 4 weeks (6 weeks for nitrosoureas or mitomycin C) prior to entering the study or those who have not recovered from adverse events due to agents administered more than 4 weeks earlier.

3.22 Use of other investigational drugs within 28 days (or five half-lives, whichever is shorter; with a minimum of 14 days from the last dose) preceding the first dose of trametinib or standard of care agent. (07/28/14)

3.23 If patients have had a potential index lesion radiated, it must have progressed post radiation therapy to be used as a measurable eligibility lesion

3.24 Patients may not have received prior MEK, KRAS, or BRAF inhibitor therapy

3.25 Current use of a prohibited medication. The following medications or non-drug therapies are prohibited:

3.251 Patients may not be receiving any other anti-cancer or investigational agents.

3.252 Because the composition, PK, and metabolism of many herbal supplements are unknown, the concurrent use of all herbal supplements is prohibited during the study (including, but not limited to St. John's Wort, kava, ephedra [ma huang], gingko biloba, dehydroepiandrosterone [DHEA], yohimbe, saw palmetto, or ginseng).

3.26 Patients with known leptomeningeal or brain metastases or spinal cord compression should be excluded from this clinical trial because of their poor prognosis and because they often develop progressive neurologic dysfunction that would confound the evaluation of neurologic and other adverse events.

3.27 Patients with a bowel obstruction or any other gastrointestinal condition that might affect absorption of the oral drug should be excluded. This would include patients with inability to swallow and retain orally-administered medication, malabsorption syndrome, or those with a major resection of the stomach or bowels.

3.28 Patients with a history of interstitial lung disease or pneumonitis.

3.29 Patients with a previous or current malignancy at other sites should be excluded, with the exception of:

a. Curatively treated local tumors such as carcinoma-in-situ of the cervix, basal or squamous cell carcinoma of the skin
b. Tumors for which no relapse has been observed within 5 years

3.30 Known Hepatitis B Virus (HBV), or Hepatitis C Virus (HCV) infection (patients with chronic or cleared HBV and HCV infection are eligible). Patients with Human Immunodeficiency Virus (HIV) are not eligible if on anti-retroviral medications (07/28/14)

3.31 Known immediate or delayed hypersensitivity reaction or idiosyncrasy to drugs chemically related to Trametinib, or excipients, or to dimethyl sulfoxide (DMSO), or to Cremophor EL (polyoxyethylated castor oil). Please note, exclusion for Cremophor is unnecessary unless paclitaxel is the only agent available and the patient randomizes to the conventional therapy option. (07/28/14)

3.32 Patients with a history or evidence of cardiovascular risk, including any of the following:

- LVEF<LLN
- QTcB $\geq$ 480 msec.
- History or evidence of current clinically significant uncontrolled arrhythmias.

- Exception: Subjects with controlled atrial fibrillation for > 30 days prior to randomization are eligible.
- History of (within 6 months prior to randomization) acute coronary syndromes (including myocardial infarction and unstable angina), coronary angioplasty, or stenting.
- History or evidence of current $\geq$ Class II congestive heart failure as defined by New York Heart Association (NYHA).
- Treatment refractory hypertension defined as a blood pressure of systolic >140mmHg and/or diastolic >90mmHg which cannot be controlled by anti-hypertensive therapy
- Patients with intra-cardiac defibrillators or permanent pacemakers
- Known cardiac metastases

3.33 Patients with a history or current evidence/risk of retinal vein occlusion (RVO) (07/28/14)

3.34 Any serious and/or unstable pre-existing medical disorder (aside from malignancy exception above), psychiatric disorder, or other conditions that could interfere with subject's safety, obtaining informed consent or compliance to the study procedures. (07/28/14)

3.35 Patients who require use of a concomitant medication that can prolong the QT interval. See the table in section 6.28 (07/28/14)

3.36 Animal reproductive studies have not been conducted with trametinib. Therefore, the study drug must not be administered to pregnant women or nursing mothers. Women of childbearing potential should be advised to avoid pregnancy and use effective methods of contraception. If a female patient or a female partner of a patient becomes pregnant while the patient receives trametinib, the potential hazard to the fetus should be explained to the patient and partner (as applicable). (07/28/14)

4.0 STUDY MODALITIES

4.1 Letrozole (Femara) (NSC #719435)

4.11 Formulation: Tablets of 2.5 mg of Letrozole

4.12 Storage and Stability: Store between 59 degrees and 86 degrees F, protect from moisture; Very stable

4.13 Adverse Effects: Vascular disorders, flushing, cerebrovascular accident, hypertension, thromboembolic event, angina, skin rash, increased sweating, increased hepatic enzymes, arthralgia/arthritis. See package insert for complete list of adverse effects.

4.14 Supplier: Commercially available.

4.15 Administration: Letrozole 2.5 mg orally daily

4.2 Tamoxifen Citrate (Nolvadex®) (NSC #180973)

4.21 Formulation: Tablets of 10 and 20 mg of tamoxifen. The 20 mg tablets are to be used for this study.

4.22 Storage: Room temperature protected from heat and light.

4.23 Adverse Effects: Transient thrombocytopenia and leukopenia, menopause-like reactions (hot flushes, nausea), skin rashes/changes, pruritus vulvae, dizziness, headaches, depression, lassitude, muscle pain, fluid retention, anorexia, menstrual irregularities, vaginal bleeding, vaginal discharge, food distaste and thromboembolic events. In patients with breast carcinoma, a flare of symptoms related to known areas of disease and hypercalcemia in patients with bone metastases have been reported. Of note also is a report of a few cases of severe retinopathy associated with decreased visual acuity in patients on high doses (>200 mg/day) for prolonged periods over a year. Dose modifications or cessation of drug because of adverse effects is seldom necessary. See package insert for complete list of adverse effects.

4.24 Supplier: Commercially available.

4.25 Administration: See Section 5.21.

4.3 Pegylated Liposomal Doxorubicin (DOXIL®, NSC #712227, Lipodox™)

Refer to the PLD package insert (Doxil, Lipodox™) for the most complete and current information on the following:

4.31 Formulation: PLD (doxorubicin HCl liposome injection) is supplied as a sterile, translucent, red liposomal dispersion in 5 mL (Lipodox only), 10 mL, or 30 mL glass, single-use vials. Each vial contains doxorubicin HCl at a concentration of 2 mg/mL.

4.32 Storage: Refrigerate unopened vials of PLD at 2°–8°C (36°–46°F). Avoid freezing. Prolonged freezing may adversely affect liposomal drug products; however, short-term freezing (less than 1 month) does not appear to have a deleterious effect on PLD.

4.33 Preparation: PLD doses up to 90 mg must be diluted in 250 mL of 5% Dextrose Injection, USP prior to administration. Doses exceeding 90 mg should be diluted in 500 mL of 5% Dextrose Injection, USP prior to administration. Aseptic technique must be strictly observed since no preservative or bacteriostatic agent is present in PLD. Diluted PLD should be refrigerated at 2°C–8°C (36°F–46°F) and administered within 24 hours.

Do not mix with other drugs.

Do not use with any diluent other than 5% Dextrose Injection.

Do not use any bacteriostatic agent, such as benzyl alcohol.

Do not infuse with in-line filters.

PLD is not a clear solution but a translucent, red liposomal dispersion.

Parenteral drug products should be inspected visually for particulate matter and discoloration prior to administration, whenever solution and container permit. Do not use if a precipitate or foreign matter is present.

4.34 Procedure for Proper Handling and Disposal: Caution should be exercised in the handling and preparation of PLD.

The use of gloves is required.

If PLD comes into contact with skin or mucosa, immediately wash thoroughly with soap and water.

PLD should be considered an irritant and precautions should be taken to avoid extravasation. With intravenous administration of PLD, extravasation may occur with or without an accompanying stinging or burning sensation, even if blood returns well on aspiration of the infusion needle. If any signs or symptoms of extravasation have occurred, the infusion should be immediately terminated and restarted in another vein. PLD must not be given by the intramuscular or subcutaneous route. PLD should be handled and disposed of in a manner consistent with other anticancer drugs.

4.35 Adverse Effects: Consult the PLD package insert for the most current and complete information.

4.36 Supplier: Commercially available.

Consult the American Hospital Formulary Service Drug Information guide, Facts and Comparisons, or the package insert for additional information.

4.4 Topotecan (NSC #609699)

4.41 Formulation: Topotecan is a cell cycle specific inhibitor of the nuclear enzyme topoisomerase I. It has a mean half-life of approximately three hours. Topotecan' s metabolism and clearance are complex but it is estimated that approximately 40% of the drug undergoes renal clearance. It is supplied as a lyophilized, light-yellow powder as the base Topotecan AS with 48 mg mannitol and 20 mg tartaric acid, NF.

4.42 Supplier/How Supplied: Topotecan is commercially available. It is supplied in a sterile form for intravenous use only. Topotecan is supplied as a lyophilized, light-yellow powder in vials containing 4 mg Topotecan AS (as the base) and 48 mg mannitol and 20 mg tartaric acid, NF. It has a reverse magenta label for identification purposes.

4.43 Solution Preparation: The contents of each 4 mg vial will be reconstituted with 4 ml Sterile Water for Injection, USP, yielding a 1 mg/ml solution of Topotecan AS. The ph is adjusted to 3.0. The vials reconstituted with Sterile Water for Injection, USP, contain no antibacterial preservative and must be used within 24 hours.

The reconstituted solution will be further diluted in plastic bags to concentrations of 10 mcg/ml to 500 mcg/ml in Dextrose 5% in Water, or Normal Saline. The plastic bags for infusion are stable at room temperature for 24 hours if reconstituted with Dextrose 5% in Water or Normal Saline. No incompatibilities with other drugs have been described. The drug is stable in IV solutions as stated above.

4.44 Storage/Stability: Un-reconstituted vials are stored at room temperature, 15-30°C (59- 86°F). The reconstituted vials, when reconstituted with Sterile Water for Injection, USP, will contain no antibacterial preservative and should be used within 24 hours.

4.45 Adverse effects:
Hematologic: thrombocytopenia, leukopenia, anemia, neutropenia
Gastrointestinal: nausea and vomiting, mucositis, diarrhea
Skin: rash
Other: alopecia, fever, flu-like symptoms

*Refer to Package Insert for additional information.

4.5 Paclitaxel (NSC#673089)

4.51 Formulation: Paclitaxel is supplied as a 6mg/mL non-aqueous solution in multi dose vials containing 30mg/5mL, 100mg/16.7mL, or 300mg/50mL of paclitaxel. In addition to 6mg of paclitaxel, each mL of sterile non-pyrogenic solution contains 527mg of purified Cremophor® EL (polyoxyethylated castor oil) and 49.7% (v/v) dehydrated alcohol, USP.

4.52 Storage: Unopened vials of paclitaxel are stable to the date indicated on the package when stored between 20 to 25°C (68 to 77°F). Protect from light.

4.53 Preparation: Paclitaxel must be diluted prior to infusion. Paclitaxel should be diluted in 0.9% Sodium Chloride for Injection, USP; 5% Dextrose Injection, USP; 5% Dextrose and 0.9% Sodium Chloride Injection, USP; or 5% Dextrose in Ringer's Injection to a final concentration of 0.3 to 1.2mg/mL. The solutions are physically and chemically stable for up to 27 hours at ambient temperature (approximately 25°C / 77°F) and room lighting conditions.

NOTE: In order to minimize patient exposure to the plasticizer DEHP, which may be leached from PVC infusion bags or sets, diluted paclitaxel solutions should be stored in bottles (glass, polypropylene) or plastic (polypropylene, polyolefin) bags and administered through polyethylene-lined administration sets. Paclitaxel should be administered through an inline filter with a microporous membrane not greater than 0.22 microns. Use of filter devices such as IVEX- 2® or IVEX-HP®, which incorporate short inlet and outlet PVC-coated tubing, has not resulted in significant leaching of DEHP.

All patients should be premedicated with corticosteroids, diphenhydramine, and H2 antagonists prior to paclitaxel administration in order to prevent severe hypersensitivity reactions. Patients who experience severe hypersensitivity reactions to paclitaxel should not be re-challenged with the drug.

4.54 Adverse Effects: Consult the package insert for the most current and complete information.

4.55 Supplier: Commercially available. Consult the American Hospital Formulary Service Drug Information guide, Facts and Comparisons, or the package insert for additional information.

4.6 Trametinib dimethyl sulfoxide (GSK1120212B) (NSC 763093) (06/08/15)(07/24/2020)

4.61 Chemical Name (IUPAC): equimolecular combination of N-(3-{3-cyclopropyl-5-[(2-fluoro-4-iodophenyl)amino]-6,8-dimethyl-2,4,7-trioxo-

3,4,6,7-tetrahydropyrido[4,3-d]pyrimidin-1(2H)-yl}phenyl)acetamide with (methylsulfinyl)methane

Other Names: trametinib, GSK1120212B, TMT212-NXA, JTP-74057, JTP-78296, JTP-75303, Mekinist®

CAS Registry Number: 1187431-43-1

Classification: MEK inhibitor

Molecular Formula: $C_{26}H_{23}FIN_5O_4 \cdot C_2H_6OS$

M.W.: 693.53 (DMSO solvate form)

Approximate Solubility: Trametinib dimethyl sulfoxide is almost insoluble in aqueous media at pH range of 2-8.

4.62 Mode of Action: Trametinib dimethyl sulfoxide is a reversible, highly selective, allosteric inhibitor of mitogen-activated extracellular signal regulated kinase 1 (MEK1) and MEK2. Tumor cells commonly have hyperactivated extracellular signal-related kinase (ERK) pathways in which MEK is a critical component. Trametinib dimethyl sulfoxide inhibits activation of MEK by RAF kinases and MEK kinases.

4.63 Description: Trametinib dimethyl sulfoxide is a white to almost white powder.

4.64 **How Supplied:** Novartis supplies and CTEP, NCI, DCTD distributes trametinib as 0.5 mg and 2 mg (as free base) tablets (05/31/16). Each investigationally-labeled bottle contains 32 tablets with a desiccant.

The tablet core contains mannitol, microcrystalline cellulose, hypromellose, croscarmellose sodium, magnesium stearate (non-animal), colloidal silicon dioxide and sodium lauryl sulfate.

- 0.5 mg tablets are yellow, modified oval, biconvex and film-coated. Aqueous film coating consists of hypromellose, titanium dioxide, polyethylene glycol, iron oxide yellow.
- 2 mg tablets are pink, round, biconvex and film-coated. Aqueous film coating consists of hypromellose, titanium dioxide, polyethylene glycol, polysorbate 80, iron oxide red.

4.65 Storage: Store tablets at 2°C -8°C (36° F to 46° F) in the original bottle and dispense unopened bottles. Do not open bottles or repackage tablets or remove desiccant. Bottles should be protected from light and moisture.

If a storage temperature excursion is identified, promptly return trametinib to 2°C -8°C and quarantine the supplies. Provide a detailed report of the

excursion (including documentation of temperature monitoring and duration of the excursion) to PMBAfterHours@mail.nih.gov for determination of suitability.

4.66 Stability: Stability studies are ongoing. Tablets are only stable for 32 days once bottle has been opened. If multiple bottles are dispensed to a patient in the same visit, please advise the patient to open only one bottle at a time.

4.67 Route of Administration: Oral. Take by mouth on an empty stomach, either 1 hour before or 2 hours after a meal. If a dose of trametinib is missed, the dose can be taken if it is more than 12 hours until the next scheduled dose.

4.68 Adverse Effects: Please refer to section 4.612 for a full list of adverse effects.

4.69 Potential Drug Interactions: *In vitro* studies suggest that trametinib dimethyl sulfoxide is not a substrate of CYP enzymes or of human BCRP, MRP2, OATP1B1, OATP1B3, OATP2B1, OCT1 or MATE1 transporters. Trametinib elimination by deacetylation to metabolite M5 is dependent on carboxylesterases (CES1b, CES1c and CES2). Trametinib is a substrate for P-gp and BSEP, but this is not expected to be clinically relevant due to trametinib's high permeability.

Trametinib is an *in vitro* inhibitor of CYP 2C8 and is anticipated to have overall low potential for drug interactions as a perpetrator. It is also a weak CYP 2B6 and 3A4 inducer and expected to have little clinical effect on sensitive substrates. Trametinib is not an inhibitor of CYP 1A2, 2A6, 2B6, 2C9, 2C19, 2D6 and 3A4 and not an inhibitor of MRP2 or BSEP, but an in vitro inhibitor of P-gp, BCRP, OATP1B1, OATP1B3, OAT1, OAT3, OCT2 and MATE1 at systemic concentrations that are not clinically relevant. No clinically relevant inhibition by trametinib is predicted in the liver or kidney and a low risk of intestinal drug-drug interaction is possible with BCRP.

Trametinib is highly bound to plasma proteins (97.3%) and has the potential to interfere with other highly protein-bound drugs. Use caution in patients taking concomitant drugs that are highly protein-bound and have narrow therapeutic ranges.

4.610 Patient Care Implications: Advise women study participants of reproductive potential to use effective contraception while receiving study treatment and for 4 months after the last dose of trametinib. Advise women not to breastfeed while receiving study treatment and for 4 months after the last dose of trametinib. Advise men study participants to use barrier contraception and not to father a child while taking study treatment and for 4 months after the last dose of trametinib.

4.611 Availability: Trametinib dimethyl sulfoxide (GSK1120212B) is an investigational agent supplied to investigators by the Division of Cancer Treatment and Diagnosis (DCTD), NCI.

Trametinib dimethyl sulfoxide (GSK1120212B) is provided to the NCI under a Collaborative Agreement between the Pharmaceutical Collaborator and the DCTD, NCI.

4.612 Agent Ordering and Agent Accountability

NCI-supplied agents may be requested by the Principal Investigator (or their authorized designee) at each participating institution. Pharmaceutical Management Branch (PMB) policy requires that agent be shipped directly to the institution where the patient is to be treated. PMB does not permit the transfer of agents between institutions (unless prior approval from PMB is obtained). The CTEP-assigned protocol number must be used for ordering all CTEP-supplied investigational agents. The responsible investigator at each participating institution must be registered with CTEP, DCTD through an annual submission of FDA Form 1572 (Statement of Investigator), Curriculum Vitae, Supplemental Investigator Data Form (IDF), and Financial Disclosure Form (FDF). If there are several participating investigators at one institution, CTEP-supplied investigational agents for the study should be ordered under the name of one lead investigator at that institution.

Active CTEP-registered investigators and investigator-designated shipping designees and ordering designees can submit agent requests through the PMB Online Agent Order Processing (OAOP) application (https://eapps-ctep.nci.nih.gov/OAOP/pages/login.jspx). Access to OAOP requires the establishment of a CTEP Identity and Access Management (IAM) account (https://eapps-ctep.nci.nih.gov/iam/) and the maintenance of an "active" account status and a "current" password. For questions about drug orders, transfers, returns, or accountability, call (240) 276-6575 Monday through Friday between 8:30 am and 4:30 pm (ET) or email PMBAfterHours@mail.nih.gov anytime.

4.612 Comprehensive Adverse Events and Potential Risks list (CAEPR) for Trametinib dimethyl sulfoxide (GSK1120212B, NSC 763093)

The Comprehensive Adverse Events and Potential Risks list (CAEPR) provides a single list of reported and/or potential adverse events (AE) associated with an agent using a uniform presentation of events by body system. In addition to the comprehensive list, a subset, the Specific Protocol Exceptions to Expedited Reporting (SPEER), appears in a separate column and is identified with bold and italicized text. This subset of AEs (SPEER) is a list of events that are protocol specific exceptions to expedited reporting to NCI (except as noted below). Refer to the

'CTEP, NCI Guidelines: Adverse Event Reporting Requirements' http://ctep.cancer.gov/protocolDevelopment/electronic_applications/docs/aeguidelines.pdf for further clarification. *Frequency is provided based on 1111 patients.* Below is the CAEPR for Trametinib (GSK1120212B).

NOTE: Report AEs on the SPEER **ONLY IF** they exceed the grade noted in parentheses next to the AE in the SPEER. If this CAEPR is part of a combination protocol using multiple investigational agents and has an AE listed on different SPEERs, use the lower of the grades to determine if expedited reporting is required.

Version 2.6, October 10, 2019[1]

**Adverse Events with Possible Relationship to Trametinib (GSK1120212B) (CTCAE 5.0 Term) [n= 1111]**			**Specific Protocol Exceptions to Expedited Reporting (SPEER)**
**Likely (>20%)**	**Less Likely (<=20%)**	**Rare but Serious (<3%)**	
BLOOD AND LYMPHATIC SYSTEM DISORDERS			
	Anemia		***Anemia (Gr 3)***
CARDIAC DISORDERS			
		Heart failure	
		Left ventricular systolic dysfunction	
	Sinus bradycardia		
EYE DISORDERS			
	Blurred vision		
	Dry eye		
		Eye disorders - Other (chorioretinopathy also known as retinal pigment epithelial detachment)	
		Eye disorders - Other (retinal vein occlusion)	
	Eye disorders - Other (visual disorders)[2]		
		Papilledema	
	Periorbital edema		
GASTROINTESTINAL DISORDERS			
	Abdominal pain		***Abdominal pain (Gr 2)***
		Colitis	
		Colonic perforation	
	Constipation		***Constipation (Gr 2)***
Diarrhea			***Diarrhea (Gr 3)***
	Dry mouth		***Dry mouth (Gr 2)***
	Dyspepsia		***Dyspepsia (Gr 2)***
	Mucositis oral		***Mucositis oral (Gr 3)***
Nausea			***Nausea (Gr 3)***
	Vomiting		***Vomiting (Gr 3)***
GENERAL DISORDERS AND ADMINISTRATION SITE CONDITIONS			
	Chills		***Chills (Gr 2)***
	Edema face		
Fatigue			***Fatigue (Gr 3)***
	Fever		***Fever (Gr 2)***
Generalized edema[3]			***Generalized edema[3] (Gr 2)***
IMMUNE SYSTEM DISORDERS			
	Allergic reaction[4]		
INFECTIONS AND INFESTATIONS			

Adverse Events with Possible Relationship to Trametinib (GSK1120212B) (CTCAE 5.0 Term) [n= 1111]			Specific Protocol Exceptions to Expedited Reporting (SPEER)
**Likely (>20%)**	**Less Likely (<=20%)**	**Rare but Serious (<3%)**	
	Folliculitis		***Folliculitis (Gr 2)***
	Lung infection		
	Paronychia		***Paronychia (Gr 2)***
	Skin infection		***Skin infection (Gr 2)***
INVESTIGATIONS			
	Alanine aminotransferase increased		***Alanine aminotransferase increased (Gr 3)***
	Alkaline phosphatase increased		***Alkaline phosphatase increased (Gr 2)***
	Aspartate aminotransferase increased		***Aspartate aminotransferase increased (Gr 3)***
	CPK increased		
	Ejection fraction decreased		
METABOLISM AND NUTRITION DISORDERS			
	Anorexia		***Anorexia (Gr 3)***
	Dehydration		***Dehydration (Gr 3)***
	Hypoalbuminemia		
	Hypomagnesemia		***Hypomagnesemia (Gr 2)***
	Hyponatremia		***Hyponatremia (Gr 3)***
MUSCULOSKELETAL AND CONNECTIVE TISSUE DISORDERS			
	Arthralgia		
	Back pain		***Back pain (Gr 2)***
	Pain in extremity		***Pain in extremity (Gr 2)***
		Rhabdomyolysis	
NERVOUS SYSTEM DISORDERS			
	Dizziness		***Dizziness (Gr 2)***
	Headache		***Headache (Gr 2)***
RESPIRATORY, THORACIC AND MEDIASTINAL DISORDERS			
	Cough		***Cough (Gr 2)***
	Dyspnea		***Dyspnea (Gr 3)***
		Pneumonitis	
SKIN AND SUBCUTANEOUS TISSUE DISORDERS			
	Alopecia		***Alopecia (Gr 2)***
	Dry skin		***Dry skin (Gr 2)***
	Nail changes		
		Palmar-plantar erythrodysesthesia syndrome	
	Pruritus		***Pruritus (Gr 2)***
		Skin and subcutaneous tissue disorders - Other (drug reaction with eosinophilia and systemic symptoms [DRESS])	
Skin and subcutaneous tissue disorders - Other (rash)[5]			***Skin and subcutaneous tissue disorders - Other (rash)[5] (Gr 3)***
		Stevens-Johnson syndrome[6]	
VASCULAR DISORDERS			
	Hypertension		***Hypertension (Gr 3)***
		Thromboembolic event (venous)	

Adverse Events with Possible Relationship to Trametinib (GSK1120212B) (CTCAE 5.0 Term) [n= 1111]			Specific Protocol Exceptions to Expedited Reporting (SPEER)
**Likely (>20%)**	**Less Likely (<=20%)**	**Rare but Serious (<3%)**	
	Vascular disorders - Other (hemorrhage)[7]		

[1]This table will be updated as the toxicity profile of the agent is revised. Updates will be distributed to all Principal Investigators at the time of revision. The current version can be obtained by contacting PIO@CTEP.NCI.NIH.GOV. Your name, the name of the investigator, the protocol and the agent should be included in the e-mail.

[2]Visual disorders include visual disturbance that can be associated with conjunctival hemorrhage, corneal graft rejection, cyclitis, eye nevus, halo vision, iritis, macular edema, retinal hemorrhage, visual acuity reduced, visual impairment, and vitreous detachment.

[3]Generalized edema includes edema, lymphedema, and edema limbs.

[4]Hypersensitivity (allergic reactions) may present with symptoms such as fever, rash, increased liver function tests, and visual disturbances.

[5]Skin and subcutaneous tissue disorders - Other (rash) may include rash, rosacea, rash acneiform, erythematous rash, genital rash, rash macular, exfoliative rash, rash generalized, erythema, rash papular, seborrhoeic dermatitis, dermatitis psoriasiform, rash follicular, skin fissures, and skin chapped.

[6]Stevens-Johnson syndrome has been observed in patients treated with trametinib and dabrafenib combination.

[7]The majority of hemorrhage events were mild. Major events, defined as symptomatic bleeding in a critical area or organ (e.g., eye, GI hemorrhage, GU hemorrhage, respiratory hemorrhage), and fatal intracranial hemorrhages have been reported.

Adverse events reported on trametinib dimethyl sulfoxide (GSK1120212B) trials, but for which there is insufficient evidence to suggest that there was a reasonable possibility that trametinib dimethyl sulfoxide (GSK1120212B) caused the adverse event:

BLOOD AND LYMPHATIC SYSTEM DISORDERS - Disseminated intravascular coagulation; Febrile neutropenia; Leukocytosis
CARDIAC DISORDERS - Atrial fibrillation; Cardiac arrest; Myocardial infarction; Restrictive cardiomyopathy; Sinus tachycardia
EYE DISORDERS - Corneal ulcer; Eyelid function disorder; Flashing lights; Floaters; Glaucoma; Photophobia
GASTROINTESTINAL DISORDERS - Ascites; Duodenal ulcer; Esophageal necrosis; Esophageal ulcer; Esophagitis; Gastric hemorrhage[7]; Gastric ulcer; Gastritis; Gastrointestinal disorders - Other (intestinal obstruction); Gastrointestinal disorders - Other (pneumatosis intestinalis); Gastrointestinal fistula; Gingival pain; Hemorrhoidal hemorrhage[7]; Ileus; Obstruction gastric; Pancreatitis; Small intestinal obstruction
GENERAL DISORDERS AND ADMINISTRATION SITE CONDITIONS - Flu like symptoms; General disorders and administration site conditions - Other (axillary pain); Localized edema; Malaise; Non-cardiac chest pain; Pain
HEPATOBILIARY DISORDERS - Cholecystitis; Hepatic failure; Hepatic pain; Hepatobiliary disorders - Other (hepatic encephalopathy)

INFECTIONS AND INFESTATIONS - Biliary tract infection; Catheter related infection; Device related infection; Endocarditis infective; Enterocolitis infectious; Hepatitis viral; Infections and infestations - Other (abscess limb); Infections and infestations - Other (necrotizing fasciitis); Infections and infestations - Other (oral infection); Pharyngitis; Sepsis; Upper respiratory infection; Urinary tract infection
INJURY, POISONING AND PROCEDURAL COMPLICATIONS - Bruising
INVESTIGATIONS - Blood bilirubin increased; Blood lactate dehydrogenase increased; Creatinine increased; Electrocardiogram QT corrected interval prolonged; GGT increased; Lipase increased; Lymphocyte count decreased; Platelet count decreased; Serum amylase increased; White blood cell decreased
METABOLISM AND NUTRITION DISORDERS - Hyperglycemia; Hyperkalemia; Hyperphosphatemia; Hyperuricemia; Hypocalcemia; Hypoglycemia; Hypokalemia
MUSCULOSKELETAL AND CONNECTIVE TISSUE DISORDERS - Generalized muscle weakness; Muscle cramp; Musculoskeletal and connective tissue disorder - Other (compression fracture); Myalgia; Neck pain
NEOPLASMS BENIGN, MALIGNANT AND UNSPECIFIED (INCL CYSTS AND POLYPS) - Tumor hemorrhage[7]; Tumor pain
NERVOUS SYSTEM DISORDERS - Dysgeusia; Encephalopathy; Intracranial hemorrhage[7]; Lethargy; Nervous system disorders - Other (diplopia); Seizure; Somnolence; Stroke; Syncope; Transient ischemic attacks
PSYCHIATRIC DISORDERS - Anxiety; Confusion; Delirium; Depression; Hallucinations; Insomnia; Personality change
RENAL AND URINARY DISORDERS - Acute kidney injury; Cystitis noninfective; Dysuria; Hematuria; Proteinuria; Urinary incontinence
REPRODUCTIVE SYSTEM AND BREAST DISORDERS - Vaginal fistula; Vaginal hemorrhage[7]
RESPIRATORY, THORACIC AND MEDIASTINAL DISORDERS - Bronchopulmonary hemorrhage[7]; Hypoxia; Laryngeal edema; Oropharyngeal pain; Pleural effusion; Pneumothorax; Productive cough; Pulmonary hypertension; Respiratory failure; Sinus disorder
SKIN AND SUBCUTANEOUS TISSUE DISORDERS - Bullous dermatitis; Photosensitivity; Purpura; Skin and subcutaneous tissue disorders - Other (erythema nodosum); Skin ulceration; Urticaria
VASCULAR DISORDERS - Hematoma; Hot flashes; Hypotension

Note: Trametinib (GSK1120212B) in combination with other agents could cause an exacerbation of any adverse event currently known to be caused by the other agent, or the combination may result in events never previously associated with either agent.

5.0 TREATMENT PLAN AND ENTRY/RANDOMIZATION PROCEDURE **(02-JUN-2020)**

5.1 Investigator and Research Associate Registration with CTEP (07/28/14)

Food and Drug Administration (FDA) regulations and National Cancer Institute (NCI) policy require all individuals contributing to NCI-sponsored trials to register and renew their registration annually. To register, all individuals must obtain a Cancer Therapy Evaluation Program (CTEP) Identity and Access Management (IAM) account at https://ctepcore.nci.nih.gov/iam. In addition, persons with a registration type of Investigator (IVR), Non-Physician Investigator (NPIVR), or Associate Plus (AP) must complete their annual registration using CTEP's web-based Registration and Credential Repository (RCR) at https://ctepcore.nci.nih.gov/rcr.

RCR utilizes five person registration types.

- IVR — MD, DO, or international equivalent;

- NPIVR — advanced practice providers (e.g., NP or PA) or graduate level researchers (e.g., PhD);
- AP — clinical site staff (e.g., RN or CRA) with data entry access to CTSU applications such as the Roster Update Management System [RUMS], OPEN, Rave, acting as a primary site contact, or with consenting privileges;
- Associate (A) — other clinical site staff involved in the conduct of NCI-sponsored trials; and
- Associate Basic (AB) — individuals (e.g., pharmaceutical company employees) with limited access to NCI-supported systems.

RCR requires the following registration documents:

Documentation Required	IVR	NPIVR	AP	A	AB
FDA Form 1572	✔	✔			
Financial Disclosure Form	✔	✔	✔		
NCI Biosketch (education, training, employment, license, and certification)	✔	✔	✔		
GCP training	✔	✔	✔		
Agent Shipment Form (if applicable)	✔				
CV (optional)	✔	✔	✔		

An active CTEP-IAM user account and appropriate RCR registration is required to access all CTEP and Cancer Trials Support Unit (CTSU) websites and applications. In addition, IVRs and NPIVRs must list all clinical practice sites and Institutional Review Boards (IRBs) covering their practice sites on the FDA Form 1572 in RCR to allow the following:

- Addition to a site roster;
- Assign the treating, credit, consenting, or drug shipment (IVR only) tasks in OPEN;
- Act as the site-protocol Principal Investigator (PI) on the IRB approval; and
- Assign the Clinical Investigator (CI) role on the Delegation of Tasks Log (DTL).

In addition, all investigators acting as the Site-Protocol PI (investigator listed on the IRB approval), consenting/treating/drug shipment investigator in OPEN, or as the CI on the DTL must be rostered at the enrolling site with a participating organization.

Additional information is located on the CTEP website at https://ctep.cancer.gov/investigatorResources/default.htm. For questions, please contact the **RCR Help Desk** by email at RCRHelpDesk@nih.gov.

5.11 CTSU Registration Procedures (03/21/16)

Permission to view and download this protocol and its supporting documents is restricted and is based on person and site roster assignment housed in the CTSU Regulatory Support System (RSS).

This study is supported by the NCI Cancer Trials Support Unit (CTSU).

5.111 IRB Approval:

For CTEP and Division of Cancer Prevention (DCP) studies open to the National Clinical Trials Network (NCTN) and NCI Community Oncology Research Program (NCORP) Research Bases after March 1, 2019, all U.S.-based sites must be members of the NCI Central Institutional Review Board (NCI CIRB). In addition, U.S.-based sites must accept the NCI CIRB review to activate new studies at the site after March 1, 2019. Local IRB review will continue to be accepted for studies that are not reviewed by the CIRB, or if the study was previously open at the site under the local IRB. International sites should continue to submit Research Ethics Board (REB) approval to the CTSU Regulatory Office following country-specific regulations.

Sites participating with the NCI CIRB must submit the Study Specific Worksheet for Local Context (SSW) to the CIRB using IRBManager to indicate their intent to open the study locally. The NCI CIRB's approval of the SSW is automatically communicated to the CTSU Regulatory Office, but sites are required to contact the CTSU Regulatory Office at CTSURegPref@ctsu.coccg.org to establish site preferences for applying NCI CIRB approvals across their Signatory Network. Site preferences can be set at the network or protocol level. Questions about establishing site preferences can be addressed to the CTSU Regulatory Office by email or calling 1-888-651-CTSU (2878).

5.112 Downloading Site Registration Documents:

Download the site registration forms from the protocol-specific page located on the CTSU members' website. Permission to view and download this protocol and its supporting documents is restricted based on person and site roster assignment. To participate, the institution and its associated investigators and staff must be associated with the LPO or a Protocol Organization (PO) on the protocol. One way to search for a protocol is listed below.

- Log in to the CTSU members' website (https://www.ctsu.org) using your CTEP-IAM username and password;
- Click on *Protocols* in the upper left of the screen
  - Enter the protocol number in the search field at the top of the protocol tree; or
  - Click on the By Lead Organization folder to expand, then select *[Corresponding Organization]*, and protocol number *[NCI Protocol #]*.

Click on *Documents*, select *Site Registration*, and download and complete the forms provided. (Note: For sites under the CIRB, IRB data will load automatically to the CTSU.)

5.113 Submitting Regulatory Documents:

Submit required forms and documents to the CTSU Regulatory Office using the Regulatory Submission Portal on the CTSU website.

To access the Regulatory Submission Portal log in to the CTSU members' website, go to the Regulatory section and select Regulatory Submission.

Institutions with patients waiting that are unable to use the Regulatory Submission Portal should alert the CTSU Regulatory Office immediately at 1-866-651-2878 in order to receive further instruction and support.

5.114 Checking Your Site's Registration Status:

Site registration status may be verified on the CTSU members' website.

- Click on *Regulatory* at the top of the screen;
- Click on *Site Registration; and*
- Enter the sites 5-character CTEP Institution Code and click on Go.
  - Additional filters are available to sort by Protocol, Registration Status, Protocol Status, and/or IRB Type.

Note: The status shown only reflects institutional compliance with site registration requirements as outlined within the protocol. It does not reflect compliance with protocol requirements for individuals participating on the protocol or the enrolling investigator's status with NCI or their affiliated networks.

5.2 Patient Screening (07/28/14) (06/08/15)

When a suitable candidate has been obtained for protocol entry, the following steps should be taken:

<u>Screening</u>

When a suitable candidate has been identified for protocol entry, the following steps should be taken:

- An approved informed consent form and authorization permitting the release of personal health information must be signed by the patient or guardian.

Current FDA, NCI and institutional regulations concerning informed consent will be followed.

- All eligibility requirements indicated in Section 3.0 must be satisfied.

- Log onto the GOG website (http://www.gog.org), go to the Web Menu page and open the link to Screening to obtain a Screening Patient Identifier.

- The patient must have histologically documented low grade serous carcinoma confirmed by prospective review. An H&E stained slide from the primary tumor or tumor recurrence specimen may be submitted to meet histologic eligibility requirements (see section 7.21), label the slide with the Screening ID, and send the slide(s) along with a copy of the patient's pathology report to the GOG Tissue Bank in Columbus, OH.

  GOG-0281 Pathology Review
  Nationwide Children's Hospital
  700 Children's Drive, WA 1340
  Columbus, OH 43205
  614-722-2865

- Receive the results of the central pathology review on the Screening web application.

- If the patient is eligible, proceed to patient registration in GOG-0281.

5.3 Patient Entry and Registration (07/28/14)

The Oncology Patient Enrollment Network (OPEN) is a web-based registration system available on a 24/7 basis. OPEN is integrated with CTSU regulatory and roster data and with the LPOs registration/randomization systems or the Theradex Interactive Web Response System (IWRS) for retrieval of patient registration/randomization assignment. OPEN will populate the patient enrollment data in NCI's clinical data management system, Medidata Rave.

Requirements for OPEN access:

- A valid CTEP-IAM account;
- To perform enrollments or request slot reservations: Must be on an LPO roster, ETCTN corresponding roster, or participating organization roster

with the role of Registrar. Registrars must hold a minimum of an Associate Plus (AP) registration type;

- If a Delegation of Tasks Log (DTL) is required for the study, the registrars must hold the OPEN Registrar task on the DTL for the site; and
- Have an approved site registration for the protocol prior to patient enrollment.

To assign an Investigator (IVR) or Non-Physician Investigator (NPIVR) as the treating, crediting, consenting, drug shipment (IVR only), or receiving investigator for a patient transfer in OPEN, the IVR or NPIVR must list the IRB number used on the site's IRB approval on their Form FDA 1572 in RCR. If a DTL is required for the study, the IVR or NPIVR must be assigned the appropriate OPEN-related tasks on the DTL.

Prior to accessing OPEN, site staff should verify the following:

- Patient has met all eligibility criteria within the protocol stated timeframes; and
- All patients have signed an appropriate consent form and HIPAA authorization form (if applicable).

Note: The OPEN system will provide the site with a printable confirmation of registration and treatment information. You may print this confirmation for your records.

Access OPEN at https://open.ctsu.org or from the OPEN link on the CTSU members' website. Further instructional information is in the OPEN section of the CTSU website at https://www.ctsu.org or https://open.ctsu.org. For any additional questions, contact the CTSU Help Desk at 1-888-823-5923 or ctsucontact@westat.com.

5.4 Treatment Plan

For each arm, one cycle is 28 days. The first dose of study medication should be administered as close as possible to the time of randomization as the site allows.

5.41 Arm A

Clinician's choice of 'control' arm is made from the list below prior to randomization:

- Letrozole 2.5mg orally once daily continuous treatment until progression or unacceptable toxicity
- Tamoxifen 20mg orally twice daily continuous treatment until progression or unacceptable toxicity
- Paclitaxel 80mg/m^2 IV infusion over one hour on days 1, 8, and 15 of a 28 day cycle until progression or unacceptable toxicity or until 6 cycles have been administered*

- Pegylated Liposomal Doxorubicin 40 or 50mg/m^2 IV infusion over one hour on day 1 every 28 days until progression or unacceptable toxicity or until 6 cycles have been administered*
- Topotecan 4.0 mg/m^2 IV infusion over 30 minutes on days 1, 8, and 15 of a 28 day cycle until progression or unacceptable toxicity or until 6 cycles have been administered*

*****more than 6 cycles of chemotherapy can be administered at investigator's discretion.

5.42 Arm B

Trametinib 2 mg orally once daily continuous treatment until progression or unacceptable toxicity

5.43 Premedication
For Arm A, premedication is given as per local policy.

No premedication is required for Arm B.

5.44 Patient Monitoring
For Arm A, monitoring is performed as per local policy

No specific monitoring is required for Arm B.

5.45 Treatment Compliance
Compliance with oral medications on both arms will be assessed through querying the subject during the site visits and documented in the source documents and CRF.A record of the number of tablets dispensed to and taken by each subject must be maintained and reconciled with study treatment and compliance records. Treatment start and stop dates, including dates for treatment delays and/or dose reductions will also be recorded in the CRF.

5.46 Crossover (03/21/16)
If a patient develops progressive disease (**<u>as defined in Section 8.134</u>**) on Arm A, she will be given the opportunity to crossover to the Trametinib arm. **No PK specimens will be collected on crossover patients. (12/22/14)**

Prior to crossover, the following must occur:

- The patient's progression must be documented on the CRFs, which must be submitted prior to starting crossover treatment and will be

reviewed to confirm progression (Retrospective review of the scans may be done to examine this.)
All eligibility criteria in section 3 must be met (with the exception of 3.143, 3.151, and 3.113). This includes the requirement 3.21 such that 4 weeks must elapse between the end of treatment with Arm A and start of treatment on Arm B. These requirements will be documented on the CRFs. If the patient meets the above requirements (with appropriate documentation on the CRFs), she will be allowed to continue on study.

6.0 TREATMENT MODIFICATIONS

Doses will be reduced for hematological and other adverse events. Dose adjustments are to be made according to the greatest degree of toxicity. Adverse events will be graded using the NCI CTCAE v4.0.

6.1 Control Treatments

Dose adjustments as per standard of care

6.2 Trametinib

The severity of adverse events will be graded utilizing the CTCAE v 4.0. Guidelines for dose modifications and interruptions for management of common toxicities associated with the study treatment are provided in this section.

The guidelines that follow outline dose adjustments for several of these toxic effects. If a patient experiences several adverse events and there are conflicting recommendations, use the recommended dose adjustment that reduces the dose to the lowest level.

The table below outlines the dose levels to be used for any necessary trametinib dose modifications:

Dose Level	Trametinib Dose/Schedule
0	2 mg QD
-1	1.5 mg QD
-2	1 mg QD

A maximum of two trametinib dose level reductions are allowed. If a third dose level reduction is required, treatment will be permanently discontinued.

6.21 Trametinib Dose Modification for Toxicities Not Specified in Subsequent Sections

**Trametinib Treatment Modification for Clinically Significant Toxicities Deemed Related to Trametinib**<br>(This section is not for specific AEs such as hypertension, rash, ejection fraction changes, pneumonitis, diarrhea, liver chemistry, QTc prolongation, or visual changes. Refer to other sections for these specific AEs).		
**CTCAE v4 Grade**	**Management Guideline**	**Dose Modification**
Grade 1	Monitor as clinically indicated.<br>Provide supportive care	Continue trametinib at current dose level.
Grade 2 (tolerable)		• Interrupt treatment until resolution to grade 1 or baseline.<br>• Upon resolution, restart treatment at current dose level.

Trametinib Treatment Modification for Clinically Significant Toxicities Deemed Related to Trametinib (This section is not for specific AEs such as hypertension, rash, ejection fraction changes, pneumonitis, diarrhea, liver chemistry, QTc prolongation, or visual changes. Refer to other sections for these specific AEs).		
**CTCAE v4 Grade**	**Management Guideline**	**Dose Modification**
Grade 2 (intolerable)/ Grade 3	according to institutional standards	• Interrupt treatment until resolution to grade 1 or baseline.<br>• Upon resolution to baseline or grade 1, restart with one level of dose reduction<br>• If the Grade 3 toxicity recurs, interrupt trametinib; When toxicity resolves to Grade 1 or baseline, restart trametinib **reduced by another dose level**
Grade 4		Permanently discontinue trametinib.
1. Trametinib should be discontinued if treatment delay is ≥21 days due to toxicities. If the investigator concludes that continued trametinib will benefit a patient, the study chair and CTEP Medical Monitor may be consulted for the possibility of resuming trametinib, provided that toxicities have resolved to baseline or grade 1.<br>2. CTEP Medical Monitor:<br>Helen Chen, MD<br>240-276-6565<br>Helen.chen@nih.gov		

6.22 Trametinib Dose Modification for Rash

Rash is a frequent AE observed in patients receiving trametinib (Investigator's Brochure, 2012a). Recommendations for supportive care and guidelines for dose modifications for rash are based on experience with other MEK inhibitors and EGFR inhibitors (Balagula *et al.*, 2010; Lacouture *et al.*, 2011).

The institutional standards for the management of skin-related AEs can differ from these guidelines. In this case, best clinical judgment should be applied and a consultation with the study chair or the CTEP Medical Monitor may be required.

Guidelines for Supportive Care of Rash	
**Type of Care**	**Action**
**Prevention/Prophylaxis**[a]	• Avoid unnecessary exposure to sunlight.<br>• Apply broad-spectrum sunscreen (containing titanium dioxide or zinc oxide) with a skin protection factor (SPF) ≥15 at least twice daily.<br>• Use thick, alcohol-free emollient cream (*e.g.,* glycerine and cetomacrogol cream) on dry areas of the body at least twice daily.<br>• Topical steroids and antibiotics should be applied at least twice daily, starting on Day 1 of study treatment, to body areas such as face, chest, and upper back.<br>• Use mild-strength topical steroid (hydrocortisone 1% cream) or topical antibiotic (*e.g.*, clindamycin) or oral antibiotics (*e.g.*, doxycycline 100 mg BID, minocycline 100 mg BID).

Guidelines for Supportive Care of Rash	
**Type of Care**	**Action**
**Symptomatic Care**[b]	• Pruritic lesions: Cool compresses and oral antihistamine therapies.<br>• Fissuring lesions: Monsel's solution, silver nitrate, or zinc oxide cream.<br>• Desquamation: Thick emollients and mild soap.<br>• Paronychia: Antiseptic bath, local potent corticosteroids in addition to antibiotics; if no improvement, consult dermatologist or surgeon.<br>• Infected lesions: Appropriate bacterial/fungal culture-driven systemic or topical antibiotics.
[a] Rash prophylaxis is recommended for the first 6 weeks of study treatment.<br>[b] Patients who develop rash/skin toxicities should be seen by a qualified physician and should receive evaluation for symptomatic/supportive care management.	

Trametinib Dose Modification Guidelines and Management for Rash		
**Rash Severity**	**Management Guideline**	**Dose Modification**
**Grade 1**	• Initiate prophylactic and symptomatic treatment measures.[1]<br>• Use moderate strength topical steroid.[2]<br>• Reassess after 2 weeks.	• Continue trametinib.<br>• If rash does not recover to baseline within 2 weeks despite best supportive care, **reduce trametinib by one dose level**.[3]
**Grade 2**	• Initiate prophylactic and symptomatic treatment measures.[1]<br>• Use moderate strength topical steroid.[2]<br>• Reassess after 2 weeks.	• **Reduce trametinib by one dose level.**<br>• If rash recovers to ≤ grade 1 within 2 weeks, increase dose to previous dose level.<br>• If no recovery to ≤ grade 1 within 2 weeks, interrupt trametinib until recovery to ≤ grade 1.<br>• **Restart trametinib at reduced dose level.**[3]
**Grade ≥3**	• Use moderate strength topical steroids PLUS oral methyl-prednisolone dose pack.[2]<br>• Consult dermatologist.	• Interrupt trametinib until rash recovers to ≤ grade 1.<br>• **Restart with trametinib reduced by one dose level.**[3,4]<br>• If no recovery to ≤ grade 2 within 4 weeks, **permanently discontinue trametinib**.
1. Rash prophylaxis is recommended for the first 6 weeks of study treatment.<br>2. Moderate-strength topical steroids: Hydrocortisone 2.5% cream or fluticasone priopionate 0.5% cream.<br>3. Approval of CTEP Medical Monitor is required to restart study treatment after >4 weeks of interruption.<br>4. Trametinib may be escalated to previous dose level if no rash is evident 4 weeks after restarting study treatment.<br>5. CTEP Medical Monitor:<br>Helen Chen, MD<br>240-276-6565<br>Helen.chen@nih.gov		

6.23 Trametinib Dose Modifications for Visual Changes (07/28/14)

Trametinib is known to be associated with visual adverse events. An ophthalmologist should be consulted if changes in vision develop. However, if the visual changes are clearly unrelated to study treatment (e.g., allergic conjunctivitis), then monitor closely as it may be reasonable to defer ophthalmic examination. Special attention should be given to

retinal findings (*e.g.*, retinal pigment epithelial detachment (RPED) or retinovascular abnormalities (*i.e.*, branch or central retinal vein occlusions [RVO]). (12/22/14)

The ophthalmology exam will include best corrected visual acuity, visual field examination, tonometry, slit lamp biomicroscopic examination, and indirect fundoscopy. Optical coherence tomography is recommended at scheduled visits and if retinal abnormalities are suspected. Other types of ancillary testing including visual field examination, fundus photography, and fluorescein angiography may also be indicated as determined by clinical exam.

Guidelines regarding event management and dose reduction for visual changes considered to be related to study treatment are provided in the table below.

**Management and Trametinib Dose Modification for Visual Changes and/or Ophthalmic Examination Findings**		
**Event CTCAE Grade**	**Management Guideline**	**Dose Modification**
**Grade 1***	• Consult ophthalmologist within 7 days of onset.	• If dilated fundus examination cannot be performed within 7 days of onset, hold trametinib until RPED and RVO can be excluded by retina specialist/ophthalmologist.<br>• If RPED and RVO excluded, continue/or restart trametinib at same dose level.<br>• If RPED suspected/diagnosed: See RPED dose modification table below (following this table); **report as SAE**.<br>• If RVO diagnosed: **Permanently discontinue trametinib and report as SAE**.
**Grade 2 and Grade 3**	• Consult ophthalmologist immediately.	• Hold trametinib<br>• If RPED or RVO excluded, restart trametinib at same dose level after visual AE is ≤ grade 1. If no recovery within 3 weeks, discontinue trametinib<br>• If RPED diagnosed: See RPED dose modification table below; **report as SAE**.<br>• If RVO: **Permanently discontinue trametinib and report as SAE**.
**Grade 4**	• Consult ophthalmologist immediately.<br>• Report as SAE.	• Hold Trametinib<br>• If RPED/RVO excluded, may restart trametinib at same or reduced dose after discussion with the CTEP Medical Monitor.<br>• **If RVO or RPED, permanently discontinue trametinib**.
Abbreviations: RPED = retinal pigment epithelial detachments; RVO = retinal vein occlusion; SAE = serious adverse event<br>*If visual changes are clearly unrelated to study treatment (e.g., allergic conjunctivitis), monitor closely but ophthalmic examination is not required.		

Trametinib Dose Modification for RPED	
**Event<br>CTCAE Grade**	**Action and Dose Modification**
**Grade 1 RPED** (Asymptomatic; clinical or diagnostic observations only)	• Continue treatment with retinal evaluation monthly until resolution. If RPED worsens, follow instructions below.
**Grade 2-3 RPED** (Symptomatic with mild to moderate decrease in visual acuity; limiting instrumental ADL)	• Interrupt trametinib.<br>• Retinal evaluation monthly.<br>• If improved to ≤ Grade 1, restart trametinib with one dose level reduction (reduced by 0.5 mg) or discontinue in patients taking trametinib 1 mg daily.<br>• If no recovery within 4 weeks permanently discontinue trametinib

6.24 Trametinib Dose Modification for Diarrhea

Episodes of diarrhea have occurred in patients receiving trametinib (Investigator's Brochure, 2012a). Other frequent causes of diarrhea may include concomitant medications (*e.g.*, stool softeners, laxatives, antacids, *etc.*), infections by *C. difficile* or other pathogens, or partial bowel obstruction. Those conditions should be excluded.

Guidelines regarding management and dose modification for diarrhea considered related to trametinib are provided in the table below.

Management and Trametinib Dose Modification Guidelines for Diarrhea		
**CTCAE Grade**	**Adverse Event Management**	**Action and Dose Modification**
**Uncomplicated Diarrhea,[1]**<br>**Grade 1 or 2**	• Diet: Stop all lactose containing products; eat small meals, BRAT-diet (banana, rice, apples, toast) recommended.<br>• Hydration: 8-10 large glasses of clear liquids per day (*e.g.*, Gatorade or broth).<br>• Loperamide[3]: Initially 4 mg, followed by 2 mg every 4 hours or after every unformed stool; maximum 16 mg/day. Continue until diarrhea-free for 12 hours.<br>• Diarrhea >24 hours: Loperamide 2 mg every 2 hours; maximum 16 mg/day. Consider adding oral antibiotics.<br>• Diarrhea >48 hours: Loperamide 2 mg every 2 hours; maximum 16 mg/day. Add budesonide or other second-line therapies (otreotide, or tincture of opium) and oral antibiotics.	• Continue trametinib.<br>• If diarrhea is grade 2 for > 48 h, interrupt trametinib until diarrhea resolves to grade ≤1.<br>• Restart trametinib at the same dose level<br>• If treatment delay is > 1 days, discontinue trametinib.
**Uncomplicated Diarrhea,[1]**<br>**Grade 3 or 4**<br><br>**Any Complicated Diarrhea[2]**	• Clinical evaluation mandatory.<br>• Loperamide[3]: Initially 4 mg, followed by 2 mg every 4 hours or after every unformed stool; maximum 16 mg/day. Continue until diarrhea-free for 12 hours.<br>• Oral antibiotics and second-line therapies if clinically indicated<br>• Hydration: Intravenous fluids if clinically indicated.<br>• Antibiotics (oral or intravenous) if clinically indicated.<br>• Intervention should be continued until the subject is diarrhea-free for ≥24 hours.<br>• Intervention may require hospitalization for subjects at risk of life-threatening complications.	• Interrupt trametinib until diarrhea resolves to ≤ grade 1.<br>• Restart with trametinib reduced by one dose level.[4]<br>• If 3 dose reductions of study treatment are clinically indicated, **permanently discontinue trametinib**.<br>• If treatment delay is >21 days, discontinue trametinib.
1. **Uncomplicated diarrhea** defined by the absence of symptoms such as cramping, nausea/vomiting, ≥ grade 2, decreased performance status, pyrexia, sepsis, neutropenia ≥ grade 3, frank bleeding, and/or dehydration requiring intravenous fluid substitution.<br>2. **Complicated diarrhea** defined by the presence of symptoms such as cramping, nausea/vomiting, ≥ grade 2, decreased performance status, pyrexia, sepsis, neutropenia ≥ grade 3, frank bleeding, and/or dehydration requiring intravenous fluid substitution.<br>3. Loperamide should be made available prior to start of study treatment so loperamide administration can begin at the first signs of diarrhea.<br>4. Escalation of trametinib to previous dose level is allowed after consultation with the medical monitor and in the absence of another episode of complicated or severe diarrhea in the 4 weeks subsequent to dose reduction.		

6.25 Trametinib Dose Modification for Liver Chemistry Changes (07/28/14)

**Trametinib Dose Modification for Liver Function Test Abnormalities**	
**Event**	**Treatment modifications and assessment/monitoring**
• ALT ≥3x ULN **but** <5x ULN **and** TB <2x ULN, **without** symptoms considered related to liver injury or hypersensitivity **and** who can be monitored weekly for 4 weeks	• **May continue study drug**.<br>• Report as SAE if CTEP-AERS reporting criteria is met.<br>• If **liver chemistry stopping criteria** are met any time, proceed as described below.<br><br>**MONITORING:**<br>Repeat LFT (ALT, AST, ALK, bilirubin) until they return to normal/baseline or stabilise (LFT may be every 2 weeks after 4 weeks if ALT <3x ULN and TB <2 ULN).
<u>**Criteria for discontinuing study drug**</u>**:** When any of the liver stopping criteria below is met, discontinue trametinib<br><br>**1.** ALT ≥3xULN **and** <u>bilirubin</u> ≥2x ULN or >35% direct bilirubin [1, 2]<br>2. ALT ≥ 3xULN **and** <u>INR</u> >1.5, if INR measured[2] (INR threshold does not apply if subject is on anticoagulant)<br>**3.** ALT ≥5x ULN<br>4. ALT ≥3x ULN persists for ≥4 weeks<br>5. ALT ≥3x ULN **and** cannot be monitored weekly for 4 weeks<br>6. ALT ≥3x ULN associated with symptoms[3] (new or worsening) believed to be related to liver injury or hypersensitivity	• **Immediately discontinue study treatment**.<br>• **Do not restart/rechallenge** unless approved by CTEP trametinib medical monitor.<br>• Report as SAE if: 1) CTEP-AERS reporting criteria are met, or 2) patients meet criteria 1-2.<br>• Perform liver event **ASSESSMENT AND WORKUP (see below)**.<br>• Monitor the subject until liver chemistries resolve, stabilize, or return to baseline (see **MONITORING** below).<br><br>**MONITORING:**<br>*<u>In patients stopping for criteria 1-2</u> (with abnormal TB and INR, indicating potentially more significant liver toxicities):*<br>• Repeat liver chemistries (ALT, AST, ALK, bilirubin) and perform liver event **follow-up assessments** within **24 hours**.<br>• Monitor subjects twice weekly until LFT return to normal/baseline or stabilize.<br>• A specialist or hepatology consultation is recommended.<br>*<u>In patients stopping for criteria 2-6:</u>*<br>• Repeat LFT and perform liver event **follow up assessments** within **24-72 hrs**<br>• Monitor subjects weekly until LFTs return to normal/baseline or stabilize.<br><br>**ASSESSMENT and WORKUP:**<br>• Viral hepatitis serology.[4]<br>• If possible, obtain blood sample for PK analysis.[5]

<table>
<tr>
<td></td>
<td>
• Serum CPK and LDH.
• Fractionate bilirubin, if total bilirubin ≥2x ULN.
• CBC with differential to assess eosinophilia.
• Record clinical symptoms of liver injury, or hypersensitivity on AE CRF.
• Record concomitant medications (including acetaminophen, herbal remedies, other over the counter medications).
• Record alcohol use.
<u>Additional work up for patient stopping for criteria 1-2 </u>(with abnormal TB and INR, indicating potentially more significant liver toxicities):
• Anti-nuclear antibody, anti-smooth muscle antibody, Type 1 anti-liver kidney microsomal antibodies, and quantitative total immunoglobulin G (IgG or gamma globulins).
• Serum acetaminophen adduct HPLC assay (in subjects with likely acetaminophen use in the preceding).
• If there is underlying chronic hepatitis B (e.g. positive hepatitis B surface antigen): quantitative hepatitis B DNA and hepatitis delta antibody.[6]
• Liver imaging (ultrasound, MRI, CT) and /or liver biopsy.
</td>
</tr>
<tr>
<td colspan="2">
Footnotes:
1. Serum bilirubin fractionation should be performed if testing is available. If serum bilirubin fractionation testing is unavailable, record presence of detectable urinary bilirubin on dipstick, which indicates direct bilirubin elevations and suggesting liver injury.
2. All events of ALT ≥3xULN and bilirubin ≥2xULN (>35% direct bilirubin) or ALT ≥3x ULN and INR >1.5 (if INR measured) may indicate severe liver injury (possible “Hy’s Law”). INR measurement is not required, and the threshold value stated will not apply to subjects receiving anticoagulants.
3. New or worsening symptoms believed to be related to liver injury (such as fatigue, nausea, vomiting, right upper quadrant pain or tenderness, or jaundice) or believed to be related to hypersensitivity (such as fever, rash or eosinophilia)
4. Includes: Hepatitis A IgM antibody; Hepatitis B surface antigen and Hepatitis B Core Antibody (IgM); Hepatitis C RNA; Cytomegalovirus IgM antibody; Epstein-Barr viral capsid antigen IgM antibody (or if unavailable, obtain heterophile antibody or monospot testing); Hepatitis E IgM antibody
5. PK sample is desired if feasible. Record the date/time of the PK blood sample draw and the date/time of the last dose of study treatment prior to blood sample draw on the CRF. If the date or time of the last dose is unclear, provide the subject’s best approximation. If the date/time of the last dose cannot be approximated OR a PK sample cannot be collected in the time period indicated above, do not obtain a PK sample.
6. If hepatitis delta antibody assay cannot be performed, it can be replaced with a PCR of hepatitis D RNA virus (where needed) (Le Gal et al., 2005).
7. CTEP Medical Monitor:
Helen Chen, MD
240-276-6565
Helen.chen@nih.gov
</td>
</tr>
</table>

6.26 Trametinib Dose Modification for Pneumonitis

Pneumonitis has been observed in patients receiving trametinib. To reduce the risk of pneumonitis, patients will be monitored closely for symptoms and evaluated with imaging and functional tests. Dose modification and supportive care guidelines for pneumonitis are described in the tables below.

Pneumonitis Guidelines for Trametinib Monotherapy		
**CTCAE Grade**	**Adverse Event Management**	**Action and Dose Modification**
**Grade 1**	• CT scan (high-resolution with lung windows) recommended.<br>• Work-up for infection.<br>• Monitoring of oxygenation via pulse-oximetry recommended.<br>• Consultation with pulmonologist recommended.	Continue trametinib at current dose.
**Grade 2**	• CT scan (high-resolution with lung windows).<br>• Work-up for infection.<br>• Consult pulmonologist.<br>• Pulmonary function tests: If < normal, repeat every 8 weeks until ≥ normal.<br>• Bronchoscopy with biopsy and/or BAL recommended.<br>• Symptomatic therapy including corticosteroids if clinically indicated.	• Interrupt trametinib until recovery to grade ≤1.<br>• If AE resolved to grade ≤1 and relationship to trametinib is equivocal, restarting trametinib with one dose reduction may be considered, after discussion with the medical monitor.<br>• If treatment delay is > 4 weeks, **permanently discontinue trametinib**.
**Grade 3**	• CT scan (high-resolution with lung windows).<br>• Work-up for infection.<br>• Consult pulmonologist.<br>• Pulmonary function tests-if < normal, repeat every 8 weeks until ≥ normal.<br>• Bronchoscopy with biopsy and/or BAL if possible.<br>• Symptomatic therapy including corticosteroids as clinically indicated.	• Interrupt trametinib until recovery to grade ≤1.<br>• If AE resolved to grade ≤1 and relationship to trametinib is equivocal, restarting trametinib with one dose reduction may be considered, after discussion with the medical monitor.<br>• If treatment delay is >4 weeks, **permanently discontinue trametinib**.
**Grade 4**	Same as grade 3.	**Permanently discontinue trametinib.**
Abbreviations: BAL = bronchoalveolar lavage; CT = computed tomography.		

6.27 Trametinib Dose Modification for Reduced Left Ventricular Ejection Fraction **(07/28/14) (06/08/15)**

Decreases of the left ventricular ejection fraction (LVEF) have been observed in patients receiving trametinib. Therefore, ECHO/MUGAs

must be performed in regular intervals outlined in the Study Calendar. The same procedure (either ECHO or MUGA, although ECHO is preferred) should be performed at baseline and at follow-up visit(s).

Trametinib Dose Modification Guidelines and Stopping Criteria for LVEF Decrease		
**Clinic**	**LVEF-drop (%) or CTCAE grade**	**Action and Dose Modification**
**Asymptomatic**	Absolute decrease of >10% in LVEF compared to baseline and ejection fraction below the institution's LLN.	• Interrupt trametinib and repeat ECHO/MUGA within 2 weeks.[a]<br>• If the LVEF **recovers** within 4 weeks (defined as an absolute increase of 10 points, or a return back to baseline of greater than or equal to 50%, would be considered resolved):<br>– Consult with the CTEP trametinib medical monitor and request approval for restart.<br>– Restart treatment with trametinib at reduced dose by one dose level.[b]<br>– Repeat ECHO/MUGA 2, 4, 8, and 12 weeks after re-start; continue in intervals of 12 weeks thereafter.<br>• If LVEF **does not** recover within 4 weeks:<br>– Consult with cardiologist.<br>– **Permanently discontinue trametinib**.<br>– Report as SAE<br>– Repeat ECHO/MUGA after 2, 4, 8, 12, and 16 weeks or until resolution.<br>– Consult with the CTEP trametinib medical monitor.[c]
**Symptomatic**[b]	• Grade 3: resting LVEF 39-20% or >20% absolute reduction from baseline<br>• Grade 4: Resting LVEF ≤20%.	• **Permanently discontinue trametinib**.<br>• **Report as SAE**.<br>• **Consult with cardiologist**.<br>• Repeat ECHO/MUGA after 2, 4, 8, 12, and 16 weeks or until resolution.
[a] If ECHO/MUGA does not show LVEF recovery after 2 weeks, repeat ECHO/MUGA 2 weeks later.<br>[b] Escalation of trametinib to previous dose level can be considered if LVEF remains stable for 4 weeks after restarting of trametinib. Approval from the CTEP trametinib medical monitor is required.<br>[c] Symptoms may include: dyspnea, orthopnea, and other signs and symptoms of pulmonary congestion and edema.		

6.28 Trametinib Dose Modification for QTc Prolongation (07/28/14) (06/08/15)

Trametinib Withholding and Stopping Criteria for QTc Prolongation	
**QTc Prolongation[a]**	**Action and Dose Modification**
• QTcB ≥501 msec, or<br>• Uncorrected QT >600 msec, or<br>• QTcB >530 msec for subjects with bundle branch block	• Interrupt study treatment until QTcB prolongation resolves to grade 1 or baseline.<br>• Test serum potassium, calcium, phosphorus, and magnesium. If abnormal, correct per routine clinical practice to within normal limits.<br>• Review concomitant medication usage for a prolonged QTc.<br>• Restart at current dose level.[b]<br>• **If the event does not resolve or recurs after restarting, permanently discontinue study treatment.**
Abbreviations: msec = milliseconds; QTcB = QT interval on electrocardiogram corrected using Bazett's formula<br>[a] Based on average QTc value of triplicate ECGs. For example, if an ECG demonstrates a prolonged QT interval, obtain two or more ECGs over a brief period, and then use the averaged QTc values of the three ECGs to determine if study treatments should be interrupted or discontinued.<br>[b] if the QTc prolongation resolves to grade 1 or baseline, the subject may resume study treatment if the investigator and the CTEP trametinib medical monitor agree that the subject will benefit from further treatment.	

MEDICATIONS THAT MAY CAUSE QTc PROLONGATION (06/08/15)

The following table presents a list of drugs that may prolong the QTc. These drugs are prohibited during the study. Trametinib may be administered after a 5 half-life washout period elapses following the use of these drugs. Washout period is based on roughly 5 half-lives and rounded to a convenient interval.

The drug list below contains drugs from the categories: Known Risk of TdP Possible Risk of TdP

Generic Name	Brand Names (Partial List)	Drug Class	Therapeutic Use	Risk Category	Route
Alfuzosin	Uroxatral®	Alpha1-blocker	Benign prostatic hyperplasia	Possible Risk of TdP	oral
Amiodarone	Cordarone®, Pacerone®, Nexterone®	Anti-arrhythmic	Abnormal heart rhythm	Risk of TdP	oral, injection
Anagrelide	Agrylin®, Xagrid®	Phosphodiesterase 3 inhibitor	Thrombocythemia	Risk of TdP	oral
Apomorphine	Apokyn®, Ixense®, Spontane®, Uprima®	Dopamine agonist	Parkinson's disease	Possible Risk of TdP	injection
Aripiprazole	Abilify®, Aripiprex®	Anti-psychotic, atypical	Schizophrenia, depression (adjunct)	Possible Risk of TdP	oral, injection
Arsenic trioxide	Trisenox®	Anti-cancer	Cancer (leukemia)	Risk of TdP	injection

Astemizole (Removed from US Market) (Removed from US Market)	Hismanal®	Antihistamine	Allergic rhinitis	Risk of TdP	oral
Atazanavir	Reyataz®	Anti-viral	Viral infection (HIV/AIDS)	Possible Risk of TdP	oral
Atomoxetine (On US Market)	Strattera®	Norepinephrine reuptake inhibitor	ADHD	Possible Risk of TdP	oral
Azithromycin	Zithromax®, Zmax®	Antibiotic	Bacterial infection	Risk of TdP	oral, injection
Bedaquiline	Sirturo®	Antibiotic	Bacterial infection (Drug resistant tuberculosis)	Possible Risk of TdP	oral
Bepridil (Removed from US Market) (Removed from US Market)	Vascor®	Anti-anginal	Angina Pectoris (heart pain)	Risk of TdP	oral
Bortezomib	Velcade®, Bortecad®	Proteasome inhibitor	Cancer (multiple myeloma,lymphoma)	Possible Risk of TdP	injection
Bosutinib	Bosulif®	Tyrosine kinase inhibitor	Cancer (leukemia)	Possible Risk of TdP	oral
Chloroquine	Aralen®	Anti-malarial	Malaria	Risk of TdP	oral
Chlorpromazine	Thorazine®, Largactil®, Megaphen®	Anti-psychotic / Anti-emetic	Schizophrenia, nausea, many others	Risk of TdP	oral, injection, suppositor
Cilostazol (On US Market)	Pletal®	Phosphodiesterase 3 inhibitor	Intermittent claudication	Risk of TdP	oral
Ciprofloxacin	Cipro®, Cipro-XR®, Neofloxin®	Antibiotic	Bacterial infection	Risk of TdP	oral, injection
Cisapride (Removed from US Market) (Removed from US Market)	Propulsid®	GI stimulant	Increase GI motility	Risk of TdP	oral
Citalopram	Celexa®, Cipramil®	Anti-depressant, SSRI	Depression	Risk of TdP	oral
Clarithromycin	Biaxin®, Prevpac®	Antibiotic	Bacterial infection	Risk of TdP	oral
Clozapine	Clozaril®, Fazaclo®, Versacloz®	Anti-psychotic, atypical	Schizophrenia	Possible Risk of TdP	oral
Cocaine	Cocaine	Local anesthetic	Anesthesia (topical)	Risk of TdP	topical
Crizotinib	Xalkori®	Kinase inhibitor	Cancer (Non-small cell lung cancer, metastatic)	Possible Risk of TdP	oral
Dabrafenib	Tafinlar®	Kinase inhibitor	Cancer (melanoma)	Possible Risk of TdP	oral

Dasatinib	Sprycel®	Tyrosine kinase inhibitor	Cancer (leukemia)	Possible Risk of TdP	oral
Dexmedetomidine	Precedex®, Dexdor®, Dexdomitor®	Sedative	Sedation	Possible Risk of TdP	injection
Dihydroartemisinin+piperaquine	Eurartesim®	Anti-malarial	Malaria	Possible Risk of TdP	oral
Disopyramide	Norpace®	Anti-arrhythmic	Abnormal heart rhythm	Risk of TdP	oral
Dofetilide	Tikosyn®	Anti-arrhythmic	Abnormal heart rhythm	Risk of TdP	oral
Dolasetron	Anzemet®	Anti-nausea	Nausea, vomiting	Possible Risk of TdP	oral, injection
Domperidone (On non US Market) (On non US Market)	Motilium®, Motillium®, Motinorm Costi®, Nomit®	Anti-nausea	Nausea, vomiting	Risk of TdP	oral, injection, suppositor
Donepezil	Aricept®	Cholinesterase inhibitor	Dementia (Alzheimer's Disease)	Risk of TdP	oral
Dronedarone	Multaq®	Anti-arrhythmic	Abnormal heart rhythm	Risk of TdP	oral
Droperidol	Inapsine®, Droleptan®, Dridol®, Xomolix®	Anti-psychotic / Anti-emetic	Anesthesia (adjunct), nausea	Risk of TdP	injection
Eribulin	Halaven®	Anti-cancer	Cancer (breast, metastatic)	Possible Risk of TdP	injection
Erythromycin	E.E.S.®, Robimycin®, EMycin®, Erymax®,	Antibiotic	Bacterial infection, increase GI motility	Risk of TdP	oral, injection
Escitalopram	Cipralex®, Lexapro®, Nexito®, Anxiset-E® (India), Exodus® (Brazil), Esto®	Anti-depressant, SSRI	Depression (major), anxiety disorders	Risk of TdP	oral
Famotidine	Pepcid®, Fluxid®, Quamatel®	H2-receptor antagonist	Gastric hyperacidity, GERD	Possible Risk of TdP	oral, injection
Felbamate	Felbatol®	Anti-convulsant	Epilepsy	Possible Risk of TdP	oral
Fingolimod	Gilenya®	Sphingosine phospate receptor modulator	Multiple Sclerosis	Possible Risk of TdP	oral
Flecainide	Tambocor®, Almarytm®, Apocard®, Ecrinal®, Flécaine®	Anti-arrhythmic	Abnormal heart rhythm	Risk of TdP	oral
Fluconazole	Diflucan®, Trican®	Anti-fungal	Fungal infection	Risk of TdP	oral, injection
Foscarnet	Foscavir®	Anti-viral	Viral infection (HIV/AIDS)	Possible Risk of TdP	injection
Gatifloxacin (Removed from US Market) (Removed from US Market)	Tequin®	Antibiotic	Bacterial infection	Possible Risk of TdP	oral, injection
Gemifloxacin	Factive®	Antibiotic	Bacterial infection	Possible Risk of TdP	oral
Granisetron	Kytril®, Sancuso®, Granisol®	Anti-nausea	Nausea, vomiting	Possible Risk of TdP	oral, injection, topical

Grepafloxacin (Off market worldwide) (Off market worldwide)	Raxar®	Antibiotic	Bacterial infection	Risk of TdP	oral
Halofantrine	Halfan®	Anti-malarial	Malaria	Risk of TdP	oral
Haloperidol	Haldol® (US & UK), Aloperidin®, Bioperidolo®, Brotopon®, Dozic®	Anti-psychotic	Schizophrenia, agitation	Risk of TdP	oral, injection
Ibutilide	Corvert®	Anti-arrhythmic	Abnormal heart rhythm	Risk of TdP	injection
Iloperidone	Fanapt®, Fanapta®, Zomaril®	Anti-psychotic, atypical	Schizophrenia	Possible Risk of TdP	oral, injection
Isradipine	Dynacirc®	Anti-hypertensive	Hypertension	Possible Risk of TdP	oral
Lapatinib	Tykerb®, Tyverb®	Anti-cancer	Cancer (breast, metastatic)	Possible Risk of TdP	oral
Levofloxacin	Levaquin®, Tavanic®	Antibiotic	Bacterial infection	Risk of TdP	oral, injection
Levomethadyl (Removed from US Market) (Removed from US Market)	Orlaam®	Opiate	Narcotic dependence	Risk of TdP	oral
Lithium	Eskalith®, Lithobid®	Anti-mania	Bipolar disorder	Possible Risk of TdP	oral, injection
Mesoridazine (Removed from US Market) (Removed from US Market)	Serentil®	Anti-psychotic	Schizophrenia	Risk of TdP	oral
Methadone	Dolophine®, Symoron®, Amidone®, Methadose®, Physeptone®, Heptadon®	Opiate	Narcotic dependence, pain	Risk of TdP	oral, injection
Mifepristone	Korlym®, Mifeprex®	Progesterone antagonist	Pregnancy termination	Possible Risk of TdP	oral
Mirabegron	Myrbetriq®	Beta3 adrenergic antagonist	Bladder spasm	Possible Risk of TdP	oral
Mirtazapine	Remeron	Anti-depressant, Tetracyclic	Depression	Possible Risk of TdP	oral
Moexipril/HCTZ	Uniretic®, Univasc®	Anti-hypertensive	Hypertension	Possible Risk of TdP	oral
Moxifloxacin	Avelox®, Avalox®, Avelon®	Antibiotic	Bacterial infection	Risk of TdP	oral, injection
Nicardipine	Cardene®	Anti-hypertensive	Hypertension	Possible Risk of TdP	oral, injection
Nilotinib	Tasigna®	Anti-cancer	Cancer (leukemia)	Possible Risk of TdP	oral
Norfloxacin	Noroxin®, Ambigram®	Antibiotic	Bacterial infection	Possible Risk of TdP	oral
Ofloxacin	Floxin®	Antibiotic	Bacterial infection	Possible Risk of TdP	oral, injection

Olanzapine	Zyprexa®, Zydis®, Relprevv®	Anti-psychotic, atypical	Schizophrenia, bipolar disorder	Possible Risk of TdP	oral, injection
Ondansetron	Zofran®, Anset®, Ondemet®, Zuplenz®, Emetron®, Ondavell®,	Anti-emetic	Nausea, vomiting	Risk of TdP	oral, injection
Oxytocin	Pitocin®, Syntocinon®	Oxytocic	Labor stimulation	Possible Risk of TdP	injection
Paliperidone	Invega®, Xepilon®	Anti-psychotic, atypical	Schizophrenia	Possible Risk of TdP	oral, injection
Pasireotide	Signifor®	Somatostatin analog	Cushings Disease	Possible Risk of TdP	injection, topical
Pazopanib	Votrient®	Tyrosine kinase inhibitor	Cancer (renal cell, sarcoma)	Possible Risk of TdP	oral
Pentamidine	Pentam®	Antifungal	Fungal infection (Pneumocystis pneumonia)	Risk of TdP	injection
Perflutren lipid microspheres	Definity®	Imaging contrast agent	Echocardiography	Possible Risk of TdP	injection
Pimozide	Orap®	Anti-psychotic	Tourette's Disorder	Risk of TdP	oral
Pipamperone (On non US Market) (On non US Market)	Dipiperon (E.U), Propitan (Japan)	Antipsychotic	Schizophrenia	Possible Risk of TdP	oral
Probucol (Removed from US Market) (Removed from US Market)	Lorelco®	Antilipemic	Hypercholesterolemia	Risk of TdP	oral
Procainamide (Oral off US mkt) (Oral off US mkt)	Pronestyl®, Procan®	Anti-arrhythmic	Abnormal heart rhythm	Risk of TdP	injection
Promethazine	Phenergan®	Anti-psychotic / Anti-emetic	Nausea, vomiting	Possible Risk of TdP	oral, injection, suppositor
Propofol	Diprivan®, Propoven®	Anesthetic, general	Anesthesia	Risk of TdP	injection
Quetiapine	Seroquel®	Anti-psychotic, atypical	Schizophrenia	Possible Risk of TdP	oral
Quinidine	Quinaglute®, Duraquin®, Quinact®, Quinidex®, Cin-Quin®, Quinora®	Anti-arrhythmic	Abnormal heart rhythm	Risk of TdP	oral, injection
Ranolazine	Ranexa®, Ranozex®	Anti-anginal	Angina Pectoris (heart pain)	Possible Risk of TdP	oral
Rilpivirine	Edurant®, Complera®, Eviplera®	Anti-viral	Viral infection (HIV/AIDS)	Possible Risk of TdP	oral
Risperidone	Risperdal®	Anti-psychotic, atypical	Schizophrenia	Possible Risk of TdP	oral, injection
Roxithromycin (On non US Market) (On non US Market)	Rulide®, Xthrocin®, Roxl-150®, Roxo®, Surlid®, Rulide®, Biaxsig®, Roxar®,	Antibiotic	Bacterial infection	Possible Risk of TdP	oral

Saquinavir	Invirase®(combo)	Anti-viral	Viral infection (HIV/AIDS)	Possible Risk of TdP	oral
Sertindole (On non US Market) (On non US Market)	Serdolect®, Serlect®	Anti-psychotic, atypical	Schizophrenia, anxiety	Possible Risk of TdP	oral
Sevoflurane	Ulane®, Sojourn®	Anesthetic, general	Anesthesia	Risk of TdP	inhaled
Sorafenib	Nexavar®	Tyrosine kinase inhibitor	Cancer (liver, renal cell, metastatic thyroid)	Possible Risk of TdP	oral
Sotalol	Betapace®, Sotalex®, Sotacor®	Anti-arrhythmic	Abnormal heart rhythm	Risk of TdP	oral
Sparfloxacin (Removed from US Market) (Removed from US Market)	Zagam®	Antibiotic	Bacterial infection	Risk of TdP	oral
Sulpiride (On non US Market) (On non US Market)	Dogmatil®, Dolmatil®, Eglonyl®, Espiride®, Modal®, Sulpor®	Anti-psychotic, atypical	Schizophrenia	Risk of TdP	oral
Sunitinib	Sutent®	Kinase inhibitor	Cancer (GIST, renal cell, pNET)	Possible Risk of TdP	oral
Tacrolimus	Prograf®, Prograf®, Advagraf®, Protopic®	Immunosuppressant	Immune suppression	Possible Risk of TdP	oral, injection
Tamoxifen	Nolvadex®(discontinued 6/13), Istubal®, Valodex®	Anti-cancer	Cancer (breast)	Possible Risk of TdP	oral
Telavancin	Vibativ®	Antibiotic	Bacterial infection	Possible Risk of TdP	injection
Telithromycin	Ketek®	Antibiotic	Bacterial infection	Possible Risk of TdP	oral
Terfenadine (Removed from US Market) (Removed from US Market)	Seldane®	Antihistamine	Allergic rhinitis	Risk of TdP	oral
Tetrabenazine (Orphan drug in US) (Orphan drug in US)	Nitoman®, Xenazine®	Monoamine Transporter Inhibitor	Chorea (Huntington's disease)	Possible Risk of TdP	oral
Thioridazine	Mellaril®, Novoridazine®, Thioril®	Anti-psychotic	Schizophrenia	Risk of TdP	oral
Tizanidine	Zanaflex®, Sirdalud®	Muscle relaxant	Muscle spasticity	Possible Risk of TdP	oral
Tolterodine	Detrol®, Detrusitol®	Muscle relaxant	Bladder spasm	Possible Risk of TdP	oral
Toremifene	Fareston®	Estrogen agonist/antagonist	Cancer (breast, metastatic)	Possible Risk of TdP	oral
Vandetanib	Caprelsa®	Anti-cancer	Cancer (thyroid)	Risk of TdP	oral
Vardenafil	Levitra®	Phosphodiesterase 5 inhibitor	Erectile dysfunction	Possible Risk of TdP	oral

Vemurafenib	Zelboraf®	Kinase inhibitor	Cancer (melanoma)	Possible Risk of TdP	oral
Venlafaxine	Effexor®, Efexor®	Anti-depressant, SNRI	Depression	Possible Risk of TdP	oral
Vorinostat	Zolinza®	Anti-cancer	Cancer (lymphoma)	Possible Risk of TdP	oral
Ziprasidone	Geodon®, Zeldox®	Anti-psychotic, atypical	Schizophrenia	Possible Risk of TdP	oral, injection

6.29 Trametinib Dose Modification for Hypertension (07/28/14)

Increases in blood pressure (BP) have been observed in patients receiving trametinib. Recommendations for BP monitoring and management are provided below.

Monitoring: All BP assessments should be performed under the following optimal conditions:

- The subject has been seated with back support, ensuring that legs are uncrossed and flat on the floor.
- The subject is relaxed comfortably for at least 5 minutes.
- Restrictive clothing has been removed from the cuff area, and the right cuff size has been selected.
- The subject's arm is supported so that the middle of the cuff is at heart level.
- The subject remains quiet during the measurement.
- In subjects with an initial BP reading within the hypertensive range, a second reading should be taken at least 1 minute later, with the two readings averaged to obtain a final BP measurement. The averaged value should be recorded in the eCRF.
- Persistent hypertension is defined as an increase of systolic blood pressure (SBP) >140 mmHg and/or diastolic blood pressure (DBP) >90 mmHg in three consecutive visits with blood pressure assessments from two readings as described above. Visits to monitor increased blood pressure can be scheduled independently from the per-protocol visits outlined in the study calendar. Ideally, subsequent blood pressure assessments should be performed within 1 week.

Management and Trametinib Dose Modification for Hypertension		
**Event**	**Management Guideline**	**Dose Modification**
Definitions used in the table:<br>- Persistent hypertension: Hypertension detected in two separate readings during up to three subsequent visits.<br>- Well-controlled hypertension: Blood pressure of SBP ≤140 mmHg and DBP ≤90 mmHg in two separate readings during up to three subsequent visits.<br>- Symptomatic hypertension: Hypertension associated with symptoms (*e.g.*, headache, light-headedness, vertigo, tinnitus, episodes of fainting or other symptoms indicative of hypertension) that resolve after the blood pressure is controlled within the normal range.<br>- Asymptomatic hypertension: SBP >140 mmHg and/or DBP >90 mmHg in the absence of the above symptoms.		
**(Scenario A)**<br>• Asymptomatic and persistent SBP of ≥140 and <160 mmHg, or DBP ≥90 and <100 mmHg,<br>or<br>Clinically significant increase in DBP of 20 mmHg (but still below 100 mmHg).	• Adjust current or initiate new antihypertensive medication(s).<br>• Titrate antihypertensive medication(s) during the next 2 weeks to achieve well-controlled BP. If BP is not well-controlled within 2 weeks, consider referral to a specialist and go to scenario (B).	Continue trametinib at the current dose.
**(Scenario B)**<br>• Asymptomatic SBP ≥160 mmHg, or DBP ≥100 mmHg,<br>or<br>Failure to achieve well-controlled BP within 2 weeks in Scenario A.	• Adjust current or initiate new antihypertensive medication(s).<br>• Titrate antihypertensive medication(s) during the next 2 weeks to achieve well-controlled BP.	• Interrupt trametinib if clinically indicated.<br>• Once BP is well-controlled, restart trametinib **reduced by one dose level**.[a]
**(Scenario C)**<br>• Symptomatic hypertension<br>or<br>Persistent SBP ≥160 mmHg, or DBP ≥100 mmHg, despite antihypertensive medication and dose reduction of trametinib	• Adjust current or initiate new antihypertensive medication(s).<br>• Titrate antihypertensive medication(s) during the next 2 weeks to achieve well-controlled BP.<br>• Referral to a specialist for further evaluation and follow-up is recommended.	• Interrupt trametinib.<br>• Once BP is well-controlled, restart trametinib **reduced by one dose level**.[a]
**(Scenario D)**<br>Refractory hypertension unresponsive to above interventions or hypertensive crisis.	Continue follow-up per protocol.	**Permanently discontinue trametinib**.
a. Escalation of trametinib to previous dose level can be considered if BPs remain well controlled for 4 weeks after restarting of trametinib. Approval from Medical Monitor is required.		

7.0 STUDY PARAMETERS

7.1 Tests and Observations (07/28/14)(06/08/15)

The following observations and tests are to be performed and recorded on the appropriate forms:

**Study Assessments**	**Prior to Study Therapy**	**During Treatment (randomized or crossover)**		**Crossover from Standard Arm to Trametinib Arm**	**Every 3 months for 2 years, every 6 months for 3 years, then annually‡**
		**Prior to Each Cycle**	**Prior to Cycles 2, 5, 8, 11 (then every 4 cycles)†**		
Confirmation of Eligibility	X			X	
History & Physical	1, 7	7		7	X
Serum pregnancy test	2, 3			X	
Ophthalmologic Exam*	1	12		X	
Assessment of visual disorders	X	X		X	
Radiographic disease assessment	4	4			
Quality of life assessment	X	5			
Vital signs (including Height/Weight)	2, 7	7		X	X
ECG	1,7		7,10	7	
Echocardiogram (ECHO)/MUGA*	1		9,10		11
CBC/Differential/ Platelets	2	6		X	X
Electrolytes (including potassium), BUN, creatinine, Ca, Mg, PO_4, Albumin	2	6		X	11
Bilirubin, SGOT(AST), SGPT(ALT), Alkaline Phosphatase	2	X		X	11
PTT, PT/INR	2			X	11
CA125	2	X		X	X
Toxicity Assessment	X	X		X	X
Urinalysis	X				

† Cycles are relative to randomized or crossover treatment. For example, after crossover, cycle 2 refers to the 2nd cycle of crossover treatment.
‡ Relative to end of most recent study treatment, whether randomized or crossover.
* Not standard of care, will be paid for by from study funds, email Nicole Lamack Nlamack@gog.org (07/28/14)
MRI = magnetic resonance imaging; ECG = electrocardiogram; ECHO = echocardiogram

1. Must be obtained within 28 days prior to initiating protocol therapy.
2. Must be obtained within 14 days prior to initiating protocol therapy.
3. Serum pregnancy test will be required in women of child-bearing potential within 14 days prior to randomization. Subsequent tests may be urine tests, and should be performed as clinically indicated.

4. Lesion assessment by contrast CT or MRI of chest, abdomen and pelvis every 8 weeks for the first 15 months, then every 3 months thereafter must be performed within 28 days prior to randomization. Assessments must be performed on a calendar basis (i.e., not delayed due to delays in administering study medication). A window of ± 7 days is allowed for scheduling. The same diagnostic method, including use of contrast when applicable, must be used throughout the study to evaluate each lesion. Lesion assessment [contrast CT (preferred) or MRI of chest, abdomen and pelvis], should occur at times shown until disease progression (even if the patient withdraws from treatment) but should continue after progression in patients who crossover from the standard arm to the Trametinib arm and should continue in that arm until progression.
   Scans should be sent on **CD*** labelled with NRG Patient ID to:
   NRG Oncology Statistics and Data Management Center – Buffalo Office
   Roswell Park Cancer Institute
   Elm & Carlton Streets
   Buffalo, NY 14263
   *Note: In that event that an Institution's policies do not permit for scans to be sent via CD, the institution staff should contact Amy Speaker (SpeakerA@nrgoncology.org) at the NRG Oncology SDMC – Buffalo Office to coordinate another Institution-provided means of electronic delivery – including, but not limited to tools such as Box.com. (11/27/17)
5. QOL assessments should be done at the following time points:
   - Prior to cycle 1
   - Prior to cycle 4 (12 weeks after starting treatment),
   - 4 weeks post cycle 6 (24 weeks after starting study therapy)
   - 36 weeks after starting treatment
   - 52 weeks after starting treatment
6. Must be obtained within 4 days prior to next cycle.
7. Record blood pressure, pulse rate and body weight. Height measured only at screening.
8. Two copies of the ECG tracing should be obtained at the time of the ECG, one to be kept in the site's study file for retrospective collection by the sponsor if necessary.
9. Post baseline ECHO (preferred)/MUGA must be done for patients receiving Trametinib. Subjects who have an asymptomatic, absolute decrease of >10% in LVEF compared to baseline and the ejection fraction is below the institution's lower limit of normal (LLN) must follow the LVEF guidelines for study drug management and requirements for subsequent ECHO/MUGA. The same modality (ECHO or MUGA) should be used at baseline and at follow-up.
10. For those on the standard arm, it is recommended that investigators follow institutional guidelines for ECG and ECHO/MUGA.
11. When clinically indicated.
12. At each visit, patients on both arms of the study will be queried regarding visual disturbances. If visual disturbances are reported, they should be carefully documented, and a follow-up ophthalmologic exam is required.

7.2 Pathology Requirements

7.21 Pathology review for confirmation of eligibility (11/27/17)

This protocol requires prospective digital review of pathology to confirm eligibility. Regardless of whether the patient's original diagnosis was ovarian or peritoneal serous tumor of low malignant potential or low-grade serous carcinoma, eligible patients include those with recurrent low-grade serous carcinoma of the ovary or peritoneum. One H&E stained slide from the recurrent tumor (obtained at secondary surgery, prior biopsy, or the image-guided fine needle/core biopsy) or from the original diagnostic specimen documenting low-grade serous carcinoma should be obtained from the pathology department and sent by overnight courier at the

institution’s expense to the GOG Tissue Bank in Columbus, Ohio, for determination of eligibility by review of digital slides. Please also send the screening form and a copy of the patient’s pathology report that has been labeled with the Screening ID with the slides. Slides must also be labeled with the Screening ID ,the surgical pathology accession number and the block number.

GOG-0281 Pathology Review
Nationwide Children’s Hospital
700 Children’s Drive, WA 1340
Columbus, OH 43205
614-722-2865

7.22 Protocol for digital review of pathology slides

A representative H&E stained slide will be scanned at the GOG Tissue Bank into a GOG-0281 digital tissue review database.

A panel of 5 pathologists will be notified by email that a case is ready for central prospective review. Confirmation of protocol eligibility requires agreement of at least 3 pathologists on the diagnosis of recurrent low-grade serous carcinoma using VIPER (Virtual Imaging for Pathology Education and Research). The institution will be notified of patient eligibility by email with 3 business days of receipt of the slide at the GOG Tissue Bank. At that time, institution personnel can go to the GOG-0281 Screening web site to confirm a patient’s eligibility for enrollment in GOG-0281.

7.3 Translational Research

Note: Testing of banked samples will not occur until an amendment to this treatment protocol (or separate correlative science protocol) is reviewed and approved in accordance with National Clinical Trials Network (NCTN) policies.

7.31 Specimen Requirements (07/28/14) (06/08/15) (11/27/17)

Note: Frozen biopsy and PK plasma specimens are no longer required as of October 2017, and should not be collected.

If the patient gives permission for her fixed tissue and blood specimens to be used for translational research, then the participating institution is required to submit the patient’s specimens as outlined below (unless otherwise specified).

A detailed description of the specimen requirements and procedures can be found in Appendix IV.

All Patients (Arms A and B) (11/27/17)

**Required Specimen (Specimen Code)**	**Collection Time Point**	**Sites Ship Specimens To**
FFPE Primary Tumor (FP01)*<br>1st Choice: block<br>2nd Choice: 20 unstained slides (10 charged, 5µm + 10 uncharged 10 µm)	Prior to all treatment<br><br>*Preferred FFPE*	GOG Tissue Bank within 8 weeks of registration[1]
FFPE Metastatic Tumor (FM01)*<br>1st Choice: block<br>2nd Choice: 20 unstained slides (10 charged, 5µm + 10 uncharged 10 µm)	Prior to all treatment<br><br>*Optional if FP01, FRP01, or FRM01 is submitted*	
FFPE Recurrent Primary Tumor (FRP01)*<br>1st Choice: block<br>2nd Choice: 20 unstained slides (10 charged, 5µm + 10 uncharged 10 µm)	Prior to study treatment<br><br>*Optional if FP01, FM01, or FRM01 is submitted*	
FFPE Recurrent Metastatic Tumor (FRM01)*<br>1st Choice: block<br>2nd Choice: 20 unstained slides (10 charged, 5µm + 10 uncharged 10 µm)	Prior to study treatment<br><br>*Optional if FP01, FM01, or FRP01 is submitted*	
Pre-Treatment Plasma (PB01) prepared from 7-10mL of blood drawn into a **K2 EDTA tube**[2]	Prior to study treatment	GOG Tissue Bank within 1 week of registration[1]
C3D1 Pre-Treatment Plasma (PB06) prepared from 7-10mL of blood drawn into a **K2 EDTA tube**[2]	Cycle 3, day 1, prior to administering study treatment	GOG Tissue Bank within 9 weeks of registration[1]

* A copy of the corresponding pathology report must be shipped with all tissue specimens sent to the GOG Tissue Bank.
1 GOG Tissue Bank / Protocol GOG-0281, Nationwide Children's Hospital, 700 Children's Drive, WA1340, Columbus, OH 43205, Phone: (614) 722-2865, FAX: (614) 722-2897, Email: BPCBank@nationwidechildrens.org
2 **Please note the protocol-specific plasma processing instructions described in appendix IV.**

7.32 Laboratory Testing
Please refer to Appendix VII for additional background information and assay details.

	**Priority**	
**Specimen**	**1**	**2**
Fixed material	WES (7.321)	IHC (7.322)

7.321 Next-Generation Sequencing Mutational Analysis

unstained sections of tumor will be used for whole exome sequencing (WES).

7.322 Immunohistochemistry

Unstained sections of tumor will be used for immunohistochemical (IHC) analysis of pERK. IHC will be performed by Kwong Wong at MD Anderson using the CLIA-certified clinical

immunohistopathology Laboratory. All stained sections will be quality controlled. They will be initially be scanned by laboratory personnel using a low-power objective. If areas suggesting technical errors are observed, they will be further examined with higher power objectives to identify problem. Appropriate steps are then taken to resolve the problem. Acceptable slides are initialed by ASCP certified technician performing Quality Control.

7.324 Pharmacokinetics

Plasma will be used to determine trametinib pharmacokinetics in a subset of patients (approximately 12 GOG and 12 UK; collected prior to October 2017). Plasma samples collected at the following seven time points will be used for PK testing:

1. pre-treatment (PB01),
2. cycle 1, day 15, 4-8 hours post-trametinib (PB02),
3. cycle 1, day 28, pre-trametinib (PB03),
4. cycle 1, day 28, 2 hours post-trametinib (PB04),
5. cycle 2, day 1, 30 minutes post-trametinib (PB05),
6. cycle 3, day 1, pre-trametinib (PB06), and
7. cycle 3, day 1, 30 minutes post-trametinib (PB07).

Due to the limited number of complete serial plasma sets received, PK assays will not be pursued at this time.

7.33 Future Research

Details regarding the banking and use of specimens for future research can be found in Appendix IV.

7.4 Quality of Life (12/22/14)
To be submitted as a separate submission to DCP.

7.41 There are two quality of life issues of interest:

7.411 To compare Trametinib to the control arm with regard to patients' self-reported acute (up to post cycle 6) quality of life as measured by the FACT-O-TOI.

7.412 To compare Trametinib to the control arm with regards to patients' self-reported acute (up to post-cycle 6) neurotoxicity as measured by the FACT-GOG-NTX.

7.42 The primary measure used in this study to assess the quality of life is the adapted self-administered FACT-GOG-NTX, available on Scantron from NRG. Each patient will be asked to complete the questionnaire at the following time points during their participation in the study:

1. Prior to Cycle 1
2. Prior to Cycle 4 (12 weeks after starting study therapy)
3. 4 weeks post cycle 6 (24 weeks after starting study therapy)
4. 36 weeks after starting study therapy
5. 52 weeks after starting study therapy

*The times in parentheses indicate the assessment time for those who terminate treatment early.

7.43 Quality of Life Background

Rationale: A thorough review of the literature specific to the low-grade serous carcinoma of the ovary or peritoneum revealed no descriptive, retrospective or prospective quality of life data on this rare tumor. As stated above, this population differs considerably from the high-grade ovarian cancer population (e.g., indolent nature of disease, lack of response to cytotoxic therapies, younger age of onset, longer survival). As a result, one could speculate regarding the QOL similarities or dissimilarities between these variants of ovarian cancer, but specific QOL concerns or symptoms in this rare tumor population are virtually unknown, and the extent to which QOL concerns in this rare tumor population are related to disease symptoms, treatment, or their combined influence on QOL are also consequently unknown.

The previous GOG-0239 trial did not measure QOL or other Patient Reported Outcomes (PROs). However, clinical data from this trial suggest that a MEK inhibitor may, among responders, potentially reduce tumor volume as measured through traditional disease response monitoring. This may, in turn, reduce abdominal symptoms often associated with recurrent disease, which may improve QOL. It is also noteworthy that targeted therapy with a MEK inhibitor is likely to be associated with significantly less toxicity than that of the cytotoxic chemotherapies. It is less apparent, however, whether QOL for patients in the MEK inhibitor arm will be substantially different from those patients in the control arm when letrozole or tamoxifen are selected. These heretofore unexamined questions are of critical importance, in this rare, unstudied patient population in which no optimal treatment exists. Since most women with LGS ovarian cancer will survive for many months, if not years, an important goal in assessing QOL is essentially to help to determine the

optimal treatment regimen, which could indeed positively influence practice changes. In addition, systematic documentation of QOL among those enrolled onto this trial may assist in providing information to future non-trial patients regarding the expected effects of therapy as they make their treatment choices. Quality of life information will also provide an important, and perhaps the first, characterization of the health-status of women with recurrent LGS ovarian cancer, thereby alerting patients and clinicians to specific areas of concern.

8.0 EVALUATION CRITERIA

8.1 Antitumor Effect – Solid Tumors

Response and progression will be evaluated in this study using the Response Evaluation Criteria in Solid Tumors (RECIST) guideline (version 1.1) [Eur *J Ca* 45:228-247, 2009]. Changes in the largest diameter (unidimensional measurement) of the tumor lesions and the shortest diameter in the case of malignant lymph nodes are used in the RECIST criteria.

8.11 Definitions (06/08/15)

Measurable disease. Measurable lesions are defined as those that can be accurately measured in at least one dimension (longest diameter to be recorded) as ≥10 mm with CT scan, as >20 mm by chest x-ray, or >10 mm with calipers by clinical exam. All tumor measurements must be recorded in decimal fractions of centimeters.

Note: Tumor lesions that are situated in a previously irradiated area will not be considered measurable unless progression is documented or a biopsy is obtained to confirm persistence at least 90 days following completion of radiation therapy.

Malignant lymph nodes. To be considered pathologically enlarged and measurable, a lymph node must be >15 mm in short axis when assessed by CT scan (CT scan slice thickness recommended to be no greater than 5 mm). At baseline and in follow-up, only the short axis will be measured and followed.

Non-measurable disease. All other lesions (or sites of disease), including small lesions (longest diameter <10 mm or pathological lymph nodes with ≥ 10 to <15 mm short axis), are considered non-measurable disease. Leptomeningeal disease, ascites, pleural/pericardial effusions, lymphangitis cutis/pulmonitis, inflammatory breast disease, and abdominal/pelvic masses (identified by physical exam and not CT or MRI), are considered as non-measurable.

Notes: Bone lesions: Lytic bone lesions or mixed lytic-blastic lesions, with identifiable soft tissue components, that can be evaluated by CT or MRI can be considered as measurable lesions if the soft tissue component meets the definition of measurability described above. Blastic bone lesions are non-measurable.

Cystic lesions that meet the criteria for radiographically defined simple cysts should not be considered as malignant lesions (neither measurable nor non- measurable) since they are, by definition, simple cysts. ‘Cystic lesions’ thought to represent cystic metastases can be considered as measurable lesions, if they meet the definition of measurability described

above. However, if non-cystic lesions are present in the same patient, these are preferred for selection as target lesions.

Target lesions. All measurable lesions up to a maximum of 2 lesions per organ and 5 lesions in total, representative of all involved organs, should be identified as **target lesions** and recorded and measured at baseline. Target lesions should be selected on the basis of their size (lesions with the longest diameter), be representative of all involved organs, and in addition should be those that lend themselves to reproducible repeated measurements. It may be the case that, on occasion, the largest lesion does not lend itself to reproducible measurement in which circumstance the next largest lesion which can be measured reproducibly should be selected. A sum of the diameters (longest for non-nodal lesions, short axis for nodal lesions) for all target lesions will be calculated and reported as the baseline sum diameters. If lymph nodes are to be included in the sum, then only the short axis is added into the sum. The baseline sum diameters will be used as reference to further characterize any objective tumor regression in the measurable dimension of the disease.

Non-target lesions. All other lesions (or sites of disease) including any measurable lesions over and above the 5 target lesions should be identified as **non-target lesions** and should also be recorded at baseline. Measurements of these lesions are not required, but the presence, absence, or in rare cases unequivocal progression of each should be noted throughout follow-up.

8.12 Methods for Evaluation of Measurable Disease

All measurements should be taken and recorded in metric notation using a ruler or calipers. All baseline evaluations should be performed as closely as possible to the beginning of treatment and never more than 4 weeks before the beginning of the treatment.

The same method of assessment and the same technique should be used to characterize each identified and reported lesion at baseline and during follow- up. Imaging-based evaluation is preferred to evaluation by clinical examination unless the lesion(s) being followed cannot be imaged but are assessable by clinical exam.

Clinical lesions: Clinical lesions will only be considered measurable when they are superficial (e.g., skin nodules and palpable lymph nodes) and >=10 mm diameter as assessed using calipers (e.g., skin nodules). In the case of skin lesions, documentation by color photography, including a ruler to estimate the size of the lesion, is recommended.

Chest x-ray: Lesions on chest x-ray are acceptable as measurable lesions when they are clearly defined and surrounded by aerated lung. However,

CT is preferable.

Conventional CT and MRI: This guideline has defined measurability of lesions on CT scan based on the assumption that CT slice thickness is 5 mm or less. If CT scans have slice thickness greater than 5 mm, the minimum size for a measurable lesion should be twice the slice thickness. MRI is also acceptable in certain situations (e.g. for body scans), but NOT lung.

Use of MRI remains a complex issue. MRI has excellent contrast, spatial, and temporal resolution; however, there are many image acquisition variables involved in MRI, which greatly impact image quality, lesion conspicuity, and measurement. Furthermore, the availability of MRI is variable globally. As with CT, if an MRI is performed, the technical specifications of the scanning sequences used should be optimized for the evaluation of the type and site of disease. Furthermore, as with CT, the modality used at follow-up should be the same as was used at baseline, and the lesions should be measured/assessed on the same pulse sequence. It is beyond the scope of the RECIST guidelines to prescribe specific MRI pulse sequence parameters for all scanners, body parts, and diseases. Ideally, subsequent image acquisitions should use the same type of scanner and follow the baseline imaging protocol as closely as possible. If possible, body scans should be performed with breath-hold scanning techniques.

PET-CT: At present, the low dose or attenuation correction CT portion of a combined PET-CT is not always of optimal diagnostic CT quality for use with RECIST measurements. PET-CT scans are not always done with oral and IV contrast. In addition, the PET portion of the CT introduces additional data which may bias an investigator if it is not routinely or serially performed. **For these reasons, the GOG will not allow PET-CT use for RECIST 1.1 response criteria.**

FDG-PET: While FDG-PET response assessments need additional study, it is sometimes reasonable to incorporate the use of FDG-PET scanning to complement CT scanning in assessment of progression (particularly possible “new” disease). New lesions on the basis of FDG-PET imaging can be identified according to the following algorithm:

a. Negative FDG-PET at baseline, with a positive FDG-PET at follow-up is a sign of PD based on a new lesion.
b. No FDG-PET at baseline and a positive FDG-PET at follow-up: If the positive FDG-PET at follow-up corresponds to a new site of disease confirmed by CT, this is PD. If the positive FDG-PET at follow-up is not confirmed as a new site of disease on CT, additional follow-up CT scans are needed to determine if there is truly progression occurring at that site (if so, the date of PD will be the date of the initial abnormal

FDG-PET scan). If the positive FDG-PET at follow-up corresponds to a pre-existing site of disease on CT that is not progressing on the basis of the anatomic images, this is not PD.

Note: A "positive" FDG-PET scan lesion means one that is FDG avid with an uptake greater than twice that of the surrounding tissue on the attenuation corrected image.

Ultrasound: Ultrasound is not useful in assessment of lesion size and should not be used as a method of measurement. Ultrasound examinations cannot be reproduced in their entirety for independent review at a later date and, because they are operator dependent, it cannot be guaranteed that the same technique and measurements will be taken from one assessment to the next. If new lesions are identified by ultrasound in the course of the study, confirmation by CT or MRI is advised. If there is concern about radiation exposure at CT, MRI may be used instead of CT in selected instances.

Endoscopy, Laparoscopy: The utilization of these techniques for objective tumor evaluation is not advised. However, such techniques may be useful to confirm complete pathological response when biopsies are obtained or to determine relapse in trials where recurrence following complete response (CR) or surgical resection is an endpoint.

CA-125 (Ovarian, fallopian tube and primary peritoneal cancer trials): CA-125 cannot be used to assess response or progression in this study. If CA-125 is initially above the upper normal limit, it must normalize for a patient to be considered in complete clinical response. Specific guidelines for CA-125 response (in recurrent ovarian cancer) have been published [JNCI 96:487-488, 2004]. In addition, the Gynecologic Cancer Intergroup has developed CA-125 progression criteria that are to be integrated with objective tumor assessment for use only in first-line trials in ovarian cancer [JNCI 92:1534-1535, 2000].

Cytology, Histology: These techniques can be used to differentiate between partial responses (PR) and complete responses (CR) in rare cases, e.g., residual lesions in tumor types, such as germ cell tumors, where known residual benign tumors can remain.

It is mandatory to obtain cytological confirmation of the neoplastic origin of any effusion that appears or worsens during treatment when measurable disease has met criteria for response or stable disease. This confirmation is necessary to differentiate response or stable disease versus progressive disease, as an effusion may be a side effect of the treatment.

8.13 Response Criteria

Determination of response should take into consideration all target (See 8.131) and non-target lesions (See 8.132) and, if appropriate, biomarkers

(See 8.133).

8.131 Evaluation of Target Lesions

Complete Response (CR): Disappearance of all target lesions. Any pathological lymph nodes (whether target or non-target) must have reduction in short axis to <10 mm.

Partial Response (PR): At least a 30% decrease in the sum of the diameters of target lesions, taking as reference the baseline sum diameters.

Progressive Disease (PD): At least a 20% increase in the sum of the diameters of target lesions, taking as reference the smallest sum on study (this includes the baseline sum if that is the smallest on study). In addition to the relative increase of 20%, the sum must also demonstrate an absolute increase of at least 5 mm. (Note: the appearance of one or more new lesions is also considered progressions).

Stable Disease (SD): Neither sufficient shrinkage to qualify for PR nor sufficient increase to qualify for PD, taking as reference the smallest sum diameters while on study.

8.132 Evaluation of Non-Target Lesions
Complete Response (CR): Disappearance of all non-target lesions. All lymph nodes must be non-pathological in size (<10 mm short axis).

Note: If CA-25 is initially above the upper normal limit, it must normalize for a patient to be considered in complete clinical response.

Non-CR/Non-PD: Persistence of one or more non-target lesion(s)
Progressive Disease (PD): Appearance of one or more new lesions and/or *unequivocal progression* of existing non-target lesions. *Unequivocal progression* should not normally trump target lesion status. It must be representative of overall disease status change, not a single lesion increase.

Not evaluable (NE): When at least one non-target lesion is not evaluated at a particular time point.

Although a clear progression of only “non-target” lesions is exceptional, the opinion of the treating physician should prevail in such circumstances, and the progression status should be confirmed at a later time by the review panel (or Principal Investigator).

8.133 Evaluation of Biomarkers

If serum CA-125 is initially above the upper normal limit, it must normalize for a patient to be considered in complete clinical response.

Progression **cannot** be based upon biomarkers, such as serum CA-125, for this study.

8.134 Evaluation of Best Overall (unconfirmed) Response

The best overall response is the best time point response recorded from the start of the treatment until disease progression/recurrence (taking as reference for progressive disease the smallest sum recorded since **Biomarker** baseline). The patient's best response assignment will depend on the achievement of both measurement and confirmation criteria in some circumstances.

Time Point Response for Patients with Measurable Disease at baseline (i.e., Target Disease)

**Target Lesions**	**Non-Target Lesions**	**CA 125**	**New Lesions***	**Time Point Response**
CR	CR	WNL	No	CR
CR	Non-CR/Non-PD	Any value	No	PR
CR	NE	Any value	No	PR
PR	Non-PD or NE	Any value	No	PR
SD	Non-PD or NE	Any value	No	SD
NE	Non-PD	Any value	No	NE
PD	Any	Any value	Yes or No	PD
Any	PD **	Any value	Yes or No	PD
Any	Any	Any value	Yes	PD

*See RECIST 1.1 manuscript for further details on what is evidence of a new lesion.

**In exceptional circumstances, unequivocal progression in non-target lesions may be accepted as disease progression.

Time Point Response for Patients with only Non-Measurable Disease at baseline (i.e., Non-Target Disease)

**Non-Target Lesions**	**Biomarker CA 125**	**New Lesions***	**Time Point Response**
CR	WNL	No	CR

CR	Above normal limits	No	Non-CR/Non-PD*
Non-CR/Non-PD	Any value	No	Non-CR/Non-PD*
NE	Any value	No	NE
Unequivocal PD	Any value	Yes or No	PD
Any	Any value	Yes	PD

*See RECIST 1.1 manuscript for further details on what is evidence of a new lesion

** 'Non-CR/Non-PD is preferred over 'stable disease' for non-target disease since SD is increasingly used as an endpoint for assessment of efficacy in some trials so to assign this category when no lesions can be measured is not advised

Complete or partial responses may be claimed only if the criteria for each are met at a subsequent time point at least 4 weeks later.

8.14 Frequency of Tumor Re-evaluation

In the present study, tumors will be re-evaluated by CT scan of the chest, abdomen, and pelvis every 8 weeks during treatment and at least 4 weeks after the first observation of a complete or partial response. After discontinuation of protocol treatment, patients who have not progressed will still be re-evaluated every 8 weeks.

8.15 Date of progression

Date of progression is defined as the first day when the RECIST (version 1.1) criteria for PD are met. CA 125 (GCIG) progression alone is **not** sufficient for progression to be defined.

8.16 Reporting of tumor response

All patients included in the study must be assessed for response to treatment, even if there is a major protocol deviation or if they are ineligible, or not followed/re-evaluated. Each patient will be assigned one of the following categories: complete response, partial response, stable disease, progressive disease, early death from malignant disease, early death from toxicity, early death from other cause or unknown (not assessable, insufficient data).

Early death is defined as any death occurring before the first per protocol time point of tumor re-evaluation. The responsible investigator will decide if the cause of death is malignant disease, toxicity, or other cause.

Patients for whom response is not confirmed will be classified as "unknown" unless they meet the criteria for stable disease (or the criteria for partial response in case of an unconfirmed complete response). Patients' response will also be classified as "unknown" if insufficient data were collected to allow evaluation per these criteria. See table above in section 8.13 for further detail.

8.17 Duration of response

Duration of overall response: The duration of overall response is measured from the time measurement criteria are met for CR or PR (whichever is first recorded) until the first date that recurrent or progressive disease is objectively documented (taking as reference for progressive disease the smallest measurements recorded since the treatment started).

The duration of overall CR is measured from the time measurement criteria are first met for CR until the first date that progressive disease is objectively documented.

Duration of stable disease: Stable disease is measured from the date of study entry until the criteria for progression are met, taking as reference the smallest measurements recorded since the date of study entry, including the baseline measurements.

8.18 Progression-free survival

Progression-free survival (PFS) is defined as the duration of time from study entry to time of progression or death, whichever occurs first.

8.19 Overall survival

Overall survival is defined as the time from study entry to time of death or date of last contact.

9.0 DURATION OF STUDY

9.1 Patients will receive therapy until disease progression or intolerable toxicity intervenes. Patients can refuse the study treatment at any time.

9.2 All patients will be treated (with completion of all required case report forms) Until disease progression or study withdrawal. All patients will then be followed (with physical exams and histories) every three months for the first two years, then every six months for the next three years and then annually for the next 5 years. All patients will be monitored for delayed toxicity and survival for this 10 year period with Follow-up forms submitted via Medidata Rave, unless consent is withdrawn.

9.3 All patients will be followed for 10 years after removal from study or until death, whichever occurs first.

9.4 In the absence of treatment delays due to adverse event(s), treatment may continue until one of the following criteria applies:

- Disease progression
- Intercurrent illness that prevents further administration of treatment
- Unacceptable adverse event(s)
- Pregnancy (12/22/14)
- Patient decides to withdraw from the study
- General or specific changes in the patient’s condition render the patient unacceptable for further treatment in the judgment of the investigator.

10.0 STUDY MONITORING AND REPORTING PROCEDURES

10.1 ADVERSE EVENT REPORTING FOR AN INVESTIGATIONAL AGENT (CTEP IND)

10.11 Definition of Adverse Events (AE)

Adverse event (21 CFR 312.32(a)): Any untoward medical occurrence associated with the use of a drug in humans, whether or not considered drug related.

The descriptions and grading scales found in the revised NCI Common Terminology Criteria for Adverse Events (CTCAE) version 4.0 will be utilized for AE reporting. All appropriate treatment areas should have access to a copy of the CTCAE version 4.0. A copy of the CTCAE version 4.0 can be downloaded from the CTEP web site (http://ctep.cancer.gov). The CTCAE v4.0 Manual is also available on the GOG member web site (http://www.gog.org under MANUALS).

10.12 Reporting Expedited Adverse Events (07/28/14)

Depending on the phase of the study, use of investigational agents, and role of the pharmaceutical sponsor, an expedited AE report may need to reach multiple destinations. For patients participating on a GOG trial, all expedited AE reports should be submitted by using the CTEP Adverse Event Reporting System (CTEP-AERS).). All CTEP-AERS submissions are reviewed by GOG before final submission to CTEP. Submitting a report through CTEP-AERS serves as notification to GOG, and satisfies the GOG requirements for expedited AE reporting. All adverse reactions will be immediately directed to the Study Chair for further action.

The requirement for timely reporting of AEs to the study sponsor is specified in the Statement of Investigator, Form FDA-1572. In signing the FDA-1572, the investigator assumes the responsibility for reporting AEs to the NCI. In compliance with FDA regulations, as contained in 21 CFR 312.64, AEs should be reported by the investigator.

10.13 Late Phase 2 and Phase 3 Studies: Expedited Reporting Requirements for Adverse Events that Occur on Studies under a CTEP IND/IDE within 30 Days of the Last Administration of the Investigational Agent/Intervention[1, 2]

Late Phase 2 and Phase 3 Studies: Expedited Reporting Requirements for Adverse Events that Occur on Studies under a CTEP IND/IDE within 30 Days of the Last Administration of the Investigational Agent/Intervention [1, 2]

FDA REPORTING REQUIREMENTS FOR SERIOUS ADVERSE EVENTS (21 CFR Part 312) (07/28/14)

NOTE: Investigators **MUST** immediately report to the sponsor (NCI) **ANY** Serious Adverse Events, whether or not they are considered related to the investigational agent(s)/intervention (21 CFR 312.64)

An adverse event is considered serious if it results in **ANY** of the following outcomes:

1) Death
2) A life-threatening adverse event
3) An adverse event that results in inpatient hospitalization or prolongation of existing hospitalization for ≥ 24 hours
4) A persistent or significant incapacity or substantial disruption of the ability to conduct normal life functions
5) A congenital anomaly/birth defect.
6) Important Medical Events (IME) that may not result in death, be life threatening, or require hospitalization may be considered serious when, based upon medical judgment, they may jeopardize the patient or subject and may require medical or surgical intervention to prevent one of the outcomes listed in this definition. (FDA, 21 CFR 312.32; ICH E2A and ICH E6).

ALL SERIOUS adverse events that meet the above criteria **MUST** be immediately reported to the NCI via CTEP-AERS within the timeframes detailed in the table below.

Hospitalization	Grade 1 Timeframes	Grade 2 Timeframes	Grade 3 Timeframes	Grade 4 & 5 Timeframes
Resulting in Hospitalization ≥ 24 hrs	7 Calendar Days			24-Hour 3 Calendar Days
Not resulting in Hospitalization ≥ 24 hrs	Not required		7 Calendar Days	

NOTE: Protocol specific exceptions to expedited reporting of serious adverse events are found in the Specific Protocol Exceptions to Expedited Reporting (SPEER) portion of the CAEPR

Expedited AE reporting timelines are defined as:

- "24-Hour; 3 Calendar Days" - The AE must initially be reported via CTEP-AERS within 24 hours of learning of the AE, followed by a complete expedited report within 3 calendar days of the initial 24-hour report.
- "7 Calendar Days" - A complete expedited report on the AE must be submitted within 7 calendar days of learning of the AE.

[1]Serious adverse events that occur **more than** 30 days after the last administration of investigational agent/intervention and have an attribution of possible, probable, or definite require reporting as follows:

Expedited 24-hour notification followed by complete report within 3 calendar days for:

- All Grade 4, and Grade 5 AEs

Expedited 7 calendar day reports for:

- Grade 2 adverse events resulting in hospitalization or prolongation of hospitalization
- Grade 3 adverse events

[2] For studies using PET or SPECT IND agents, the AE reporting period is limited to 10 radioactive half lives, rounded UP to the nearest whole day, after the agent/intervention was last administered. Footnote "1" above applies after this reporting period.

Additional Instructions or Exceptions to CTEP-AERS Expedited Reporting Requirements:

There are no additional instructions or exceptions to CTEP-AERS expedited reporting requirements for this protocol.

10.14 Procedures for Expedited Adverse Event Reporting: (07/28/14)

10.141 CTEP-AERS Expedited Reports: Expedited reports are to be submitted using CTEP-AERS available at http://ctep.cancer.gov. The CTEP, NCI Guidelines: Adverse Event Reporting Requirements for expedited adverse event reporting requirements are also available at this site.

AML/MDS events must be reported via CTEP-AERS (in addition to routine AE reporting mechanisms). In CTCAE v4.0, the event(s) may be reported as either: 1) Leukemia secondary to oncology chemotherapy, 2) Myelodysplastic syndrome, or 3) Treatment related secondary malignancy. Any malignancy possibly related to cancer treatment (including AML/MDS) should also be reported via the routine reporting mechanisms outlined in each protocol.

Second Malignancy:

A second malignancy is one unrelated to the treatment of a prior malignancy (and is **NOT** a metastasis from the initial malignancy). Second malignancies require **ONLY** routine reporting via CDUS unless otherwise specified.

The Comprehensive Adverse Event and Potential Risks list (CAEPR) provides a single list of reported and/or potential adverse events (AE) associated with an agent using a uniform presentation of events by body system. In addition to the comprehensive list, a subset of AEs, the Specific Protocol Exceptions to Expedited Reporting (SPEER), appears in a separate column and is identified with ***bold*** and ***italicized*** text. The SPEER is a list of events that are protocol-specific exceptions to expedited reporting to NCI via CTEP-AERS (except as noted below). Refer to the 'CTEP, NCI Guidelines: Adverse Event Reporting Requirements' http://ctep.cancer.gov/protocolDevelopment/default.htm#adverse_events_CTEP-AERS for further clarification.

In the rare event when Internet connectivity is disrupted a 24-hour notification is to be made to NCI by telephone at: 301-897-7497. An electronic report MUST be submitted immediately upon re-establishment of internet connection. Please note that all paper CTEP-AERS forms have been removed from the CTEP website and will NO LONGER be accepted.

10.2 ADVERSE EVENT REPORTING FOR A COMMERCIAL AGENT (06/08/15)

10.21 Definition of Adverse Events (AE)

An adverse event (AE) is any unfavorable and unintended sign (including an abnormal laboratory finding), symptom, or disease that occurs in a patient administered a medical treatment, whether the event is considered related or unrelated to the medical treatment.

The descriptions and grading scales found in the revised NCI Common Terminology Criteria for Adverse Events (CTCAE) version 4.0 will be utilized for AE reporting. All appropriate treatment areas should have access to a copy of the CTCAE version 4.0. A copy of the CTCAE version 4.0 can be downloaded from the CTEP web site (http://ctep.cancer.gov). The CTCAE v4.0 Manual is also available on the GOG member web site (http://www.gog.org under MANUALS).

10.22 Reporting Expedited Adverse Events

Depending on the phase of the study, use of investigational or commercial agents, and role of the pharmaceutical sponsor, an AE report may need to reach multiple destinations. For patients participating on a GOG trial, all expedited AE reports should be submitted by using the CTEP automated system for expedited reporting (CTEP-AERS). All CTEP-AERS submissions are reviewed by GOG before final submission to CTEP-AERS. Submitting a report through CTEP-AERS serves as notification to GOG, and satisfies the GOG requirements for expedited AE reporting. All adverse reactions will be immediately directed to the Study Chair for further action.

The requirement for timely reporting of AEs to the study sponsor is specified in the Statement of Investigator, Form FDA-1572. In signing the FDA-1572, the investigator assumes the responsibility for reporting AEs to the NCI. In compliance with FDA regulations, as contained in 21 CFR 312.64, AEs should be reported by the investigator.

10.23 Phase 2 and 3 Trials Utilizing a Commercial Agent: CTEP-AERS Expedited Reporting Requirements for Adverse Events That Occur Within 30 Days of the Last Dose of Any Commercial Study Agent

Reporting Requirements for Adverse Events that occur within 30 Days[1] of the Last Dose of the Commercial Agent on Phase 2 and 3 Trials

	**Grade 1**	**Grade 2**	**Grade 2**	**Grade 3**		**Grade 3**		**Grades 4 & 5[2]**	**Grades 4 & 5[2]**
				**Unexpected**		**Expected**			
	**Unexpected and Expected**	**Unexpected**	**Expected**	**With Hospitali-zation**	**Without Hospitali-zation**	**With Hospitali-zation**	**Without Hospitali-zation**	**Unexpected**	**Expected**
**Unrelated Unlikely**	Not Required	Not Required	Not Required	7 Calendar Days	Not Required	7 Calendar Days	Not Required	7 Calendar Days	7 Calendar Days
**Possible Probable Definite**	Not Required	7 Calendar Days	Not Required	7 Calendar Days	7 Calendar Days	7 Calendar Days	Not Required	24-Hrs; 3 Calendar Days	7 Calendar Days

[1] Adverse events with attribution of possible, probable, or definite that occur greater than 30 days after the last dose of treatment with a commercial agent require reporting as follows:

CTEP-AERS 24-hour notification followed by complete report within 3 calendar days for:
- Grade 4 and Grade 5 unexpected events

CTEP-AERS 7 calendar day report:
- Grade 3 unexpected events with hospitalization or prolongation of hospitalization
- Grade 5 expected events

[2] Although an CTEP-AERS 24-hour notification is not required for death clearly related to progressive disease, a full report is required as outlined in the table.

Please see exceptions below under the section entitled, "Additional Instructions or Exceptions to CTEP-AERS Expedited Reporting Requirements for Phase 2 and 3 Trials Utilizing a Commercial Agent." **March 2005**

Note: All deaths on study require both routine and expedited reporting regardless of causality. Attribution to treatment or other cause must be provided.

- Expedited AE reporting timelines defined:
  - "24 hours; 3 calendar days" – The investigator must initially report the AE via CTEP-AERS within 24 hours of learning of the event followed by a complete CTEP-AERS report within 3 calendar days of the initial 24-hour report.

  "7 calendar days" – A complete CTEP-AERS report on the AE must be submitted within 7 calendar days of the investigator learning of the event.

- Any medical event equivalent to CTCAE grade 3, 4, or 5 that precipitates hospitalization (or prolongation of existing hospitalization) must be reported regardless of attribution and designation as expected or unexpected with the exception of any events identified as protocol-specific expedited adverse event reporting exclusions.

- Any event that results in persistent or significant disabilities/incapacities, congenital anomalies, or birth defects must be reported to GOG via CTEP-AERS if the event occurs following treatment with a commercial agent.

- Use the NCI protocol number and the protocol-specific patient ID provided during trial registration on all reports.

Additional Instructions or Exceptions to CTEP-AERS Expedited Reporting Requirements for Phase 2 and 3 Trials Utilizing a Commercial Agent:

- *[Include any applicable instructions or exceptions here. For example, "All Grade 2 and 3 myelosuppression (including neutropenia, anemia, and thrombocytopenia) that does not require hospitalization is exempt from expedited reporting.*"

- *[Include the following statement if there are no exceptions]* There are no additional instructions or exceptions to CTEP-AERS expedited reporting requirements for this protocol.

10.24 Procedures for Expedited Adverse Event Reporting:

10.241 CTEP-AERS Expedited Reports: Expedited reports are to be submitted using CTEP-AERS available at http://ctep.cancer.gov. The CTEP, NCI Guidelines: Adverse Event Reporting Requirements for expedited adverse event reporting requirements are also available at this site.

AML/MDS events must be reported via CTEP-AERS (in addition to routine AE reporting mechanisms). In CTCAE v4.0, the event(s) may be reported as either: 1) Leukemia secondary to oncology chemotherapy, 2) Myelodysplastic syndrome, or 3) Treatment related secondary malignancy.

In the rare event when Internet connectivity is disrupted a 24-hour notification is to be made to GOG by telephone at: 215-854-0770. An electronic report MUST be submitted immediately upon re-establishment of internet connection. Please note that all paper CTEP-AERS forms have been removed from the CTEP website and will NO LONGER be accepted.

10.25 Automated CDUS reporting

For studies using commercial agents, the GOG Statistical and Data Center (SDC) routinely reports adverse events electronically to the CTEP Clinical Data Update System (CDUS Version 3.0). The SDC submits this data quarterly. The AEs reported through CTEP-AERS will also be included with the quarterly CDUS data submissions.

10.3 Medidata Rave Data Submission and Reporting (07/28/14)(03/21/16)**(02-JUN-2020)**

Medidata Rave is a clinical data management system being used for data collection for this trial/study. Access to the trial in Rave is controlled through the CTEP-IAM system and role assignments.

Requirements to access Rave via iMedidata:

- A valid CTEP-IAM account; and
- Assigned a Rave role on the LPO or PO roster at the enrolling site of: Rave CRA, Rave Read Only, Rave CRA (LabAdmin), Rave SLA, or Rave Investigator.

Rave role requirements:

- Rave CRA or Rave CRA (Lab Admin) role must have a minimum of an Associate Plus (AP) registration type;
- Rave Investigator role must be registered as an Non-Physician Investigator (NPIVR) or Investigator (IVR); and
- Rave Read Only role must have at a minimum an Associates (A) registration type.

Refer to https://ctep.cancer.gov/investigatorResources/default.htm for registration types and documentation required.

This study has a Delegation of Tasks Log (DTL). Therefore, those requiring write access to Rave must also be assigned the appropriate Rave tasks on the DTL.

Upon initial site registration approval for the study in Regulatory Support System (RSS), all persons with Rave roles assigned on the appropriate roster will be sent a study invitation e-mail from iMedidata. To accept the invitation, site staff must log in to the Select Login (https://login.imedidata.com/selectlogin) using their CTEP-IAM username and password and click on the *accept* link in the upper right-corner of the iMedidata page. Site staff will not be able to access the study in Rave until all required Medidata and study specific trainings are completed. Trainings will be in the form of electronic learnings (eLearnings) and can be accessed by clicking on the link in the upper right pane of the iMedidata screen. If an eLearning is required and has not yet been taken, the link to the eLearning will appear under the study name in iMedidata instead of the *Rave EDC* link; once the successful completion of the eLearning has been recorded, access to the study in Rave will be granted, and a *Rave EDC* link will display under the study name.

Site staff that have not previously activated their iMedidata/Rave account at the time of initial site registration approval for the study in RSS will receive a separate invitation from iMedidata to activate their account. Account activation instructions are located on the CTSU website in the Data Management section

under the Rave resource materials (Medidata Account Activation and Study Invitation Acceptance). Additional information on iMedidata/Rave is available on the CTSU members' website in the Data Management > Rave section at www.ctsu.org/RAVE/ or by contacting the CTSU Help Desk at 1-888-823-5923 or by e-mail at ctsucontact@westat.com.

10.4 GOG DATA MANAGEMENT FORMS (06/08/15)

The following forms must be completed for all patients registered and submitted to the GOG Statistical and Data Center (SDC) according to the schedule below. GOG electronic case report forms must be submitted through the Medidata Rave Electronic Data Entry System (www.imedidata.com). All amendments to forms must also be submitted through Medidata Rave. The operative report, discharge summary and pathology reports can be sent to the GOG Statistical and Data Center via postal mail or uploaded in Medidata Rave. The upload option is an alternative method for submitting paper reports. Quality of Life questionnaires are to be completed on the original Scantron forms and submitted via postal mail

Form	Comments
**Baseline Folder**<br>***(Forms due within 2 weeks of registration)***	
Baseline/History Forms:<br>- Visit Information – Baseline Form<br>- Registration Form<br>- History Information Form<br>- Primary Surgery Form<br>- Chemotherapy Information Form<br>- Pre-Treatment Summary Form<br>- Specimen Consent<br>- Vitals Form<br>- ECHO/MUGA Form<br><br>Solid Tumor Evaluation Forms:<br>- Target Lesions Form<br>- Non-Target Lesions Form	The appropriate forms will load in the Baseline Folder based on the answers reported on the corresponding Baseline Visit Information form.

Form	Comments
**Visit Folder**<br>***(Forms due within 2 weeks of the completion of each cycle)***	
Cycle Information and Treatment Forms:<br>- Visit Information Form<br>- Cycle Patient Information Form<br>- Cycle Drug Information Form<br>- Labs and Chemistries Form<br>- Vitals Form<br>- ECHO/MUGA Form<br><br>Toxicity Forms:<br>- Section 1 Form<br>- NADIRS Form<br>- Adverse Event Form<br>- Adverse Event Grades<br>Solid Tumor Evaluation Forms:<br>- Target Lesions Form<br>- No Target lesion Form<br>- Non-Target Form<br>- New Target Lesions Form<br>- Status and Response Form	The appropriate forms will load in the Visit Folder based on the answers reported on the corresponding Visit Information forms.
**Pathology Folder**<br>***(Reports due within 6 weeks of registration)***	
Primary disease:<br>Pathology Report<br><br>Recurrent or Persistent Disease:<br>Pathology Report	Submit one copy of the pathology report to SDC via postal mail or upload the pathology report online via RAVE.<br><br>Stained pathology slides are required for prospective digital review See Section 7.11 for mailing instructions.

Form	Comments
**Translational Research Folder** (11/27/17)	
TR Forms:<br>- Snap Frozen Recurrent Primary Biopsy (RRP01)*<br>- Snap Frozen Recurrent Metastatic Biopsy (RRM01)*<br>- FFPE Primary Tumor (FP01)<br>- FFPE Metastatic Tumor (FM01) *optional*<br>- FFPE Recurrent Primary Tumor (FRP01) *optional*<br>- FFPE Recurrent Metastatic Tumor (FRM01) *optional*<br>- Pre-Treatment Plasma (PB01)<br>- C1D15 4-8 Hour Post-Treatment Plasma (PB02)*<br>- C1D28 Pre-Treatment Plasma (PB03)*<br>- C1D28 2 Hour Post-Treatment Plasma (PB04)*<br>- C2D1 30 Minute Post-Treatment Plasma (PB05)*<br>- C3D1 Pre-Treatment Plasma (PB06)<br>- C3D1 30 Minute Post-Treatment Plasma (PB07)*	A completed copy of Form TR must accompany each specimen shipped to the GOG Tissue Bank (or alternate laboratory). Handwritten forms will not be accepted.<br><br>FP01, FM01, FRP01, and FRM01 are due 8 weeks from registration.<br><br>RRP01 or RRM01 and PB01 are due 1 week from registration.<br><br>PB02-PB07* are due 9 weeks from registration.<br><br>*RRP01, RRM01, PB02-PB05, and PB07 are no longer required as of October 2017, and should not be collected.
**Quality of Life**	
Scantron Form	Submit Original Scantron Form to SDC via postal mail
**Treatment Completion Folder**<br>***(Forms due within 2 weeks of treatment completion)***	
Treatment Completion Form	
**Follow-up Visit Folder**<br>***(Forms due within 2 weeks of follow-up visits, disease progression or death)***	
Visit Information Follow-Up Form<br><br>Follow-Up Form<br><br>Follow-Up Period Adverse Event:<br>- Reporting Form – Part 1<br>- Reporting Form – Part 2	Follow-up visits should be scheduled quarterly for 2 years, semi-annually for 3 more years, and annually thereafter.<br><br>The appropriate forms will in the Follow-up Visit Folder based on the answers reported on the corresponding Follow-up Visit Information forms.

Solid Tumor Evaluation:<br>- Target Lesions Form<br>- No Target lesion Form<br>- Non-Target Form<br>- New Target Lesions Form<br>- Status and Response Form	

This study will be monitored by the **Abbreviated** Clinical Data System (CDUS) Version 3.0 CDUS data will be submitted quarterly **by the January 31, April 30, July 31 and October 31 due dates** to CTEP by electronic mean.

11.0 STATISTICAL CONSIDERATIONS

11.1 Design Summary

This is an unblinded, randomized phase II/III study of comparing trametinib to "standard therapy" (consisting of one of five commercially available agents) in patients with low-grade serous carcinoma of the ovary or peritoneum previously treated with platinum-based chemotherapy treated with in women with recurrent low-grade serous.

Patients will be randomized in a 1:1 ratio to receive Trametinib or standard of care (the control arm). The randomization will be stratified—using minimization—by the following factors: geographic region (UK or US), performance status (0 or 1), number of prior treatment regimens, and planned treatment regimen (if in the control arm).

All patients will be registered centrally via OPEN. Prior to registration, eligibility will be reviewed via Eligibility check list verification. All reports will include a complete accounting of all patients registered to this protocol.

All reports will include an accounting of all patients registered onto the study, regardless of their eligibility status or compliance to the assigned treatment.

All analyses will be done on the data combined from the US and UK. This is one, international study. The data will be collected in the GOG database but will be transferred to the UK for analysis.

11.2 Principal Parameters

The principle parameters employed to evaluate the primary, secondary, and translational research objectives are:

11.21 Primary efficacy endpoint: progression-free survival

11.22 Secondary endpoints

11.221 Adverse events assessed using CTCAE version 4

11.222 Overall survival

11.223 Objective tumor response rate

11.224 Quality of Life as measured by FACT and FACT-NTX

11.23 Translational Research Endpoints:

11.231 Mutations of genes in the MAPK pathway

11.232 Protein levels of pERK

11.3 Accrual Goal, Accrual Rate, and Study Duration

The targeted accrual for this study is 250 patients (125 per arm). The accrual rate for this same patient population in GOG-0239 was 3 patients per month. We project an accrual rate of 5.5 patients per month, and project half the accrual to be from the US and half to be from the UK. The higher rate here compared to GOG-0239 is due to (i) the participation of NCRN institutions in the UK, and (ii) this is a randomized phase II/III trial, in which more institutions are likely to participate. Women with low-grade serous carcinoma have a significantly better prognosis than those with typical high-grade ovarian cancers although they recur at approximately the same rate. Thus, this patient cohort typically receives multiple sequential chemotherapy and hormonal therapy regimens.

We expect to accrue 250 patients over 46 months, with a follow-up period of 10 months to provide the requisite number of events, which is specified below.

This randomized study will follow CTEP slow accrual Guidelines; for this, the total accrual (GOG and International) will be considered.

Participation in the study by non-US, non-NCTN sites will require an official amendment with approval by CTEP.

11.4 Design and Analyses for the Primary Objective

This is as a two-arm randomized phase II/III study. The primary endpoint is progression-free survival (PFS). The null and alternative hypotheses with regard to the relative PFS event rate for the trametinib versus standard of care arms are as follows:

$$H_0: \Delta=\lambda_T / \lambda_S = 1.0 \text{ vs. } H_1: \Delta=\lambda_T / \lambda_S < 1.0$$

where λ_T and λ_S are the PFS hazards in the trametinib and standard of care arms, respectively.

PFS is being used as the primary endpoint as opposed to survival in the study because of the relatively long overall survival in this patient population.

The primary analyses of PFS will include all patients enrolled onto the study regardless of eligibility or compliance to their assigned study regimen. Patients will be grouped by their randomized treatment for intention-to-treat analyses (ITT).

In order to have 80% power to detect a 50% improvement in PFS (from 8 to 12 months) and using a one-sided 2.5% significance level at the final analysis, 213 events are required. A sample size of 250 patients accrued over 46 months, with a follow-up period of 10 months should provide this number of events. The final

analysis will occur when at least 112 events (deaths or progressions) are observed in the control arm (which is the expected number to occur with 213 events total and a 33% reduction in the hazard for the experimental arm). The analysis will be based on a log rank test stratified by geographic region (UK or US), performance status (0 or 1), and planned treatment regimen (in the control arm). The study includes an interim analysis for futility, which is described below. (When the trial's futility analysis is considered, the overall type I error of the trial is 0.022.)

11.5 Interim Analysis (11/27/17)

The phase II portion of the trial corresponds to results prior to the interim, futility analysis, which has an aggressive stopping rule. The futility analysis will occur when 58 PFS events are observed in the either arm (which is the expected number to occur within the control arm when there are 106 events total, i.e., 50% information, and a 33% reduction in the hazard for the experimental arm). This is expected to occur after approximately 32 months of accrual. A Rho family spending function with a parameter of 0.95 will be used [32]. If the test statistic from the log-rank test is <0.826, the trial will be stopped for futility. Under the null hypothesis, the probability of stopping for futility is 0.796, and under the alternative hypothesis, the probability is 0.104 (i.e., 0.104 of the 0.20 beta is spent at the interim analysis).

The results of interim analyses will be examined by an independent data monitoring committee, made up of representatives from GOG and the UK. The decision to terminate accrual will include consideration of toxicities, treatment compliance, and progression-free survival and other endpoints, and results from external studies.

The trial will not be suspended for the interim analysis.

Expansion of the study from the phase 2 portion to the phase 3 portion of the study will require an official amendment with approval by CTEP.

The results of the interim futility analysis indicated that the trial could go forward to the phase III portion of the study, and the NRG Data Monitoring Committee (DMC) recommended doing so in a meeting on July 13, 2017. In addition, no new safety signals were seen during this (or previous) reviews.

11.6 Monitoring Adverse Events and the Role of the GOG DSMB

The GOG Data Safety and Monitoring Board (DSMB) reviews accumulating summaries of toxicities and all serious adverse event (SAE) reports on an ongoing basis (not efficacy results). This committee also reviews those deaths in which study treatment may have been a contributing cause. The DSMB reports to the DMC and it may recommend study amendments pertaining to patient safety. Data sheets from patients on this protocol will be reviewed before each semi-annual meeting and will also be reviewed by the Study Chair in conjunction with the

Statistical and Data Center. In some instances, because of unexpectedly severe toxicity, the Statistical and Data Center may elect, after consultation with the Study Chair and the Medical Oncology Committee, to recommend early suspension of a study.

The frequency and severity of all toxicities are tabulated from submitted case report forms and summarized for review by the study chair, Ovarian Committee, and GOG Data Safety and Monitoring Board (DSMB) in conjunction with each semi-annual GOG meeting. For studies sponsored by the Cancer Therapy Evaluation Program (CTEP) of
The National Cancer Institute (NCI), standardized toxicity reports are also submitted to the drug and disease monitors at the Investigational Drug Branch (IDB) and Clinical Investigation Branch (CIB).

11.7 Analyses for Secondary Objectives
All tests for secondary objectives will be done using one-sided tests with $\alpha=0.05$.

Safety Analyses
The National Cancer Institute (NCI) Common Terminology Criteria for Adverse Events (CTCAE) criteria version 4.0 will be used to classify adverse events (AEs) observed during treatment. The severity of each AE will be assessed according to the NCI CTCAE 4.0 grading system. Patients will be tabulated according to their maximum severity for each organ system or preferred term.

Safety endpoints will be summarized with descriptive statistics for the patients in the safety analysis dataset. The safety analysis dataset will include all patients enrolled to the study who receive any of their assigned study treatment, and patients will be grouped by their assigned treatment. Patients who do not receive any of their assigned study treatment will not be included in these analyses.

Analyses of Overall Survival
Secondary endpoints include overall survival (OS) and tumor response. With the projected accrual above, and assuming a median overall survival of 30 months in the control arm, the study has 80% power to detect a 33% improvement in median OS from 30 to 40 months at a one-sided 25% significance level. The analysis of overall survival will be based on a log-rank test.

Overall survival will be characterized by treatment group with Kaplan-Meier plots and estimates of the median time until death. The log rank test[33] will be used to compare treatment groups with respect to overall survival. Patients will **not** be censored at the time of crossover. We note that analyses of overall survival will be problematic (and biased in undetermined ways) due to the crossover allowed at progression. Therefore, these analyses must be considered in this light. Prior to the analysis, other potential analysis methods (e.g., inverse probability weighting[37]) will be considered and pre-specified.

Response rates (Complete and Partial combined) will be compared between the arms using logistic regression via a model incorporating study arm and the factors used in the minimization algorithm. The p-value associated with study arm will be obtained from this model. An 80% confidence interval for the odds ratio will be provided.

Analyses of Quality of Life

The Functional Assessment of Cancer Therapy scale developed for ovarian cancer (FACT-O TOI) is a tool that provides a general QoL score. It consists of 3 subscales: physical well-being (7 items), functional well-being (7 items) and the Ovarian Cancer subscale (12 items). The FACT-GOG/NTX4 is a four-item subscale which assesses patient reported platinum/taxane-induced symptoms of neuropathy.

Hypotheses and analyses

QOL Objective 1: To compare the MEK inhibitor to the "cytotoxic standard therapy" (i.e., PLD, weekly topotecan or weekly paclitaxel) on patients' self-reported acute (up to post-cycle 6) quality of life as measured by the FACT-O-TOI.

QOL Hypothesis 1: Patients' randomized to the MEK inhibitor treatment arm will report significantly better acute QOL compared to patients randomized to the cytotoxic standard therapy arm.

QOL Objective 2: To compare the MEK inhibitor to the "cytotoxic standard therapy" (i.e., PLD, weekly topotecan or weekly paclitaxel) on patients' self-reported acute (up to post-cycle 6) neurotoxicity as measured by the FACT-GOG-NTX.

QOL Hypothesis 2: Patients' randomized to the MEK inhibitor treatment arm will report significantly less acute neurotoxicity compared to patients randomized to the cytotoxic standard therapy arm.

QOL Objective 3: To compare the MEK inhibitor to the "endocrine standard therapy" (i.e., letrozole, tamoxifen) with respect to patients' self-reported acute quality of life and neurotoxicity, as measured by the FACT-O-TOI, and FACT-GOG-NTX, respectively.

QOL Hypothesis 3: Patients' randomized to the MEK inhibitor treatment arm will report significantly better acute QOL compared to patients randomized to the endocrine standard therapy arm.

The primary QoL hypothesis will be examined with a mixed model, adjusting for pretreatment TOI score, age, and treatment option (PLD, weekly topotecan, or weekly paclitaxel) and using post-baseline scores from pre-cycle 4 and post-cycle 6. Patients will be included in these analyses regardless of the amount of study treatment they received. For the primary analyses, patients will be categorized by their randomized treatment assignment rather than the treatment received.

Analyses which classify patients by the actual treatment they received will be considered exploratory.

The second QoL hypothesis will be examined as above but the FACT-NTX.

The third QoL hypothesis will be examined as above but with the treatment options restricted to letrozole or tamoxifen.

Analyses of all four post-baseline QoL assessments will be considered exploratory.

Multiplicity of Outcomes
The overall type I error for the two primary QoL hypotheses (1 and 2) will be controlled at $\alpha=0.10$ by using a Bonferroni adjustment: each will be tested at $\alpha=0.0513$. The overall alpha for the QoL hypotheses will be controlled by testing the secondary hypothesis (3) only if one or both of the two primary hypotheses are rejected. All tests will be two-sided.

Statistical Power Considerations
Based on a trial of GOG of 415 patients with advanced ovarian cancer were treated with platinum and paclitaxel for 6 cycles every 21 days, we estimate the standard deviation (SD) of the FAC-TOI scores to be approximately 15. We roughly project the following numbers of patients will receive each of the following standard of care treatments (with equal numbers to the experimental therapy in each strata)

- Letrozole: 50
- Tamoxifen: 25
- Taxol: 25
- PLD: 20
- Topotecan: 5

This would yield 25 patients per treatment arm for the primary QoL hypothesis, and 100 patients per treatment arm for the secondary hypothesis.

The table below shows the between-treatment difference that is detectable with 90% power, given the sample sizes shown per arm, and assuming a SD=15 (and two-sided $\alpha=0.0513$).

N per arm	Detectable Difference	
	With 80% power	With 90% power
25	12.1	14.0
50	8.5	9.8
75	6.9	8.0
100	5.9	6.9

11.8 Analyses for Integrated Biomarker Objectives

Secondary Objectives 1.24 and 1.25 consider the prognostic and predictive abilities of several biomarkers relative to Objective Response Rate (ORR, section 8.16) or progression free survival (section 8.16, 8.18). The biomarkers of interest are:

1. (Objective 1.24) pERK expression, quantified using the H-Score (page 117) derived from IHC analysis of patient tumor tissue. The H-Score is expected to present as a continuous measure.
2. (Objective 1.25) Markers of genotype status within the MAPK pathway, quantified by Whole Exome Sequencing (WES). WES output includes indicators of
   a. single nucleotide polymorphisms (SNPs)
   b. multiple nucleotide polymorphisms (MNPs)
   c. insertions (INS)
   d. deletions (DEL)

   These measures are expected to present as dichotomous factors. When 3 or more levels are presented, the modeling will likely require collapsing the analysis variable down to 2 biologically rational levels.

Notation and Definitions

Let $\lambda_{i,j}$ denote the true odds of response or PFS hazard associated with marker expression i ϵ {High(H), Low (L)} receiving randomized treatment j ϵ {Trametinib(T), Control(C)}. For continuous markers, $\lambda_{i,j}$ represents a one-unit difference in the expression measurement. We assume that higher expression levels correspond to better prognosis.

Then let $\delta_{\bullet j} = \lambda_{H,j} / \lambda_{L,j}$ denote the true odds- or hazard ratio related to high (ref: low) marker expression among patients randomized to treatment = (j). Similarly, let $\delta_{i\bullet} = \lambda_{i,T} / \lambda_{i,C}$ denote the true odds- or hazard- ratio related to trametinib (ref: control) among patients with marker expression = (i).

Prognostic Hypothesis Testing and Statistical Power

A prognostic marker is expected to provide information on the clinical outcome of patients receiving the standard therapy.[34,35] The null and alternative hypotheses for a prognostic marker are

$$H_0: \delta_{\bullet C} = \lambda_{H,C} / \lambda_{L,C} = 1.0 \quad \text{vs} \quad H_A: \delta_{\bullet C} \neq 1.0$$

The design parameters in Section 11.3-11.4 suggest the dataset supporting the prognostic marker analyses will include 125 patients randomized to the control arm, with 100 PFS events.

The power of the proportional hazards and logistic regression models is a function of the proportion of patients with evaluable tumor tissue, the distribution of the marker within the evaluable patient set and the number of events or responses.[36] The following tables summarize the biomarker effect that can be detected by a 2-sided, with α=0.05 test with 80% power, given assumptions for the size of the evaluable patient set. These estimates assume no impact from the multivariable covariate adjustment.

Proportion with Favorable Marker	# Evaluable Events		
	100	80	60
50%	0.571	0.534	0.485
35%	0.556	0.519	0.468
20%	0.496	0.457	0.405

PFS HAZARD RATIOS

Shows the maximum detectable hazard ratio (MDHR) associated with favorable (vs unfavorable) biomarker expression. Differences in PFS are tested using a logrank test with 2-sided alpha=0.05, power=0.80. If all patients have sufficient tissue, the analysis is supported by 100 PFS events. The MDHR decreases with as the number of evaluable patients decreases, or the prevalence of favorable expression decreases.

OBJECTIVE RESPONSE RATES

Proportion with Favorable Marker	# Evaluable Patients		
	120	100	80
50%	0.647	0.669	0.697
35%	0.657	0.679	0.707
20%	0.698	0.720	0.749

(Response rate for Unfavorable Marker = 0.400)

Shows the minumum detectable Objective Response Rate (MDORR) among patient with favorable biomarker expression. Proportions are compared using a z-test with 2-sided alpha=0.05, power=0.80. If all patients have sufficient tissue, the analysis is supported by 120 patients. The MDORR increases as the number of evaluable patients decreases, or the prevalence of favorable expression decreases.

Prognostic Effect Estimation

The prognostic models will be supported by patients randomized to the control arm, excluding information collected after crossover to trametinib.

The prognostic hazard ratio and odds ratio estimates related to each marker/outcome combination will be estimated by multivariable proportional hazards or logistic regression models, with covariate adjustment for the main effects of geographic region, performance status and number of prior regimens (the stratification factors in Section 11.2). If the marker-effect term from this model has p-value < 0.05, the marker will be considered prognostic, and interesting for future validation studies.

Similar methods will support exploratory analyses among patients randomized to trametinib therapy.

Predictive Hypothesis Testing and Statistical Power

Predictive markers are expected to provide information on the likelihood of response to a particular treatment. The predictive hypothesis considers whether the treatment effect depends on marker status. As shown by Peterson,[37] the null and alternative hypotheses for a predictive marker are

$$H_0: \delta_{H\bullet} = \delta_{L\bullet} \quad \text{vs} \quad H_A: \delta_{H\bullet} \neq \delta_{L\bullet}$$

Gail (1985) assumes the marker effect estimates in the treatment subsets ($\delta_{H\bullet}$, $\delta_{L\bullet}$) are independent, suggesting these treatment effect estimates should be obtained from separate, marker-specific models.[38] Peterson shows the z-test statistic implied by this prediction hypothesis simplifies to

$$ln\left[\widehat{\frac{\delta_{H\cdot}}{\delta_{L\cdot}}}\right] \sim N\left(ln\left[\frac{\delta_{H\cdot}}{\delta_{L\cdot}}\right], \sqrt{\frac{1}{D_{HT}} + \frac{1}{D_{HC}} + \frac{1}{D_{LT}} + \frac{1}{D_{LC}}}\right)$$

where D_{HT} is the expected number of events among the high marker trametinib patients.[37]

The power/effect size relationship shown in the table below considers the simplified scenario where the treatment effect is limited to the high marker patients, i.e $\delta_{L\bullet}$=1.0 throughout. High

marker status has a small prognostic effect ($\delta_{\bullet C}$ = 0.83, or a 3-month improvement in PFS in control patients with high marker expression). These assumptions focus the predictive effect into the high marker patients treated with trametinib. The table shows the treatment effect estimate among marker high patients. A 2-sided, α=0.05 z-test has 80% power to detect the hazard ratios shown below, assuming no impact from the multivariable covariate adjustment.

HAZARD RATIOS (Tram/Control, Marker=High)

Evaluable Patients	% Marker High 50%	35%	20%
**250**	0.396	0.359	0.250
**200**	0.347	0.306	0.182
**150**	0.280	0.232	N/A

Shows the maximum detectable hazard ratio (MDHR) associated with Trametinib (vs control) in patients with high marker expression. The treatment HR in low marker patients is set to =1. The ratio of hazard ratios is tested using z-test with 2-sided alpha=0.05, power=0.80. Assumes all Marker=Low pts have median PFS=15 months, and the Marker= High Control pts have median PFS=18 months (prognostic HR = 0.83). With 150 evaluable pts and 20% Marker=High, the event count is too low for analysis.

Predictive Effect Estimation

These models will include all evaluable patients according to the randomized treatment assignment. For patients randomized to the control arm, information collected after crossover to trametinib will be excluded.

The predictive hypotheses will be assessed using multivariable logistic or proportional hazards modeling, specified with main effects for the randomized treatment assignment, baseline marker status, and the interaction terms. Covariate adjustment will include the main effects of geographic region, performance status and number of prior regimens (the stratification factors in Section 11.2). If the interaction term from this model has p-value < 0.05, the marker will be considered predictive and interesting for future validation studies.

Qualitative Interaction

The predictive modeling above considers so-called quantitative differences in the treatment effects within patient subsets defined by marker status. Gail (1985) defines a qualitative interaction as when "the direction of the true treatment differences varies among subsets of patients."[38] This working definition is satisfied by the combination of two scenarios:

$$(\delta_{H\cdot} < 1.0 \text{ and } \delta_{L\cdot} > 1.0) \text{ or } (\delta_{H\cdot} > 1.0 \text{ and } \delta_{L\cdot} < 1.0)$$

These possibilities will be addressed using a forest plot of the point estimates and simultaneous confidence intervals for ($\delta_{H\cdot}, \delta_{L\cdot}$) obtained from the multivariable modeling described above. Juxtaposition of these confidence intervals will show whether the data support the presence of a qualitative interaction. If the number of events and distribution of

the markers permit modeling within marker-based subsets, independent-sample modeling will be used instead.

Shared Analysis Details

All estimates will be derived using patient information collected while receiving the randomized treatment. Information from control patients collected after crossing over to trametinib will be excluded.

Our preference is to model the pERK H-Score as continuous variable, centered and scaled to reflect a clinically meaningful change. For explanatory purposes, the expected PFS distributions for specific pERK values may be estimated from the proportional hazards modeling. For confirmation, these expected distributions will be compared to Kaplan-Meier estimates based on the dichotomized H-Score.

Analysis of the dichotomous markers will be supported by Kaplan Meier plots, and forest plots of the odds-ratio and hazard ratio estimates. Duration of response will be depicted using swimmer plots, with median duration estimated using Kaplan Meier methods.

The multivariable models will include covariate adjustment for geographic region, performance status and number of prior regimens, presented using effect coding. If the data cannot reasonably support this many covariates, geographic region and performance status may be dropped. This approach assumes the number of prior therapy lines is the strongest confounder for the marker/outcome associations.

Cases with undue influence will be identified using deviance or martingale residual diagnostics, changes in the estimated effects (e.g. diff betas), and likelihood displacement measures. If a problem exists, subsequent exploratory analyses will be conducted with these cases removed (these cases will be explicitly stated as being dropped from the analysis).

The adjusted hazard- and odds- ratio estimates from the multivariable models will be supported by nominal p-values and 2-sided, 95% confidence intervals. Confidence intervals will be interpreted as the plausible range of values for the true (unobserved) ratio that is supported by the data. For the prediction analyses, point estimates and confidence intervals will be provided for the treatment effect within the biomarker levels.

Multiplicity adjustments will be conducted within the prognostic and predictive testing families, and if at least one nominal p-value is <0.05. Familywise type I error rates will be controlled to 0.05 using methods described by Hommel.[39] Both nominal and adjusted p-values will be presented.

Additional information on Human Tumor Tissues: Refer to Appendix IV.

11.9 Anticipated Gender and Minority Inclusion

This study restricts entry to women by nature of the site of disease. The table below lists the anticipated percentages of patients by racial and ethnic subgroups (based on previous studies in this patient population) for patients in the US.

NOTE: approximately half of the patients are expected to come from accrual in the United Kingdom, in which race and ethnicity information is not collected.

**Ethnic Category**	**Sex/Gender**				
	**Females**		**Males**		**Total**
Hispanic or Latino	18	+	0	=	18
Not Hispanic or Latino	107	+	0	=	107
**Ethnic Category: Total of all subjects**	125	+	0	=	125
**Racial Category**					
American Indian or Alaskan Native	1	+	0	=	1
Asian	6	+	0	=	6
Black or African American	6	+	0	=	6
Native Hawaiian or other Pacific Islander	1	+	0	=	1
White	111	+	0	=	111
**Racial Category: Total of all subjects**	125	+	0	=	125

12.0 BIBLIOGRAPHY

1. Baak JP, Langley FA, Talerman A, Delemarre JF. Interpathologist and intrapathologist disagreement in ovarian tumor grading and typing. Anal Quant Cytol Histol. 1986 Dec;8(4): 354-7, NLM ID: 8506819, PMID: 3814303.
2. Stalsberg H, Abeler V, Blom GP, Bostad L, Skarland E, Westgaard G. Observer variation in histologic classification of malignant and borderline ovarian tumors. Hum Pathol. 1988; Sept;19(9):1030-5, NLM ID: 9421547, PMID: 3417288.
3. Bichel P, Jakobsen A. A new histologic grading index in ovarian carcinoma. Int J Gynecol Pathol.1989;8(2):147-55, NLM ID: 8214845, PMID: 2714932.
4. Shimizu Y, Kamoi S, Amada S, Hasumi K, Akiyama F, Silverberg SG. Toward the development of a universal grading system for ovarian epithelial carcinoma. I. Prognostic significance of histopathologic features--problems involved in the architectural grading system. Gynecol Oncol 1998;Jul;70(1):2-12, NLM ID: 0365304, PMID: 9698465.
5. Silverberg SG. Histopathologic grading of ovarian carcinoma: a review and proposal. Int J Gynecol Pathol 2000 Jan;19(1):7-15, NLM ID: 8214845, PMID: 10638449.
6. Malpica A, Deavers MT, Lu K, Bodurka DC, Atkinson EN, Gershenson DM, et al. Grading ovarian serous carcinoma using a two-tier system. Am J Surg Pathol. 2004 Apr;28(4):496-504, NML ID 7707904, PMID 15087669.
7. Malpica A, Deavers MT, Tornos C, Kurman RJ, Soslow R, Seidman JD, et al. Interobserver and intraobserver variability of a two-tier system for grading ovarian serous carcinoma. Am J Surg Pathol. 2007 Aug;31(8):1168-74, NML ID: 7707904, PMID: 17667538.
8. Bodurka DC, Deavers MT, Tian C, Sun CC, Malpica A, Coleman RL, et al. Reclassification of serous ovarian carcinoma by a 2-tier system: A Gynecologic Oncology Group Study. Cancer. 2012 June 15;118(12):3087-94, NML ID: 0374236, PMID: 22072418.
9. Singer G, Shih Ie M, Truskinovsky A, Umudum H, Kurman RJ. Mutational analysis of K-ras segregates ovarian serous carcinomas into two types: invasive MPSC (low-grade tumor) and conventional serous carcinoma (high-grade tumor). Int J Gynecol Pathol. 2003 Jan; 22(1): 37-41, NML ID: 8214845, PMID:12496696.
10. Singer G, Oldt R, 3rd, Cohen Y, Wang BG, Sidransky D, Kurman RJ, et al. Mutations in BRAF and KRAS characterize the development of low-grade ovarian serous carcinoma. J Natl Cancer Inst. 2003 Mar 19;95(6): 484-6, NML ID: 7503089, PMID: 12644542.
11. Singer G, Kurman RJ, Chang HW, Cho SK, Shih leM. Diverse tumorigenic pathways in ovarian serous carcinoma. Am J Pathol. 2002 Apr;160(4):1223-8, NML ID: 0370502, PMID: 11943707.
12. Gilks CB. Subclassification of ovarian surface epithelial tumors based on correlation of histologic and molecular pathologic data. Int J Gynecol Pathol. 2004 Jul;23(3):200-5, NML ID: 8214845, PMID: 15213595.

13. Hsu CY, Bristow R, Cha MS, Wang BG, Ho CL, Kurman RJ, et al. Characterization of active mitogen-activated protein kinase in ovarian serous carcinomas. Clin Cancer Res. 2004 Oct1;10(19):6432-6, NML ID: 9502500, PMID: 15475429.

14. Singer G, Stohr R, Cope L, Dehari R, Hartmann A, Cao DF, et al. Patterns of p53 mutations separate ovarian serous borderline tumors and low- and high-grade carcinomas and provide support for a new model of ovarian carcinogenesis: a mutational analysis with immunohistochemical correlation. Am J Surg Pathol. 2005 Feb;29(2):218-24,NML ID: 7707904, PMID: 15644779.

15. Bonome T, Lee JY, Park DC, Radonovich M, Pise-Masison C, Brady J, et al. Expression profiling of serous low malignant potential, low-grade, and high-grade tumors of the ovary. Cancer Res. 2005 Nov 1;65(22):10602-12, NML ID: 2984705R, PMID: 16288054.

16. Jazaeri AA, Lu K, Schmandt R, Harris CP, Rao PH, Sotiriou C, et al. Molecular determinants of tumor differentiation in papillary serous ovarian carcinoma. Mol Carcinog. 2003 Feb;36(2):53-9, NML ID: 8811105, PMID: 12557260.

17. Meinhold-Heerlein I, Bauerschlag D, Hilpert F, Dimitrov P, Sapinoso LM, Orlowska-Volk M, et al. Molecular and prognostic distinction between serous ovarian carcinomas of varying grade and malignant potential. Oncogene. 2005 Feb3; 24(6):1053-65, NML ID: 8711562, PMID: 15558012.

18. O'Neill CJ, Deavers MT, Malpica A, Foster H, McCluggage WG. An immunohistochemical comparison between low-grade and high-grade ovarian serous carcinomas: significantly higher expression of p53, MIB1, BCL2, HER-2/neu, and C-KIT in high-grade neoplasms. Am J Surg Pathol. 2005 Aug;29(8):1034-41, NML ID: 7707904, PMID: 16006797.

19. Wong KK, Lu KH, Malpica A, Bodurka DC, Shvartsman HS, Schmandt RE, et al. Significantly greater expression of ER, PR, and ECAD in advanced-stage low-grade ovarian serous carcinoma as revealed by immunohistochemical analysis. Int J Gynecol Pathol. 2007 Oct;26(4):404-9, NML ID: 8214845, PMID: 17885490.

20. Gershenson DM, Sun CC, Lu KH, Coleman RL, Sood AK, Malpica A, et al. Clinical behavior of stage II-IV low-grade serous carcinoma of the ovary. Obstet Gynecol. 2006 Aug;108(2): 361-8, NML ID: 0401101, PMID: 16880307.

21. Shvartsman HS, Sun CC, Bodurka DC, Mahajan V, Crispens M, Lu KH, et al. Comparison of the clinical behavior of newly diagnosed stages II-IV low-grade serous carcinoma of the ovary with that of serous ovarian tumors of low malignant potential that recur as low-grade serous carcinoma. Gynecol Oncol. 2007 June;105(3):625-9, NML ID: 0365304, PMID: 17320156.

22. Schmeler KM, Sun CC, Bodurka DC, Deavers MT, Malpica A, Coleman RL, et al. Neoadjuvant chemotherapy for low-grade serous carcinoma of the ovary or peritoneum. Gynecol Oncol. 2008 Mar;108(3):510-14, NML ID: 0365304, PMID 18155273.

23. Gershenson DM, Sun CC, Bodurka DC, Coleman RL, Lu KH, Sood AK, et al. Recurrent low-grade serous ovarian carcinoma is relatively chemoresistant. Gynecol Oncol. 2009 Jul;114(1):48-52, NLM ID: 0365304, PMID 19361839.

24. Crispens MA, Bodurka D, Deavers M, Lu K, Silva EG, Gershenson DM. Response and survival in patients with progressive or recurrent serous ovarian tumors of low malignant potential. Obstet Gynecol. 2002 Jan;99:3-10, 2002 NLM ID: 0401101, PMID: 11777502.

25. Silva EG, Gershenson DM, Malpica A, Deavers M. The recurrence and the overall survival rates of ovarian serous borderline neoplasms with noninvasive implants is time dependent. Am J Surg Pathol. 2006 Nov;30(11):1367-71, NLM ID: 7707904, PMID: 17063075.

26. Gershenson DM, Sun CC, Iyer R, Malpica AL, Kavanagh JJ, Bodurka DC, et al. Hormonal therapy for recurrent low-grade serous carcinoma of the ovary or peritoneum. Gynecol Oncol. 2012 Jun;125(3):661-666, NLM ID: 0365304, PMID: 22406638.

27. Farley J, Brady WE, Vathipadiekal V, Lankes HA, Coleman R, Morgan MA, Mannel R, Yamada SD, Mutch D, Rodgers WH, Birrer M, Gershenson DM. Selumetinib in women with recurrent low-grade serous carcinoma of the ovary or peritoneum: an open-label, single-arm, phase 2 study. Lancet Oncol 2013 Feb;14:134-40, PMID: 23261356.

28. Wong KK, Tsang YTM, Deavers MT, Mok SC, Zu Z, Sun C, et al. BRAF mutation is rare in advanced stage low-grade ovarian serous carcinomas. Am J Pathol. 2010 Oct;177(4):1611-17, NML ID: 0370502, PMID: 20802181.

29. Nakayama N, Nakayama K, Yeasmin S, Ishibashi M, Katagiri A, lida K, et al. KRAS or BRAF mutation status is a useful predictor of sensitivity to MEK inhibition in ovarian cancer. Brit J Cancer. 2008 Dec 16;99(12):2020-8, NLM ID: 0370635, PMID: 19018267.

30. Stuart GC, Kitchener H, Bacon M, duBois A, Friedlander M, Ledermann J, et al. 2010 Gynecologic Cancer InterGroup (GCIG) consensus statement on clinical trials in ovarian cancer: Report from the Fourth Ovarian Cancer Consensus Conference. Int J Gynecol Cancer. 2011 May; 21(4):750-755, NML ID: 9111626, PMID: 21543936.

31. Yeh et al. KRAS/BRAF mutation status and ERK1/2 activation as biomarkers for MEK1/2 inhibitor therapy in colorectal cancer. Mol Cancer Ther 2009;8:834-43

32. Jennison C and Turnbull BW (2000). Group Sequential Methods with Applications to Clinical Trials. Chapman and Hall/CRC, London.

33. Robins JM, Finkelstein DM (2000): Correcting for noncompliance and dependent censoring in an AIDS clinical trial with inverse probability of censoring weighted (IPCW) log rank tests. Biometrics 56:779-788.

34. Altman DG (2012) Reporting Recommendations for Tumor Marker Prognostic Studies (REMARK): Explanation and Elaboration. *PLoS Medicine* 9(5):e1001216.

35. McShane L. (2012) Publication of Tumor Marker Research Results: The Necessity for Complete and Transparent Reporting. *Journal of Clinical Oncology* 30(34):4223-4232.

36. Cox DR. Regression models and life-tables. Journal of the Royal Statistical Society 1972; 34: 187-220.

37. Peterson (1993) Sample size requirements and length of study for testing interaction is a 2xk factorial design when time-to-failure is the outcome. *Controlled Clinical Trials* 14:511-522

38. Gail (1985) Testing for Qualitative Interactions between Treatment Effects and Patient Subsets *Biometrics* 41(2), 361-37.
39. Hommel G. (1989) A comparison of two modified Bonferroni procedures. *Biometrika* 76(3) 624-625.
40. Schmeler KM, Gershenson DM. Low-grade serous ovarian cancer: a unique disease. Curr Oncol Rep 2008; 10: 519-523.
41. Romero I, Sun CC, Wong KK et al. Low-grade serous carcinoma: new concepts and emerging therapies. Gynecol Oncol 2013; 130: 660-666.
42. Gershenson DM, Bodurka DC, Lu KH et al. Impact of Age and Primary Disease Site on Outcome in Women With Low-Grade Serous Carcinoma of the Ovary or Peritoneum: Results of a Large Single-Institution Registry of a Rare Tumor. J Clin Oncol 2015.
43. Okoye E, Euscher ED, Malpica A. Ovarian Low-grade Serous Carcinoma: A Clinicopathologic Study of 33 Cases With Primary Surgery Performed at a Single Institution. Am J Surg Pathol 2016; 40: 627-635.
44. Wong KK, Gershenson D. The continuum of serous tumors of low malignant potential and low-grade serous carcinomas of the ovary. Dis Markers 2007; 23: 377-387.
45. Nik NN, Vang R, Shih Ie M, Kurman RJ. Origin and pathogenesis of pelvic (ovarian, tubal, and primary peritoneal) serous carcinoma. Annu Rev Pathol 2014; 9: 27-45.
46. Zhao Y, Adjei AA. The clinical development of MEK inhibitors. Nat Rev Clin Oncol 2014; 11: 385-400.
47. Pohl G, Ho CL, Kurman RJ et al. Inactivation of the mitogen-activated protein kinase pathway as a potential target-based therapy in ovarian serous tumors with KRAS or BRAF mutations. Cancer Res 2005; 65: 1994-2000.
48. Vang R, Shih Ie M, Kurman RJ. Ovarian low-grade and high-grade serous carcinoma: pathogenesis, clinicopathologic and molecular biologic features, and diagnostic problems. Adv Anat Pathol 2009; 16: 267-282.
49. Gershenson DM, Sun CC, Wong KK. Impact of mutational status on survival in low-grade serous carcinoma of the ovary or peritoneum. Br J Cancer 2015; 113: 1254-1258.
50. Tsang YT, Deavers MT, Sun CC et al. KRAS (but not BRAF) mutations in ovarian serous borderline tumour are associated with recurrent low-grade serous carcinoma. J Pathol 2013; 231: 449-456.
51. Van Allen EM, Wagle N, Stojanov P et al. Whole-exome sequencing and clinical interpretation of formalin-fixed, paraffin-embedded tumor samples to guide precision cancer medicine. Nat Med 2014; 20: 682-688.

52. Rennert H, Eng K, Zhang T et al. Development and validation of a whole-exome sequencing test for simultaneous detection of point mutations, indels and copy-number alterations for precision cancer care. NPJ Genom Med 2016; 1.

53. Grisham RN, Sylvester BE, Won H et al. Extreme Outlier Analysis Identifies Occult Mitogen-Activated Protein Kinase Pathway Mutations in Patients With Low-Grade Serous Ovarian Cancer. J Clin Oncol 2015; 33: 4099-4105.

54. Takekuma M, Wong KK, Coleman RL. A long-term surviving patient with recurrent low-grade serous ovarian carcinoma treated with the MEK1/2 inhibitor, selumetinib. Gynecol Oncol Res Pract 2016; 3: 5.

APPENDIX I - CRADA/CTA LANGUAGE

NCI/ DCTD Standard Language to Be Incorporated into All Protocols Involving Agent(s) Covered by a Clinical Trials Agreement (CTA) a Cooperative Research and Development Agreement (CRADA) or a Clinical Supply Agreement, hereinafter referred to as Collaborative Agreement:

The agent(s) supplied by CTEP, DCTD, NCI used in this protocol is/are provided to the NCI under a Collaborative Agreement (CRADA, CTA, CSA) between the Pharmaceutical Company(ies) (hereinafter referred to as "Collaborator(s)") and the NCI Division of Cancer Treatment and Diagnosis. Therefore, the following obligations/guidelines, in addition to the provisions in the "Intellectual Property Option to Collaborator" (http://ctep.cancer.gov/industryCollaborations2/intellectual_property.htm) contained within the terms of award, apply to the use of the Agent(s) in this study:

1. Agent(s) may not be used for any purpose outside the scope of this protocol, nor can Agent(s) be transferred or licensed to any party not participating in the clinical study. Collaborator(s) data for Agent(s) are confidential and proprietary to Collaborator(s) and shall be maintained as such by the investigators. The protocol documents for studies utilizing investigational Agents contain confidential information and should not be shared or distributed without the permission of the NCI. If a copy of this protocol is requested by a patient or patient's family member participating on the study, the individual should sign a confidentiality agreement. A suitable model agreement can be downloaded from: http://ctep.cancer.gov.

2. For a clinical protocol where there is an investigational Agent used in combination with (an)other investigational Agent(s), each the subject of different collaborative agreements , the access to and use of data by each Collaborator shall be as follows (data pertaining to such combination use shall hereinafter be referred to as "Multi-Party Data":

    a. NCI will provide all Collaborators with prior written notice regarding the existence and nature of any agreements governing their collaboration with NIH, the design of the proposed combination protocol, and the existence of any obligations that would tend to restrict NCI's participation in the proposed combination protocol.

    b. Each Collaborator shall agree to permit use of the Multi-Party Data from the clinical trial by any other Collaborator solely to the extent necessary to allow said other Collaborator to develop, obtain regulatory approval or commercialize its own investigational Agent.

    c. Any Collaborator having the right to use the Multi-Party Data from these trials must agree in writing prior to the commencement of the trials that it will use the Multi-Party Data solely for development, regulatory approval, and commercialization of its own investigational Agent.

3. Clinical Trial Data and Results and Raw Data developed under a Collaborative Agreement will be made available to Collaborator(s), the NCI, and the FDA, as appropriate and unless additional disclosure is required by law or court order as described in the IP Option to Collaborator (http://ctep.cancer.gov/industryCollaborations2/intellectual_property.htm). Additionally, all Clinical Data and Results and Raw Data will be collected, used and disclosed consistent with all applicable federal statutes and regulations for the protection of human subjects, including, if applicable, the Standards for Privacy of Individually Identifiable Health Information set forth in 45 C.F.R. Part 164.

4. When a Collaborator wishes to initiate a data request, the request should first be sent to the NCI, who will then notify the appropriate investigators (Group Chair for Cooperative Group studies, or PI for other studies) of Collaborator's wish to contact them.

5. Any data provided to Collaborator(s) for Phase 3 studies must be in accordance with the guidelines and policies of the responsible Data Monitoring Committee (DMC), if there is a DMC for this clinical trial.

6. Any manuscripts reporting the results of this clinical trial must be provided to CTEP by the Group office for Cooperative Group studies or by the principal investigator for non-Cooperative Group studies for immediate delivery to Collaborator(s) for advisory review and comment prior to submission for publication. Collaborator(s) will have 30 days from the date of receipt for review. Collaborator shall have the right to request that publication be delayed for up to an additional 30 days in order to ensure that Collaborator's confidential and proprietary data, in addition to Collaborator(s)'s intellectual property rights, are protected. Copies of abstracts must be provided to CTEP for forwarding to Collaborator(s) for courtesy review as soon as possible and preferably at least three (3) days prior to submission, but in any case, prior to presentation at the meeting or publication in the proceedings. Press releases and other media presentations must also be forwarded to CTEP prior to release. Copies of any manuscript, abstract and/or press release/ media presentation should be sent to:

Email: ncicteppubs@mail.nih.gov

The Regulatory Affairs Branch will then distribute them to Collaborator(s). No publication, manuscript or other form of public disclosure shall contain any of Collaborators confidential proprietary information.

APPENDIX II - NRG Oncology General Therapy Guidelines (06/08/15)

- For 21 or 28 day cycles, a patient will be permitted to have a new cycle of therapy delayed up to 7 days (without this being considered to be a protocol violation) for major life events (e.g., serious illness in a family member, major holiday, vacation which is unable to be re-scheduled). Documentation to justify this decision should be provided.
- It will be acceptable for individual doses to be delivered within a "24-hour window before and after the protocol-defined date" for "Day 1" treatment of 21 or 28 day cycles. If the treatment due date is a Friday, and the patient cannot be treated on that Friday, then the window for treatment would include the Thursday (1 day earlier than due) through the Monday (day 3 past due).
- For weekly regimens, it will be acceptable for individual doses to be delivered within a "24-hour window," for example; "Day 8 therapy" can be delivered on Day 7, Day 8, or Day 9 and "Day 15 therapy" can be given on Day 14, Day 15, or Day 16.
- Doses can be "rounded" according to institutional standards without being considered a protocol violation (most institutions use a rule of approximately +/- 5% of the calculated dose).
- Doses are required to be recalculated if the patient has a weight change of greater than or equal to 10%. Patients are permitted to have chemotherapy doses recalculated for less than 10% weight changes.

APPENDIX III - New York Heart Association Classifications

Clinical Evaluation of Functional Capacity of Patients
with Heart Disease in Relation to Ordinary Physical Activity

Class	Cardiac Symptoms	Limitations	Need for Additional Rest*	Physical Ability to work**
I	None	None	None	Full time
II	Only moderate	Slight	Usually only slight or occasional	Usually full time
III	Defined, with less than ordinary activity	Marked	Usually moderate	Usually part time
IV	May be present even at rest, and any activity increases discomfort	Extreme	Marked	Unable to work

* To control or relieve symptoms, as determined by the patient, rather than as advised by the physician.

** At accustomed occupation or usual tasks.

APPENDIX IV – Translational Research Specimen Procedures (07/28/14)(06/08/15) (11/27/17)

I. Obtaining a Bank ID for Translational Research Specimens

Only one Bank ID (# # # # - # # - G # # #) is assigned per patient. All translational research specimens and accompanying paperwork must be labeled with this coded patient number.

A Bank ID is automatically assigned once the Specimen Consent is completed and indicates that a patient has agreed to participate in the translational science component. If a patient has previously been assigned a Bank ID, please ensure the Bank ID appearing in Rave is the same as the previously assigned Bank ID.

Please contact User Support if you need assistance or have assigned more than one Bank ID to a patient (Email: support@nrgoncology.org; Phone: 716-845-7767).

II. Requesting Translational Research Specimen Kits

Single chamber specimen kits will be provided for the collection and shipment of the frozen plasma specimens. Separate kits are provided for baseline and cycle 3 specimens.

Kits can be ordered online via the Kit Management system (http://ricapps.nationwidechildrens.org/KitManagement/). Each site may order two kits per protocol per day (daily max = 6 kits).

Please contact the GOG Tissue Bank if you need assistance (Email: BPCBank@nationwidechildrens.org; Phone: 866-464-2262).

Be sure to plan ahead and allow time for kits to be shipped by ground transportation. Kits should arrive within 3-5 business days.

Note: Unused supplies and kits should be returned to the GOG Tissue Bank. A pre-paid shipping label for the return of unused supplies and kits may be obtained via the Kit Management system. Select "Empty Kit" for package contents when returning unused kits.

III. Labeling Translational Research Specimens

A waterproof permanent marker or printed label should be used to label each translational research specimen with:

Bank ID (# # # # - # # - G # # #)
protocol number (GOG 0281)
specimen code (see protocol section 7.3)
collection date (mm/dd/yyyy)
surgical pathology accession number (tissue specimens only)
block number (tissue specimens only)

Note: If labeling slides, only label on the top, front portion of the slide. Do not place a label on the back of the slide or over the tissue. The label must fit on the slide and should not be wrapped around the slide or hang over the edge.

IV. Submitting Frozen Tissue

Note: Frozen biopsy and PK plasma specimens are no longer required as of October 2017, and should not be collected.

VI. Submitting Formalin-Fixed, Paraffin-Embedded Tissue

Formalin-fixed, paraffin embedded (FFPE) tissue should be the most representative of the tumor type (primary, metastatic, recurrent). Primary and metastatic tumor should be collected prior to all treatment. Recurrent tumor should be collected prior to the study treatment. Only one block may be submitted per tissue type.

Every attempt should be made to provide a FFPE block; however, if a block cannot be provided on a permanent basis, then 20 unstained slides (10 charged, 5µm, and 10 uncharged, 10µm) should be submitted. All tissue sections should be cut sequentially from the same block.

The type of specimen (block or slides) should be specified on Form TR. If submitting slides, the slide type, thickness, and count should also be specified.

All FFPE tissue should be submitted with the corresponding pathology report.

VII. Submitting Plasma

Please note the protocol-specific plasma processing instructions described below.

1. Label 2mL cryovials as described above.
2. Draw 7-10mL of blood into a **K2 EDTA tube**.
3. Immediately after collection, gently invert the blood collection tube 5-10 times to mix the blood and EDTA.
4. **Immediately after mixing the blood and EDTA, the blood collection tube must be placed on wet ice until centrifuged. The blood sample must be centrifuged within 15 minutes (30 minutes maximum).***
5. Centrifuge the blood at **approximately 1600g** for 15 minutes at **4ºC*** to separate the plasma (top, straw-colored layer) from the red blood cells (bottom, red layer).
6. Quickly, evenly dispense (aliquot) the plasma into the pre-labeled cryovials and cap the tubes securely. Place a minimum of 0.25mL into each cryovial.
7. Immediately **freeze the plasma in an upright position** in a -70°C to -80°C freezer or by direct exposure with dry ice until ready to ship. If a -70°C to -80°C freezer is not available for storage, store and ship on dry ice within 24 hours of collection.

*** Deviations to the plasma processing protocol <u>must</u> be noted in Rave.**

VIII. Submitting Form TR

A specimen transmittal form (i.e., Form TR) for each specimen will be available in the **Translational Research Folder in Rave**, once the Specimen Consent (located in the Baseline Folder) has been completed.

An electronically (i.e., Rave) completed copy of Form TR must accompany each specimen shipped to the GOG Tissue Bank. Handwritten forms will not be accepted.

Note: A copy does not need to be sent to the GOG Tissue Bank if specimens are not collected.

Form TR **<u>must</u>** be printed from the Translational Research Form screen in Rave using the **"PDF File" link at the top of the form**. Clicking this link will generate a single page PDF. Do not use the "Printable Version" or "View PDF" links at the bottom of the form or any other method to print the form, as these formats will not be accepted.

Note: Specimens will not be marked as received in Rave without receipt of a corresponding electronically completed Form TR. **Incomplete forms or those containing incorrect information will not be processed.**

Retain a printout of the completed form for your records.

Please contact Support if you need assistance (Email: support@nrgoncology.org; Phone: 716-845-7767).

IX. Shipping Translational Research Specimens

A completed copy of Form TR must be included for each translational research specimen.

A. FFPE Tissue

FFPE tissue and a copy of the corresponding pathology report should be shipped using your own container at your own expense to:

GOG Tissue Bank / Protocol GOG-0281
Nationwide Children's Hospital
700 Children's Dr, WA1340
Columbus, OH 43205
Phone: (614) 722-2865
FAX: (614) 722-2897
Email: BPCBank@nationwidechildrens.org

Do not ship FFPE tissue for Saturday delivery.

B. Frozen Plasma

The frozen plasma should be shipped using the specimen kit provided to the GOG Tissue Bank (address above).

Frozen specimens should be shipped **Monday through Thursday for Tuesday through Friday delivery.** Do not ship frozen specimens the day before a holiday.

Frozen specimens should be stored in an ultra-cold freezing/storage space (i.e., ultra cold ≤-70oC freezer, liquid nitrogen, or direct exposure with dry ice) until the specimens can be shipped.

Shipping Frozen Translational Research Specimens in a Single Chamber Kit

1. Pre-fill the kit chamber about 1/3 full with dry ice.
2. Place the frozen specimens in a zip-lock bag. If shipping specimens from more than one time point, place specimens for each time point in separate bags.
3. Place the zip-lock bag(s) in the biohazard envelope containing absorbent material. If batch shipping more than one patient in the same shipment, do not put more than 20 vials in a biohazard envelope. Put the secondary envelope into a Tyvek envelope. Expel as much air as possible before sealing both envelopes.
4. Place the Tyvek envelope containing the frozen specimens into the kit and fill the chamber to the top with dry ice.
5. Insert a copy of Form TR for each specimen.
6. Place the cover on top of the kit. Tape the outer box of the kit closed with filament or other durable sealing tape. Please do not tape the inner chamber.
7. Print a pre-paid FedEx air bill using the Kit Management system (http://ricapps.nationwidechildrens.org/KitManagement/). Attach the air bill.
8. Attach the dry ice label (UN1845) and the Exempt Human Specimen sticker.
9. Arrange for FedEx pick-up through your usual Institutional procedure or by calling 800-238-5355.

X. Distributing Translational Research Specimens

The GOG Statistical and Data Center and Tissue Bank (or alternate laboratory) will coordinate the distribution of translational research specimens to approved investigators.

Investigators will not be given access to any personal identifiers.

Investigators will be responsible for the direct supervision and oversight of translational research performed and for keeping accurate records.

Investigators will ensure the results are linked to the appropriate specimen-specific identifiers and are responsible for transferring relevant laboratory data to the Statistical and Data Center.

At the discretion of the Chair of the Committee on Experimental Medicine and the Director of the GOG Tissue Bank, investigators may be required to ship any specimens (or by-products) remaining after the completion of the translational research to the GOG Tissue Bank.

XI. Banking Translational Research Specimens for Future Research

Specimens will remain banked in the GOG Tissue Bank and made available for approved projects if the patient has provided permission for the use of her specimens for future health research. The patient's choices will be recorded on the signed informed consent document and electronically via the online Specimen Consent Application. At the time of specimen selection for project distribution, the most recent consent information will be used.

GOG institutions can amend a patient's choices regarding the future use of her specimens at any time if the patient changes her mind.

If the patient revokes permission to use her specimens, the GOG Tissue Bank will destroy or return any remaining specimens. The patient's specimens will not be used for any further research; however, any specimens distributed for research prior to revoking consent cannot be returned or destroyed. In addition, the patient cannot be removed from any research that has been done with her specimens prior to revoking consent.

Note: If return of specimens is requested, shipping will be at the institution's expense.

APPENDIX V - Patient Medication Calendar (12/22/14)

PILL DIARY

INSTRUCTIONS FOR THE PATIENT:

This is a monthly calendar on which you are to record the total number of pills (study medication) you take each day and the time of day you take your pills (study medication).

Bring the bottle(s) with any unused pills and your calendar with you each time you have an appointment.

If you have any questions, please contact: ____________________________________
Telephone: ________________________
Your next appointment is: ____

#of pills taken_____<br><br>Time:<br>a.m. □<br><br>Time<br>p. m. □	#of pills taken_____<br><br>Time:<br>a.m. □<br><br>Time<br>p. m. □	#of pills taken_____<br><br>Time:<br>a.m. □<br><br>Time<br>p. m. □	#of pills taken_____<br><br>Time:<br>a.m. □<br><br>Time<br>p. m. □	#of pills taken_____<br><br>Time:<br>a.m. □<br><br>Time<br>p. m. □	#of pills taken_____<br><br>Time:<br>a.m. □<br><br>Time<br>p. m. □	#of pills taken_____<br><br>Time:<br>a.m. □<br><br>Time<br>p. m. □
#of pills taken_____<br><br>Time:<br>a.m. □<br><br>Time<br>p. m. □	#of pills taken_____<br><br>Time:<br>a.m. □<br><br>Time<br>p. m. □	#of pills taken_____<br><br>Time:<br>a.m. □<br><br>Time<br>p. m. □	#of pills taken_____<br><br>Time:<br>a.m. □<br><br>Time<br>p. m. □	#of pills taken_____<br><br>Time:<br>a.m. □<br><br>Time<br>p. m. □	#of pills taken_____<br><br>Time:<br>a.m. □<br><br>Time<br>p. m. □	#of pills taken_____<br><br>Time:<br>a.m. □<br><br>Time<br>p. m. □
#of pills taken_____<br><br>Time:<br>a.m. □<br><br>Time<br>p. m. □	#of pills taken_____<br><br>Time:<br>a.m. □<br><br>Time<br>p. m. □	#of pills taken_____<br><br>Time:<br>a.m. □<br><br>Time<br>p. m. □	#of pills taken_____<br><br>Time:<br>a.m. □<br><br>Time<br>p. m. □	#of pills taken_____<br><br>Time:<br>a.m. □<br><br>Time<br>p. m. □	#of pills taken_____<br><br>Time:<br>a.m. □<br><br>Time<br>p. m. □	#of pills taken_____<br><br>Time:<br>a.m. □<br><br>Time<br>p. m. □
#of pills taken_____<br><br>Time:<br>a.m. □<br><br>Time<br>p. m. □	#of pills taken_____<br><br>Time:<br>a.m. □<br><br>Time<br>p. m. □	#of pills taken_____<br><br>Time:<br>a.m. □<br><br>Time<br>p. m. □	#of pills taken_____<br><br>Time:<br>a.m. □<br><br>Time<br>p. m. □	#of pills taken_____<br><br>Time:<br>a.m. □<br><br>Time<br>p. m. □	#of pills taken_____<br><br>Time:<br>a.m. □<br><br>Time<br>p. m. □	#of pills taken_____<br><br>Time:<br>a.m. □<br><br>Time<br>p. m. □

This section is to be completed by the physician, nurse or staff:

Reporting period (mm/dd/yy): Start _______ Stop _______Total # of pills taken: ____ # pills left in bottle: ____

Patient’s Signature: ____ Date: _________

APPENDIX VI – FROZEN BIOPSY PATHOLOGY VERIFICATION (12/22/14) (11/27/17)

Note: Frozen biopsy and PK plasma specimens are no longer required as of October 2017, and should not be collected.

A copy of the corresponding pathology report must be shipped with all tissue specimens sent to the GOG Tissue Bank.

If a pathology report is not available for the frozen biopsy, a copy of the radiology report or operative report from the biopsy procedure <u>must</u> be sent to the GOG Tissue Bank. A completed copy of this appendix (i.e., Frozen Biopsy Pathology Verification) <u>must</u> also be submitted to the GOG Tissue Bank.

Note: If this information is not provided with the frozen biopsy specimen, it will not be accepted by the GOG Tissue Bank.

Please have the Pathologist responsible for signing out this patient's case complete the following:

GOG Bank ID: __ __ __ __ - __ __ - G __ __ __

GOG Study ID: __ __ __ - 0 2 8 1 - __ __ __

Date of Procedure (mm/dd/yyyy): ____

Tissue Type (circle one): Recurrent Primary Recurrent Metastatic

Site Tissue Taken From: ____

Diagnosis: ____

Recurrent disease documented by: ______________________________________

I agree that this tissue may be released for research purposes only and that the release of this tissue will not have any impact on the patient's care.

__________________________________ ____________________
Pathologist's Signature Date

____
Pathologist's Printed Name

APPENDIX VII – Translational Research Laboratory Testing Procedures

A. OVERVIEW: Low-grade ovarian serous carcinomas (LGSC) account for a significant proportion of epithelial ovarian cancers in young women. The exact biological pathways that underlie this disease are incompletely understood. Chemotherapy has a very poor response rate (< 4%), and the disease is associated with high morbidity and mortality.[40,41] With a median overall survival exceeding 80 months, an updated analysis of 350 women with LGSC from the MD Anderson Low-Grade Serous Tumor Database indicated that over 85% develop recurrent disease, and almost all ultimately die of the disease.[42] This is particularly important, as LGSC is more common in young women. Also, the overall response rate of recurrent low-grade serous cancer to various chemotherapies is dismal (< 4%).[23] Therefore, patients with LGSC have a 10-year overall survival rate (~21%) comparable to that of high-grade serous carcinoma (HGSC).[43] Even though LGSC and HGSC develop from a different pathogenetic pathway,[44,45] they do share several cancer hallmarks—invasiveness, peritoneal implantation and angiogenesis.

Because the Ras/Raf/MEK/ERK or MAP kinase (MAPK) pathway is frequently activated in LGSC, targeting this pathway has been examined as a potential treatment.[27] In a recent phase II Gynecologic Oncology Group (GOG) trial for patients with recurrent LGSC, GOG-0239 (Co-PI and senior author, Dr. Gershenson), 52 patients were treated with the MEK inhibitor (MEKi) selumetinib. Of these, one had a complete response, seven had a partial response, and 34 had stable disease.[27] Because of the encouraging overall response rate of 15% in this therapy-resistant disease, we have initiated, through NRG Oncology, an international phase II/III trial (NCT02101788, GOG-0281) to assess the efficacy of another MEKi (trametinib), with improved pharmacology, for patients with recurrent or progressive LGSC.

SIGNIFICANCE AND PRELIMINARY DATA:
This study using MEKi in the randomized setting provides the opportunity to investigate potential predictive biomarkers.[46] Based on our preliminary data, we will interrogate MAPK pathway mutations as well as protein expression of pERK. The Ras/Raf/MEK/ERK pathway is frequently dysregulated in LGSC[41] with pERK protein expressed (**Figure 1**). Activation of the Ras/Raf/MEK/ERK pathway by oncogenic *KRAS* mutation is well established.[9,47-49] We recently also demonstrated that *KRAS*-mutated clones are selected during the progression of a subset of serous borderline tumors to LGSC.[50] Preliminary data from the completed GOG-0239 trial of the MEKi selumetinib indicated that the percentage of patients with a response as measured by reduction in CA-125 level was significantly higher in patients with pERK H-score >109 than in those with pERK H-score ≤109: 46% versus 5% (p=0.008) (**Table 1**). Higher levels of pERK in the pre-treatment specimens from patients enrolled in GOG-0239 may suggest the tumor is more addicted to the pERK pathway and thus more sensitive to MEKi. In the GOG-0281 trial, we expect to find a similar correlation between pERK levels and response measured by reduction in CA-125 level. In addition, since we will use WES to interrogate mutations in genes involved in the Ras/Raf/MEK/ERK pathway (KRAS, BRAF, NRAS, HRAS, MEK, ERRB2 and NF-1), we anticipate that, in contrast to the findings of GOG-0239 (only KRAS and BRAF mutations were

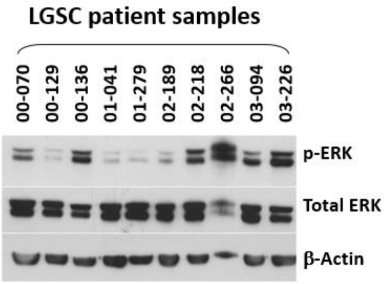

Figure 1. pERK was detected in the majority of low-grade OSC samples; however, only sample 00-136 had a *KRAS* mutation.

Table 1. CA-125 response by pERK positivity (score >109) (unpublished data from GOG 239 trial)

	pERK (H-Score)*			
	**N**	**≤109 n (%)**	**>109 n (%)**	**p value†**
**Total**	33	20	13	
**CA-125 Response‡**				
No	26	19 (95)	7 (54)	0.008
Yes	7	1 (5)	6 (46)	

*H-Score is derived from multiplying the immunostaining intensity (1, 2, or 3) by the percentage of cells stained (range: 0-300).
† Fisher's exact test.
‡ ≥50% decrease from baseline confirmed ≥28 days later.

checked), we will observe a more robust correlation between response to trametinib and mutation status. [Although NF-1 is not an integral part of the MAPK pathway, it has long been recognized that loss of function mutations result in MAPK activation and this event has been detected in our pilot sequencing studies (see below)].

SUMMARY:
If trametinib is found to be effective in this phase 2/3 clinical trial, the MAPK pathway signaling and gene mutations landscape investigated in this study will be critical in identifying the best candidates for this unique targeted therapy and guiding further studies and management strategies for LGSC. Alternatively, if trametinib is not found to be overall effective in the intention to treat population, we may be able to identify subgroups of patient who did derive benefit.

Integrated Biomarker Research Objective

(1) To test whether high expression of pERK, as quantified by immunohistochemistry (IHC), is associated with better prognosis (RR or PFS) among patients receiving the randomized treatment. <u>(IHC analysis will be performed at MD Anderson Cancer Center by Kwong Wong, PhD)</u>.

To quantify the expression level of pERK, we will perform IHC using formalin fixed paraffin embedded (FFPE) sections. The details of the pERK IHC are discussed in the laboratory procedure section below.

(2) To test whether genetic changes associated with MAPK pathway activation (KRAS, NRAS, HRAS, BRAF, MEK,ERBB2 or NF1) are associated with improved prognosis (RR or PFS) among patients receiving the randomized treatment. (<u>Whole Exome sequencing using FFPE samples will be performed at the University of Edinburgh by Dr. Charlie Gourley's group</u>)

Whole Exome Sequencing (WES) has been used to identify clinical actionable mutations from FFPE patient samples.[51,52] As the sequencing cost of WES is now comparable to targeted sequencing using cancer gene panels, we will perform WES of all the available FFPE samples to identify mutations that are associated with trametinib treatment response in LGSC. The details of the WES using FFPE samples are discussed in the laboratory procedure section. As well as providing a robust readout of sequence changes in the key genes of interest, the additional information provided by WES (over panel sequencing) will allow future exploratory analysis of mutational and copy number events across the genome.

Expected Findings:
We expect to be able to determine the sensitivity and specificity of each of the biomarkers individually or in combination to predict MEKi response. We also expect to have several exceptional responders with complete response as we have observed in the GOG-0239 trial. For those exceptional responders, the integrated WES and IHC analyses will reveal potential factors that contribute to the exceptional response. From previous study, Next Generation Sequencing analysis of an extreme responder with complete response in the GOG-0239 trial revealed a 15-nucleotide deletion in the negative regulatory helix of the *MAP2K1* gene encoding for MEK1. This mutated MEK protein induced extracellular signal-regulated kinase pathway activation and promoted anchorage-independent growth and tumor formation in mice.[53] Another extreme responder had a *KRAS G12V* mutation[54] and is still on selumetinib treatment with stable disease for more than nine years.

B. Laboratory Testing Procedures

Immunohistochemistry:
Using FFPE samples from patients receiving the randomized treatment , the expression level of pERK will be determined by immunocytochemistry and test for its prognostic significance and predictive value to trametinib. Two FFPE slides for each patient will be needed for reproducibility of pERK IHC.

Procedures will follow the IHC standard operating procedure (SOP) at the MD Anderson clinical histopathology lab. Paraffin-embedded specimens will be sliced into 5-µm sections, placed on glass slides. The BondMax Protocol described below using the BondMax autostainer will be used for staining of pERK in FFPE sections.

Bond Max Protocol

Step	Reagent	Time	°C	
1	Bond Dewax Solution	30 minutes	72	
2	Alcohol Rinse	(x3)		
3	Bond Wash	5 minutes		
4	Epitope Retrieval #1 (Citrate Buffer)	20 minutes	100	
5	Bond Wash	(x6)	35	
6	Bond Protein Block	15 minutes		
7	Bond Wash	(x3)		
8	Marker	15 minutes		
9	Bond Wash	(x3)		
10	Peroxide Block (3.0% Hydrogen Peroxide)	5 minutes		
11	Bond Wash	(x3)		
12	Post Primary (Polymer Enhancer)	8 minutes		
13	Polymer (Poly-HRP anti-mouse/anti-rabbit IgG	8 minutes		
14	Bond Wash	(x2)		
15	Deionized Water	(x1)		
16	DAB	10 minutes		
17	Deionized Water	(x3)		
18	DAB Enhancer	5 minutes		
19	Bond Wash	(x3)		
20	Hematoxylin	8 minutes		
21	Bond Wash	(x3)		
22	Dehydrate slides and cover slip			
	**MARKER**	**CLONE**	**DILUTION**	**SOURCE**
	Phospho-p44/42 MAPK (Erkl/2) (Thr202/Tyr204) pERK Positive Control Tissue: Tonsil	(D13.14.4E)	1:300	Cell Signaling Catalog#4370S

Sources of antibodies
Anti-Phospho-p44/42 MAPK (pERK) (Thr202/Tyr204) (cat#4370s), which is a Rabbit monoclonal antibody (D13.14.4E), will be purchased from Cell Signaling Technology. The specificity and performance of the anti-pERK antibody has been confirmed using FFPE NIH/3T3 cells, treated with U0126 (MEK inhibitor) and FFPE section of lung carcinoma treated with λ phosphatase (Figure 3)

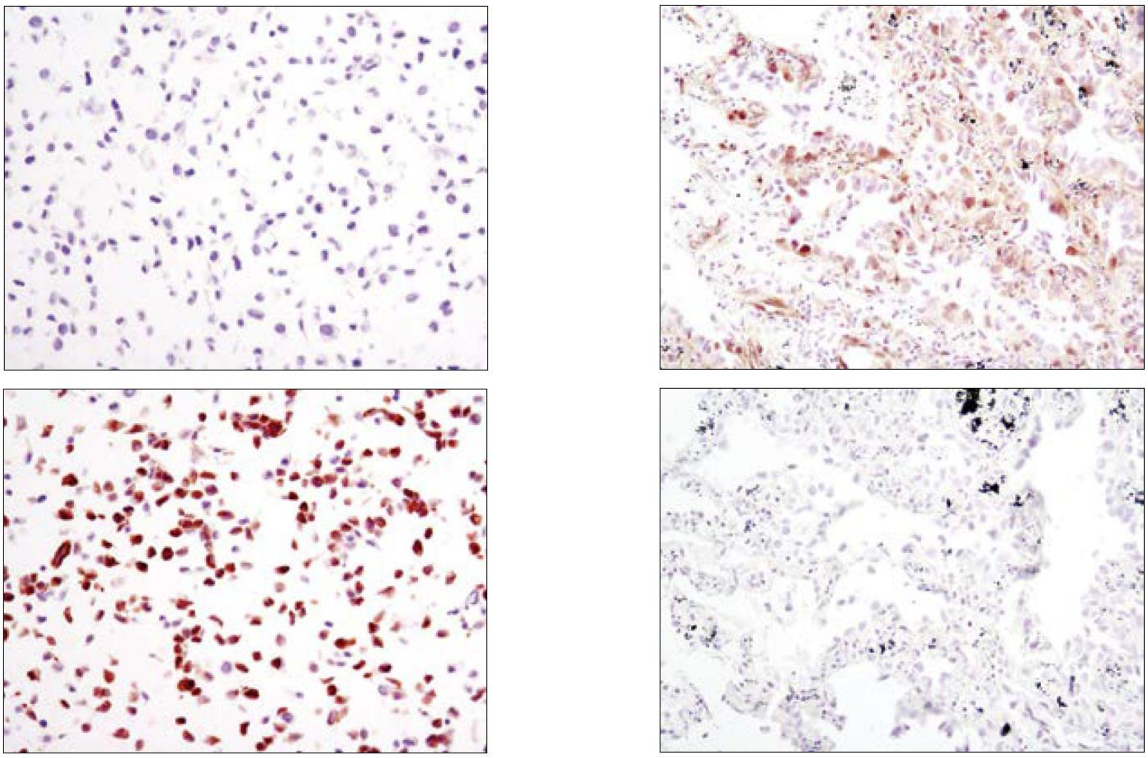

Immunohistochemical analysis using Phospho-p44/42 MAPK (Erk1/2) (Thr202/Tyr204) (D13.14.4E) XP® Rabbit mAb on SignalSlide™ Phospho-p44/42 MAPK (Thr202/Tyr204) IHC Controls #8103 (paraffin-embedded NIH/3T3 cells, treated with U0126 #9903 (upper) or TPA #4174 (lower).

Immunohistochemical analysis of paraffin-embedded human lung carcinoma, untreated (upper) or λ phosphatase-treated (lower), using Phospho-p44/42 MAPK (Erk1/2) (Thr202/Tyr204) (D13.14.4E) XP® Rabbit mAb.

Figure 3. Performance of Anti-Phospho-p44/42 MAPK (pERK) (Thr202/Tyr204) in IHC with FFPE samples.

<u>Analysis IHC images and scoring procedures</u>

All the immunostained tissue sections will be captured by Vectra 3.0 Automated Quantitative Pathology Imaging System. For each case, 15-20 fields at a magnification of x200 will be assessed. The staining intensity will be semi-quantified into four categories (0, negative; 1, low, 2, moderate; 3, strong) and the percentage of cells stained will be categorized into three groups (1, <25% positive cells; 2, 25-75% positive cells; 3, >75% positive cells). Quantification and scoring will be generated by InForm® software (PerkinElmer). 2000 to 10000 tumor cells will be quantified. The threshold Max for the selected nuclear component will be calculated automatically by the software and the positivity threshold value will be defined by the user based on region with negative stain and negative control slides. An H-score is assigned to each case using the following formula and H-score will be generated automatically by InForm® imaging software :

[1 × (% cells 1+) + 2 × (% cells 2+) + 3 × (% cells 3+)]; the final score will range from 0 to 300. An example of the imaging processing using the The BondMax Protocol for IHC analysis of pERK in LGSC FFPE section is shown in Figure 4.

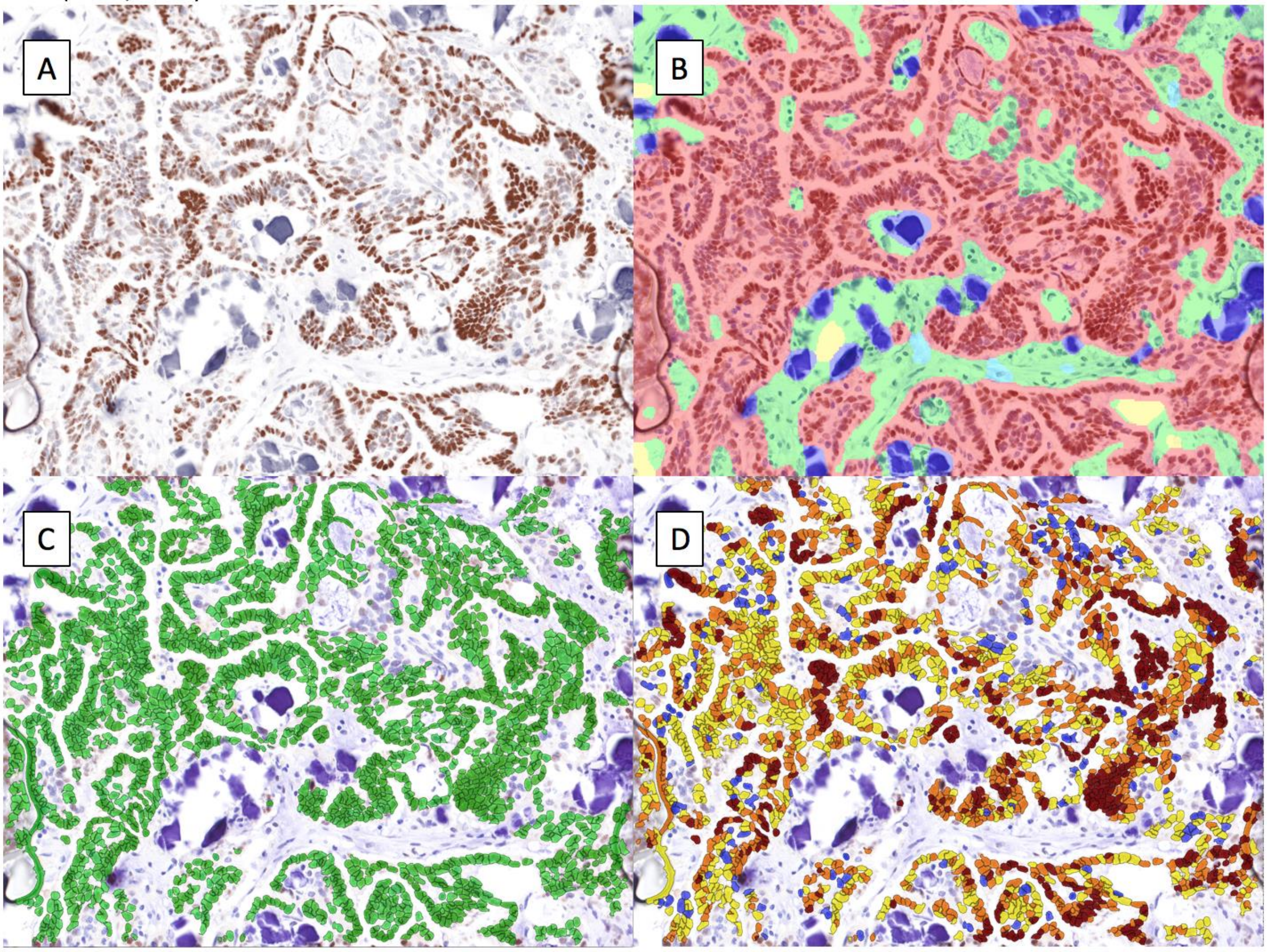

Figure 4. H-score analysis of a LGSC FFPE section after IHC staining of pERK. Image was captured by Vectra 3.0 Automated Quantitative Pathology Imaging System. Image was then analyzed by inForm® imaging analysis software. A, DAB pathology view. B, tissue segmentation view (red, tumor cell region; green, stromal cell region; blue, psammoma body; yellow, non-cellular region). C, cell segmentation view, individual cell was identified. D, scoring view (blue, negative=0; yellow, low=1; orange, moderate=2; brown, strong=3). H-score of this IHC staining was calculated as 172.

To select the immunohistochemical cut-off scores for pERK as a biomarker of tumor response in LGSC, the receiver operating characteristic (ROC) curve analysis will be adapted as described previously. The score with the shortest distance from the curve to the point with both maximum sensitivity and specificity, i.e. the point (0.0, 1.0), will be selected as the cut-off score leading to the greatest number of tumors correctly classified as having or not having the clinical outcome.

<u>Whole Exome sequencing (WES)</u>:
For WES, we will extract DNA from FFPE tissues using the Qiagen FFPE Tissue kit (P/N 56404) using the Qiagen deparaffinization solution (P/N 19093) and finally eluting in 30ul EB buffer. The WES will require at least 100ng of input DNA and as a rule we would QC the FFPE DNA via UV spectroscopy (for 260/280 and 260/230 ratio) and by Qubit for DNA quantitation. In some circumstances if there have been issues with yield or purity at this stage, we have been able to rescue the DNA using the Zymo Clean and Concentrate kit. Finally, we will use a fragment analyser to visualise the DNA and to generate a Genomic Quality Number (GQN), for FFPE if this score is greater than 0.3 then the DNA is of sufficient quality to continue for sequencing library preparation. FFPE DNA samples with sufficient library yield generated will proceed for sequencing using Illumina platform with a sequencing depth of 50X. Potential mutations associated with response can be validated upon CLIA-certified method for confirmation of specific sequencing results.

Depending on the size of the tumor tissue, four FFPE slides will be needed for microdissection of tumor cells. 50x exome capture will be performed using the Illumina TruSeq Exome Library Prep kit. Such depth has been used in a pilot study and has allowed us to robustly detect key mutations present in LGSOC samples (adequate sequence data obtained from 24/24 specimens, see below). Libraries are prepared from each DNA sample using the TruSeq Exome Library Prep kit according to the provided protocol using modifications for working with FFPE sourced material. 100ng of DNA is end-repaired to remove 3’ and 5’ overhangs, and fragment length optimised using sample purification beads. For a limited number of patients with no archival FFPE material or FFPE material is insufficient, 5 mg fresh frozen material will be needed for exome sequencing. **(02-JUN-2020)**

DNA sequencing is carried out on an Illumina NextSeq 550 (Illumina, Inc., San Diego, CA, USA). A 2x75bp sequencing run on the Nextseq 550 using a high-output flow cell is expected to generate up to 400M paired-end (PE) reads (50-60Gb). When multiplexing 12 samples per flow cell we would therefore expect to see up to ~33M reads per sample. If we opt for a 2x75bp sequencing run on the Nextseq 550 using a mid-output flow cell we would expected to generate up to 130M PE reads (16-19Gb). When multiplexing 6 samples per flow cell we would therefore expect to see up to ~22M reads per sample.

DNA sequencing data will undergo informatic based QC and then alignment to the human reference genome (hg38) using in house bioinformatic pipelines built on bwa-0.7.1. Further informatic processing will correct for sequencing artefacts such as duplication and base quality scores will be recalibrated with the Genome Analysis Toolkit (GATK) v4 again using in house informatic pipelines developed within the University of Edinburgh and Gourley lab.

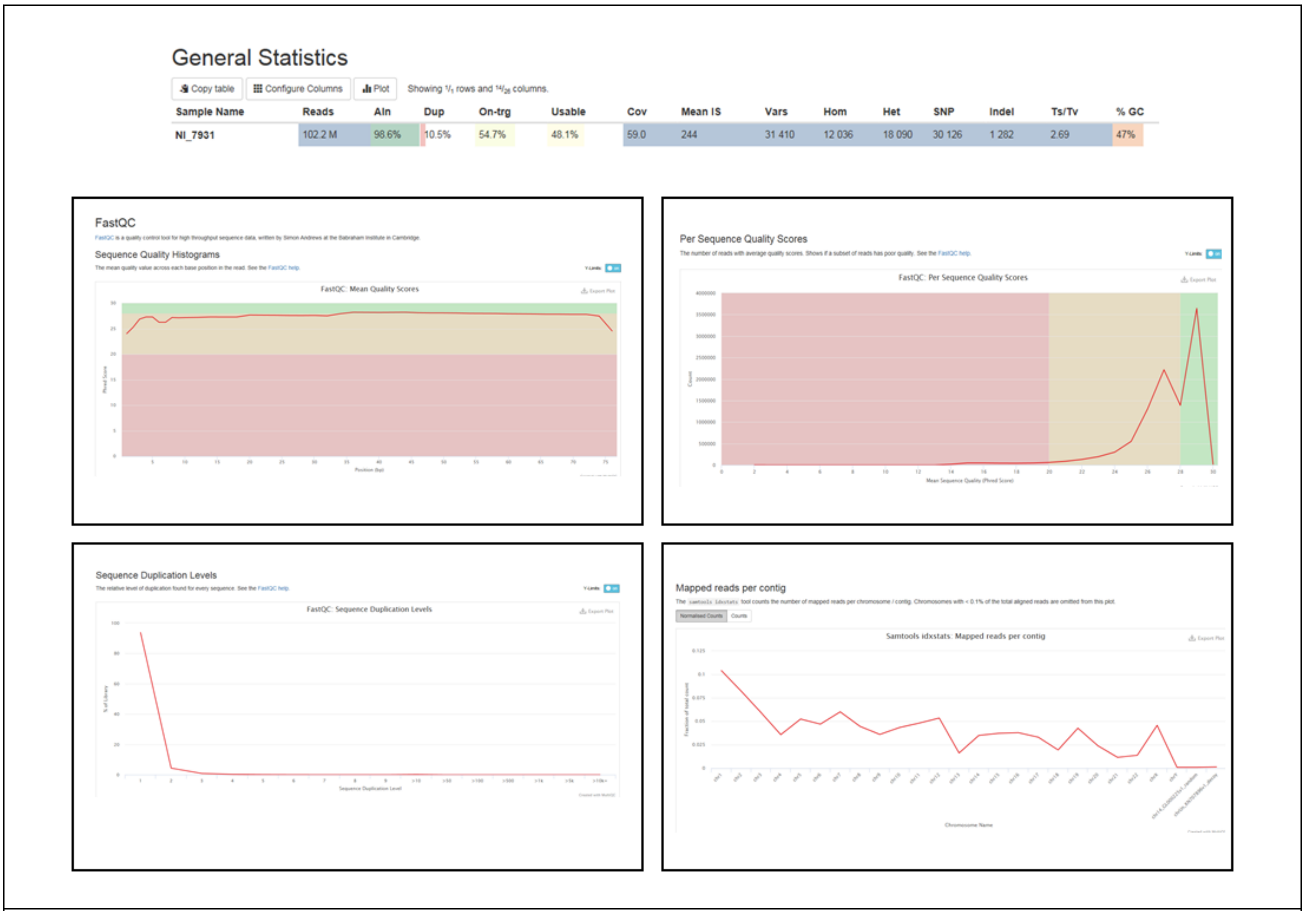

Figure 5. Example data following sequencing QC. Sequence quality scores > 25 indicate good

quality data. Sequence duplication is also checked. Mapped reads can also be checked across the genome to ensure adequate coverage. Data is then trimmed accordingly to remove low quality reads.

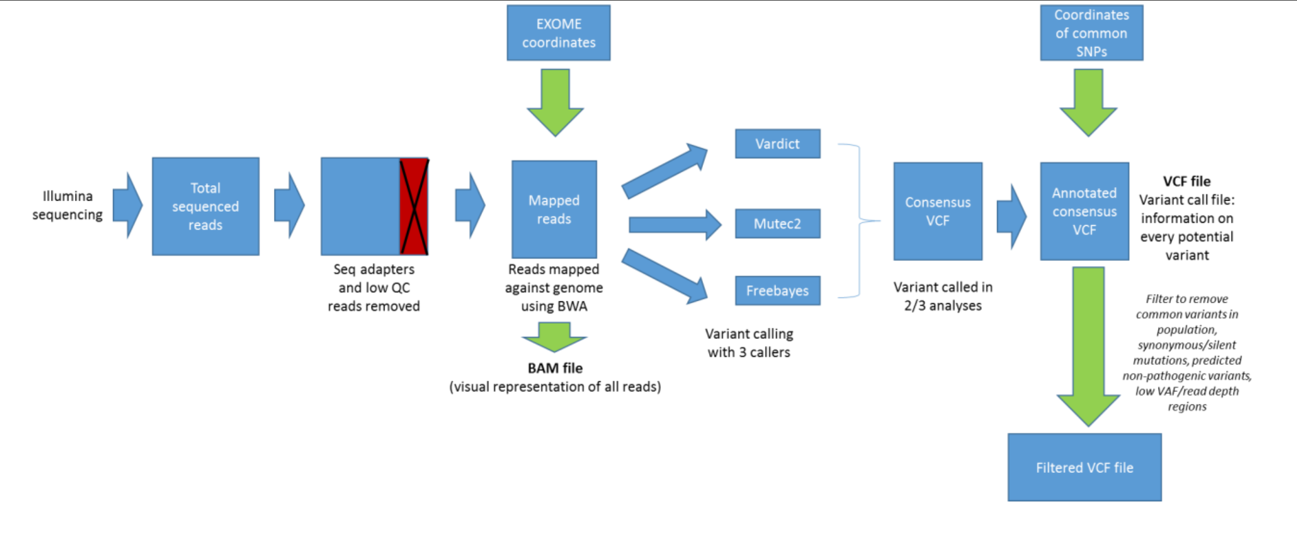

Figure 6. Workflow for WES processing and variant calling informatic pipelines

Detection of somatic single-nucleotide variants (SNVs) and short insertion and deletion (INDEL) variants.

Somatic variant calling will be carried out using an informatics pipeline developed within the Gourley lab based on a majority vote from three variant caller algorithms; VarDict, Mutect2, Freebayes. Filtering for C>T (FFPE artifacts) and G>T (oxidation artifacts) are applied using GATK (Collect Sequencing Artifact Metrics and Filter By Orientation Bias). Data is then filtered to remove common variants in the population (1000 genomes phase 1 snp and indel dataset; (http://www.internationalgenome.org/) and the Exome Aggregation consortium (EXAC) reference datasets (ExAC.0.3.GRCh38 : http://exac.broadinstitute.org/). Variants reported as “silent” or those which are not predicted to result in causal mutations were filtered using the Polymorphism Phenotyping (Polyphen : http://genetics.bwh.harvard.edu/pph/) and Sorting Intolerant from Tolerant (SIFT: http://sift.bii.a-star.edu.sg/) algorithms. Finally, filtering is applied to define high impact mutations where the variant allele frequency of a given mutation was > 10% across regions with a minimum read coverage of 20x. From the resulting data we can look across gene panels of interest as well as carry out discovery based studies.

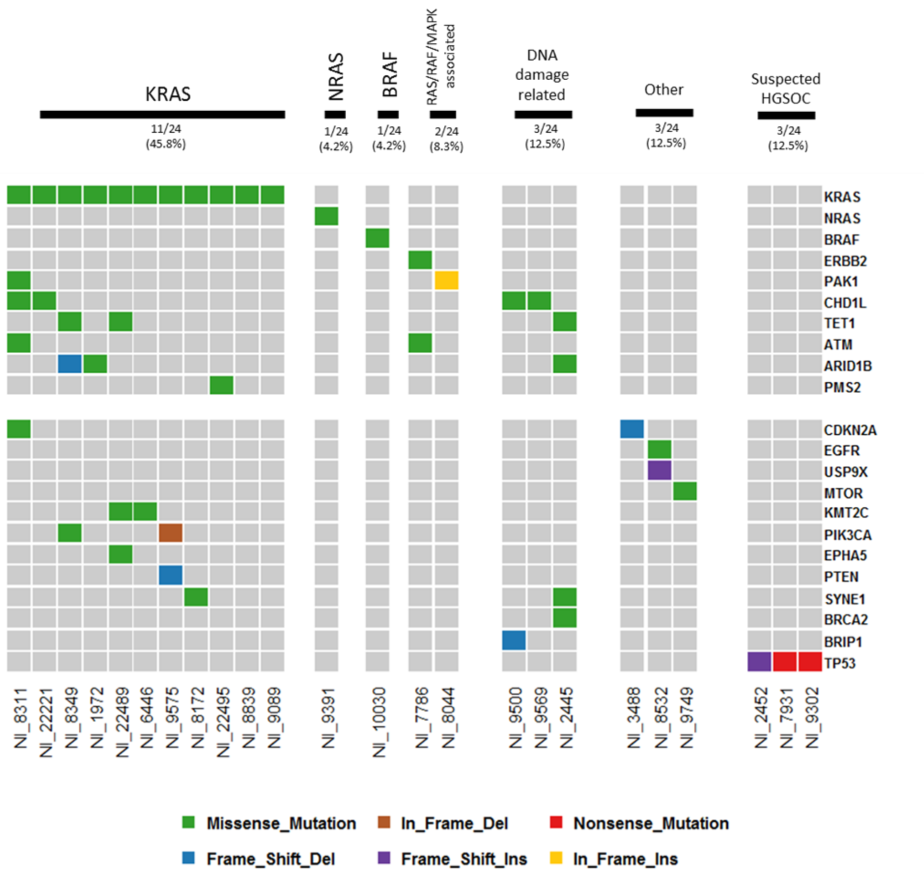

Figure 7. Example “oncoplot” displaying most frequent mutations across genes of interest taken from **existing LGSOC data** generated in the Gourley lab. Key is shown below for mutation types.

Appendix VIII - UK Group Specific Appendix to GOG protocol (GOG-0281) (07/28/14) (12/14/15) (05/31/16) (09/19/16) (01/16/17)

LOGS: A randomized phase II/III study to assess the efficacy of trametinib (GSK 1120212) in patients with recurrent or progressive low-grade serous ovarian cancer of peritoneal cancer
(GOG-0281)

(EudraCT Number: 2013-001627-39)
(NCT02101788)
(NHS GG&C ref: GN12ON121)

UK Group Specific Appendix: Version 7, 05th February 2020

The study is being co-ordinated in UK by Cancer Research UK Clinical Trials Unit, Glasgow on behalf of the National Institute for Health Research (NIHR) Clinical Research Network Cancer and National Cancer Research Institute (NCRI) Gynaecology Clinical Study Group

**UK Study Co-ordinator/:** **Chief Investigator**	Professor Charlie Gourley Email: Charlie.gourley@ed.ac.uk

IMPORTANT INFORMATION

The protocol to which this appendix refers to has been initiated and written by the Gynecologic Oncology Group (GOG)

All technical and administrative aspects of the protocol specific to the UK (registration, responsibilities, safety reporting, etc.) are covered by this UK Group Specific Appendix.

Sponsor

The Sponsor of this clinical trial in the UK is NHS Greater Glasgow and Clyde (NHSGG&C) and The University of Glasgow (GU).

Sponsor Contacts:-

Mrs. Joanne McGarry<br>NHS Greater Glasgow and Clyde<br>Clinical Research and Development Central Office, Dykebar Hospital<br>Ward 11, Grahamston Road<br>Paisley, PA2 7DE<br><br>Tel no: 0141 232 1810<br>Email: joanne.Mcgarry@ggc.scot.nhs.uk	Dr Debra Stuart<br>The University of Glasgow<br>College of Medical, Veterinary and Life Sciences, University of Glasgow, Room 327<br>Wolfson Medical School Building<br>University Avenue<br>Glasgow, G3 8SW<br><br>Tel no: 0141 330 4539<br>Email: debra.stuart@glasgow.ac.uk
**Sponsor Pharmacist**<br>Paula Morrison MRPharms<br>Senior Pharmacist Oncology Clinical Trials<br>NHS Greater Glasgow & Clyde<br>Clinical Research and Development Central Office, Dykebar Hospital, Ward 11, Grahamston Road<br>Paisley, PA2 7DE<br><br>Tel No.: +44 (0)141 314 4019<br>Email: paula.morrison@ggc.scot.nhs.uk	

Contact addresses for UK study co-ordinating centre

UK Study Co-ordinator/:<br>Chief Investigator	Professor Charlie Gourley<br>Edinburgh Cancer Research UK Centre,<br>MRC IGMM,<br>The University of Edinburgh,<br>Crewe Road South,<br>Edinburgh,<br>EH4 2XR<br>Tel no: +44 (0) 131 777 3555<br>Email: charlie.gourley@ed.ac.uk
UK Lead Pathologist:	Dr David Millan<br>Department of Pathology Laboratory<br>Medicine + Facilities Management Building<br>Southern General Hospital<br>1345 Govan Road, Glasgow, G52 4TF<br>Email: david.millan@ggc.scot.nhs.uk
Project Manager:	Karen Carty<br>Cancer Research UK Clinical Trials Unit, Level 0<br>The Beatson West of Scotland Cancer Centre<br>1053 Great Western Road<br>Glasgow, G12 0YN<br>Tel no:+44 (0) 141 301 7197 Fax no:++44 (0) 0141 301 7946<br>Email: karen.carty@glasgow.ac.uk
Clinical Trial Co-ordinator:	Avril Trevethan<br>Cancer Research UK Clinical Trials Unit, Level 0<br>The Beatson West of Scotland Cancer Centre<br>1053 Great Western Road<br>Glasgow, G12 0YN<br>Tel no:++44 (0)141 301 7467 Fax no:++(0)141 301 7946<br>Email: avril.trevethan@glasgow.ac.uk
Clinical Trial Monitor:	Jill Fleming<br>Cancer Research UK Clinical Trials Unit,<br>Quality Assurance Office, Admin Building<br>1055 Great Western Road<br>Glasgow, G12 0XH<br>Tel no:++ 44(0) 141 211 0237<br>Email: jill.fleming@glasgow.ac.uk
Pharmacovigilance:	Lindsey Connery<br>Pharmacovigilance Manager<br>Cancer Research UK Clinical Trials Unit,<br>Level 0, Room 15 Admin Building<br>1055 Great Western Road<br>Glasgow, G12 0XH<br>Tel no. 0141 211 0352 Fax no: 0141 232 2157<br>Email: mvls-ctu-pv@glasgow.ac.uk

UK Signatory Page

Sponsor:

____ __________________

Sponsor’s Representative **Date**

UK Study Coordinator/Chief Investigator:

____ __________________

Professor Charlie Gourley **Date**

Table of Contents

1. Trial Organisation 132

2. Investigator Authorisation Procedure 132

3. Patient Registration & Randomisation Procedure 133

3.1 UK Central Pathological Review (Prospective) 133

3.2 Crossover 134

4. Management and Handling of Investigational Medicinal Products (IMP) 134

4.1 General 134

4.2 Initial Shipment of Trametinib (GSK1120212) 135

4.3 Re-supply ordering of Trametinib (GSK1120212) 135

4.4 Accountability of IMPs 136

4.5 IMP Disposal & Destruction 136

5. Drug Interactions/ Contraindications with Concomitant Medications 136

6. Trial Management and Data Collection 136

6.1 Case report forms 136

6.2 Data Flow 136

7. Assessment of Safety/ Reporting of Serious Adverse Events 137

7.1 Definitions 137

7.2 Detection of Adverse Events 141

7.3 Recording of Adverse Events 141

7.4 Assessment of Adverse Events 142

7.5 Serious Adverse Event Reporting 142

7.6 Identifying SUSARs 142

7.7 Reporting of a SUSAR 142

7.8 Reference Safety Information 143

7.9 Pregnancy Reporting 143

7.10 Development Safety Update Reports 143

8. Study Responsibilities 143

8.1 UK Co Sponsor Responsibilities (NHSGG&C/GU) 144

8.2 CR-UK Clinical Trials Unit (CTU) 144

8.3 Chief Investigator (C.I.) 144

8.4 Participating Site 144

8.5 Principal Investigator (P.I.) 144

9. Liability and Indemnity 144

10. Regulatory Issues 145

10.1 Clinical Trials Authorisation (CTA) 145

10.2 Ethics and Research & Development Approval 145

10.3 Informed Consent 145

10.4 Confidentiality 145

10.5 Quality Assurance/Quality Control 146

10.6 Monitoring 146

10.7 Audits and Inspections 146

11. Patient Information Sheets and Consent Forms 146

12. GP Letter 146

13. Translational Research 147

14. Central Radiographic Image Review 149

14.1 Form 01 Central CT/MRI Scan Assessment Form 151

1. Trial Organisation

- This trial is an Intergroup Trial jointly conducted by Cancer Therapy Evaluation Program (CTEP)/Gynecologic Oncology Group (GOG) from USA and National Institute for Health Research (NIHR) Clinical Research Network Cancer United Kingdom.
- CTEP is overall legal sponsor of the study and the Gynecologic Oncology Group (GOG) is lead coordinating group in this intergroup trial.
- GOG as the lead coordinating group is therefore responsible for overall trial conduct (including trial design, protocol finalisation, trial activation, data management [including the quality control of data], statistical analysis and publication.)
- The Sponsor of this clinical trial in the UK is NHS Greater Glasgow and Clyde (NHSGG&C) and The University of Glasgow (GU).
- NHSGG&C (Legal name Greater Glasgow Health Board [GGHB] and GU have entered into an agreement with CTEP and GOG for the study which outlines the roles and responsibilities of each party in relation to UK participation in the trial.
- In the UK the trial is being run under the auspices of the NIHR Clinical Research Network Cancer/NCRI Gynaecology Clinical Study Group. This study is endorsed by Cancer Research UK (CTAAC), and is part funded by Novartis under the terms of their collaboration with the NIHR Clinical Research Network Cancer.
- The Cancer Research UK Clinical Trials Unit, Glasgow (CTU) is co-ordinating the UK participation in the trial on behalf of NCRI/NIHR Clinical Research Network Cancer, NHS GG&C and GU.
- The Cancer Research UK Clinical Trials Unit, Glasgow will be the main point contact for UK sites. All questions in relation to the trial should be addressed to the Cancer Research UK Clinical Trials Unit.
- The content of this appendix is applicable only for UK participating investigators; this appendix complements or supercedes the corresponding sections in the protocol.
- This trial is an academic trial.

2. Investigator Authorisation Procedure

Investigators will be authorised to register and/or randomise patients to the trial when they have returned the following documents to the Cancer Research UK Clinical Trial Unit, Glasgow and completed the site initiation process for the trial:

- Board/Trust R&D approval letter
- Fully signed Clinical Study Site Agreement.
- Completed Staff Contact and Responsibilities Sheets for all members of staff.
- Up to date, signed and dated CV for the Principal Investigator The CV should detail the qualifications, experience and training (including GCP training) relevant to their role in the study, and should be updated **yearly.**
- Up to date, signed and dates CVs for Co-Investigators and study team must be provided. The CV should detail the qualifications, experience and training (including GCP training) of site personnel relevant to their role in the study, and should be updated every 2 years.
- Copy of GCP certificate for Principal Investigator, Co Investigators and study team (if no formal certificate is available, evidence should be present that GCP training has been undertaken [e.g. register log or email confirmation]. If PI has had no GCP training, this should be arranged as soon as possible, and evidence of this provided).
- Local versions of Patient Information Sheets, Consent Forms and GP Letters on hospital headed paper.
- Full contact details for all site personnel.
- Biochemistry and Haematology normal ranges and laboratory accreditation certificates
- Each site/institution participating in the study requires to hold a valid Federalwide Assurance (FWA) with the Office for Human Resource Protection (OHRP) in the US.

- Completed FDA 1572 Form (wet signature), Financial Disclosure Form and Supplemental Investigator Data Form for Principal Investigator *** These forms required to be updated yearly*** (The Cancer Research UK Clinical Trials Unit will forward these forms on behalf of UK sites along with copy of Principal Investigator's CV to the Pharmaceutical Management Branch (PMB) of CTEP)

Once all of the above documentation has been received and the site initiation process for the site is complete the Cancer Research UK Clinical Trials Unit will inform the NCI Cancer Trials Support Unit (CTSU) to enable the institution to be registered on the authorisation list.

After authorisation of each site, the Cancer Research UK Clinical Trials Unit, Glasgow will take the necessary steps to request a username and password for each site and communicate this to the site.

The site will be notified by email or fax when they are activated and are authorised to recruit patients to the trial.

3. Patient Registration & Randomisation Procedure

UK Investigators will register/randomise patients **ONLY** through the Cancer Research UK Clinical Trials Unit, Glasgow. The Cancer Research UK Clinical Trials Unit will perform the randomisation on behalf of UK sites via the NCI Cancer Trials Support Unit (CTSU) online registration system "OPEN".

Patient registration/randomisations will only be accepted from authorised investigators (see section 2 Investigator authorisation procedure).

Registration forms should be faxed to the Cancer Research UK Clinical Trials Office (Fax no: +44 141 301 7228 during office hours Monday- Friday).Any further details relevant to UK Investigators for the registration & randomisation procedure will be provided at a later time in a separate document.

Please note: prior to registration /randomisation, UK Central Pathological Review is required. Please see below section regarding procedure to be followed for this

3.1 UK Central Pathological Review (Prospective)

Central pathology review is required to take place prior to entry to confirm patient eligibility.

This is **mandatory** for the study.

Process for UK Central Pathology review:

- As patients are identified for the trial and once informed consent has been given, Investigator or designee should contact the Cancer Research UK Clinical Trials Unit, Glasgow immediately to request a screening identifier for the patient.

- Sites should then organise to send 3 H&E stained slides from their pathology department (the 3 most representative slides should be sent) obtained at primary surgery and/or relapse documenting low grade serous carcinoma to the UK Lead Pathologist for the study (Dr David Millan) at the below address to allow central pathology review to take place:

  Dr David Millan, Department of Pathology Laboratory
  Medicine + Facilities Management Building
  Southern General Hospital,1345 Govan Road,

Glasgow, G52 4TF

The slides should be labelled with the screening identifier allocated to the patient and sent with a copy of the patient's pathology report. Personal data of the patient must be anonymised (black out the patient's name and any other identifiable information) and replaced with the screening identifier allocated to the patient.

Please also include the patient's initials and protocol number (GOG-0281) on all pages of the pathology report. A supply of Royal Mail "Safeboxes" for transfer of slides will be provided to sites prior to activation to recruitment to the trial.

- The slides will then be reviewed by the UK pathology review panel for study and agreement reached as to whether the accepted criteria are met. A majority decision will be acceptable.

- The review decision will be emailed by Dr David Millan, to the Cancer Research UK Clinical Trials Unit, Glasgow who will subsequently inform the site.

- Patients who meet the accepted criteria will then be able to be randomised to the trial.

Further instruction for the processing, labelling, handling storage and shipment of these specimens will be provided in the LOGS translational research manual.

3.2 Crossover

In this study if a patient develops progressive disease on Arm A – Control Arm (as defined in study protocol section 8.134), the patient will be given the opportunity to crossover to Arm B -Experimental Arm [Trametinib Arm].

Prior to crossover, the following must occur;

- The patient's progression must be fully documented on the relevant GOG electronic case report forms (CRFs) and submitted via Medidata Rave Electronic Data Entry System (www.imedidata.com) online application which is being used for the study. The relevant CRFs require to be submitted prior to the patient starting crossover treatment.

- All eligibility criteria as defined in study protocol section 3 must be met (with the exception of 3.143, 3.15, and 3.114). This includes requirement that 4 weeks **must** elapse between the end of treatment with Arm A and start of treatment on Arm B. These requirements will be documented on the CRFs.

If the patient meets all of the above noted requirements, she will be able to crossover and commence treatment on Arm B.

4. Management and Handling of Investigational Medicinal Products (IMP)

4.1 General

The investigational medicinal products in this study are:

Arm A Control Arm:

Physician's choice of:

- Letrozole 2.5 mg PO daily continuously
- Tamoxifen 20 mg PO twice daily continuously ***Please note this is not UK standard dose***
- Paclitaxel 80 mg/m2 IV D1, 8, 15
- Pegylated Liposomal Doxorubicin 40 or 50 mg/m2 IV D1
- Topotecan 4.0 mg/m2 D1, 8, 15

Each cycle is 28 days.

Arm B Experimental Arm:
- Trametinib (GSK1120212) 2.0 mg PO daily continuously

Each cycle is 28 days.

Trametinib (GSK1120212) will be provided free-of-charge by Novartis and supplied & distributed by Catalent to UK sites for use in study. It will be labelled as investigational medicinal product clinical trial stock. The presentation may be either fully packaged clinical trial supplies or commercial stock over-labelled for clinical trial use, in accordance with current UK regulatory requirements. The transition from fully packaged clinical trial stock to over-labelled commercial stock will be managed by the UK Sponsor in conjunction with Novartis when the supplies of fully packaged clinical trial stock for the study have been used which is estimated to be towards end of 2016.

All IMPs for use in the trial with the exception of Trametinib (GSK1120212) should be taken from routine pharmacy stock; there is no provision for funding, reimbursement or discounted stock. Site stock of the IMPs in the control arm will not require ring fencing, but all prepared IMP for administration to trial subjects should be labelled at site in accordance with all applicable regulatory requirements with the addition of Annexe 13-compliant labels.

Although specific formulations are mentioned in the protocol for the IMPs in the control arm, UK sites are permitted to use their locally approved formulations. This must be confirmed to the Cancer Research UK Clinical Trials Unit, Glasgow during the initiation process.

Chemotherapy doses may be recalculated every cycle during treatment if it is local practice to do so e.g. automatic updates by electronic prescribing systems. Where it is not local practice to recalculate every cycle the doses MUST be recalculated if the subject's weight changes by greater than or equal to 10% from baseline.

BSA calculations should be performed as routine local practice and capped at $2.0m^2$.

Chemotherapy doses may be dose-banded if it is routine local practice to do so. The Cancer Research UK Clinical Trials Unit, Glasgow must be informed of this during the initiation process.

The Investigator or a delegated individual (e.g. pharmacist) must ensure all IMPs are stored and dispensed in accordance with study protocol, local standard operating procedures, applicable regulatory requirements and the information contained within the current summary of product characteristics/investigator brochure for each product

Full instructions regarding management, labelling, accountability and destruction of Trametinib (GSK1120212) and the control arm IMPs will be given in the IMP Management Document for the study.

4.2 Initial Shipment of Trametinib (GSK1120212)

The Cancer Research UK Clinical Trials Unit, Glasgow will trigger the initial supply of Trametinib (GSK1120212) at the time of site activation when all necessary documentation received and the initiation process completed as outlined in section 2 of this appendix.

Drug supply will be addressed to the responsible Pharmacist recorded on Staff Contact and Responsibilities sheet for the site. Thereafter the site will be responsible for ensuring they have sufficient supplies of Trametinib (GSK1120212) and the stock is within date and there is stock rotation of supplies to ensure the shortest expiry date are used first. The size of the initial shipment will vary depending on anticipated recruitment rates at each site.

4.3 Re-supply ordering of Trametinib (GSK1120212)

After the initial shipment, Trametinib (GSK1120212) supplies will be managed using manual ordering systems. Further supplies can be obtained by completing the **Clinical Trials Request and Receipt Form** and forwarding by fax to the Cancer Research UK Clinical Trials Unit, Glasgow who will order the supplies on behalf of the site. The order form should be signed by a member of pharmacy clinical trial staff at the site who has delegated IMP management related responsibilities. The original signed order form should be retained in the pharmacy site file.

Orders will take approximately 5 working days from the date of request to delivery at site. Please note deliveries will only be made to site pharmacies Monday to Friday.

4.4 Accountability of IMPs

IMP accountability logs will be provided by the UK Sponsor for recording the movement of all IMPs used within the study. Each patient taking part in the study should have a log maintained of the IMP administered, the date of administration, the cycle number, the dose administered and the brand, batch number and expiry date of the product administered.

Local documentation for accountability may be permitted after approval by the Cancer Research UK Clinical Trials Unit, Glasgow.

Full accountability records for Trametinib (GSK1120212) are required, documenting receipt of bulk supplies as well as patient dispensing. These must be accurately maintained and updated at the time of each dispensing or other drug movement for the duration of the study and should be kept in the study pharmacy file.

4.5 IMP Disposal & Destruction

For the control arm, used or partially used vials, dose-banded infusions or syringes may be disposed of at site according to local hospital policy with no additional accountability required. Oral products will require reconciliation of empty or part-used containers of IMP returned from patients, within the accountability logs, as a measure of patient compliance, before disposal as per local policy.

Destruction of Trametinib (GSK1120212) un-dispensed stock, if necessary, should be undertaken after the Sponsor has given written permission, in line with local policies and procedures and a destruction log completed. A destruction log will be provided for use.

5. Drug Interactions/ Contraindications with Concomitant Medications

Please refer to the current Summary of Product Characteristics/ Investigator Brochure for the products for details on drug interactions and contraindications with concomitant medications.

6. Trial Management and Data Collection

6.1 Case report forms

6.2 Data Flow

Data collection for this study will be done exclusively through the Medidata Rave clinical data management system. For full instructions please see the data submission section of the protocol: section 10.2 GOG Data Management.

In summary the data flow for the study will be as follow

- All queries will be sent from GOG Statistical and Data Centre (SDC) to the participating sites via Medidata Rave Electronic Data Entry System (www.imedidata.com) online application.
- Investigators/ Authorised personnel will submit GOG electronic case report forms via Medidata Rave Electronic Data Entry System (www.imedidata.com) online application.
- The GOG SDC is responsible for raising validation checks and for overall quality control of trial data.

7. Assessment of Safety/ Reporting of Serious Adverse Events

Safety assessments will be performed in line with the guidance specified in The Medicines for Human Use (Clinical Trials) Regulations 2004.

7.1 Definitions

These definitions apply to all study participants from the first dose of study medication.

Adverse Event (AE)
An adverse event (AE) is any untoward medical occurrence in a subject to whom a medicinal product has been administered, including occurrences which are not necessarily caused by or related to that product.

Adverse Reaction (AR)
An adverse reaction (AR) is any untoward and unintended occurrence in a subject to whom a medicinal product has been administered which is thought to be caused by or related to that product.

Serious Adverse Event (SAE)
A serious adverse event (SAE) is defined as any of the following, whether or not considered related to the trial treatment:

- Results in Death
- Life-threatening (i.e. at the time of the event)*
- Requires inpatient hospitalisation or prolongation of existing hospitalisation**
- Results in persistent or significant disability or incapacity
- Is a congenital anomaly/birth defect
- Is considered medically significant by the Investigator***

*Life threatening means that the patient was at immediate risk of death from the event as it occurred. It does not include an event that, had it occurred in a more serious form, might have caused death.

**Requires in-patient hospitalisation should be defined as a hospital admission required for treatment of an AE.

***Considered medically significant by the Investigator are events that may not result in death, are not life threatening, or do not require hospitalisation, but may be considered a serious adverse experience when, based upon appropriate medical judgement, the event may jeopardise the patient and may require medical or surgical intervention to prevent one of the outcomes listed above.

Please note in addition to above definitions of an SAE for this study there are additional SAE reporting requirements.

A table is provided below of the Comprehensive Adverse Event and Potential Risks list (CAEPR) for Trametinib (GSK1120212). The CAEPR provides a single list of reported and/or potential adverse events (AE) associated with an agent using a uniform presentation of events by body system. In addition to the comprehensive list, a subset, the Specific Protocol Exceptions to Expedited Reporting (SPEER), appears in a separate column and is identified with bold and italicized text. This subset of AEs (SPEER) is a list of events that are protocol specific exceptions which require to be reported as SAEs (except as noted below). *Frequency is provided based on 1111 patients.* Below is the CAEPR for trametinib dimethyl sulfoxide (GSK1120212B,).

> **NOTE**: Report AEs on the SPEER as an SAE **<u>ONLY IF</u>** they exceed the grade noted in parentheses next to the AE in the SPEER. If this CAEPR is part of a combination protocol using multiple investigational agents and has an AE listed on different SPEERs, use the lower of the grades to determine if expedited reporting is required.

Version 2.6, October 10, 2019[1]

Adverse Events with Possible Relationship to Trametinib (GSK1120212B) (CTCAE 5.0 Term) [n= 1111]			Specific Protocol Exceptions to Expedited Reporting (SPEER)
**Likely (>20%)**	**Less Likely (<=20%)**	**Rare but Serious (<3%)**	
BLOOD AND LYMPHATIC SYSTEM DISORDERS			
	Anemia		***Anemia (Gr 3)***
CARDIAC DISORDERS			
		Heart failure	
		Left ventricular systolic dysfunction	
	Sinus bradycardia		
EYE DISORDERS			
	Blurred vision		
	Dry eye		
		Eye disorders - Other (chorioretinopathy also known as retinal pigment epithelial detachment)	
		Eye disorders - Other (retinal vein occlusion)	
	Eye disorders - Other (visual disorders)[2]		
		Papilledema	
	Periorbital edema		
GASTROINTESTINAL DISORDERS			
	Abdominal pain		***Abdominal pain (Gr 2)***
		Colitis	
		Colonic perforation	
	Constipation		***Constipation (Gr 2)***
Diarrhea			***Diarrhea (Gr 3)***
	Dry mouth		***Dry mouth (Gr 2)***
	Dyspepsia		***Dyspepsia (Gr 2)***
	Mucositis oral		***Mucositis oral (Gr 3)***
Nausea			***Nausea (Gr 3)***
	Vomiting		***Vomiting (Gr 3)***
GENERAL DISORDERS AND ADMINISTRATION SITE CONDITIONS			
	Chills		***Chills (Gr 2)***
	Edema face		
Fatigue			***Fatigue (Gr 3)***
	Fever		***Fever (Gr 2)***
Generalized edema[3]			***Generalized edema[3] (Gr 2)***
IMMUNE SYSTEM DISORDERS			
	Allergic reaction[4]		
INFECTIONS AND INFESTATIONS			
	Folliculitis		***Folliculitis (Gr 2)***
	Lung infection		
	Paronychia		***Paronychia (Gr 2)***
	Skin infection		***Skin infection (Gr 2)***
INVESTIGATIONS			
	Alanine aminotransferase increased		***Alanine aminotransferase increased (Gr 3)***
	Alkaline phosphatase increased		***Alkaline phosphatase increased (Gr 2)***
	Aspartate aminotransferase increased		***Aspartate aminotransferase increased (Gr 3)***
	CPK increased		
	Ejection fraction decreased		

Adverse Events with Possible Relationship to Trametinib (GSK1120212B) (CTCAE 5.0 Term) [n= 1111]			Specific Protocol Exceptions to Expedited Reporting (SPEER)
**Likely (>20%)**	**Less Likely (<=20%)**	**Rare but Serious (<3%)**	
METABOLISM AND NUTRITION DISORDERS			
	Anorexia		***Anorexia (Gr 3)***
	Dehydration		***Dehydration (Gr 3)***
	Hypoalbuminemia		
	Hypomagnesemia		***Hypomagnesemia (Gr 2)***
	Hyponatremia		***Hyponatremia (Gr 3)***
MUSCULOSKELETAL AND CONNECTIVE TISSUE DISORDERS			
	Arthralgia		
	Back pain		***Back pain (Gr 2)***
	Pain in extremity		***Pain in extremity (Gr 2)***
		Rhabdomyolysis	
NERVOUS SYSTEM DISORDERS			
	Dizziness		***Dizziness (Gr 2)***
	Headache		***Headache (Gr 2)***
RESPIRATORY, THORACIC AND MEDIASTINAL DISORDERS			
	Cough		***Cough (Gr 2)***
	Dyspnea		***Dyspnea (Gr 3)***
		Pneumonitis	
SKIN AND SUBCUTANEOUS TISSUE DISORDERS			
	Alopecia		***Alopecia (Gr 2)***
	Dry skin		***Dry skin (Gr 2)***
	Nail changes		
		Palmar-plantar erythrodysesthesia syndrome	
	Pruritus		***Pruritus (Gr 2)***
		Skin and subcutaneous tissue disorders - Other (drug reaction with eosinophilia and systemic symptoms [DRESS])	
Skin and subcutaneous tissue disorders - Other (rash)[5]			***Skin and subcutaneous tissue disorders - Other (rash)[5] (Gr 3)***
		Stevens-Johnson syndrome[6]	
VASCULAR DISORDERS			
	Hypertension		***Hypertension (Gr 3)***
		Thromboembolic event (venous)	
	Vascular disorders - Other (hemorrhage)[7]		

[1]This table will be updated as the toxicity profile of the agent is revised. Updates will be distributed to all Principal Investigators at the time of revision. The current version can be obtained by contacting PIO@CTEP.NCI.NIH.GOV. Your name, the name of the investigator, the protocol and the agent should be included in the e-mail.

[2]Visual disorders include visual disturbance that can be associated with conjunctival hemorrhage, corneal graft rejection, cyclitis, eye nevus, halo vision, iritis, macular edema, retinal hemorrhage, visual acuity reduced, visual impairment, and vitreous detachment.

[3]Generalized edema includes edema, lymphedema, and edema limbs.

[4]Hypersensitivity (allergic reactions) may present with symptoms such as fever, rash, increased liver function tests, and visual disturbances.

[5]Skin and subcutaneous tissue disorders - Other (rash) may include rash, rosacea, rash acneiform, erythematous rash, genital rash, rash macular, exfoliative rash, rash generalized, erythema, rash papular, seborrhoeic dermatitis, dermatitis psoriasiform, rash follicular, skin fissures, and skin chapped.

[6]Stevens-Johnson syndrome has been observed in patients treated with trametinib and dabrafenib combination.

[7]The majority of hemorrhage events were mild. Major events, defined as symptomatic bleeding in a critical area or organ (e.g., eye, GI hemorrhage, GU hemorrhage, respiratory hemorrhage), and fatal intracranial hemorrhages have been reported.

Adverse events reported on trametinib dimethyl sulfoxide (GSK1120212B) trials, but for which there is insufficient evidence to suggest that there was a reasonable possibility that trametinib dimethyl sulfoxide (GSK1120212B) caused the adverse event:

BLOOD AND LYMPHATIC SYSTEM DISORDERS - Disseminated intravascular coagulation; Febrile neutropenia; Leukocytosis
CARDIAC DISORDERS - Atrial fibrillation; Cardiac arrest; Myocardial infarction; Restrictive cardiomyopathy; Sinus tachycardia
EYE DISORDERS - Corneal ulcer; Eyelid function disorder; Flashing lights; Floaters; Glaucoma; Photophobia
GASTROINTESTINAL DISORDERS - Ascites; Duodenal ulcer; Esophageal necrosis; Esophageal ulcer; Esophagitis; Gastric hemorrhage[7]; Gastric ulcer; Gastritis; Gastrointestinal disorders - Other (intestinal obstruction); Gastrointestinal disorders - Other (pneumatosis intestinalis); Gastrointestinal fistula; Gingival pain; Hemorrhoidal hemorrhage[7]; Ileus; Obstruction gastric; Pancreatitis; Small intestinal obstruction
GENERAL DISORDERS AND ADMINISTRATION SITE CONDITIONS - Flu like symptoms; General disorders and administration site conditions - Other (axillary pain); Localized edema; Malaise; Non-cardiac chest pain; Pain
HEPATOBILIARY DISORDERS - Cholecystitis; Hepatic failure; Hepatic pain; Hepatobiliary disorders - Other (hepatic encephalopathy)
INFECTIONS AND INFESTATIONS - Biliary tract infection; Catheter related infection; Device related infection; Endocarditis infective; Enterocolitis infectious; Hepatitis viral; Infections and infestations - Other (abscess limb); Infections and infestations - Other (necrotizing fasciitis); Infections and infestations - Other (oral infection); Pharyngitis; Sepsis; Upper respiratory infection; Urinary tract infection
INJURY, POISONING AND PROCEDURAL COMPLICATIONS - Bruising
INVESTIGATIONS - Blood bilirubin increased; Blood lactate dehydrogenase increased; Creatinine increased; Electrocardiogram QT corrected interval prolonged; GGT increased; Lipase increased; Lymphocyte count decreased; Platelet count decreased; Serum amylase increased; White blood cell decreased
METABOLISM AND NUTRITION DISORDERS - Hyperglycemia; Hyperkalemia; Hyperphosphatemia; Hyperuricemia; Hypocalcemia; Hypoglycemia; Hypokalemia
MUSCULOSKELETAL AND CONNECTIVE TISSUE DISORDERS - Generalized muscle weakness; Muscle cramp; Musculoskeletal and connective tissue disorder - Other (compression fracture); Myalgia; Neck pain
NEOPLASMS BENIGN, MALIGNANT AND UNSPECIFIED (INCL CYSTS AND POLYPS) - Tumor hemorrhage[7]; Tumor pain
NERVOUS SYSTEM DISORDERS - Dysgeusia; Encephalopathy; Intracranial hemorrhage[7]; Lethargy; Nervous system disorders - Other (diplopia); Seizure; Somnolence; Stroke; Syncope; Transient ischemic attacks
PSYCHIATRIC DISORDERS - Anxiety; Confusion; Delirium; Depression; Hallucinations; Insomnia; Personality change
RENAL AND URINARY DISORDERS - Acute kidney injury; Cystitis noninfective; Dysuria; Hematuria; Proteinuria; Urinary incontinence
REPRODUCTIVE SYSTEM AND BREAST DISORDERS - Vaginal fistula; Vaginal hemorrhage[7]
RESPIRATORY, THORACIC AND MEDIASTINAL DISORDERS - Bronchopulmonary hemorrhage[7]; Hypoxia; Laryngeal edema; Oropharyngeal pain; Pleural effusion; Pneumothorax; Productive cough; Pulmonary hypertension; Respiratory failure; Sinus disorder

SKIN AND SUBCUTANEOUS TISSUE DISORDERS - Bullous dermatitis; Photosensitivity; Purpura; Skin and subcutaneous tissue disorders - Other (erythema nodosum); Skin ulceration; Urticaria
VASCULAR DISORDERS - Hematoma; Hot flashes; Hypotension

Note: Trametinib (GSK1120212B) in combination with other agents could cause an exacerbation of any adverse event currently known to be caused by the other agent, or the combination may result in events never previously associated with either agent.

Serious Adverse Reaction (SAR)
A serious adverse reaction (SAR) is a SAE that may be related to trial treatment. The assessment of "relatedness" is primarily the responsibility of the Principal Investigator (PI) at site or agreed designee. [1]****NOTE**: SAEs will be considered related if the SAE is documented as possibly, probably or definitely related to protocol treatment. SAEs will be considered unrelated if the SAE is documented as unrelated or unlikely related to protocol treatment. ** The assessment of relatedness is made using the following:

**Relationship**	**Attribution**	**Description**
Unrelated to protocol treatment[1]	**Unrelated**	There is no evidence of any causal relationship.
	**Unlikely**	There is doubtful evidence of any causal relationship.
Related to protocol treatment[1]	**Possible**	There is some evidence to suggest a causal relationship (e, g. the event occurs within a reasonable time after administration of the trial medication). However the influence of other factors may have contributed to the event (e.g. the patient's clinical condition, other concomitant treatments).
	**Probable**	There is evidence to suggest a causal relationship and the influence of other factors in unlikely.
	**Definitely**	There is clear evidence to suggest a causal relationship and other possible contributing factors can be ruled out.

Suspected Unexpected Serious Adverse Reaction (SUSAR)
A Suspected Unexpected Serious Adverse Relation (SUSAR) is any suspected SAR that is unexpected. Unexpected is any reaction that is not a known reaction listed in the section of the current regulatory approved Comprehensive Adverse Event and Potential Risks list (CAEPR)or Summary of Product Characteristics (SmPC), which is acting as the reference safety information (RSI) for the trial treatments. Please note the version of the RSI that has regulatory approval may not be the most up-to-date version of the CAEPR or SmPC that has been provided to Investigators for advice on the clinical management of their trial patients.

7.2 Detection of Adverse Events

Participants will be asked at each study visit about the occurrence of AEs since their last visit.
AEs will be recorded, notified, assessed, reported, analysed and managed in accordance with the Medicines for Human Use (Clinical Trials) Regulations 2004 (as amended) and the study protocol.
AEs must be recorded as they are reported, whether spontaneously volunteered or in response to questioning about well-being at trial visits. The questioning about AEs will cover the current visit as well as the period of time between the previous and the current visit. All AEs must be documented in the patient's medical records whether they are required to be recorded in the CRF or not.

7.3 Recording of Adverse Events

Full details of AEs including the nature of the event, start and stop dates, severity, relationship to study drug and outcome will be recorded in the patient's medical records and on the study case report form as required by the study protocol. All AEs must be followed until resolution, or for at least 30 days after discontinuation of study medication, whichever comes

first or until toxicity has resolved to baseline or ≤ grade 1, or until the toxicity is considered to be irreversible. Perceived lack of efficacy is not an AE.

An exacerbation of a pre-existing condition is an AE.

7.4 Assessment of Adverse Events

All AEs and toxicities must be coded and graded according to the NCI Common Terminology Criteria for Adverse Events (NCI-CTCAE) Version 4.0. A copy of the CTCAE version 4.0 can be downloaded from the CTEP website (http://ctep.cancer.gov).
AEs must be assessed for seriousness, causality and severity. This assessment is the responsibility of the PI (or designee). The assessment of expectedness for regulatory reporting will be undertaken by the CTU and CI.

7.5 Serious Adverse Event Reporting

For guidance on completing the initial and follow up serious adverse event report forms please refer to the serious adverse event form completion guidelines, which will be provided to UK sites by the Pharmacovigilance Office, Cancer Research UK Clinical Trials Unit, Glasgow

If a Serious Adverse Event occurs, a Serious Adverse Event form should be completed and faxed within 24 hours of becoming aware of the event to:

Pharmacovigilance Office, CR-UK CTU, Glasgow
Fax No: +44 (0) 141 301 7213
Tel No: +44(0) 141 301 7211/7953/7209

The UK Chief Investigator will receive notification, by email, of all UK SAEs received.

SAEs must be reported locally by the Principal Investigator at each site in accordance with the local practice at their site (i.e. R&D Office).

A follow-up report must be completed when the SAE resolves, is unlikely to change, or when additional information becomes available. If the SAE is a suspected SUSAR then follow up information must be provided as quickly as possible and as requested by the CR-UK CTU and Chief Investigator. All follow-up information is required to be reported as per the SAE Completion Guidelines.

SAEs are required to be reported from randomisation for up to 30 days after last administration of study treatment. Any SAE that occurs after 30 days post treatment (with no time limit) is also required to be reported if the Investigator thinks that the SAE is related to protocol treatment (is a SAR), or is medically important.

The Pharmacovigilance Department of the CR-UK CTU, Glasgow will enter all UK SAE reports into AdEERs/CTEP-AERS.

For any questions relating to SAE reporting, please contact the pharmacovigilance team at CR-UK CTU, Glasgow.

7.6 Identifying SUSARs

The assessment of expectedness for SAEs and regulatory reporting will be undertaken by the CR-UK CTU and UK Chief Investigator based on the section of the trial Reference Safety Information (RSI) that has regulatory approval at the time the SAE is received.

7.7 Reporting of a SUSAR

CR-UK CTU on behalf of the UK (Co) Sponsors is responsible for the expedited reporting of all SUSARs to the required Regulatory Authorities, Main Research Ethics Committee, PI at trial sites and the trial Sponsor(s) as well as pharmaceutical company (if applicable).

- Fatal or life threatening SUSARs will be reported within 7 days of the CR-UK CTU receiving the first notification of the unexpected event. Any additional information will be reported within eight days of sending the initial report
- All other SUSARs will be reported within 15 days of the CR-UK CTU receiving the first notification of the unexpected reaction.

7.8 Reference Safety Information

The Comprehensive Adverse Event and Potential Risks list (CAEPR) for Trametinib (GSK1120212) as detailed in study protocol section 4.612 and UK appendix to protocol section 7.1 will act as the RSI for the trial for trametinib (GSK1120212). The CAEPR with current regulatory approval for use in the trial will be used to assess SAE reports to identify SUSARs.

The contents of Section 4.8 Undesirable Effects in the Summary of Product Characteristics (SmPC) identified by UK Sponsor for:

- Letrozole
- Tamoxifen
- Paclitaxel
- Pegylated Liposomal Doxorubicin
- Topotecan

Will act as the reference safety information for the trial. The UK Sponsor is responsible for identifying and informing the CR-UK CTU of updates to the reference SmPC. The SmPC with current regulatory approval for the trial will be used to assess SAE reports to identify SUSARs

7.9 Pregnancy Reporting

Pregnancy occurring in a clinical trial participant while not considered an AE or a SAE, requires monitoring and follow-up. The Investigator must collect pregnancy information for female trial subjects. This includes subjects who become pregnant while participating in a clinical trial of an investigational medicinal product or during a stage where the foetus could have been exposed to the IMP.

Any pregnancy occurring in a female subject who becomes pregnant while participating in a trial will be reported by the Principal Investigator (PI) to the Pharmacovigilance Office of the Cancer Research UK Clinical Trials Unit, Glasgow using the Pregnancy Notification Form (PNF). This notification must be made within 2 weeks of the PI first becoming aware of the pregnancy. The PI will update the PNF with the outcome of the delivery or if there is a change in the subject’s condition such as miscarriage. The updated PNF must be sent to Pharmacovigilance Office of the Cancer Research UK Clinical Trials Unit, Glasgow as soon as the information becomes available,

7.10 Development Safety Update Reports

Development Safety Update Reports (DSURs) will be prepared and submitted by the UK Chief Investigator and CR-UK CTU in conjunction with GOG/CTEP on behalf of the UK sponsor. DSURs will be submitted to the Regulatory Authorities, Main Research Ethics Committee, Trial Sites, Trial Sponsor(s) and Pharmaceutical Company on the anniversary of obtaining the UK Clinical Trial Authorisation or aligned to the Investigational New Drug (IND) application.

8. Study Responsibilities

The co-sponsors of this clinical trial in the UK are NHS Greater Glasgow and Clyde (NHSGG&C) and The University of Glasgow (GU).

Prior to study initiation, a non-commercial funded clinical trial co-sponsorship agreement will be put in place between NHS GG&C and GU. The role and liabilities each organisation will take under The Medicines for Human Use (Clinical Trials) Regulations, 2004 SI 2004: 1031 are laid out in this agreement signed by both organisations. GU shall be responsible for carrying out the obligations and responsibilities set out in the aforementioned agreement, and shall be deemed the “sponsor” for the purposes of, Part 3 of the regulations in relation to the study. NHS GG&C shall be responsible for carrying out the obligations and responsibilities set out in the agreement, and shall be deemed the “sponsor” for the purposes of, Parts 4,5,6 and 7 of the Regulations in relation to the study.

Prior to study initiation, an intergroup agreement will be put in place between CTEP, GOG and NHS GG&C and GU. The roles and responsibilities of each party in relation to UK participation in the trial are laid out in this agreement signed by all three organisations.

A Clinical Study Agreement will be put in place between NHS GG&C and each of the participating sites. This agreement outlines the responsibilities of each party’s responsibilities in the running of the trial; the co-sponsors, the Chief Investigator (C.I.), the Cancer Research UK Clinical Trials Unit, Glasgow (CTU), the Principal Investigator (P.I.) at the Participating Site and the Participating Site. In summary, they are as follows:

8.1 UK Co-Sponsor Responsibilities (NHSGG&C/GU)

The Sponsor’s responsibilities will be for Authorisation and Ethics Committee opinion, GCP and Conduct and Pharmacovigilance. In the UK the majority of the Sponsor’s responsibilities have been delegated to the CI who performs these via the CTU as the co-ordinating centre for the study in the UK. As such, the main role of the Sponsor is to ensure that the CI and CTU fulfil their responsibilities as outlined in the Clinical Study Agreement and to ensure that any identified “risks” either have controls or action points put in place.

8.2 CR-UK Clinical Trials Unit (CTU)

The CTU is responsible for the co-ordination of the clinical trial in the UK. This includes all regulatory submissions (ethics, R&D and CTA), all administration relating to the submissions and any amendments, circulation of all correspondence to participating sites, ongoing communication with participating sites, management of SAE/SUSAR reporting, and where applicable the management of any financial arrangements (e.g. payments to sites for pathology samples etc).

8.3 Chief Investigator (C.I.)

The CI has delegated the majority of his/her responsibilities to the CTU. The C.I. is directly responsible for ensuring the protocol and any amendments are in place, for review of SAE forms and determination of whether they meet the criteria for a SUSAR, and to provide advice and recommendations on medical issues that arise involving the management of the patients on the study. As the CI is external to the Co Sponsors an agreement will be put in place between the CI’s employer and the Co-Sponsors for this study to outline the responsibilities of each party.

8.4 Participating Site

The Participating Site is responsible for the management of the trial within their site. This includes ensuring local management approval has been given, ensuring the study is conducted according to ICH GCP requirements, and ensuring the appropriate insurance or indemnity is in place. The Participating Site is also responsible for arranging access for on-site monitoring and auditing as identified in the study protocol and also for regulatory inspections.

8.5 Principal Investigator (P.I.)

The P.I. is responsible for the delegation of study activities within their unit and ensuring all personnel are adequately trained and qualified to carry out their responsibilities. The P.I will be required to provide evidence of GCP training (usually a certificate) or undergo the required GCP training. Regarding the management of patients within their site, the P.I. is responsible for the safety and well being of trial patients, reporting any deviations from the protocol to the CTU as well as any SAEs or safety issues. Full details of the responsibilities of the P.I. are outlined in the Clinical Study Agreement. Two original copies of this will be held – one with the sponsor and the other at the Participating Site. A photocopy of the signed agreement will also be held within the CTU.

9. Liability and Indemnity

The Health Board/NHS Trust at each participating site is responsible for the following:

1. Acts and omissions of its own staff and others engaged by it, including the Clinical Trials Unit and PI;
2. Ensuring the appropriate insurance administered by the National Health Service Litigation Authority is in place;
3. Ensuring any non-NHS employee involved in the clinical trial has an Honorary Contract with the Trust to cover access to patients and liability arrangements.

These responsibilities are outlined and agreed within the Clinical Study Agreement.

No special insurance is in place for patients in this study other than standard NHS liability insurance providing indemnity against clinical negligence. This does not provide cover for non-negligence e.g. harm caused by an unexpected side effect of participating in a study.

10. Regulatory Issues

10.1 Clinical Trials Authorisation (CTA)

On behalf of the Co-Sponsor the Cancer Research UK Clinical Trials Unit, Glasgow will apply to the MHRA for a clinical trials authorisation (CTA) to conduct the trial in the UK and will also be responsible for the maintenance of the CTA.

10.2 Ethics and Research & Development Approval

Ethics favourable opinion will be sought for the study from a Main REC prior to commencement of this trial. Further to that approval each participating site will be responsible for obtaining their own local approval by submitting an SSI to their appropriate R&D department for management approval.

10.3 Informed Consent

Consent to enter the study must be sought from each participant only after full explanation has been given, the participant has been given an information sheet and a minimum of 24 hours to consider trial participation. Signed participant consent must be obtained, the consent forms should also be signed by the person undertaking the consent procedure at site, who must be detailed on the Staff Contact and Responsibility Log as having this authorisation. The Principal Investigator is responsible for ensuring if taking consent is delegated to a designee, the designee is suitably qualified by training or experience to take informed consent.

The right of the participant to refuse to participate without giving reasons must be respected. After the participant has entered the trial the clinician remains free to give alternative treatment to that specified in the protocol at any stage if he/she feels it is in the best interests of the participant, but the reasons for doing so must be recorded. In these cases the participants remain within the study for the purposes of follow-up and data analysis. All participants are free to withdraw at any time from the protocol treatment without giving reasons and without prejudicing further treatment.

An original completed consent form must be retained at each site in the appropriate section of the Investigator Site File, and a copy placed in the patient’s medical records. All patients must be given an original of the signed patient information sheet and consent form for their records. Consent forms must be retained on site and not submitted to the Trials Office.

10.4 Confidentiality

National Health Service Guidelines for storage, transmittal and disclosure of patient information will be followed at all times.

This study will be carried out in accordance with ICH GCP Guidelines. Following formal admission to the study, patient data will be recorded in the hospital case record in the usual way including the circumstances of their entry to the study. Additionally data will be held in study case report forms (CRF). These files will be identified by a trial number and patient initials only.

Representatives from the Study Sponsors and from the regulatory authorities will be given access to the records that relate to the study. They will have full access to the anonymous CRF for the purposes of data validation.

Results of the study may be communicated at scientific meetings and will contribute to the scientific literature. At no time will this be done in such a way that an individual patient may be identified.

10.5 Quality Assurance/Quality Control

Quality Assurance/Quality Control will be maintained by the following requirements and activities:

- All study sites taking part in the trial will be required to participate in site initiation to ensure compliance with the protocol and allow training on study procedures and data collection methods.

- Trial Investigators and site staff must ensure that the trial is conducted in compliance with the protocol, GCP and applicable regulatory requirements.

- The CR-UK CTU, Glasgow will the assist the Trial Investigators and check they are complying with protocol, GCP and regulatory requirements by monitoring trial documentation. Trial data and documentation will be checked for completeness, accuracy and reliability at monitoring visits.

10.6 Monitoring

Central Monitoring
Study sites will be centrally monitored by checking CRFs and other study documents for protocol compliance, data accuracy and completeness.

On-Site and Telephone Monitoring
All UK participating study sites will be monitored remotely by the CR-UK Clinical Trials Unit monitoring team on behalf of the Sponsor(s) by telephone and will also be visited for on site monitoring visit(s). The PI will allow the study staff access to source documents as requested. In addition, the pharmacy department responsible for the trial will be visited to allow monitoring of the pharmacy site file and review of security, storage and accountability of trial drug. Investigators and site staff will be notified in advance about forthcoming monitoring visits. On occasion, members of the CR-UK CTU monitoring team may be accompanied by other trial staff from the unit for training purposes. Where a participating site is using electronic data reporting systems or electronic patient records and hard copies are not available, the CR-UK CTU monitor will require access to a computer for the duration of the visit in order to verify all relevant source data against the case report forms. This may involve being given a temporary log-in. If this is not permitted by local policy, there must be a member of site staff available to provide access to the monitor.

10.7 Audits and Inspections

Trial Investigators must permit trial related monitoring, audits, REC review and regulatory inspections as required, by providing direct access to source data, CRFs and other documents (patients medical records, trial site file, and other pertinent data).

The study may be subject to inspection and audit by NHS GG&C and GU under their remit as Co Sponsors, the CR-UK CTU and other regulatory bodies, i.e. the MHRA, to ensure adherence to GCP, GOG as the lead co-ordinating group for the trial and CTEP as overall legal sponsor of the study.

If an inspection is scheduled at any participating site, the site must notify the CR-UK CTU at the earliest opportunity.

11. Patient Information Sheets and Consent Forms

The UK Patient Information Sheet and Consent Forms will be provided separately for this study. Please check local approval letters to ensure the most current approved Patient Information Sheets and Consent Forms are being used.

12. GP Letter

The UK GP letter will be provided separately for this study. Please check local approval letters to ensure the most current approved GP letter is being used.

13. Translational Research

In this study, tissue specimens will be collected for future translational research. These specimens include a mandatory pre-treatment fresh frozen biopsy and non-mandatory collections of archival formalin fixed paraffin embedded (FFPE) specimen(s), plasma drawn at various points in the patient journey and optional on-treatment/post-progression tumour biopsies. Specimens will be collected from enrolled patients who have consented to the future translational research.

Tissue specimens collected within the translational study (TransLOGS) will be collected and stored in the approved biobank at the Edinburgh Experimental Cancer Medicine Centre. The LOGS Translational Research Committee will assess and approve translational research projects after which samples will be distributed for analysis.

Sample collection, storage and processing:
Detailed instructions for the processing, labelling, handling storage and shipment of these specimens will be provided in the LOGS translational research manual

A table providing a summary of the specimen requirements for the study is detailed on the next page:

Summary of Specimen Requirements

The patient must give permission for her fresh tissue biopsy specimen to be used for translational research. Participating institutions are required to submit the biopsy specimen as outlined below.

If the patient gives permission for her fixed tissue and blood specimens to be used for translational research, then the participating institution is required to submit the patient's specimens as outlined below.

**Required Specimen (Specimen Code)**	**Collection Time Point**	**Ship To**
Fresh **Recurrent Primary** Biopsy (**RRP01 and RRPO2**)[1]	Prior to trial treatment (either arm)<br>**Mandatory (eligibility requirement)**<br>Two cores to be taken, one snap frozen in liquid nitrogen (**RRP01** and second placed into Tissue-Tek Xpress fixative (**RRPO2**)	Send biopsies placed in fixative to Edinburgh Cancer Centre within 1 week of registration, snap frozen biopsies will be batch transferred [2]
FFPE Primary Tumor (FP01)<br>1st Choice: block<br>2nd Choice: 20 unstained slides (10 charged, 5µm + 10 uncharged 10 µm)	Prior to trial treatment (either arm)<br>Optional[3]	Edinburgh Cancer Centre within 8 weeks of registration[2]
FFPE Metastatic Tumor (FM01) 1st Choice: block<br>2nd Choice: 20 unstained slides (10 charged, 5µm + 10 uncharged 10 µm)	Prior to trial treatment (either arm)<br>Optional[3]	
FFPE Recurrent **Primary Tumor** (**FRP01**)<br>1st Choice: block<br>2nd Choice: 20 unstained slides (10 charged, 5µm + 10 uncharged 10 µm)	Prior to trial treatment (either arm)<br>Optional[3]	
FFPE **Recurrent Metastatic** Tumor (**FRM01**)<br>1st Choice: block<br>2nd Choice: 20 unstained slides (10 charged, 5µm + 10 uncharged 10 µm)	Prior to trial treatment (either arm)<br>Optional[3]	
Fresh frozen tissue taken at the time of original diagnosis; if available and stored in liquid nitrogen (**FF01)**	Either study arm; these fresh frozen samples can remain in liquid nitrogen prior to batch transfer (it is accepted that few patients will have archival fresh frozen samples available).<br>Optional [3]	Samples will be batch transferred[2]

Pre-Treatment whole blood (**WBO1**); 10ml of blood drawn into **EDTA tube(s);** this specimen can be taken at the same time as PB01 (below).	Prior to trial treatment (either arm)<br>Optional[3]	Samples will be batch transferred from sites to Edinburgh Cancer Centre periodically e.g. every 6 months or annually depending on recruitment[2]
Pre-Treatment Plasma (PB01) prepared from 7-10mL of blood drawn into **EDTA tube(s)**[4]	Prior to trial treatment (either arm)<br><br>Optional[3]	
C3D1 Pre-Treatment Plasma (PB06) prepared from 7-10mL of blood drawn into **EDTA tube(s)**[4]	Cycle 3, day 1, prior to administering treatment<br>Optional[3]	
C6D1 Pre-Treatment Plasma (PB08) prepared from 7-10mL of blood drawn into **EDTA tube(s)**[4]	Cycle 6, day 1, prior to administering treatment<br>Optional[3]	
Disease Progression Plasma (PB09) prepared from 7-10mL of blood drawn into **EDTA tube(s)**[4]	Within 4 weeks of documented disease progression<br>Optional[3]	
Fresh **Progression Tumour** Biopsy (PTB**01 and PTBO2**)[3]	Within 4 weeks of documented disease progression<br>Optional[3]<br>Two cores to be taken, one snap frozen in liquid nitrogen (**PTB01**) and the second placed into Tissue-Tek Xpress fixative (**PTBO2)**	Send biopsies placed in fixative to Edinburgh Cancer Centre within 1 week of being taken, snap frozen biopsies will be batch transferred[2]

1 Please note the protocol-specific biopsy processing instructions described in the laboratory manual.
2 Please ship all specimens to: Mr Alex MacLellan, Experimental Cancer Medicine Centre, University of Edinburgh Cancer Research UK Centre, Crewe Road South, Edinburgh, EH4 2XR, UK.
3 Centres are encouraged to submit optional specimens wherever possible.
4 Please note the protocol-specific plasma processing instructions described in the laboratory manual

Additional plasma specimens for PK testing will be collected from a subset of patients (approximately 12 US and 12 UK)

Required Specimen (S ecimen Code)	Collection Time Point	Ship To
C1D15 4-8 Hour Post-Treatment (PB02) prepared from 7-10mL of blood drawn into **EDTA tube(s)**[4]	Cycle 1, day 15, 4 to 8 hours (±15 minutes) after administering trametinib	**Samples will be batch transferred from sites every 6 months to Covance in US.**
C1D29 Pre-Treatment Plasma (PB03) prepared from 7-10mL of blood drawn into **EDTA tube(s)**[4]	The last day of cycle 1, prior to administering trametinib	
C1D29 2 Hour Post-Treatment Plasma (PB04) prepared from 7-10mL of blood drawn into **EDTA tube(s)**[4]	The last day of cycle 1, 2 hours (±15 minutes) after administering trametinib	
C2D1 30 Minute Post-Treatment Plasma (PB05) prepared from 7-10mL of blood drawn into **EDTA tube(s)**[4]	Cycle 2, day 1, 30 minutes (±5 minutes) after administering trametinib	
C3D1 30 Minute Post-Treatment Plasma (PB07) prepared from 7-10mL of blood drawn into **EDTA tube(s)**[4]	Cycle 3, day 1, 30 minutes (±5 minutes) after administering trametinib	

1 Please note the protocol-specific plasma processing instructions described in the laboratory manual.

14. Central Radiographic Image Review

Retrospective central radiographic review is planned for this study. It is therefore a requirement for sites to send anonymised scans on disc for patients for all scans the patient receives whilst on study.

Timing of scans

- The timing of the radiological assessments is the same for both arms of the study.
- As outlined within the protocol the scheduled scanning of patients will take place at baseline (must be performed within 28 days of randomisation) and every 8 weeks for the first 15 months then every 3 months thereafter until disease progression (even if the patient withdraws from treatment). Please note scanning should continue after progression in patients who crossover from the standard arm to the Trametinib arm and should continue in that arm until progression.
- Assessments must be performed on a calendar basis (i.e. not delayed due to delays in administering study medication).

Type of scan

- Contrast CT or MRI of chest, abdomen and pelvis.
- A contrast CT scan is the preferred modality.
- The same diagnostic method, including use of contrast when applicable, must be used throughout the study to evaluate each lesion.

Preparation and transfer of scans

- The CR-UK Clinical Trials Unit, Glasgow will provide a batch of CDs for use within the trial before the trial site opens to recruitment. The CR-UK Clinical Trials Unit, Glasgow should be contacted for further supplies.
- Referring to the patient's schedule of assessments, the Research Nurse or Clinical Trial Co-ordinator/Data Manager is aware that scan has been/is to be performed and contacts the radiology department to prompt the scan must be anonymised and downloaded to CD.

- The radiology department prepares an anonymised CD ensuring that:
    - Only the patients trial ID and initials are present
    - The scan is it fit for diagnostic purposes
    - DICOM images and viewing tool is used
    - The date of the scan is clearly identifiable
    - Vertical and/or horizontal scales are included
    - Electronic callipers or an equivalent measurement tool is included

- The Research Nurse or Clinical Trial Co-ordinator/Data Manager makes the patients trial ID, initials, date of scan and " **LOGS:GOG-0281 study**" on the CD, completes *Form 01 Central CT/MRI Scan Assessment Form* and includes in envelope.

- The Research Nurse or Clinical Trial Co-ordinator/Data Manager posts the CD together with *Form 01 Central CT/MRI Scan Assessment Form* to the following address:

  LOGS (GOG-0281) Clinical Trial Co-ordinator
  CR-UK Clinical Trials Unit, Level 0
  The Beatson West of Scotland Cancer Centre
  1053 Great Western Road
  Glasgow, G12 OYN

- The Research Nurse of Clinical Trial Co-ordinator/Data Manager notifies the CR-UK Clinical Trials Unit, Glasgow by email to confirm dispatch of CD and that a delivery should be expected.

Central Review

- The CR-UK Clinical Trials Unit will notify the Research Nurse or Clinical Trial Co-ordinator/Data Manager by email or fax to confirm receipt of anonymised scan and to confirm that is fit to for diagnostic purposes.

14.1 Form 01 Central CT/MRI Scan Assessment Form

CANCER RESEARCH UK<br>Clinical Trials Unit, Glasgow	**Central CT/MRI Scan Assessment Form**

LOGS Trial: A randomised phase II/III study to assess the efficacy of trametinib (GSK1120212) in patients with recurrent or progressive low-grade serous ovarian cancer or peritoneal cancer (GOG-0281)

(EudraCT no: 2013-001627-39)

**Patient Initials:**<br>(forename) ____ (surname) ____	**Patients Trial Identifier:**<br>☐☐☐ / 0 2 8 1 / ☐☐☐<br>Institution No Study No Sequential on Study No

To be completed for every CT/MRI scan performed.

Scans should be sent to: LOGS (GOG-0281) Clinical Trial Co-ordinator, CR-UK Clinical Trials Unit, Level 0, The Beatson West of Scotland Cancer Centre, 1053 Great Western Road, Glasgow, G12 0YN

**Section A – Scan Details** *(To be completed by site and sent with each CT/MRI scan)*	
**Date of CT/MRI Scan:**	☐☐ / ☐☐☐ / ☐☐☐☐<br>Day Month Year
**Scan number:**	☐☐ (i.e. Baseline = 01, 8 weeks =02 etc)
**Type of scan performed:**	☐ Contrast CT (chest, abdomen and pelvis)<br>☐ Non-contrast CT (chest, abdomen and pelvis).<br>If a non-contrast CT has been performed, please state reason below;<br>________________________________<br>☐ MRI scan (chest, abdomen and pelvis)
**Contact details at site:**	Name: ____________<br>Email: ____________<br>Fax: ____________<br>Postal Address: ____________<br>____________<br>____________<br>____________<br>____________
**Section A completed by:**	**Signature:** ____________ **Date**: ____________<br>Form continues on next page

Prior to returning this form to the CR-UK Clinical Trials Unit, please make a copy of the form for retention at site.

CANCER RESEARCH UK<br>Clinical Trials Unit, Glasgow	**Central CT/MRI Scan Assessment Form**

**LOGS Trial: A randomised phase II/III study to assess the efficacy of trametinib (GSK1120212) in patients with recurrent or progressive low-grade serous ovarian cancer or peritoneal cancer (GOG-0281)**<br>**(EudraCT no: 2013-001627-39)**	
**Patient Initials:**<br>(forename) ____ (surname) ____	**Patients Trial Identifier:**<br>☐☐☐ / 0 2 8 1 / ☐☐☐<br>Institution No Study No Sequential on Study No
**Section B – Scan Assessment** *(To be completed by CR-UK Clinical Trials Unit and Central Radiologist)*	
**Date scan received by CR-UK Clinical Trials Unit:**	☐☐ ☐☐☐/ ☐☐☐☐<br>Day Month Year
**Date sent to Central Radiologist:**	☐☐ ☐☐☐/ ☐☐☐☐<br>Day Month Year
**Date received by Central Radiologist:**	☐☐ ☐☐☐/ ☐☐☐☐<br>Day Month Year
**Checks performed by Central Radiologist:**	
**Does the patient trial identifier noted on form match the identifier recorded on CD?**	**Yes** ☐ **No** ☐
**Date of scan on the CD:**	☐☐ ☐☐/☐ ☐☐☐☐<br>Day Month Year
**Do the images on the disc fulfil the requirements of study?**	**Yes** ☐ **No** ☐<br>If no, please state reasons below:<br>____
**Section B completed by Central Radiologist:**	**Signature**: ____________________________ **Date:** ______________
**Date completed form and disc sent back to CR-UK Clinical Trials Unit:**	☐☐/ ☐☐☐ ☐☐☐☐<br>Day Month Year
**Section C – Final Receipt CR-UK Clinical Trials Unit** *(To be completed by CR-UK Clinical Trials Unit)*	
**Date completed form and disc received CR-UK Clinical Trials Unit:**	☐ ☐/ ☐☐☐ ☐☐☐☐<br>Day Month Year
**Section C completed by:**	**Signature**: ____________________________ **Date:** ______________

Final Specification of Statistical Analyses to be Undertaken Relating Clinical Study Data to Translational Biomarker Data for the Primary Clinical Publication

Version 1

James Paul and Austin Miller, November 12, 2020

The biomarker analyses for the paper will be as described in "11.8 Analyses for Integrated Biomarker Objectives" of the protocol NCI Version Date: July 24, 2020 (including amendments 1-20) with the following clarifications and modifications:-

1.1 The analysis of pERK expression is not being undertaken for the primary clinical publication. This will be part of a subsequent publication with its own statistical analysis plan.
1.2 The primary clinical publication will include the biomarker of genotype status within the MAPK pathway, quantified by Whole Exome Sequencing (WES). This dichotomous biomarker will be categorised as mutation present/absent, based on the presence of activating mutations in any of KRAS, BRAF and NRAS. These correspond to the marker expression High/Low categories used to describe analyses in the protocol.
1.3 The effects of the WES biomarker will assessed on both PFS and ORR clinical outcomes, including the multiplicity adjustments (protocol page 98).
1.4 The covariate adjustment used in the multivariable prognostic and predictive models will be for the minimization factors: geographic region (UK or US), performance status (0 or 1), number of prior treatment regimens, and planned treatment regimen (if in the control arm). The last of these was omitted in error from the protocol specified analyses.
1.5 Given the relatively small proportion of patients for whom the WES biomarker is available, the "independent sample modelling" will not be used (see last sentence starting at bottom of page 99 of the protocol). Note that the correlation between the estimated treatment effect in the two biomarker subgroups will be assessed.
1.6 Duration of response and associated swimmer plots will not be part of this analysis (page 100 of protocol).

Note that these modifications and clarifications have been made and agreed prior to any biomarker data being linked to the clinical data set.

The following output will be provided from these analyses:-

2.1 A table showing the distribution of the biomarker in the two randomised study arms.
2.2 An estimate of the magnitude of the PFS hazard ratio interaction (predictive analysis) that can be detected with 80% power at the 5% two-sided level of statistical significance (see table on page 99 of the protocol).
2.3 An estimate of the magnitude of the PFS hazard ratio (prognostic analysis) that can be detected with 80% power at the 5% two-sided level of statistical significance (see table on page 98 of the protocol).
2.4 The minimum detectable Objective Response Rate (MDORR) among patient with favourable biomarker expression that can be detected with 80% power at the 5% two-sided level of statistical significance (see table on page 98 of the protocol).
2.5 A p-value for the interaction/predictive value of the biomarker and treatment arm for PFS.
2.6 The estimate of the treatment PFS hazard ratio and associated 95% confidence intervals separately for the two biomarker groups. These will be plotted in a Forest plot. A Kaplan-Meier plot of PFS in the two study arms within each biomarker group will be produced.
2.7 A p-value for the interaction/predictive value of the biomarker and treatment arm for objective response.
2.8 The estimate of the treatment response odds ratio and associated 95% confidence intervals separately for the two biomarker groups. In addition, the objective response rates in the four groups defined by the combination of

randomised treatment arm and biomarker group will be provided.

2.9 A p-value for the prognostic value of the biomarker for PFS in the control arm together with the associated 95% confidence interval for the hazard ratio. A Kaplan-Meier plot of PFS by biomarker arm for control arm patients will be produced.

2.10 The 95% confidence interval for the corresponding hazard ratio in the trametinib arm. A Kaplan-Meier plot of PFS by biomarker arm for trametinib patients will be produced.

2.11 A p-value for the prognostic value of the biomarker for objective response in the control arm together with the associated 95% confidence interval for the odds ratio.

2.12 The 95% confidence interval for the corresponding odds ratio in the trametinib arm.

Approved by:

	Signature	Date
David M. Gershenson (US Study Chair)		13/Nov/2020
Charles Gourley (UK Study Lead)		13/Nov/2020
Austin Miller (US statistician)		13 Nov 2020
James Paul (UK statistician)		12/Nov/2020
